# Childhood adiposity, serum metabolites and breast density in young women

**DOI:** 10.1186/s13058-022-01588-y

**Published:** 2022-12-19

**Authors:** Joanne F. Dorgan, Heather J. Baer, Kimberly A. Bertrand, Erin S. LeBlanc, Seungyoun Jung, Laurence S. Magder, Linda G. Snetselaar, Victor J. Stevens, Yuji Zhang, Linda Van Horn

**Affiliations:** 1 Division of Cancer Epidemiology, Department of Epidemiology and Public Health, University of Maryland School of Medicine, 660 West Redwood St., Howard Hall, Room 102E, Baltimore, MD 21201 USA; 2 Department of Epidemiology, Harvard T.H. Chan School of Public Health, Boston, MA 02115 USA; 3 Department of Medicine, Harvard Medical School, Boston, MA 02115 USA; 4 Slone Epidemiology Center, Boston University School of Medicine, Boston, MA 02118 USA; 5 Kaiser Permanente Center for Health Research, Portland, OR 97227 USA; 6 Department of Nutritional Science and Food Management, Ewha Womans University, Seoul, South Korea; 7 Graduate Program in System Health Science and Engineering, Ewha Womans University, Seoul, South Korea; 8 Department of Epidemiology, University of Iowa College of Public Health, Iowa City, IA 52242 USA; 9 Department of Preventive Medicine, Northwestern University Feinberg School of Medicine, Chicago, IL 60611 USA; 10 Department of Epidemiology and Public Health, University of Maryland School of Medicine, Baltimore, Maryland 21201 USA

## Abstract

**Background:**

Childhood adiposity is inversely associated with young adult percent dense breast volume (%DBV) and absolute dense breast volume (ADBV), which could contribute to its protective effect for breast cancer later in life. The objective of this study was to identify metabolites in childhood serum that may mediate the inverse association between childhood adiposity and young adult breast density.

**Methods:**

Longitudinal data from 182 female participants in the Dietary Intervention Study in Children (DISC) and the DISC 2006 (DISC06) Follow-Up Study were analyzed. Childhood adiposity was assessed by anthropometry at the DISC visit with serum available that occurred closest to menarche and expressed as a body mass index (BMI) *z*-score. Serum metabolites were measured by untargeted metabolomics using ultra-high-performance liquid chromatography–tandem mass spectrometry. %DBV and ADBV were measured by magnetic resonance imaging at the DISC06 visit when participants were 25–29 years old. Robust mixed effects linear regression was used to identify serum metabolites associated with childhood BMI *z*-scores and breast density, and the R package *mediation* was used to quantify mediation.

**Results:**

Of the 115 metabolites associated with BMI *z*-scores (FDR < 0.20), 4 were significantly associated with %DBV and 6 with ADBV before, though not after, adjustment for multiple comparisons. Mediation analysis identified 2 unnamed metabolites, X-16576 and X-24588, as potential mediators of the inverse association between childhood adiposity and dense breast volume. X-16576 mediated 14% (95% confidence interval (CI) = 0.002, 0.46; *P* = 0.04) of the association of childhood adiposity with %DBV and 11% (95% CI = 0.01, 0.26; *P* = 0.02) of its association with ADBV. X-24588 also mediated 7% (95% CI = 0.001, 0.18; *P* = 0.05) of the association of childhood adiposity with ADBV. None of the other metabolites examined contributed to mediation of the childhood adiposity–%DBV association, though there was some support for contributions of lysine, valine and 7-methylguanine to mediation of the inverse association of childhood adiposity with ADBV.

**Conclusions:**

Additional large longitudinal studies are needed to identify metabolites and other biomarkers that mediate the inverse association of childhood adiposity with breast density and possibly breast cancer risk.

**Supplementary Information:**

The online version contains supplementary material available at 10.1186/s13058-022-01588-y.

## Introduction

Maturation of the breasts occurs during adolescence, and the developing breast may be particularly susceptible to exposures related to later cancer risk. Childhood adiposity is associated with decreased breast cancer risk in adulthood [1–6], as well as decreased percent breast density [7–15]. Breast density is one of the strongest breast cancer risk factors. Risk increases monotonically with increasing density, and women with extremely dense breasts are at a fourfold excess risk of breast cancer compared to those with mostly fatty breasts [16, 17]. Approximately 4.7 million US women aged 40–74 years are estimated to have extremely dense breasts [18]. Results from cohort studies suggest that decreased breast density may mediate, in part, the protective effect of childhood adiposity on breast cancer risk [6, 13].

In our earlier analysis from the Dietary Intervention Study in Children (DISC) and the DISC 2006 (DISC06) Follow-Up Study, adiposity in childhood was significantly inversely associated with dense breast volume measured by magnetic resonance imaging (MRI); women who were heavier as children and adolescents had lower percent dense breast volume (%DBV) and less absolute dense breast volume (ADBV) in young adulthood independent of adult adiposity and several additional covariates [19]. Associations were strongest at the 3-year and 5-year DISC follow-up visits when participants were 11–15 years old, or around the age of menarche, which averaged 12.9 years in this cohort.

Overweight and obesity have a multitude of physiologic effects that could influence breast morphogenesis during childhood and subsequent breast density. To better understand early life determinants of adult breast density, we conducted an exploratory study that used untargeted metabolomics profiling in serum collected in childhood during DISC to identify potential mediators of the association of childhood adiposity with young adult breast density phenotypes measured during the DISC06 Follow-Up Study.

## Materials and methods

### Design

DISC was a multicenter randomized controlled clinical trial sponsored by the National Heart, Lung, and Blood Institute (NHLBI) to test the safety and efficacy of a dietary intervention to reduce serum low-density lipoprotein cholesterol (LDL-C) in children with elevated LDL-C. The trial’s design and results have been described previously [20–23]. Briefly, between 1988 and 1990, 301 healthy, prepubertal 8–10-year-old girls (and 362 boys) with elevated LDL-C were recruited into DISC at 6 clinical centers^1^ and randomized by the data coordinating center^2^ to a behavioral dietary intervention or usual care control group. Planned intervention continued until 1997 when the mean age of participants was 16.7 years. Assent was obtained from DISC participants and informed consent was obtained from parents/guardians prior to randomization. In 2006–2008 when participants were 25 to 29 years old, the DISC06 Follow-Up Study was conducted to evaluate the longer-term effects of the diet intervention on biomarkers associated with breast cancer in DISC female participants [24]. Informed consent was obtained from participants again prior to the DISC06 follow-up visit.

### Participants

DISC participants originally were recruited through schools, health maintenance organizations and pediatric practices. A total of 301 8–10-year-old girls with elevated serum LDL-C who met several additional eligibility criteria were enrolled [20].

All female DISC participants were invited to participate in the DISC06 Follow-Up Study and 260 (86.4%) attended visits. Those who were pregnant or breast feeding at or within 12 weeks before visits (*n* = 30), who had breast augmentation or reduction surgery (*n* = 13), or whose breast MRI was missing or not technically acceptable (*n* = 35) were excluded leaving a total of 182 participants with breast density measurements.

DISC participants provided blood samples on multiple occasions during childhood. Because of limited volume of serum remaining, we measured metabolites in a single sample that had adequate volume (> 0.1 ml) and was collected at the DISC visit that occurred closest in time before or after menarche.

### Data collection

Data and serum were collected previously in DISC or the DISC06 Follow-Up Study. DISC data were collected at baseline, before randomization and annually thereafter. Height and weight were measured, and a brief physical examination including Tanner staging of sexual maturation was performed. Data on demographics, medical history, medication use, physical activity and onset of menses were collected. At baseline, Year-1, Year-3, Year-5 and last visits, a venous blood sample was collected. Girls who were postmenarcheal completed menstrual cycle calendars to estimate the day of the menstrual cycle when blood was collected. At these visits, three non-consecutive 24-h dietary recalls were collected over 2 weeks and averaged to estimate nutrient intake [25].

For the DISC06 Follow-Up Study, participants attended a single visit between 2006 and 2008. Visits occurred within 14 days of onset of next menses whenever possible. Participant data, including demographics, medical and reproductive history, hormonal contraceptive and medication use, and physical activity, were collected on the same day, while 24-h dietary recalls were collected over two weeks.

In both studies, centralized data collection training was conducted prior to data collection. Data were collected by staff masked to treatment group assignment.

#### Anthropometry

Height and weight were measured annually in DISC and again in DISC06 using the same procedures. Height was measured using a stadiometer, and weight was measured on an electronic or beam balance scale. Measurements were taken twice. A third measurement was taken if the first two measurements were not within allowable tolerances (0.5 cm for height and 0.2 kg for weight) and the two closest values were averaged. BMI was calculated as wt(kg)/ht(m^2^) and for DISC visits during childhood expressed as a *z*-score relative to Centers for Disease Control and Prevention (CDC) 2000 Growth Charts [26] to account for changes with age. The BMI *z*-score at the DISC visit when blood used for metabolomics assays was collected was used in all analyses.

#### Blood collection and processing

Blood was collected at DISC and DISC06 follow-up visits in the morning after an overnight fast by venipuncture using standard procedures. Blood was allowed to clot for 45 min at room temperature and centrifuged at 1500×*g* for 20 min before separating serum, which was aliquoted into glass vials in DISC and cryovials in DISC06 and stored continuously at − 80 °C.

#### Breast density assessment

Breast density was measured at the DISC06 follow-up visit using non-contrast MRI. Imaging was performed using a whole-body 1.5 Tesla or higher-field-strength MRI scanner and dedicated breast imaging radiofrequency coil. A standard protocol was followed consisting of a 3D T1-weighted fast gradient echo pulse sequence performed with and without fat suppression and in transaxial and coronal orientations. A 32–40 cm field of view was used for bilateral coverage.

MRI technologists at the clinical centers were individually trained to recognize and correct failures due to incomplete fat suppression, motion artifacts and inadequate breast coverage. Acceptable image quality on 3 volunteers was required for site certification. Participant scans that were inaccurate due to artifacts, motion or technique were excluded (*n* = 21).

All MRI image data were processed at the University of California San Francisco using customized software to identify the chest wall–breast tissue boundary and skin surface, and to separate breast fibroglandular and fatty tissue using a segmentation method based on fuzzy C-means (FCM) clustering [27]. FCM segmentation was performed using fat-suppressed images; nonfat-suppressed images were used when incorrect or failed segmentation occurred due to poor fat suppression. In problematic cases that could not be segmented with automated FCM methods, manual delineation was used.

Separately for each breast, total breast volume and ADBV were measured and absolute non-dense breast volume (ANDBV) was estimated by subtraction. %DBV was calculated as the ratio ADBV:total breast volume × 100. All breast density measures on the two breasts were highly correlated (*r* > 0.94). Results for the two breasts were averaged to provide single measures of %DBV, ADBV and ANDBV for each participant.

### Metabolomics assays

Untargeted metabolomic profiling was performed by Metabolon (Durham, NC). DISC serum samples were randomly ordered with 10% blind quality control (QC) samples integrated throughout to monitor laboratory performance. A pooled matrix sample served as a technical replicate throughout analyses, extracted water samples served as process blanks, and a cocktail of QC standards that was spiked into every sample allowed instrument performance monitoring and aided chromatographic alignment. Forty-two technical replicates from DISC06 samples that had previously been analyzed by Metabolon were re-assayed to facilitate comparison of metabolite levels in serum collected in childhood and young adulthood. A limited dataset with recalibrated levels of named metabolites measured in 180 participants with both DISC and DISC06 samples was created to allow adjustment for adult metabolite levels when analyzing associations of child levels with breast density.

Samples were prepared using the automated MicroLab StAR system (Hamilton Co.). Proteins were precipitated with methanol and the resulting extract was divided into 5 fractions for analysis by four ultra-high-performance liquid chromatography–tandem mass spectrometry (UPLC-MS/MS) methods with one sample reserved for backup. Samples were placed briefly on a TurboVap (Zymark) to remove the organic solvent and were stored overnight under nitrogen before preparation for analysis.

All methods used a Waters ACQUITY ultra-high-performance liquid chromatography and a Thermo Scientific Q-Exactive high-resolution/accurate mass spectrometer interfaced with a heated electrospray ionization (HESI-II) source and Orbitrap mass analyzer operated at 35,000 mass resolution. After drying, sample extracts were reconstituted in solvents compatible with each of the four analytical methods. Details of the methods have been reported previously [28, 29]. Briefly, two aliquots were analyzed under acidic positive ion conditions using a C18 column. One was chromatographically optimized for more hydrophilic compounds, whereas the other chromatographically optimized for more hydrophobic compounds. The third aliquot was analyzed under basic negative ion optimized conditions following gradient elution on a dedicated C18 column. The fourth aliquot also was analyzed under negative ionization following gradient elution from a HILIC column. The MS analysis alternated between MS and data-dependent MS^n^ scans using dynamic exclusion and a scan range of 70–1000 m/z.

Compounds were identified by comparison to library entries of purified standards or recurrent unknowns. Biochemical identifications were based on three criteria: retention index, accurate mass match to the library and the MS/MS forward and reverse scores between the experimental data and authentic standards. At the time DISC samples were analyzed, more than 3300 commercially available purified standard compounds had been acquired and characterized.

Proprietary visualization and interpretation software were used to confirm the consistency of peak identification among samples. Peaks were quantified using area under the curve. A data normalization step corrected for day-to-day variation from instrument tuning differences.

Biochemicals are named according to the following guidelines. Biochemicals without any symbols appended to the end of their name were confirmed based on an authentic chemical standard. Biochemicals with a single asterisk appended to the end of their name were not confirmed based on a standard, but Metabolon is confident in their identify. Biochemicals with a double asterisk appended to the end of their name do not have a standard available, but Metabolon is reasonably confident in their identity. Biochemicals with a number appended to the end of their name are structural isomers of another biochemical in Metabolon’s library.

### Statistical analysis

A total of 880 biochemicals including 650 named biochemicals of known identify and 230 unnamed biochemicals of unknown structural identity were semi-quantified as relative peak intensity by Metabolon. Metabolites with ≥ 30% of values less than the limit of detection or with coefficients of variation ≥ 25% calculated from masked quality control samples were dropped, leaving 571 metabolites for analysis. For metabolites with < 30% of values below the limit of detection, undetected values were imputed at the lowest observed value. Metabolites were transformed to the natural log scale, and extreme values were winsorized using the median absolute deviation [30].

#### Statistical models

**Fig. 1:**
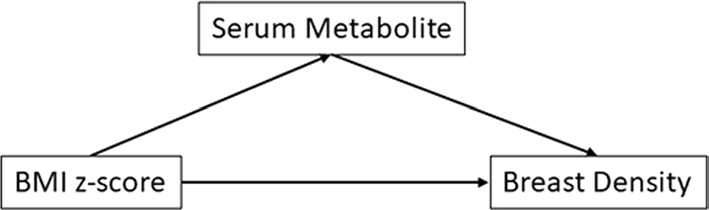
Hypothesized associations among BMI *z*-scores, serum metabolites and breast density. Childhood adiposity (BMI *z*-score) could affect adult breast density directly or serum metabolites could mediate this association

The hypothesized associations among childhood BMI *z*-scores, childhood serum metabolites and young adult breast density phenotypes are shown in Fig. 1. Childhood BMI *z*-score could directly influence adult breast density and/or act indirectly via childhood serum metabolites. We, therefore, conducted a series of analyses to evaluate associations of BMI *z*-scores with breast density and serum metabolites, and serum metabolites with breast density. Associations were evaluated using robust mixed effects multivariable linear regression implemented using the R package *robustlmm* [31]. P-values, which are not reported by *robustlmm,* were estimated by borrowing degrees of freedom (*df*) from the same model fit with R package *lmerTest* [32]. All analyses were conducted with 2 levels of adjustment. Initial models adjusted for age at childhood BMI measurement (years, continuous) and treatment group assignment as fixed effects and DISC clinic as a random effect. When breast density phenotypes were the dependent variables, BMI and BMI^2^ at time of breast density assessment (continuous) also were included as fixed effects. Fully adjusted models with serum metabolites as the dependent variables also included fixed effects for race (white/nonwhite), whereas fixed effects for race, college graduate (yes/no), duration hormone use (continuous), number live births (0/1+) and current smoker (yes/no) also were included when breast density phenotypes were the dependent variables. These covariates were identified by backward stepwise elimination. When serum metabolites were included in models either as dependent or independent variables, menstrual cycle phase at blood collection was adjusted for by including a factor with 4 levels—premenarche/follicular phase/luteal phase/postmenarche unknown phase. Associations of BMI *z*-score with serum metabolites and serum metabolites with breast density were adjusted for multiple comparisons using the Benjamini Hochberg false discovery rate (FDR).

#### BMI z-score and breast density

The association of childhood BMI *z*-score with breast density was assessed separately for %DBV, ADBV and ANDBV. ADBV and ANDBV were natural log transformed prior to analysis. Breast density phenotype was modeled as a continuous dependent variable while childhood BMI *z*-score was included as a continuous fixed effect. Percent difference in ADBV and ANDBV for each unit increase in BMI *z*-score was estimated from the model coefficient for BMI *z*-score as (exp(*β*) − 1) × 100 [33].

#### BMI z-score and serum metabolites

Associations of childhood BMI *z*-score with serum metabolites were evaluated similarly to ADBV and ANDBV except natural log transformed serum metabolite levels were included as the dependent variable. Percent difference in metabolite levels for each unit increase in BMI *z*-score were calculated by back transforming the model coefficient for BMI *z*-score as shown above for ADBV and ANDBV.

#### Serum metabolites and breast density phenotypes

Metabolites that were associated with BMI *z*-score at FDR < 0.20 were evaluated in association with %DBV and ADBV. For these analyses, breast density phenotype was modeled as a continuous dependent variable and natural log transformed serum metabolite levels and BMI *z*-scores were included as continuous fixed effects. The difference in %DBV for a 10% increase in serum metabolite was estimated from the model coefficient for the metabolite as *β* × ln(1.10) [33]. Percent difference in ADBV for a 10% increase in serum metabolite was estimated from the model coefficient for the metabolite as (1.10^β^ − 1) * 100 [33].

To explore the influence of dietary intake of nutrients on associations of nutrient metabolites with breast density phenotypes, average intakes from foods and supplements from three 24-h dietary recalls collected at the DISC visit when blood was collected were included as fixed effects in fully adjusted models described above.

Spearman correlations were used to estimate associations between metabolite levels in serum from childhood (DISC) and adulthood (DISC06) using the limited dataset described under Metabolomics Assays. Models described earlier were refit including fixed effects for both child and adult metabolite levels to evaluate whether these correlations explained associations between child metabolite levels and young adult breast density.

#### Mediation analysis

Mediation analysis was performed using the model-based approach as implemented in R package *mediation* [34]. Because childhood BMI *z*-scores were inversely associated with %DBV and ADBV, mediation analysis was performed for metabolites associated in opposite directions with BMI *z*-score (FDR < 0.20) and these breast density phenotypes (*P* < 0.05). Two multivariable linear regression models were fit for each metabolite–breast density phenotype combination evaluated. The first model included the metabolite as the dependent variable and BMI *z*-score, age, treatment group assignment, race and menstrual cycle phase at blood collection as fixed effects. The second model included the breast density phenotype as the dependent variable and metabolite, BMI *z*-score, age, treatment group assignment and several additional potential confounders measured at the DISC06 visit described above as fixed effects. Mediation was evaluated by applying the function mediate to these two models with BMI *z*-score as the ‘treatment’ and the metabolite as the ‘mediator,’ using bootstrap variances estimated with 5000 simulations.

All tests of statistical significance were two-sided. All analyses were conducted using SAS 9.4 and R 4.1 statistical software.

## Results

Table 1 summarizes participant characteristics during childhood (at the DISC visit when blood used for metabolomic assays was collected) and young adulthood (at the DISC06 follow-up visit). Ninety percent of participants were white, their mean age at blood collection was 11.63 ± 2.13 years, and their mean BMI *z*-score was 0.34 ± 0.93. Participants’ mean age at menarche was 12.90 ± 1.26 years, and 26% were postmenarcheal at the visit. At the DISC06 follow-up visit, participants’ mean age was 27.17 ± 1.02 years and their mean BMI was 25.39 ± 5.36 kg/m^2^. Over half (66%) were college graduates, 71% were nulliparous, 58% were currently using hormonal contraceptives, and 24% were current smokers. Mean breast density phenotypes were 27.64 ± 20.48% for %DBV, 104.18 ± 70.64 cm^3^ for ADBV and 413.25 ± 364.27 cm^3^ for ANDBV.

**Table 1:** Characteristics of participants (*N* = 182)

Child characteristics	Mean (sd)
Age (year)	11.63 (2.13)
BMI Z-score	0.34 (0.93)
Age at menarche (year)	12.90 (1.26)

	*N* (%)
Race	
White	164 (90%)
Nonwhite	18 (10%)
Days until start of next menses at blood collection	
Premenarche	134 (74%)
Postmenarche	
≤ 14 days (luteal)	14 (8%)
> 14 days (follicular)	19 (10%)
Unknown	15 (8%)
Treatment group	
Intervention	87 (48%)
Usual care	95 (52%)

Young adult characteristics	Mean (sd)
Age (year)	27.17 (1.02)
BMI (kg/m^2^)	25.39 (5.36)
Duration hormone use (year)	5.27 (3.65)

	*N* (%)
Education	
High school or equivalent	18 (10%)
Some college	44 (24%)
College graduate	95 (52%)
Graduate school	25 (14%)
Hormonal contraceptive use	
Current	105 (58%)
Former	66 (36%)
Never	11 (6%)
Number live births	
0	129 (71%)
1	30 (16%)
2+	23 (13%)
Smoking status	
Current	44 (24%)
Former	38 (21%)
Never	100 (55%)

Breast phenotypes	Mean (sd)
Breast density (%)	27.64 (20.48)
Dense breast volume (cm^3^)	104.18 (70.64)
Non-dense breast volume (cm^3^)	413.25 (364.27)

### BMI *z*-score and breast density

Associations of BMI *z*-scores with breast density phenotypes are shown in Table 2. BMI *z*-score was significantly inversely associated with %DBV and ADBV. In fully adjusted models, for each unit increase in BMI *z*-score, %DBV decreased by 3.43 (95% CI = − 6.04, − 0.82, *P* = 0.01) while ADBV decreased by 24.44% (95% CI = − 34.27%, − 13.14%, *P* = 0.0001). BMI *z*-score was not significantly associated with ANDBV, and this phenotype was not examined further.

**Table 2:** Association of childhood BMI Z-score with young adult breast density phenotypes

Phenotype	Minimally adjusted model^a^			Fully adjusted model^b^		
	Δ/unit BMI	95% CI	*P*-value	Δ/unit BMI	95% CI	*P*-value
Breast density (%)	− 3.60	− 6.30, − 0.90	9.70E−03	− 3.43	− 6.04, − 0.82	1.09E−02
Dense breast Volume (cm^3^)	− 24.36%	− 34.66%, − 12.43%	2.52E−04	− 24.44%	− 34.27%, − 13.14%	1.18E−04
Non-dense breast volume (cm^3^)	− 8.59%	− 17.02%, 0.69%	7.05E−02	− 8.57%	− 17.04%, 0.78%	7.30E−02

^a^Estimates are from robust mixed effects multivariable linear regression models including breast density phenotype as the dependent variable; childhood BMI *z*-score (continuous), age at childhood BMI measurement (continuous), treatment group assignment, BMI and BMI^2^ at breast density measurement in young adulthood (continuous) as fixed effects; and DISC clinic as a random effect

^b^Estimates are from robust mixed effects multivariable linear regression models as described above under (1) plus fixed effects for race (white/nonwhite), college graduate (yes/no), duration hormone use (continuous), number live births (0/1+) and current smoker (yes/no)

### *BMI *z*-score and serum metabolites*

**Fig. 2:**
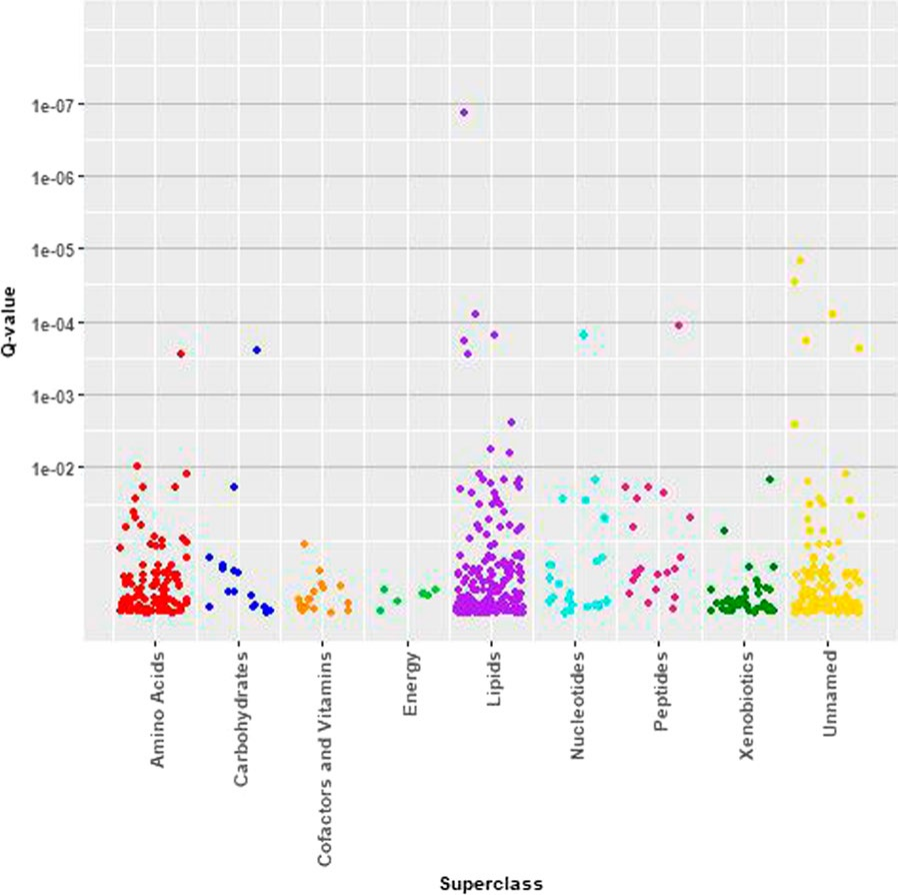
Manhattan plot—Associations of childhood adiposity (BMI *z*-scores) with serum metabolites by metabolite superclass membership

Figure 2 illustrates associations of BMI *z*-scores with serum metabolites from fully adjusted models by metabolite superclass. Detailed results for all metabolites are shown in Additional file 1: Table S1. One hundred and seven known and unknown metabolites representing diverse superclasses were associated with BMI *z*-scores at a FDR < 0.20 in fully adjusted models. Sphingomyelin (d18:2/14:0, d18:1/14:1)*, a lipid, was the most significantly associated (FDR = 7.45e−08); for each one unit increase in BMI *z*-score, sphingomyelin increased by 13.65% (95% CI = 9.49, 17.97).

### Serum metabolites and breast density phenotypes

Associations of %DBV and ADBV with serum metabolites that were associated with childhood BMI Z-score are shown in Tables 3 and 4, respectively. Associations of these metabolites with childhood BMI Z-score also are shown. Detailed results for all metabolites are shown in Additional file 2: Table S2 and Additional file 3: Table S3. Two unnamed metabolites, X-16576 and X-12104, were significantly positively associated with both %DBV and ADBV, while the amino acid lysine was significantly inversely associated with both these breast density phenotypes. For each 10% increase in serum lysine, %DBV decreased by 1.13 (95% CI = − 2.23, − 0.03) and ADBV decreased by 7.68% (95% CI = − 12.76%, − 2.31%). The nucleotide N1-methylinosine also was significantly positively associated with %DBV, while the unnamed metabolite X-24588, the nucleotide 7-methylquanine and the amino acid valine were significantly inversely associated with ADBV.

**Table 3:** Association of %DBV with serum metabolites associated with childhood BMI *z*-score (FDR < 0.20) and association of these metabolites with BMI *Z*-score

Metabolite	Superclass	Difference in %DBV associated with 10% increase in serum metabolite^a^			Percent difference in serum metabolites associated with a unit increase in BMI *z*-score^b^		
		Δ	95% CI	*P*-value	Δ	95% CI	*P*-value
X-16576	Unknown	0.39	0.08, 0.71	1.57E−02	− 13.65	− 21.53, − 4.99	3.01E−03
X-12104	Unknown	0.50	0.03, 0.97	3.88E−02	9.66	3.48, 16.22	2.15E−03
lysine	Amino acid	− 1.13	− 2.23, − 0.03	4.57E−02	3.05	0.50, 5.65	1.98E−02
N1-methylinosine	Nucleotide	0.76	0.01, 1.51	4.83E−02	6.92	2.95, 11.05	6.67E−04
X-15492	Unknown	− 0.31	− 0.64, 0.01	6.11E−02	29.01	17.12, 42.10	6.56E−07
X-17340	Unknown	− 0.31	− 0.66, 0.03	7.96E−02	28.52	17.89, 40.11	5.17E−08
Androstenediol (3beta,17beta) monosulfate (2)	Lipid	− 0.23	− 0.50, 0.04	9.51E−02	33.61	19.66, 49.19	6.96E−07
2-Aminoheptanoate	Lipid	0.37	− 0.06, 0.81	9.60E−02	8.25	0.90, 16.14	2.85E−02

^a^Results shown for metabolites associated with %DBV at *P* < 0.10. Estimates are from robust mixed effects multivariable linear regression models including %DBV as the dependent variable; childhood fixed effects – ln(metabolite level) (continuous), BMI *z*-score (continuous), age at BMI measurement (continuous), treatment group assignment, race (white/nonwhite) and menstrual cycle phase at blood collection (premenarche/luteal/follicular/unknown); adult fixed effects—BMI and BMI^2^ at breast density (continuous), college graduate (yes/no), duration hormone use (continuous), number live births (0/1 +), current smoker (yes/no); and DISC clinic as a random effect

^b^Estimates are from robust mixed effects multivariable linear regression models including ln(metabolite level) (continuous) as the dependent variable; childhood fixed effects—BMI *z*-score (continuous), age at BMI measurement (continuous), treatment group assignment, race (white/nonwhite) and menstrual cycle phase at blood collection (premenarche/luteal/follicular/unknown); and DISC clinic as a random effect

**Table 4:** Association of ADBV with serum metabolites associated with childhood BMI *Z*-score (FDR < 0.20) and association of these metabolites with BMI Z-score

Metabolite	Superclass	Percent difference in ADBV associated with 10% increase in serum metabolite^a^			Percent difference in serum metabolites associated with a unit increase in BMI *z*-score^b^		
		Δ	95% CI	*P*-value	Δ	95% CI	*P*-value
X-16576	Unknown	2.91	1.25, 4.60	6.92E−04	− 13.65	− 21.53, − 4.99	3.01E−03
X-12104	Unknown	3.81	1.24, 6.45	4.06E−03	9.66	3.48, 16.22	2.15E−03
lysine	Amino acid	− 7.68	− 12.76, − 2.31	6.26E−03	3.05	0.50, 5.65	1.98E−02
X-24588	Unknown	− 2.84	− 5.05, − 0.57	1.53E−02	8.18	0.73, 16.19	3.21E−02
7-Methylguanine	Nucleotide	− 5.13	− 9.63, − 0.39	3.57E−02	4.77	1.45, 8.20	5.16E−03
Valine	Amino acid	− 5.28	− 10.01, − 0.31	3.90E−02	4.13	1.65, 6.66	1.20E−03
Homoarginine	Amino acid	− 2.71	− 5.40, 0.06	5.64E−02	7.22	1.63, 13.12	1.16E−02
X-17340	Unknown	− 1.70	− 3.51, 0.14	7.13E−02	28.52	17.89, 40.11	5.17E−08
5alpha-androstan-3alpha,17beta-diol monosulfate (1)	Lipid	0.99	− 0.08, 2.06	7.14E−02	37.90	17.09, 62.41	1.66E−04
N-acetylvaline	Amino acid	− 4.55	− 9.25, 0.39	7.21E−02	3.68	0.63, 6.83	1.87E−02
Glycochenodeoxycholate 3-sulfate	Lipid	− 1.01	− 2.10, 0.10	7.63E−02	− 14.80	− 24.58, − 3.75	1.09E−02
Kynurenine	Amino acid	− 2.72	− 5.76, 0.42	9.10E−02	6.20	1.74, 10.85	6.65E−03

^a^Results shown for metabolites associated with ADBV at *P* < 0.10. Estimates are from robust mixed effects multivariable linear regression models including ADBV as the dependent variable; childhood fixed effects – ln(metabolite level) (continuous), BMI *z*-score (continuous), age at BMI measurement (continuous), treatment group assignment, race (white/nonwhite) and menstrual cycle phase at blood collection (premenarche/luteal/follicular/unknown); adult fixed effects—BMI and BMI^2^ at breast density (continuous), college graduate (yes/no), duration hormone use (continuous), number live births (0/1 +), current smoker (yes/no); and DISC clinic as a random effect

^b^Estimates are from robust mixed effects multivariable linear regression models including ln(metabolite level) (continuous) as the dependent variable; childhood fixed effects—BMI *z*-score (continuous), age at BMI measurement (continuous), treatment group assignment, race (white/nonwhite) and menstrual cycle phase at blood collection (premenarche/luteal/follicular/unknown); and DISC clinic as a random effect

Associations of lysine and valine with %DBV and ADBV were not materially changed after adjustment for dietary intake of the respective amino acid (data not shown). Levels of lysine, valine, N1-methylinosine and 7-methylguanine in serum from DISC and DISC06 were only weakly correlated, and adjustment for DISC06 levels also did not materially alter associations of DISC levels with breast density phenotypes (data not shown). Unnamed metabolites were not measured in DISC06 samples.

### Mediation analysis

Mediation analysis identified the unnamed metabolite X-16576 as a potential mediator of the childhood adiposity–breast density association. This metabolite significantly mediated 14% (95% CI = 0.002, 0.46; *P* = 0.04) of the association with %DBV and 11% (95% CI = 0.01, 0.26; *P* = 0.02) of the association with ADBV. The unnamed metabolite X-24588 also mediated 7% of the childhood adiposity–ADBV association (95% CI = 0.001, 0.18; *P* = 0.05). No other metabolites contributed significantly to mediation of the childhood adiposity–%DBV association. Lysine, valine and the nucleotide 7-methylguanine each mediated 6–7% of the association of childhood adiposity with ADBV, though the effect was only borderline significant.

## Discussion

This analysis confirmed the previously reported inverse association of childhood adiposity with adult breast density and identified two unnamed metabolites, X-16576 and X-24588, as potential mediators of this association. X-16576 significantly mediated 14% of the inverse association of childhood adiposity with %DBV and 11% of its inverse association with ADBV. X-24588 also significantly mediated 7% of the inverse association of childhood adiposity with ADBV. None of the other metabolites examined contributed to mediation of the inverse childhood adiposity–%DBV association, though there was some support for contributions of lysine, valine and 7-methylguanine to mediation of the inverse association of childhood adiposity with ADBV.

To our knowledge, this is the first study to examine associations of untargeted metabolite profiles in serum from childhood with adult breast density phenotypes. Because the goal of the analysis was to identify mediators of the inverse association between childhood adiposity with %DBV and ADBV, only metabolites that were associated with childhood BMI *z*-score were evaluated in association with breast density phenotypes. Nonetheless, we identified several named and unnamed metabolites associated with %DBV and ADBV at *P* < 0.05, though none remained significant after adjusting for multiple comparisons. The amino acid lysine was inversely associated with %DBV and ADBV, and valine was inversely associated with ADBV, as well. However, support for these amino acids mediating the inverse association between childhood adiposity and breast density was weak. Inverse associations of lysine and valine with breast density phenotypes suggest greater uptake from the circulation into tissues where they can be incorporated into proteins, regulate cellular processes or be metabolized. Lysine’s highly reactive terminal ε-amino group contributes importantly to protein stability and makes it a target for numerous posttranslational modifications that alter DNA–protein interactions and transcriptional activity with consequences for cancer, including breast cancer [35]. Collagen is rich in glycated lysine [36] and is a major constituent of dense breast stroma [37]. Extracellular lysine and other essential amino acids including valine are higher in dense compared to non-dense breast tissue where they are available to promote growth and proliferation [38]. Conversely, valine is a branched chain amino acid (BCAA) involved in regulation of protein synthesis, glucose homeostasis and the phosphoinositide 3-kinase-protein kinase B-mammalian target of rapamycin (PI3K-AKT-mTOR) signaling pathway [39]. mTOR regulates cellular proliferation and growth [40], and the PI3K-AKT-mTOR pathway is dysregulated in several cancers including breast cancer [41]. The PI3K-AKT signaling network also has been reported to integrate mechanical and biochemical signaling to control branching morphogenesis of mammary epithelial cells [42]. Specifically, PI3K-AKT is a positive regulator of mammary epithelial cell branching [43].

Few epidemiologic studies have evaluated serum/plasma amino acids with breast density or breast cancer risk. In the Mexican Teachers’ Cohort, lysine and valine in plasma from premenopausal women were not associated with percent mammographic density [44]. Regardless, disruption of BCAA biosynthesis and degradation was associated with breast cancer risk in the Korean Cancer Prevention Study-II [45], and higher plasma levels of valine were associated with increased breast cancer risk in the SU.VI.MAX prospective cohort [46]. Neither lysine nor valine were associated with breast cancer risk in 4 additional prospective cohorts [47–50].

7-Methylguanine was inversely associated with ADBV in our analysis, though similar to the amino acids, support for mediation of the inverse association of childhood adiposity with breast density was weak. 7-methylguanine is a marker of DNA damage caused by endogenous and exogenous methylating agents [51]. It is elevated in the urine of smokers [52–54], though at the time of blood collection in childhood only 2 DISC participants included in the current analysis reported smoking cigarettes. In a study of steel workers, urinary 7-methylguanine also was positively associated with age and inversely associated with BMI and an index of diet quality [53]. 7-Methylguanine also is a marker of RNA turnover and metabolic rate [55]. In the Alpha-Tocopherol Beta-Carotene (ATBC) Cancer Prevention Study, 7-methylguanine was associated with all-cause and cardiovascular disease mortality [56]. We are not aware of any reports of associations of 7-methylguanine with breast density or breast cancer risk.

Our study had several strengths. To ensure scientific rigor and data quality centralized data collection training was conducted prior to DISC and DISC06, and all questionnaire data, anthropometry and breast images were collected by trained personnel according to strict protocols. Serum was collected after an overnight fast and continuously stored at − 80 °C. Metabolites were measured by Metabolon, a leader in the field, using UPLC-MS/MS with inclusion of multiple quality control samples to monitor performance. Breast density was measured by MRI, which is not impaired by high parenchymal breast density, making it especially effective for younger women with dense breast tissue. MRI technologists at the clinical sites were individually trained, and acceptable image quality on 3 volunteers was required for site certification. The median %DBV in DISC06 was 21.9% with a range of 1.5–77.0%. Thus, even though young women, on average, have dense breasts, there was wide variation across women.

Our study also has some limitations. Most notably, our study was exploratory and the sample size of 182 women limited power to detect even moderate associations. Several factors could potentially limit generalizability of results—16% of otherwise eligible participants had missing or technically unacceptable breast MRIs and could not be included in analyses, most participants were Caucasian and well educated and DISC eligibility required elevated LDL-C at baseline. However, similar to others [57], we did not observe an association of LDL-C with breast density in DISC06. DISC was a clinical trial of a diet intervention aimed at blood cholesterol lowering during preadolescence with no expectations of influence on breast density. Regardless, we adjusted for treatment group assignment in all analyses. As a precaution due to the sensitivity of the young breast to radiation, in DISC06 we measured breast density by MRI, while most studies quantify breast density from mammographic images. Because breast density measured by MRI and mammography are highly correlated with *r* ≥ 0.75 [27, 58], associations of metabolites with breast density would not be expected to differ depending on the modality used to measure density. Though we adjusted for several covariates in analysis, uncontrolled confounding cannot be ruled out. Many of the metabolites evaluated, including the two most strongly associated with %DBV and ADBV, were unnamed, which hampers interpretation of results.

## Conclusion

Childhood adiposity is inversely associated with adult breast density. Identification of serum metabolites that mediate this association could lead to discovery of underlying metabolic pathways and improve understanding of breast development in relation to breast density, an established breast cancer risk factor. Support from the current analysis for mediation of the inverse association of childhood adiposity with %DBV and ADBV was strongest for the unnamed metabolites X-16576 and X-24588, while it was more limited for the amino acids lysine and valine and the nucleotide metabolite 7-methylguanine. Larger studies in more diverse populations are needed.

## Supplementary Information

**Additional file 1.** Percent difference in metabolite levels associated with a one unit increase in BMI z-score.

**Additional file 2.** Difference in %DBV associated with a 10% increase in metabolite.

**Additional file 3.** Difference in ADBV associated with a 10% increase in metabolite.

## Data Availability

All data from participants who consented to data sharing will be deposited in the NHLBI biorepository at BioLINCC.

## Abbreviations

ADBV: Absolute dense breast volume
AKT: Protein kinase B
ATBC: Alpha-Tocopherol Beta-Carotene Cancer Prevention Study
BCAA: Branched chain amino acids
BMI: Body mass index
CDC: Centers for Disease Control and Prevention
CI: Confidence interval
DHEAS: Dehydroepiandrosterone sulfate
DISC: Dietary Intervention Study in Children
DISC06: Dietary Intervention Study in Children 2006 Follow-Up Study
DNA: Deoxyribonucleic acid
FCM: Fuzzy c-means
FDR: False discovery rate
HESI-II: Heated electrospray ionization
LDL-C: Low-density lipoprotein cholesterol
MRI: Magnetic resonance imaging
mTOR: Mammalian target of rapamycin
NHLBI: National Heart Lung and Blood Institute
PI3K: Phosphoinositide 3-kinase
QC: Quality control
RIA: Radioimmunoassay
SHBG: Sex hormone-binding globulin
UPLC-MS/MS: Ultra-high-performance liquid chromatography–tandem mass spectroscopy
%DBV: Percent dense breast volume

## Footnotes

1 Children’s Hospital, New Orleans, LA; Johns Hopkins Hospital, Baltimore, MD, Kaiser Permanente Center for Health Research, Portland, OR; University of Medicine and Dentistry of New Jersey, Newark, NJ; Northwestern University Medical School, Chicago, IL; University of Iowa Hospital and Clinics, Iowa City, IA.

2 Maryland Medical Research Institute, Baltimore, MD.
