# Supplementary material for: Childhood adiposity, serum metabolites and breast density in young women
Source: Breast Cancer Res. 2022 Dec 19;24:91. doi: 10.1186/s13058-022-01588-y (PMC9764542; doi:10.1186/s13058-022-01588-y)
Supplement: Supplementary file 1 — Additional file 1. Percent difference in metabolite levels associated with a one unit increase in BMI z-score. [file 13058_2022_1588_MOESM1_ESM.docx]

| **Supplemental Table 1. Percent difference in metabolite levels associated with a one unit increase in BMI z-score** | | | | | | | | | |
| --- | --- | --- | --- | --- | --- | --- | --- | --- | --- |
|  |  | **Minimally Adjusted Model^1^** | | | | **Fully Adjusted Model^2^** | | | |
| **Compid** | **Biochemical** | **Δ** | **95% CI** | **P-value** | **Q-value** | **Δ** | **95% CI** | **P-value** | **Q-value** |
|  | **AMINO ACIDS** |  |  |  |  |  |  |  |  |
|  | **Glycine, Serine and Threonine Metabolism** |  |  |  |  |  |  |  |  |
| c58 | glycine | -0.80 | -3.36, 1.81 | 5.44E-01 | 7.80E-01 | -0.30 | -2.98, 2.45 | 8.28E-01 | 9.34E-01 |
| c27710 | N-acetylglycine | -3.35 | -8.59, 2.20 | 2.33E-01 | 5.19E-01 | -3.11 | -8.66, 2.78 | 2.96E-01 | 6.17E-01 |
| c1516 | sarcosine | 1.40 | -3.25, 6.27 | 5.63E-01 | 7.87E-01 | 2.34 | -2.60, 7.54 | 3.61E-01 | 6.76E-01 |
| c5086 | dimethylglycine | 2.86 | -0.98, 6.85 | 1.49E-01 | 4.10E-01 | 2.72 | -1.34, 6.94 | 1.94E-01 | 4.73E-01 |
| c3141 | betaine | 0.17 | -2.72, 3.16 | 9.08E-01 | 9.69E-01 | -0.02 | -3.04, 3.10 | 9.90E-01 | 9.98E-01 |
| c1648 | serine | 1.01 | -1.87, 3.97 | 4.97E-01 | 7.65E-01 | 1.42 | -1.54, 4.48 | 3.52E-01 | 6.68E-01 |
| c37076 | N-acetylserine | -1.06 | -3.58, 1.53 | 4.20E-01 | 7.01E-01 | -1.06 | -3.66, 1.62 | 4.36E-01 | 7.33E-01 |
| c1284 | threonine | 3.91 | 0.56, 7.36 | 2.28E-02 | 1.29E-01 | 3.83 | 0.35, 7.44 | 3.22E-02 | 1.79E-01 |
| c33939 | N-acetylthreonine | 2.81 | 0.26, 5.42 | 3.16E-02 | 1.59E-01 | 3.23 | 0.68, 5.85 | 1.38E-02 | 1.02E-01 |
|  | **Alanine and Aspartate Metabolism** |  |  |  |  |  |  |  |  |
| c1126 | alanine | 3.54 | 1.15, 5.98 | 3.96E-03 | 4.26E-02 | 3.64 | 1.18, 6.16 | 3.99E-03 | 4.22E-02 |
| c1585 | N-acetylalanine | 2.74 | 0.81, 4.69 | 5.65E-03 | 5.29E-02 | 2.78 | 0.85, 4.75 | 5.14E-03 | 5.08E-02 |
| c443 | aspartate | 6.21 | 1.12, 11.56 | 1.73E-02 | 1.11E-01 | 4.23 | -0.98, 9.71 | 1.15E-01 | 3.53E-01 |
| c22185 | N-acetylaspartate (NAA) | -1.82 | -5.18, 1.66 | 3.02E-01 | 6.03E-01 | -1.87 | -5.38, 1.76 | 3.10E-01 | 6.31E-01 |
| c512 | asparagine | 0.54 | -2.66, 3.84 | 7.46E-01 | 8.97E-01 | 0.79 | -2.55, 4.25 | 6.46E-01 | 8.54E-01 |
| c33942 | N-acetylasparagine | 3.42 | -2.13, 9.29 | 2.34E-01 | 5.19E-01 | 5.35 | -0.41, 11.44 | 7.10E-02 | 2.80E-01 |
|  | **Glutamate Metabolism** |  |  |  |  |  |  |  |  |
| c57 | glutamate | 7.64 | 0.78, 14.98 | 2.99E-02 | 1.54E-01 | 5.50 | -1.56, 13.07 | 1.31E-01 | 3.79E-01 |
| c53 | glutamine | -0.93 | -4.10, 2.34 | 5.72E-01 | 7.87E-01 | -0.26 | -3.60, 3.20 | 8.81E-01 | 9.55E-01 |
| c15720 | N-acetylglutamate | 3.62 | -0.19, 7.58 | 6.48E-02 | 2.40E-01 | 3.23 | -0.70, 7.32 | 1.11E-01 | 3.49E-01 |
| c46225 | pyroglutamine* | 3.12 | -4.26, 11.07 | 4.18E-01 | 7.01E-01 | 1.09 | -6.24, 8.99 | 7.78E-01 | 9.16E-01 |
| c35665 | N-acetyl-aspartyl-glutamate (NAAG) | -0.65 | -7.96, 7.24 | 8.68E-01 | 9.48E-01 | 0.46 | -7.25, 8.82 | 9.11E-01 | 9.70E-01 |
| c54923 | beta-citrylglutamate | 1.20 | -7.41, 10.60 | 7.93E-01 | 9.23E-01 | 1.37 | -7.68, 11.31 | 7.76E-01 | 9.16E-01 |
| c42370 | S-1-pyrroline-5-carboxylate | 3.76 | -2.52, 10.44 | 2.48E-01 | 5.35E-01 | 4.45 | -2.09, 11.42 | 1.89E-01 | 4.72E-01 |
|  | **Histidine Metabolism** |  |  |  |  |  |  |  |  |
| c59 | histidine | 2.66 | 0.05, 5.33 | 4.72E-02 | 2.01E-01 | 2.20 | -0.50, 4.98 | 1.13E-01 | 3.51E-01 |
| c30460 | 1-methylhistidine | 2.71 | -2.62, 8.34 | 3.27E-01 | 6.24E-01 | 2.03 | -3.51, 7.90 | 4.81E-01 | 7.61E-01 |
| c33946 | N-acetylhistidine | -2.94 | -9.08, 3.61 | 3.72E-01 | 6.61E-01 | -2.55 | -8.78, 4.12 | 4.46E-01 | 7.38E-01 |
| c43255 | N-acetyl-1-methylhistidine* | 4.87 | -5.39, 16.24 | 3.67E-01 | 6.58E-01 | 5.23 | -5.46, 17.13 | 3.52E-01 | 6.68E-01 |
| c40473 | hydantoin-5-propionate | 5.97 | -5.76, 19.16 | 3.34E-01 | 6.31E-01 | 5.68 | -6.52, 19.49 | 3.79E-01 | 6.86E-01 |
| c607 | trans-urocanate | -3.17 | -9.69, 3.82 | 3.66E-01 | 6.58E-01 | -2.60 | -9.46, 4.78 | 4.81E-01 | 7.61E-01 |
| c40730 | imidazole propionate | 4.71 | -3.95, 14.15 | 2.98E-01 | 6.03E-01 | 6.78 | -2.41, 16.83 | 1.55E-01 | 4.21E-01 |
| c15716 | imidazole lactate | 2.04 | -2.73, 7.03 | 4.10E-01 | 6.96E-01 | 1.88 | -3.12, 7.14 | 4.70E-01 | 7.61E-01 |
| c43488 | N-acetylcarnosine | 9.23 | 2.93, 15.92 | 4.07E-03 | 4.30E-02 | 10.97 | 4.30, 18.06 | 1.21E-03 | 1.94E-02 |
| c32350 | 1-methyl-4-imidazoleacetate | 2.82 | -1.11, 6.91 | 1.63E-01 | 4.34E-01 | 2.94 | -1.02, 7.05 | 1.49E-01 | 4.11E-01 |
|  | **Lysine Metabolism** |  |  |  |  |  |  |  |  |
| c1301 | lysine | 3.17 | 0.73, 5.66 | 1.15E-02 | 8.32E-02 | 3.05 | 0.50, 5.65 | 1.98E-02 | 1.27E-01 |
| c36752 | N6-acetyllysine | 3.14 | 0.03, 6.34 | 4.91E-02 | 2.01E-01 | 3.08 | -0.18, 6.45 | 6.57E-02 | 2.74E-01 |
| c1498 | N6,N6,N6-trimethyllysine | 4.90 | 0.34, 9.67 | 3.63E-02 | 1.74E-01 | 4.28 | -0.47, 9.25 | 7.98E-02 | 2.90E-01 |
| c15685 | 5-hydroxylysine | 5.73 | 0.20, 11.56 | 4.34E-02 | 1.94E-01 | 7.17 | 1.54, 13.12 | 1.28E-02 | 9.63E-02 |
| c44664 | glutarylcarnitine (C5-DC) | 1.20 | -4.34, 7.07 | 6.79E-01 | 8.56E-01 | 0.21 | -5.55, 6.32 | 9.44E-01 | 9.79E-01 |
| c1444 | pipecolate | 3.71 | -3.70, 11.69 | 3.37E-01 | 6.33E-01 | 3.91 | -3.75, 12.18 | 3.27E-01 | 6.53E-01 |
|  | **Phenylalanine Metabolism** |  |  |  |  |  |  |  |  |
| c64 | phenylalanine | 2.92 | 0.12, 5.80 | 4.22E-02 | 1.91E-01 | 2.46 | -0.43, 5.44 | 9.79E-02 | 3.23E-01 |
| c33950 | N-acetylphenylalanine | 10.90 | 4.61, 17.56 | 6.49E-04 | 1.23E-02 | 12.20 | 5.50, 19.32 | 3.28E-04 | 9.86E-03 |
| c566 | phenylpyruvate | -4.78 | -12.26, 3.33 | 2.42E-01 | 5.27E-01 | -6.22 | -13.93, 2.18 | 1.44E-01 | 4.05E-01 |
| c22130 | phenyllactate (PLA) | 2.43 | -1.34, 6.35 | 2.12E-01 | 4.98E-01 | 2.53 | -1.33, 6.54 | 2.03E-01 | 4.82E-01 |
|  | **Tyrosine Metabolism** |  |  |  |  |  |  |  |  |
| c1299 | tyrosine | 6.20 | 3.35, 9.12 | 2.42E-05 | 9.21E-04 | 6.89 | 3.93, 9.94 | 6.60E-06 | 2.90E-04 |
| c32197 | 3-(4-hydroxyphenyl)lactate (HPLA) | 4.64 | 0.61, 8.83 | 2.49E-02 | 1.37E-01 | 4.44 | 0.19, 8.88 | 4.19E-02 | 2.17E-01 |
| c32553 | phenol sulfate | -0.77 | -7.20, 6.11 | 8.22E-01 | 9.40E-01 | -0.02 | -6.81, 7.27 | 9.95E-01 | 9.98E-01 |
| c1567 | vanillylmandelate (VMA) | -3.28 | -6.83, 0.41 | 8.28E-02 | 2.80E-01 | -3.07 | -6.77, 0.79 | 1.19E-01 | 3.57E-01 |
| c12017 | 3-methoxytyrosine | -0.89 | -5.03, 3.43 | 6.82E-01 | 8.56E-01 | -1.47 | -5.80, 3.05 | 5.18E-01 | 7.87E-01 |
| c48841 | p-cresol glucuronide* | -10.82 | -25.60, 6.90 | 2.17E-01 | 5.08E-01 | -13.26 | -28.18, 4.76 | 1.41E-01 | 4.00E-01 |
| c2761 | thyroxine | -0.90 | -4.08, 2.39 | 5.89E-01 | 7.99E-01 | -0.48 | -3.73, 2.89 | 7.78E-01 | 9.16E-01 |
|  | **Tryptophan Metabolism** |  |  |  |  |  |  |  |  |
| c54 | tryptophan | 1.75 | -0.89, 4.45 | 1.98E-01 | 4.88E-01 | 1.83 | -0.96, 4.70 | 2.03E-01 | 4.82E-01 |
| c33959 | N-acetyltryptophan | 8.88 | 1.47, 16.84 | 1.91E-02 | 1.15E-01 | 10.77 | 2.87, 19.27 | 7.41E-03 | 6.61E-02 |
| c48782 | C-glycosyltryptophan | 1.84 | -0.63, 4.38 | 1.48E-01 | 4.09E-01 | 2.29 | -0.22, 4.86 | 7.58E-02 | 2.87E-01 |
| c37097 | tryptophan betaine | 2.70 | -10.35, 17.66 | 7.01E-01 | 8.61E-01 | 3.34 | -9.91, 18.54 | 6.39E-01 | 8.51E-01 |
| c15140 | kynurenine | 5.54 | 1.15, 10.13 | 1.39E-02 | 9.34E-02 | 6.20 | 1.74, 10.85 | 6.65E-03 | 6.16E-02 |
| c1417 | kynurenate | 10.22 | 4.00, 16.80 | 1.23E-03 | 1.95E-02 | 10.07 | 3.63, 16.90 | 2.11E-03 | 2.76E-02 |
| c15679 | xanthurenate | 13.84 | 3.55, 25.16 | 8.03E-03 | 7.04E-02 | 13.09 | 2.36, 24.94 | 1.66E-02 | 1.14E-01 |
| c2342 | serotonin | -5.85 | -13.26, 2.18 | 1.51E-01 | 4.13E-01 | -6.61 | -14.28, 1.74 | 1.19E-01 | 3.57E-01 |
| c18349 | indolelactate | 2.64 | -1.66, 7.12 | 2.35E-01 | 5.19E-01 | 3.15 | -1.39, 7.90 | 1.79E-01 | 4.57E-01 |
| c27513 | indoleacetate | 2.12 | -3.21, 7.74 | 4.44E-01 | 7.17E-01 | 2.69 | -2.79, 8.48 | 3.44E-01 | 6.63E-01 |
| c32405 | indolepropionate | 2.74 | -7.24, 13.80 | 6.05E-01 | 8.04E-01 | 3.58 | -6.97, 15.32 | 5.22E-01 | 7.88E-01 |
| c27672 | 3-indoxyl sulfate | -1.88 | -8.18, 4.85 | 5.76E-01 | 7.91E-01 | -0.42 | -7.09, 6.73 | 9.05E-01 | 9.66E-01 |
|  | **Leucine, Isoleucine and Valine Metabolism** |  |  |  |  |  |  |  |  |
| c60 | leucine | 3.88 | 1.89, 5.91 | 1.64E-04 | 4.67E-03 | 3.82 | 1.72, 5.96 | 4.29E-04 | 1.22E-02 |
| c22116 | 4-methyl-2-oxopentanoate | -2.47 | -7.97, 3.35 | 3.98E-01 | 6.84E-01 | -2.43 | -8.25, 3.76 | 4.34E-01 | 7.31E-01 |
| c44656 | isovalerate (C5) | 14.87 | 3.11, 27.96 | 1.27E-02 | 8.80E-02 | 12.08 | 0.28, 25.27 | 4.60E-02 | 2.27E-01 |
| c34407 | isovalerylcarnitine (C5) | 6.44 | 0.28, 12.99 | 4.18E-02 | 1.91E-01 | 4.46 | -1.77, 11.08 | 1.66E-01 | 4.37E-01 |
| c12129 | beta-hydroxyisovalerate | 3.36 | -1.50, 8.45 | 1.80E-01 | 4.63E-01 | 3.61 | -1.36, 8.84 | 1.59E-01 | 4.28E-01 |
| c46548 | 3-methylglutarylcarnitine (2) | 4.07 | -3.68, 12.44 | 3.14E-01 | 6.07E-01 | 1.53 | -5.40, 8.97 | 6.74E-01 | 8.79E-01 |
| c1125 | isoleucine | 2.94 | 0.77, 5.16 | 8.46E-03 | 7.10E-02 | 2.83 | 0.57, 5.15 | 1.50E-02 | 1.08E-01 |
| c15676 | 3-methyl-2-oxovalerate | -1.80 | -6.96, 3.64 | 5.09E-01 | 7.71E-01 | -1.99 | -7.47, 3.82 | 4.95E-01 | 7.70E-01 |
| c36746 | 2-hydroxy-3-methylvalerate | -1.17 | -7.06, 5.09 | 7.07E-01 | 8.64E-01 | -0.69 | -6.90, 5.95 | 8.35E-01 | 9.34E-01 |
| c45095 | 2-methylbutyrylcarnitine (C5) | 3.80 | -0.72, 8.52 | 1.02E-01 | 3.23E-01 | 3.46 | -1.24, 8.38 | 1.53E-01 | 4.19E-01 |
| c32397 | 3-hydroxy-2-ethylpropionate | 1.85 | -2.61, 6.52 | 4.23E-01 | 7.01E-01 | 2.32 | -2.36, 7.21 | 3.39E-01 | 6.55E-01 |
| c15765 | ethylmalonate | -0.57 | -6.78, 6.05 | 8.61E-01 | 9.48E-01 | -0.94 | -7.12, 5.65 | 7.74E-01 | 9.16E-01 |
| c53031 | methylsuccinoylcarnitine | -1.01 | -9.00, 7.67 | 8.12E-01 | 9.37E-01 | -1.37 | -9.50, 7.50 | 7.54E-01 | 9.14E-01 |
| c1649 | valine | 4.04 | 1.69, 6.45 | 8.44E-04 | 1.51E-02 | 4.13 | 1.65, 6.66 | 1.20E-03 | 1.94E-02 |
| c1591 | N-acetylvaline | 3.51 | 0.61, 6.49 | 1.84E-02 | 1.13E-01 | 3.68 | 0.63, 6.83 | 1.87E-02 | 1.23E-01 |
| c44526 | 3-methyl-2-oxobutyrate | -4.43 | -9.31, 0.71 | 9.19E-02 | 3.01E-01 | -4.32 | -9.49, 1.15 | 1.21E-01 | 3.60E-01 |
| c33937 | alpha-hydroxyisovalerate | -2.20 | -6.85, 2.68 | 3.71E-01 | 6.61E-01 | -2.31 | -7.13, 2.75 | 3.65E-01 | 6.76E-01 |
| c33441 | isobutyrylcarnitine (C4) | -2.03 | -8.62, 5.03 | 5.63E-01 | 7.87E-01 | -2.93 | -9.75, 4.40 | 4.24E-01 | 7.24E-01 |
|  | **Methionine, Cysteine, SAM and Taurine Metabolism** | |  |  |  |  |  |  |  |
| c1302 | methionine | -0.95 | -3.98, 2.19 | 5.51E-01 | 7.86E-01 | -0.54 | -3.74, 2.76 | 7.45E-01 | 9.13E-01 |
| c1589 | N-acetylmethionine | -5.17 | -20.86, 13.63 | 5.66E-01 | 7.87E-01 | -8.40 | -24.23, 10.73 | 3.66E-01 | 6.76E-01 |
| c2829 | N-formylmethionine | 0.46 | -3.62, 4.72 | 8.29E-01 | 9.41E-01 | 0.59 | -3.63, 4.99 | 7.88E-01 | 9.19E-01 |
| c44878 | methionine sulfone | -0.19 | -5.16, 5.05 | 9.42E-01 | 9.82E-01 | -1.77 | -6.75, 3.48 | 5.03E-01 | 7.76E-01 |
| c18374 | methionine sulfoxide | 8.63 | 1.50, 16.27 | 1.80E-02 | 1.12E-01 | 6.33 | -0.79, 13.95 | 8.43E-02 | 2.97E-01 |
| c45428 | N-acetylmethionine sulfoxide | 5.36 | -9.56, 22.75 | 5.04E-01 | 7.65E-01 | 1.27 | -13.68, 18.81 | 8.77E-01 | 9.54E-01 |
| c42382 | S-adenosylhomocysteine (SAH) | 5.79 | -4.03, 16.60 | 2.59E-01 | 5.50E-01 | 4.54 | -5.55, 15.71 | 3.93E-01 | 6.96E-01 |
| c15705 | cystathionine | 1.15 | -13.63, 18.46 | 8.88E-01 | 9.60E-01 | 1.56 | -13.83, 19.70 | 8.53E-01 | 9.43E-01 |
| c39592 | S-methylcysteine | -6.30 | -12.23, 0.04 | 5.28E-02 | 2.14E-01 | -5.83 | -12.13, 0.92 | 9.06E-02 | 3.06E-01 |
| c22176 | cysteine s-sulfate | 9.28 | -0.94, 20.54 | 7.81E-02 | 2.72E-01 | 10.97 | 0.32, 22.75 | 4.47E-02 | 2.26E-01 |
| c37443 | cysteine sulfinic acid | 5.65 | -0.55, 12.23 | 7.67E-02 | 2.69E-01 | 5.76 | -0.68, 12.62 | 8.26E-02 | 2.93E-01 |
| c590 | hypotaurine | -3.79 | -10.50, 3.42 | 2.96E-01 | 6.03E-01 | -3.70 | -10.80, 3.97 | 3.36E-01 | 6.55E-01 |
| c2125 | taurine | 0.40 | -5.08, 6.20 | 8.88E-01 | 9.60E-01 | 0.07 | -5.75, 6.25 | 9.82E-01 | 9.95E-01 |
|  | **Urea cycle; Arginine and Proline Metabolism** |  |  |  |  |  |  |  |  |
| c1638 | arginine | 3.02 | -0.45, 6.61 | 9.05E-02 | 3.00E-01 | 3.27 | -0.35, 7.02 | 7.92E-02 | 2.90E-01 |
| c1670 | urea | -0.03 | -3.87, 3.96 | 9.87E-01 | 9.97E-01 | -0.48 | -4.44, 3.65 | 8.17E-01 | 9.32E-01 |
| c1493 | ornithine | 1.26 | -3.03, 5.74 | 5.72E-01 | 7.87E-01 | 1.42 | -3.08, 6.12 | 5.44E-01 | 7.98E-01 |
| c55072 | 2-oxoarginine* | -2.06 | -10.09, 6.70 | 6.35E-01 | 8.21E-01 | -1.85 | -10.33, 7.43 | 6.85E-01 | 8.83E-01 |
| c2132 | citrulline | -1.91 | -5.34, 1.65 | 2.91E-01 | 5.95E-01 | -2.39 | -5.99, 1.34 | 2.08E-01 | 4.88E-01 |
| c22137 | homoarginine | 8.05 | 2.54, 13.85 | 4.22E-03 | 4.38E-02 | 7.22 | 1.63, 13.12 | 1.16E-02 | 8.93E-02 |
| c1898 | proline | 4.93 | 1.13, 8.87 | 1.13E-02 | 8.26E-02 | 4.80 | 0.82, 8.95 | 1.89E-02 | 1.23E-01 |
| c36808 | dimethylarginine (ADMA + SDMA) | 0.33 | -1.78, 2.50 | 7.60E-01 | 9.06E-01 | 0.76 | -1.41, 2.99 | 4.94E-01 | 7.70E-01 |
| c33953 | N-acetylarginine | 2.23 | -3.30, 8.08 | 4.38E-01 | 7.17E-01 | 3.24 | -2.45, 9.27 | 2.72E-01 | 5.85E-01 |
| c43249 | N-delta-acetylornithine | 0.77 | -7.90, 10.26 | 8.68E-01 | 9.48E-01 | 1.38 | -7.69, 11.34 | 7.75E-01 | 9.16E-01 |
| c32306 | hydroxyproline | 2.98 | -1.52, 7.69 | 2.00E-01 | 4.88E-01 | 4.33 | -0.31, 9.19 | 6.98E-02 | 2.80E-01 |
| c35127 | prolylhydroxyproline | 8.26 | -10.71, 31.26 | 4.21E-01 | 7.01E-01 | 7.66 | -12.17, 31.97 | 4.78E-01 | 7.61E-01 |
| c37431 | N-methylproline | -3.63 | -18.48, 13.93 | 6.66E-01 | 8.45E-01 | -3.79 | -18.94, 14.19 | 6.59E-01 | 8.69E-01 |
|  | **Creatine Metabolism** |  |  |  |  |  |  |  |  |
| c27718 | creatine | -0.26 | -5.56, 5.35 | 9.27E-01 | 9.75E-01 | 0.82 | -4.79, 6.77 | 7.79E-01 | 9.16E-01 |
| c513 | creatinine | 1.82 | -0.29, 3.99 | 9.34E-02 | 3.03E-01 | 1.84 | -0.33, 4.07 | 9.93E-02 | 3.24E-01 |
|  | **Polyamine Metabolism** |  |  |  |  |  |  |  |  |
| c37496 | N-acetylputrescine | -0.01 | -4.92, 5.16 | 9.98E-01 | 9.98E-01 | 0.55 | -4.56, 5.93 | 8.37E-01 | 9.34E-01 |
| c485 | spermidine | 6.27 | -7.00, 21.43 | 3.73E-01 | 6.61E-01 | 3.92 | -9.52, 19.35 | 5.87E-01 | 8.30E-01 |
| c1419 | 5-methylthioadenosine (MTA) | 6.93 | -6.08, 21.73 | 3.13E-01 | 6.07E-01 | 7.48 | -6.26, 23.24 | 3.03E-01 | 6.23E-01 |
|  | **Guanidino and Acetamido Metabolism** |  |  |  |  |  |  |  |  |
| c15681 | 4-guanidinobutanoate | 7.83 | -1.66, 18.25 | 1.11E-01 | 3.42E-01 | 7.55 | -2.44, 18.57 | 1.45E-01 | 4.05E-01 |
|  | **Glutathione Metabolism** |  |  |  |  |  |  |  |  |
| c35637 | cysteinylglycine | -7.79 | -19.58, 5.72 | 2.46E-01 | 5.33E-01 | -7.95 | -20.43, 6.48 | 2.67E-01 | 5.79E-01 |
| c1494 | 5-oxoproline | 1.46 | -5.80, 9.29 | 7.02E-01 | 8.61E-01 | 1.53 | -5.96, 9.61 | 6.99E-01 | 8.97E-01 |
| c42374 | 2-aminobutyrate | 4.89 | 0.06, 9.94 | 4.85E-02 | 2.01E-01 | 4.50 | -0.47, 9.72 | 7.84E-02 | 2.90E-01 |
| c52281 | 2-hydroxybutyrate/2-hydroxyisobutyrate | 2.45 | -3.25, 8.48 | 4.09E-01 | 6.96E-01 | 3.01 | -2.97, 9.36 | 3.32E-01 | 6.55E-01 |
|  | **PEPTIDE** |  |  |  |  |  |  |  |  |
|  | **Gamma-glutamyl Amino Acid** |  |  |  |  |  |  |  |  |
| c37063 | gamma-glutamylalanine | 13.47 | 4.68, 23.00 | 2.46E-03 | 3.05E-02 | 12.24 | 3.20, 22.07 | 7.73E-03 | 6.72E-02 |
| c36738 | gamma-glutamylglutamate | 13.81 | 2.68, 26.15 | 1.47E-02 | 9.78E-02 | 9.48 | -1.29, 21.42 | 8.82E-02 | 3.02E-01 |
| c2730 | gamma-glutamylglutamine | 7.34 | -1.00, 16.38 | 8.79E-02 | 2.93E-01 | 7.92 | -0.90, 17.51 | 8.14E-02 | 2.90E-01 |
| c33949 | gamma-glutamylglycine | 9.88 | 1.14, 19.37 | 2.71E-02 | 1.45E-01 | 10.19 | 1.07, 20.13 | 2.91E-02 | 1.71E-01 |
| c34456 | gamma-glutamylisoleucine* | 18.52 | 8.55, 29.40 | 2.07E-04 | 5.63E-03 | 16.19 | 6.25, 27.06 | 1.23E-03 | 1.94E-02 |
| c18369 | gamma-glutamylleucine | 14.18 | 6.03, 22.96 | 5.73E-04 | 1.17E-02 | 12.64 | 4.51, 21.40 | 2.17E-03 | 2.76E-02 |
| c55015 | gamma-glutamyl-alpha-lysine | 11.61 | 5.22, 18.38 | 3.41E-04 | 8.41E-03 | 10.60 | 4.16, 17.43 | 1.21E-03 | 1.94E-02 |
| c44872 | gamma-glutamylmethionine | 9.87 | 0.87, 19.67 | 3.23E-02 | 1.60E-01 | 9.11 | -0.19, 19.27 | 5.68E-02 | 2.51E-01 |
| c33422 | gamma-glutamylphenylalanine | 12.40 | 2.62, 23.13 | 1.28E-02 | 8.80E-02 | 9.23 | -0.18, 19.52 | 5.64E-02 | 2.51E-01 |
| c33364 | gamma-glutamylthreonine | 12.09 | 5.42, 19.19 | 3.54E-04 | 8.41E-03 | 11.16 | 4.20, 18.58 | 1.59E-03 | 2.27E-02 |
| c2734 | gamma-glutamyltyrosine | 15.86 | 9.70, 22.37 | 3.72E-07 | 3.04E-05 | 15.78 | 9.36, 22.57 | 1.19E-06 | 1.14E-04 |
| c43829 | gamma-glutamylvaline | 16.62 | 6.98, 27.12 | 6.05E-04 | 1.19E-02 | 13.75 | 4.10, 24.31 | 4.95E-03 | 5.05E-02 |
| c54914 | gamma-glutamylserine | 6.91 | 0.48, 13.76 | 3.62E-02 | 1.74E-01 | 6.21 | -0.37, 13.23 | 6.66E-02 | 2.76E-01 |
|  | **Dipeptide** |  |  |  |  |  |  |  |  |
| c42027 | histidylalanine | 13.34 | -3.72, 33.42 | 1.34E-01 | 3.84E-01 | 11.85 | -5.59, 32.51 | 1.97E-01 | 4.73E-01 |
| c40010 | leucylalanine | 14.52 | -5.03, 38.09 | 1.57E-01 | 4.22E-01 | 18.79 | -2.39, 44.58 | 8.76E-02 | 3.01E-01 |
| c40045 | leucylglycine | 9.58 | -1.45, 21.85 | 9.29E-02 | 3.03E-01 | 6.47 | -4.12, 18.24 | 2.43E-01 | 5.42E-01 |
| c39994 | valylleucine | 11.94 | -5.26, 32.25 | 1.87E-01 | 4.70E-01 | 9.95 | -7.80, 31.13 | 2.93E-01 | 6.14E-01 |
|  | **Acetylated Peptides** |  |  |  |  |  |  |  |  |
| c48425 | phenylacetylcarnitine | -5.46 | -17.67, 8.56 | 4.27E-01 | 7.05E-01 | -5.84 | -18.60, 8.92 | 4.19E-01 | 7.23E-01 |
| c35126 | phenylacetylglutamine | -6.81 | -13.86, 0.82 | 8.06E-02 | 2.77E-01 | -6.49 | -13.91, 1.56 | 1.13E-01 | 3.51E-01 |
| c55017 | 4-hydroxyphenylacetylglutamine | -4.50 | -14.93, 7.22 | 4.37E-01 | 7.17E-01 | -2.11 | -13.12, 10.29 | 7.26E-01 | 9.04E-01 |
|  | **CARBOHYDRATE** |  |  |  |  |  |  |  |  |
|  | **Glycolysis, Gluconeogenesis, and Pyruvate Metabolism** | |  |  |  |  |  |  |  |
| c20675 | 1,5-anhydroglucitol (1,5-AG) | 5.44 | 1.54, 9.49 | 6.46E-03 | 5.95E-02 | 6.74 | 2.69, 10.96 | 1.16E-03 | 1.94E-02 |
| c48152 | glucose | 1.10 | -0.29, 2.50 | 1.24E-01 | 3.70E-01 | 1.49 | 0.05, 2.94 | 4.40E-02 | 2.24E-01 |
| c48990 | pyruvate | -2.90 | -12.80, 8.13 | 5.93E-01 | 8.01E-01 | -1.62 | -12.20, 10.24 | 7.79E-01 | 9.16E-01 |
| c527 | lactate | 1.28 | -3.06, 5.81 | 5.71E-01 | 7.87E-01 | 1.32 | -3.31, 6.17 | 5.83E-01 | 8.27E-01 |
| c1572 | glycerate | 1.59 | -3.37, 6.80 | 5.38E-01 | 7.78E-01 | 1.64 | -3.57, 7.13 | 5.45E-01 | 7.98E-01 |
|  | **Pentose Metabolism** |  |  |  |  |  |  |  |  |
| c15772 | ribitol | 1.20 | -1.05, 3.51 | 2.98E-01 | 6.03E-01 | 1.31 | -1.03, 3.71 | 2.77E-01 | 5.89E-01 |
| c48885 | arabitol/xylitol | 1.12 | -1.92, 4.25 | 4.75E-01 | 7.45E-01 | 1.92 | -1.18, 5.12 | 2.30E-01 | 5.21E-01 |
| c48255 | arabonate/xylonate | 1.27 | -3.75, 6.55 | 6.27E-01 | 8.19E-01 | 1.38 | -3.84, 6.89 | 6.11E-01 | 8.41E-01 |
|  | **Glycogen Metabolism** |  |  |  |  |  |  |  |  |
| c15586 | maltose | -10.58 | -22.53, 3.22 | 1.29E-01 | 3.74E-01 | -13.28 | -25.18, 0.52 | 6.04E-02 | 2.61E-01 |
|  | **Disaccharides and Oligosaccharides** |  |  |  |  |  |  |  |  |
| c1519 | sucrose | -11.24 | -20.58, -0.80 | 3.70E-02 | 1.76E-01 | -10.58 | -20.20, 0.21 | 5.59E-02 | 2.51E-01 |
|  | **Fructose, Mannose and Galactose Metabolism** |  |  |  |  |  |  |  |  |
| c48195 | fructose | 0.47 | -4.90, 6.14 | 8.67E-01 | 9.48E-01 | 0.07 | -5.47, 5.93 | 9.81E-01 | 9.95E-01 |
| c46142 | mannitol/sorbitol | -6.93 | -12.34, -1.20 | 1.97E-02 | 1.16E-01 | -6.86 | -12.63, -0.71 | 3.07E-02 | 1.77E-01 |
| c48153 | mannose | 9.44 | 5.38, 13.65 | 5.61E-06 | 2.47E-04 | 9.58 | 5.48, 13.83 | 5.12E-06 | 2.44E-04 |
| c27719 | galactonate | -19.07 | -33.19, -1.98 | 3.18E-02 | 1.59E-01 | -17.08 | -32.19, 1.41 | 7.00E-02 | 2.80E-01 |
|  | **Aminosugar Metabolism** |  |  |  |  |  |  |  |  |
| c15443 | glucuronate | 1.96 | -2.15, 6.24 | 3.56E-01 | 6.46E-01 | 1.16 | -3.05, 5.56 | 5.96E-01 | 8.36E-01 |
| c42420 | erythronate* | 3.24 | -0.84, 7.49 | 1.23E-01 | 3.69E-01 | 2.57 | -1.57, 6.89 | 2.29E-01 | 5.21E-01 |
|  | **ENERGY** |  |  |  |  |  |  |  |  |
|  | **TCA Cycle** |  |  |  |  |  |  |  |  |
| c1564 | citrate | -2.27 | -5.44, 1.01 | 1.75E-01 | 4.55E-01 | -2.26 | -5.54, 1.12 | 1.89E-01 | 4.72E-01 |
| c528 | alpha-ketoglutarate | -8.52 | -20.23, 4.90 | 2.04E-01 | 4.94E-01 | -7.75 | -19.99, 6.36 | 2.68E-01 | 5.80E-01 |
| c37058 | succinylcarnitine (C4-DC) | 1.98 | -1.92, 6.03 | 3.26E-01 | 6.24E-01 | 2.39 | -1.69, 6.64 | 2.56E-01 | 5.59E-01 |
| c1437 | succinate | 2.17 | -2.28, 6.82 | 3.47E-01 | 6.43E-01 | 2.78 | -1.93, 7.73 | 2.54E-01 | 5.57E-01 |
| c1303 | malate | 0.16 | -4.27, 4.80 | 9.45E-01 | 9.83E-01 | -0.34 | -4.98, 4.52 | 8.88E-01 | 9.59E-01 |
| c52282 | 2-methylcitrate/homocitrate | 2.43 | -2.00, 7.06 | 2.89E-01 | 5.95E-01 | 2.07 | -2.48, 6.83 | 3.79E-01 | 6.86E-01 |
|  | **Oxidative Phosphorylation** |  |  |  |  |  |  |  |  |
| c42109 | phosphate | -5.29 | -10.66, 0.41 | 7.00E-02 | 2.52E-01 | -4.03 | -9.74, 2.05 | 1.91E-01 | 4.72E-01 |
|  | **LIPID** |  |  |  |  |  |  |  |  |
|  | **Medium Chain Fatty Acid** |  |  |  |  |  |  |  |  |
| c32489 | caproate (6:0) | 3.79 | -3.70, 11.87 | 3.32E-01 | 6.30E-01 | 3.70 | -4.08, 12.12 | 3.63E-01 | 6.76E-01 |
| c1644 | heptanoate (7:0) | 3.25 | -4.04, 11.08 | 3.93E-01 | 6.84E-01 | 2.03 | -5.32, 9.94 | 6.00E-01 | 8.37E-01 |
| c32492 | caprylate (8:0) | -1.38 | -5.54, 2.97 | 5.28E-01 | 7.77E-01 | -1.47 | -5.80, 3.05 | 5.18E-01 | 7.87E-01 |
| c1642 | caprate (10:0) | -6.62 | -12.05, -0.84 | 2.65E-02 | 1.43E-01 | -6.49 | -12.20, -0.42 | 3.80E-02 | 2.01E-01 |
| c32497 | 10-undecenoate (11:1n1) | 1.65 | -4.27, 7.93 | 5.94E-01 | 8.01E-01 | 0.84 | -5.27, 7.34 | 7.93E-01 | 9.19E-01 |
| c1645 | laurate (12:0) | -7.51 | -14.24, -0.26 | 4.42E-02 | 1.94E-01 | -6.91 | -13.88, 0.63 | 7.34E-02 | 2.85E-01 |
| c33968 | 5-dodecenoate (12:1n7) | -3.84 | -10.75, 3.60 | 3.04E-01 | 6.03E-01 | -2.73 | -9.78, 4.87 | 4.72E-01 | 7.61E-01 |
|  | **Long Chain Saturated Fatty Acid** |  |  |  |  |  |  |  |  |
| c1365 | myristate (14:0) | -1.70 | -8.71, 5.85 | 6.51E-01 | 8.29E-01 | -0.18 | -7.51, 7.73 | 9.63E-01 | 9.87E-01 |
| c1336 | palmitate (16:0) | -0.03 | -5.75, 6.03 | 9.92E-01 | 9.98E-01 | 1.53 | -4.47, 7.91 | 6.25E-01 | 8.48E-01 |
| c1121 | margarate (17:0) | -1.31 | -6.58, 4.27 | 6.40E-01 | 8.22E-01 | 0.02 | -5.59, 5.96 | 9.94E-01 | 9.98E-01 |
| c1358 | stearate (18:0) | -3.76 | -7.85, 0.50 | 8.48E-02 | 2.85E-01 | -2.67 | -6.97, 1.84 | 2.43E-01 | 5.42E-01 |
|  | **Long Chain Monounsaturated Fatty Acid** |  |  |  |  |  |  |  |  |
| c32418 | myristoleate (14:1n5) | -0.01 | -8.23, 8.95 | 9.98E-01 | 9.98E-01 | 1.67 | -6.73, 10.83 | 7.07E-01 | 9.01E-01 |
| c33447 | palmitoleate (16:1n7) | 2.67 | -6.03, 12.16 | 5.61E-01 | 7.87E-01 | 5.62 | -3.49, 15.58 | 2.37E-01 | 5.34E-01 |
| c33971 | 10-heptadecenoate (17:1n7) | 3.22 | -4.87, 12.00 | 4.48E-01 | 7.17E-01 | 5.77 | -2.81, 15.10 | 1.96E-01 | 4.73E-01 |
| c52285 | oleate/vaccenate (18:1) | -2.29 | -8.83, 4.72 | 5.13E-01 | 7.75E-01 | -0.81 | -7.57, 6.45 | 8.21E-01 | 9.32E-01 |
| c33972 | 10-nonadecenoate (19:1n9) | -1.06 | -7.41, 5.74 | 7.54E-01 | 9.03E-01 | 0.31 | -6.40, 7.49 | 9.31E-01 | 9.72E-01 |
|  | **Long Chain Polyunsaturated Fatty Acid (n3 and n6)** | |  |  |  |  |  |  |  |
| c33969 | stearidonate (18:4n3) | -0.51 | -10.64, 10.77 | 9.26E-01 | 9.75E-01 | 1.88 | -8.68, 13.66 | 7.39E-01 | 9.09E-01 |
| c18467 | eicosapentaenoate (EPA; 20:5n3) | 6.61 | -2.92, 17.09 | 1.82E-01 | 4.67E-01 | 7.76 | -2.18, 18.70 | 1.32E-01 | 3.79E-01 |
| c32504 | docosapentaenoate (DPA; 22:5n3) | 1.03 | -6.43, 9.10 | 7.93E-01 | 9.23E-01 | 2.89 | -4.90, 11.33 | 4.79E-01 | 7.61E-01 |
| c44675 | docosahexaenoate (DHA; 22:6n3) | 2.19 | -4.50, 9.36 | 5.31E-01 | 7.77E-01 | 4.45 | -2.58, 11.98 | 2.23E-01 | 5.13E-01 |
| c1105 | linoleate (18:2n6) | -1.13 | -8.16, 6.45 | 7.64E-01 | 9.07E-01 | 0.88 | -6.52, 8.87 | 8.23E-01 | 9.32E-01 |
| c34035 | linolenate (18:3n3 or 3n6) | 0.50 | -7.59, 9.30 | 9.07E-01 | 9.69E-01 | 1.70 | -6.80, 10.97 | 7.06E-01 | 9.01E-01 |
| c17805 | dihomolinoleate (20:2n6) | -2.62 | -9.22, 4.45 | 4.59E-01 | 7.28E-01 | -1.07 | -8.04, 6.42 | 7.72E-01 | 9.16E-01 |
| c35718 | dihomolinolenate (20:3n3 or 3n6) | 0.75 | -6.38, 8.43 | 8.41E-01 | 9.48E-01 | 2.43 | -5.01, 10.45 | 5.34E-01 | 7.96E-01 |
| c1110 | arachidonate (20:4n6) | 1.34 | -5.92, 9.15 | 7.27E-01 | 8.83E-01 | 2.77 | -4.95, 11.11 | 4.94E-01 | 7.70E-01 |
| c37478 | docosapentaenoate (n6 DPA; 22:5n6) | -2.14 | -8.58, 4.76 | 5.34E-01 | 7.77E-01 | -0.74 | -7.47, 6.49 | 8.37E-01 | 9.34E-01 |
|  | **Fatty Acid, Branched** |  |  |  |  |  |  |  |  |
| c38768 | (14 or 15)-methylpalmitate (a17:0 or i17:0) | -0.04 | -7.64, 8.18 | 9.92E-01 | 9.98E-01 | 2.04 | -5.79, 10.52 | 6.20E-01 | 8.46E-01 |
|  | **Fatty Acid, Dicarboxylate** |  |  |  |  |  |  |  |  |
| c396 | glutarate (C5-DC) | 3.72 | -6.23, 14.72 | 4.79E-01 | 7.49E-01 | 3.44 | -6.84, 14.84 | 5.27E-01 | 7.88E-01 |
| c37253 | 2-hydroxyglutarate | -0.36 | -5.67, 5.25 | 8.98E-01 | 9.64E-01 | -2.09 | -7.40, 3.53 | 4.60E-01 | 7.53E-01 |
| c32398 | sebacate (C10-DC) | -6.86 | -15.93, 3.18 | 1.75E-01 | 4.55E-01 | -8.25 | -17.57, 2.12 | 1.17E-01 | 3.55E-01 |
| c35678 | hexadecanedioate (C16) | -4.66 | -11.13, 2.28 | 1.85E-01 | 4.70E-01 | -3.33 | -10.20, 4.07 | 3.70E-01 | 6.79E-01 |
| c36754 | octadecanedioate (C18) | -9.23 | -15.56, -2.44 | 9.31E-03 | 7.44E-02 | -9.66 | -16.22, -2.58 | 9.07E-03 | 7.50E-02 |
| c39831 | eicosanedioate (C20-DC) | -3.15 | -10.84, 5.19 | 4.48E-01 | 7.17E-01 | -3.51 | -11.56, 5.28 | 4.23E-01 | 7.24E-01 |
| c31787 | 3-carboxy-4-methyl-5-propyl-2-furanpropanoate (CMPF) | 0.57 | -11.03, 13.68 | 9.28E-01 | 9.75E-01 | 2.30 | -10.00, 16.27 | 7.29E-01 | 9.05E-01 |
|  | **Fatty Acid, Amino** |  |  |  |  |  |  |  |  |
| c43761 | 2-aminoheptanoate | 6.65 | -0.31, 14.10 | 6.33E-02 | 2.36E-01 | 8.25 | 0.90, 16.14 | 2.85E-02 | 1.71E-01 |
| c43343 | 2-aminooctanoate | -2.82 | -11.67, 6.91 | 5.57E-01 | 7.87E-01 | -3.56 | -12.74, 6.58 | 4.78E-01 | 7.61E-01 |
|  | **Fatty Acid Metabolism (also BCAA Metabolism)** |  |  |  |  |  |  |  |  |
| c32412 | butyrylcarnitine (C4) | 0.47 | -8.64, 10.50 | 9.22E-01 | 9.75E-01 | 2.20 | -7.27, 12.63 | 6.62E-01 | 8.69E-01 |
| c32452 | propionylcarnitine (C3) | 4.10 | -1.64, 10.17 | 1.67E-01 | 4.41E-01 | 4.80 | -1.22, 11.20 | 1.22E-01 | 3.61E-01 |
| c54907 | hexanoylglutamine | -1.02 | -14.60, 14.72 | 8.92E-01 | 9.60E-01 | 2.33 | -12.15, 19.19 | 7.68E-01 | 9.16E-01 |
| c32198 | acetylcarnitine (C2) | 3.65 | -1.95, 9.57 | 2.07E-01 | 4.96E-01 | 3.14 | -2.71, 9.34 | 3.01E-01 | 6.23E-01 |
| c32328 | hexanoylcarnitine (C6) | 2.38 | -4.82, 10.11 | 5.28E-01 | 7.77E-01 | 1.37 | -6.05, 9.37 | 7.27E-01 | 9.04E-01 |
| c33936 | octanoylcarnitine (C8) | -0.40 | -7.75, 7.54 | 9.19E-01 | 9.75E-01 | -1.32 | -8.70, 6.65 | 7.37E-01 | 9.09E-01 |
| c33941 | decanoylcarnitine (C10) | -0.86 | -8.84, 7.81 | 8.40E-01 | 9.48E-01 | -2.16 | -10.11, 6.50 | 6.15E-01 | 8.42E-01 |
| c34534 | laurylcarnitine (C12) | 0.13 | -7.32, 8.19 | 9.73E-01 | 9.90E-01 | -1.10 | -8.65, 7.07 | 7.85E-01 | 9.19E-01 |
| c33952 | myristoylcarnitine (C14) | 3.62 | -2.13, 9.72 | 2.24E-01 | 5.13E-01 | 2.92 | -2.91, 9.09 | 3.35E-01 | 6.55E-01 |
| c44681 | palmitoylcarnitine (C16) | 5.45 | 0.08, 11.11 | 4.82E-02 | 2.01E-01 | 5.58 | -0.02, 11.49 | 5.23E-02 | 2.42E-01 |
| c38178 | cis-4-decenoylcarnitine (C10:1) | 1.01 | -5.62, 8.12 | 7.71E-01 | 9.12E-01 | 0.60 | -6.18, 7.87 | 8.68E-01 | 9.49E-01 |
| c48182 | myristoleoylcarnitine (C14:1)* | 0.84 | -6.77, 9.06 | 8.35E-01 | 9.46E-01 | 0.38 | -7.36, 8.77 | 9.26E-01 | 9.72E-01 |
| c53223 | palmitoleoylcarnitine (C16:1)* | 5.34 | -0.96, 12.05 | 1.00E-01 | 3.19E-01 | 5.45 | -1.04, 12.37 | 1.04E-01 | 3.34E-01 |
| c35160 | oleoylcarnitine (C18:1) | 1.75 | -3.42, 7.20 | 5.15E-01 | 7.76E-01 | 1.19 | -4.23, 6.92 | 6.73E-01 | 8.79E-01 |
| c46223 | linoleoylcarnitine (C18:2)* | 7.22 | 1.58, 13.17 | 1.24E-02 | 8.71E-02 | 6.45 | 0.66, 12.58 | 2.98E-02 | 1.73E-01 |
| c52988 | adipoylcarnitine (C6-DC) | 1.44 | -7.50, 11.24 | 7.61E-01 | 9.06E-01 | 1.51 | -7.59, 11.52 | 7.54E-01 | 9.14E-01 |
| c53224 | pimeloylcarnitine/3-methyladipoylcarnitine (C7-DC) | -15.42 | -22.91, -7.21 | 5.10E-04 | 1.08E-02 | -15.02 | -22.91, -6.32 | 1.29E-03 | 1.99E-02 |
| c43264 | (R)-3-hydroxybutyrylcarnitine | 8.20 | -7.64, 26.77 | 3.30E-01 | 6.29E-01 | 5.27 | -10.69, 24.07 | 5.41E-01 | 7.98E-01 |
|  | **Carnitine Metabolism** |  |  |  |  |  |  |  |  |
| c36747 | deoxycarnitine | 2.81 | -0.13, 5.83 | 6.25E-02 | 2.35E-01 | 2.25 | -0.81, 5.41 | 1.53E-01 | 4.19E-01 |
| c15500 | carnitine | 5.77 | -0.93, 12.93 | 9.47E-02 | 3.06E-01 | 4.71 | -2.23, 12.16 | 1.90E-01 | 4.72E-01 |
|  | **Fatty Acid Metabolism (Acyl Choline)** |  |  |  |  |  |  |  |  |
| c53257 | palmitoloelycholine | -11.93 | -24.93, 3.32 | 1.21E-01 | 3.65E-01 | -11.88 | -25.23, 3.85 | 1.33E-01 | 3.80E-01 |
| c53262 | dihomo-linolenoyl-choline | -12.77 | -24.29, 0.51 | 6.04E-02 | 2.30E-01 | -13.24 | -25.12, 0.53 | 6.04E-02 | 2.61E-01 |
| c53263 | docosahexaenoylcholine | -11.87 | -22.67, 0.44 | 5.99E-02 | 2.30E-01 | -11.65 | -22.89, 1.22 | 7.60E-02 | 2.87E-01 |
|  | **Fatty Acid, Monohydroxy** |  |  |  |  |  |  |  |  |
| c22036 | 2-hydroxyoctanoate | 1.18 | -5.42, 8.25 | 7.34E-01 | 8.89E-01 | 1.33 | -5.60, 8.76 | 7.16E-01 | 9.03E-01 |
| c42489 | 2-hydroxydecanoate | -1.75 | -6.12, 2.83 | 4.50E-01 | 7.17E-01 | -1.80 | -6.39, 3.00 | 4.56E-01 | 7.49E-01 |
| c35675 | 2-hydroxypalmitate | -0.17 | -3.41, 3.19 | 9.21E-01 | 9.75E-01 | 0.84 | -2.56, 4.35 | 6.34E-01 | 8.51E-01 |
| c17945 | 2-hydroxystearate | -3.82 | -6.94, -0.60 | 2.17E-02 | 1.25E-01 | -2.96 | -6.26, 0.46 | 9.05E-02 | 3.06E-01 |
| c53230 | 3-hydroxyhexanoate | 1.11 | -4.22, 6.73 | 6.90E-01 | 8.56E-01 | 1.39 | -4.14, 7.23 | 6.31E-01 | 8.51E-01 |
| c22001 | 3-hydroxyoctanoate | 1.06 | -4.24, 6.65 | 7.02E-01 | 8.61E-01 | 1.65 | -3.91, 7.54 | 5.69E-01 | 8.16E-01 |
| c22053 | 3-hydroxydecanoate | -0.12 | -6.65, 6.86 | 9.72E-01 | 9.90E-01 | 0.39 | -6.41, 7.69 | 9.14E-01 | 9.70E-01 |
| c32457 | 3-hydroxylaurate | -2.86 | -10.45, 5.38 | 4.86E-01 | 7.54E-01 | -2.43 | -10.27, 6.08 | 5.65E-01 | 8.14E-01 |
| c39609 | 16-hydroxypalmitate | -0.81 | -5.68, 4.30 | 7.50E-01 | 9.00E-01 | 0.09 | -4.85, 5.28 | 9.73E-01 | 9.90E-01 |
|  | **Fatty Acid, Dihydroxy** |  |  |  |  |  |  |  |  |
| c38395 | 12,13-DiHOME | -4.28 | -12.59, 4.83 | 3.47E-01 | 6.43E-01 | -4.50 | -13.16, 5.04 | 3.45E-01 | 6.63E-01 |
|  | **Eicosanoid** |  |  |  |  |  |  |  |  |
| c37536 | 12-HETE | 26.38 | 6.10, 50.53 | 9.48E-03 | 7.44E-02 | 23.24 | 2.49, 48.19 | 2.76E-02 | 1.68E-01 |
|  | **Endocannabinoid** |  |  |  |  |  |  |  |  |
| c52608 | linoleoyl ethanolamide | 7.19 | -13.75, 33.20 | 5.32E-01 | 7.77E-01 | 9.35 | -12.91, 37.29 | 4.43E-01 | 7.38E-01 |
|  | **Inositol Metabolism** |  |  |  |  |  |  |  |  |
| c1124 | myo-inositol | 0.36 | -3.59, 4.48 | 8.59E-01 | 9.48E-01 | 0.36 | -3.79, 4.68 | 8.69E-01 | 9.49E-01 |
|  | **Phospholipid Metabolism** |  |  |  |  |  |  |  |  |
| c15506 | choline | -0.60 | -8.27, 7.71 | 8.83E-01 | 9.58E-01 | -0.73 | -8.66, 7.90 | 8.63E-01 | 9.46E-01 |
| c34396 | phosphocholine | -0.41 | -4.63, 4.00 | 8.53E-01 | 9.48E-01 | -0.69 | -5.01, 3.82 | 7.59E-01 | 9.16E-01 |
| c15990 | glycerophosphorylcholine (GPC) | -0.93 | -6.69, 5.18 | 7.60E-01 | 9.06E-01 | -0.32 | -6.38, 6.13 | 9.21E-01 | 9.70E-01 |
| c40406 | trimethylamine N-oxide | 15.79 | 5.40, 27.21 | 2.59E-03 | 3.05E-02 | 16.48 | 5.54, 28.56 | 2.81E-03 | 3.21E-02 |
|  | **Phosphatidylcholine (PC)** |  |  |  |  |  |  |  |  |
| c19130 | 1,2-dipalmitoyl-GPC (16:0/16:0) | 2.27 | -0.03, 4.63 | 5.45E-02 | 2.18E-01 | 2.10 | -0.29, 4.55 | 8.70E-02 | 3.01E-01 |
| c52470 | 1-palmitoyl-2-palmitoleoyl-GPC (16:0/16:1)* | 6.15 | -1.90, 14.85 | 1.40E-01 | 3.93E-01 | 6.82 | -1.64, 16.02 | 1.19E-01 | 3.57E-01 |
| c52461 | 1-palmitoyl-2-oleoyl-GPC (16:0/18:1) | 0.13 | -2.46, 2.78 | 9.25E-01 | 9.75E-01 | 0.09 | -2.59, 2.84 | 9.50E-01 | 9.81E-01 |
| c42446 | 1-palmitoyl-2-linoleoyl-GPC (16:0/18:2) | 0.81 | -1.66, 3.34 | 5.26E-01 | 7.77E-01 | 0.20 | -2.36, 2.84 | 8.78E-01 | 9.54E-01 |
| c52462 | 1-palmitoyl-2-arachidonoyl-GPC (16:0/20:4n6) | 2.31 | -0.66, 5.38 | 1.30E-01 | 3.74E-01 | 1.69 | -1.37, 4.85 | 2.83E-01 | 5.99E-01 |
| c52438 | 1-stearoyl-2-oleoyl-GPC (18:0/18:1) | -3.65 | -7.86, 0.75 | 1.04E-01 | 3.28E-01 | -3.72 | -8.12, 0.89 | 1.14E-01 | 3.51E-01 |
| c52452 | 1-stearoyl-2-linoleoyl-GPC (18:0/18:2)* | -0.64 | -2.92, 1.69 | 5.89E-01 | 7.99E-01 | -0.98 | -3.37, 1.47 | 4.31E-01 | 7.27E-01 |
| c42450 | 1-stearoyl-2-arachidonoyl-GPC (18:0/20:4) | 1.37 | -1.27, 4.10 | 3.14E-01 | 6.07E-01 | 1.75 | -1.02, 4.59 | 2.20E-01 | 5.10E-01 |
| c52453 | 1-oleoyl-2-linoleoyl-GPC (18:1/18:2)* | -1.14 | -3.51, 1.28 | 3.55E-01 | 6.45E-01 | -1.10 | -3.64, 1.50 | 4.04E-01 | 7.10E-01 |
| c52603 | 1,2-dilinoleoyl-GPC (18:2/18:2) | -6.87 | -10.94, -2.61 | 2.12E-03 | 2.81E-02 | -7.94 | -12.14, -3.53 | 6.63E-04 | 1.49E-02 |
| c53176 | 1-linoleoyl-2-linolenoyl-GPC (18:2/18:3)* | -2.26 | -10.40, 6.61 | 6.06E-01 | 8.04E-01 | -3.22 | -11.58, 5.94 | 4.79E-01 | 7.61E-01 |
| c52710 | 1-linoleoyl-2-arachidonoyl-GPC (18:2/20:4n6)* | 0.30 | -4.00, 4.79 | 8.93E-01 | 9.60E-01 | -0.01 | -4.52, 4.72 | 9.97E-01 | 9.98E-01 |
|  | **Phosphatidylethanolamine (PE)** |  |  |  |  |  |  |  |  |
| c19263 | 1-palmitoyl-2-oleoyl-GPE (16:0/18:1) | 2.01 | -6.16, 10.90 | 6.41E-01 | 8.22E-01 | 2.49 | -5.97, 11.71 | 5.76E-01 | 8.24E-01 |
| c42449 | 1-palmitoyl-2-linoleoyl-GPE (16:0/18:2) | -0.73 | -8.21, 7.35 | 8.54E-01 | 9.48E-01 | -1.62 | -9.17, 6.57 | 6.90E-01 | 8.87E-01 |
| c52464 | 1-palmitoyl-2-arachidonoyl-GPE (16:0/20:4)* | 0.13 | -6.72, 7.49 | 9.71E-01 | 9.90E-01 | -0.34 | -7.35, 7.20 | 9.27E-01 | 9.72E-01 |
| c42448 | 1-stearoyl-2-oleoyl-GPE (18:0/18:1) | 0.67 | -6.48, 8.37 | 8.60E-01 | 9.48E-01 | 0.37 | -6.98, 8.30 | 9.23E-01 | 9.71E-01 |
| c52446 | 1-stearoyl-2-linoleoyl-GPE (18:0/18:2)* | 2.57 | -4.40, 10.05 | 4.81E-01 | 7.49E-01 | 2.02 | -5.00, 9.57 | 5.83E-01 | 8.27E-01 |
| c52447 | 1-stearoyl-2-arachidonoyl-GPE (18:0/20:4) | 0.77 | -5.09, 7.00 | 8.01E-01 | 9.28E-01 | -0.14 | -6.18, 6.29 | 9.65E-01 | 9.87E-01 |
|  | **Phosphatidylinositol (PI)** |  |  |  |  |  |  |  |  |
| c52669 | 1-palmitoyl-2-oleoyl-GPI (16:0/18:1)* | -2.05 | -7.36, 3.56 | 4.67E-01 | 7.38E-01 | -1.72 | -7.18, 4.05 | 5.51E-01 | 8.05E-01 |
| c52450 | 1-palmitoyl-2-linoleoyl-GPI (16:0/18:2) | 1.17 | -4.37, 7.04 | 6.86E-01 | 8.56E-01 | 1.99 | -3.55, 7.84 | 4.90E-01 | 7.70E-01 |
| c52726 | 1-stearoyl-2-oleoyl-GPI (18:0/18:1)* | -7.69 | -12.87, -2.21 | 7.17E-03 | 6.40E-02 | -7.81 | -13.12, -2.17 | 7.95E-03 | 6.78E-02 |
| c52468 | 1-stearoyl-2-linoleoyl-GPI (18:0/18:2) | -4.90 | -8.79, -0.85 | 1.94E-02 | 1.16E-01 | -4.78 | -8.81, -0.58 | 2.75E-02 | 1.68E-01 |
| c52449 | 1-stearoyl-2-arachidonoyl-GPI (18:0/20:4) | -1.61 | -5.21, 2.13 | 3.94E-01 | 6.84E-01 | -0.44 | -4.11, 3.37 | 8.19E-01 | 9.32E-01 |
|  | **Lysophospholipid** |  |  |  |  |  |  |  |  |
| c33955 | 1-palmitoyl-GPC (16:0) | -0.22 | -4.88, 4.68 | 9.30E-01 | 9.76E-01 | -0.06 | -4.91, 5.03 | 9.80E-01 | 9.95E-01 |
| c33230 | 1-palmitoleoyl-GPC* (16:1)* | -0.53 | -6.42, 5.72 | 8.64E-01 | 9.48E-01 | -0.41 | -6.36, 5.92 | 8.96E-01 | 9.60E-01 |
| c47118 | 2-palmitoleoyl-GPC* (16:1)* | 14.13 | -1.31, 32.00 | 7.65E-02 | 2.69E-01 | 15.24 | -0.98, 34.11 | 6.85E-02 | 2.80E-01 |
| c33961 | 1-stearoyl-GPC (18:0) | -2.07 | -7.60, 3.79 | 4.82E-01 | 7.49E-01 | -1.26 | -7.06, 4.90 | 6.82E-01 | 8.81E-01 |
| c48258 | 1-oleoyl-GPC (18:1) | -6.82 | -11.86, -1.50 | 1.37E-02 | 9.29E-02 | -6.47 | -11.72, -0.91 | 2.43E-02 | 1.54E-01 |
| c34419 | 1-linoleoyl-GPC (18:2) | -4.47 | -7.63, -1.20 | 8.38E-03 | 7.10E-02 | -5.09 | -8.37, -1.70 | 3.99E-03 | 4.22E-02 |
| c45951 | 1-linolenoyl-GPC (18:3)* | -2.95 | -8.94, 3.43 | 3.58E-01 | 6.46E-01 | -3.45 | -9.51, 3.02 | 2.90E-01 | 6.12E-01 |
| c33228 | 1-arachidonoyl-GPC* (20:4)* | 1.55 | -3.22, 6.56 | 5.31E-01 | 7.77E-01 | 1.67 | -3.39, 7.00 | 5.25E-01 | 7.88E-01 |
| c49617 | 1-lignoceroyl-GPC (24:0) | -5.27 | -9.91, -0.38 | 3.63E-02 | 1.74E-01 | -5.07 | -9.80, -0.08 | 4.79E-02 | 2.34E-01 |
| c35631 | 1-palmitoyl-GPE (16:0) | -0.52 | -6.39, 5.73 | 8.68E-01 | 9.48E-01 | -0.16 | -6.06, 6.12 | 9.60E-01 | 9.86E-01 |
| c42398 | 1-stearoyl-GPE (18:0) | -2.00 | -8.19, 4.60 | 5.44E-01 | 7.80E-01 | -2.03 | -8.33, 4.69 | 5.45E-01 | 7.98E-01 |
| c35628 | 1-oleoyl-GPE (18:1) | -8.33 | -14.05, -2.22 | 8.92E-03 | 7.28E-02 | -9.14 | -15.03, -2.85 | 5.59E-03 | 5.32E-02 |
| c36600 | 1-linoleoyl-GPE (18:2)* | -3.18 | -9.52, 3.60 | 3.51E-01 | 6.44E-01 | -4.33 | -10.82, 2.63 | 2.19E-01 | 5.10E-01 |
| c35186 | 1-arachidonoyl-GPE (20:4n6)* | 0.61 | -3.98, 5.41 | 7.99E-01 | 9.27E-01 | 0.22 | -4.57, 5.24 | 9.31E-01 | 9.72E-01 |
| c19324 | 1-stearoyl-GPI (18:0) | 4.30 | -1.79, 10.76 | 1.72E-01 | 4.53E-01 | 6.25 | -0.25, 13.18 | 6.15E-02 | 2.62E-01 |
| c36594 | 1-linoleoyl-GPI* (18:2)* | -2.27 | -6.85, 2.55 | 3.51E-01 | 6.44E-01 | -1.94 | -6.72, 3.09 | 4.44E-01 | 7.38E-01 |
| c34214 | 1-arachidonoyl-GPI* (20:4)* | 0.18 | -4.34, 4.91 | 9.41E-01 | 9.82E-01 | 1.25 | -3.52, 6.27 | 6.14E-01 | 8.42E-01 |
|  | **Plasmalogen** |  |  |  |  |  |  |  |  |
| c52477 | 1-(1-enyl-palmitoyl)-2-oleoyl-GPE (P-16:0/18:1)* | -4.05 | -9.38, 1.60 | 1.58E-01 | 4.22E-01 | -5.13 | -10.34, 0.40 | 7.01E-02 | 2.80E-01 |
| c52677 | 1-(1-enyl-palmitoyl)-2-linoleoyl-GPE (P-16:0/18:2)* | -2.00 | -7.90, 4.28 | 5.24E-01 | 7.77E-01 | -2.87 | -8.83, 3.47 | 3.68E-01 | 6.77E-01 |
| c52716 | 1-(1-enyl-palmitoyl)-2-palmitoyl-GPC (P-16:0/16:0)* | 3.68 | 0.06, 7.43 | 4.75E-02 | 2.01E-01 | 3.68 | -0.04, 7.54 | 5.42E-02 | 2.45E-01 |
| c52713 | 1-(1-enyl-palmitoyl)-2-palmitoleoyl-GPC (P-16:0/16:1)* | 5.66 | 0.47, 11.10 | 3.33E-02 | 1.64E-01 | 5.79 | 0.46, 11.40 | 3.44E-02 | 1.87E-01 |
| c52673 | 1-(1-enyl-palmitoyl)-2-arachidonoyl-GPE (P-16:0/20:4)* | 5.79 | -0.56, 12.55 | 7.62E-02 | 2.69E-01 | 5.36 | -0.93, 12.05 | 9.85E-02 | 3.23E-01 |
| c52478 | 1-(1-enyl-palmitoyl)-2-oleoyl-GPC (P-16:0/18:1)* | -2.63 | -5.88, 0.73 | 1.26E-01 | 3.71E-01 | -2.43 | -5.80, 1.07 | 1.73E-01 | 4.49E-01 |
| c52614 | 1-(1-enyl-stearoyl)-2-oleoyl-GPE (P-18:0/18:1) | -6.03 | -10.95, -0.85 | 2.43E-02 | 1.35E-01 | -7.05 | -11.93, -1.90 | 8.57E-03 | 7.19E-02 |
| c52748 | 1-(1-enyl-stearoyl)-2-linoleoyl-GPE (P-18:0/18:2)* | -7.48 | -13.07, -1.53 | 1.55E-02 | 1.02E-01 | -9.31 | -14.84, -3.41 | 2.72E-03 | 3.21E-02 |
| c52689 | 1-(1-enyl-palmitoyl)-2-arachidonoyl-GPC (P-16:0/20:4)* | 3.73 | -0.81, 8.47 | 1.11E-01 | 3.42E-01 | 3.68 | -1.06, 8.65 | 1.32E-01 | 3.79E-01 |
| c52682 | 1-(1-enyl-palmitoyl)-2-linoleoyl-GPC (P-16:0/18:2)* | -1.43 | -4.99, 2.25 | 4.42E-01 | 7.17E-01 | -1.55 | -5.24, 2.28 | 4.23E-01 | 7.24E-01 |
| c52475 | 1-(1-enyl-stearoyl)-2-arachidonoyl-GPE (P-18:0/20:4)* | 0.67 | -5.15, 6.85 | 8.25E-01 | 9.40E-01 | 0.22 | -5.72, 6.52 | 9.45E-01 | 9.79E-01 |
|  | **Lysoplasmalogen** |  |  |  |  |  |  |  |  |
| c39270 | 1-(1-enyl-palmitoyl)-GPE (P-16:0)* | 5.61 | -2.89, 14.85 | 2.04E-01 | 4.94E-01 | 5.96 | -2.89, 15.62 | 1.95E-01 | 4.73E-01 |
| c39271 | 1-(1-enyl-stearoyl)-GPE (P-18:0)* | -2.70 | -10.42, 5.68 | 5.17E-01 | 7.76E-01 | -2.34 | -10.49, 6.55 | 5.95E-01 | 8.36E-01 |
|  | **Glycerolipid Metabolism** |  |  |  |  |  |  |  |  |
| c15122 | glycerol | -0.28 | -8.28, 8.43 | 9.48E-01 | 9.83E-01 | 1.62 | -6.80, 10.80 | 7.16E-01 | 9.03E-01 |
|  | **Monoacylglycerol** |  |  |  |  |  |  |  |  |
| c21184 | 1-oleoylglycerol (18:1) | 1.17 | -7.25, 10.35 | 7.93E-01 | 9.23E-01 | 4.36 | -4.33, 13.83 | 3.37E-01 | 6.55E-01 |
|  | **Diacylglycerol** |  |  |  |  |  |  |  |  |
| c46799 | oleoyl-linoleoyl-glycerol (18:1/18:2) [2] | 7.22 | -1.59, 16.82 | 1.13E-01 | 3.46E-01 | 9.97 | 1.21, 19.50 | 2.61E-02 | 1.64E-01 |
|  | **Sphingolipid Synthesis** |  |  |  |  |  |  |  |  |
| c52605 | sphinganine-1-phosphate | 6.11 | -1.86, 14.74 | 1.38E-01 | 3.91E-01 | 6.53 | -1.84, 15.60 | 1.31E-01 | 3.79E-01 |
|  | **Ceramides** |  |  |  |  |  |  |  |  |
| c44877 | N-palmitoyl-sphingosine (d18:1/16:0) | -0.07 | -2.87, 2.82 | 9.63E-01 | 9.90E-01 | 0.36 | -2.59, 3.41 | 8.13E-01 | 9.32E-01 |
| c54979 | N-stearoyl-sphingosine (d18:1/18:0)* | 8.17 | 3.16, 13.42 | 1.39E-03 | 2.09E-02 | 8.89 | 3.59, 14.47 | 1.01E-03 | 1.86E-02 |
|  | **Hexosylceramides (HCER)** |  |  |  |  |  |  |  |  |
| c53013 | glycosyl-N-palmitoyl-sphingosine (d18:1/16:0) | 3.52 | 0.12, 7.04 | 4.38E-02 | 1.94E-01 | 3.83 | 0.28, 7.52 | 3.58E-02 | 1.93E-01 |
| c52234 | glycosyl-N-stearoyl-sphingosine (d18:1/18:0) | 0.29 | -3.68, 4.41 | 8.90E-01 | 9.60E-01 | 0.45 | -3.73, 4.80 | 8.37E-01 | 9.34E-01 |
|  | **Lactosylceramides (LCER)** |  |  |  |  |  |  |  |  |
| c57370 | lactosyl-N-nervonoyl-sphingosine (d18:1/24:1)* | 0.23 | -3.83, 4.46 | 9.12E-01 | 9.72E-01 | 0.80 | -3.46, 5.25 | 7.18E-01 | 9.03E-01 |
|  | **Dihydrosphingomyelins** |  |  |  |  |  |  |  |  |
| c57365 | myristoyl dihydrosphingomyelin (d18:0/14:0)* | 2.51 | -1.78, 6.99 | 2.57E-01 | 5.48E-01 | 2.55 | -1.95, 7.25 | 2.73E-01 | 5.85E-01 |
| c52434 | palmitoyl dihydrosphingomyelin (d18:0/16:0)* | -1.52 | -4.36, 1.41 | 3.08E-01 | 6.05E-01 | -1.10 | -4.07, 1.96 | 4.77E-01 | 7.61E-01 |
| c57331 | behenoyl dihydrosphingomyelin (d18:0/22:0)* | 10.37 | 3.58, 17.60 | 2.67E-03 | 3.05E-02 | 11.95 | 4.89, 19.47 | 8.38E-04 | 1.65E-02 |
|  | **Sphingomyelins** |  |  |  |  |  |  |  |  |
| c37506 | palmitoyl sphingomyelin (d18:1/16:0) | -1.14 | -3.15, 0.91 | 2.74E-01 | 5.74E-01 | -0.83 | -2.97, 1.35 | 4.53E-01 | 7.45E-01 |
| c19503 | stearoyl sphingomyelin (d18:1/18:0) | 3.55 | 0.34, 6.86 | 3.13E-02 | 1.59E-01 | 3.44 | -0.03, 7.03 | 5.35E-02 | 2.45E-01 |
| c48492 | behenoyl sphingomyelin (d18:1/22:0)* | -0.10 | -2.67, 2.54 | 9.38E-01 | 9.82E-01 | -0.19 | -2.88, 2.57 | 8.92E-01 | 9.60E-01 |
| c52436 | tricosanoyl sphingomyelin (d18:1/23:0)* | -0.09 | -3.11, 3.02 | 9.52E-01 | 9.84E-01 | -0.11 | -3.25, 3.13 | 9.46E-01 | 9.79E-01 |
| c57330 | lignoceroyl sphingomyelin (d18:1/24:0) | -0.68 | -3.93, 2.69 | 6.90E-01 | 8.56E-01 | -0.61 | -4.01, 2.92 | 7.33E-01 | 9.07E-01 |
| c42463 | sphingomyelin (d18:1/14:0, d16:1/16:0)* | 1.33 | -1.72, 4.47 | 3.98E-01 | 6.84E-01 | 1.31 | -1.88, 4.60 | 4.27E-01 | 7.26E-01 |
| c47154 | sphingomyelin (d18:2/14:0, d18:1/14:1)* | 12.30 | 8.15, 16.60 | 8.81E-09 | 5.03E-06 | 13.65 | 9.49, 17.97 | 2.38E-10 | 1.36E-07 |
| c52433 | sphingomyelin (d17:1/16:0, d18:1/15:0, d16:1/17:0)* | 0.05 | -2.76, 2.94 | 9.72E-01 | 9.90E-01 | 0.26 | -2.66, 3.28 | 8.62E-01 | 9.46E-01 |
| c42459 | sphingomyelin (d18:2/16:0, d18:1/16:1)* | 3.67 | 1.49, 5.89 | 1.06E-03 | 1.77E-02 | 3.98 | 1.71, 6.30 | 6.77E-04 | 1.49E-02 |
| c52615 | sphingomyelin (d18:1/17:0, d17:1/18:0, d19:1/16:0) | -0.24 | -3.12, 2.72 | 8.71E-01 | 9.49E-01 | 0.20 | -2.71, 3.20 | 8.94E-01 | 9.60E-01 |
| c37529 | sphingomyelin (d18:1/18:1, d18:2/18:0) | 4.90 | 1.79, 8.10 | 2.11E-03 | 2.81E-02 | 5.65 | 2.40, 9.01 | 7.21E-04 | 1.52E-02 |
| c48490 | sphingomyelin (d18:1/20:0, d16:1/22:0)* | 1.26 | -1.83, 4.45 | 4.29E-01 | 7.05E-01 | 1.53 | -1.73, 4.90 | 3.64E-01 | 6.76E-01 |
| c48491 | sphingomyelin (d18:1/20:1, d18:2/20:0)* | 1.90 | -1.01, 4.90 | 2.05E-01 | 4.94E-01 | 2.54 | -0.48, 5.65 | 1.02E-01 | 3.30E-01 |
| c52495 | sphingomyelin (d18:1/21:0, d17:1/22:0, d16:1/23:0)* | -1.18 | -5.86, 3.73 | 6.32E-01 | 8.21E-01 | -1.31 | -6.11, 3.73 | 6.04E-01 | 8.38E-01 |
| c48493 | sphingomyelin (d18:1/22:1, d18:2/22:0, d16:1/24:1)* | 0.93 | -1.96, 3.90 | 5.35E-01 | 7.77E-01 | 0.77 | -2.23, 3.86 | 6.21E-01 | 8.46E-01 |
| c52435 | sphingomyelin (d18:2/23:0, d18:1/23:1, d17:1/24:1)* | 0.09 | -3.62, 3.95 | 9.61E-01 | 9.90E-01 | 0.63 | -3.19, 4.60 | 7.51E-01 | 9.14E-01 |
| c47153 | sphingomyelin (d18:1/24:1, d18:2/24:0)* | 1.07 | -1.15, 3.34 | 3.49E-01 | 6.44E-01 | 1.57 | -0.74, 3.93 | 1.87E-01 | 4.72E-01 |
| c34445 | sphingosine 1-phosphate | 2.57 | -0.93, 6.19 | 1.54E-01 | 4.18E-01 | 2.62 | -1.05, 6.42 | 1.65E-01 | 4.37E-01 |
|  | **Mevalonate Metabolism** |  |  |  |  |  |  |  |  |
| c531 | 3-hydroxy-3-methylglutarate | -0.11 | -4.95, 4.98 | 9.66E-01 | 9.90E-01 | -0.71 | -5.71, 4.56 | 7.88E-01 | 9.19E-01 |
|  | **Sterol** |  |  |  |  |  |  |  |  |
| c63 | cholesterol | 6.36 | 1.51, 11.44 | 1.05E-02 | 7.76E-02 | 6.21 | 1.18, 11.48 | 1.59E-02 | 1.12E-01 |
| c36776 | 7-HOCA | 2.40 | -1.47, 6.41 | 2.29E-01 | 5.17E-01 | 3.69 | -0.41, 7.96 | 8.01E-02 | 2.90E-01 |
| c36803 | 3beta,7alpha-dihydroxy-5-cholestenoate | 0.05 | -4.33, 4.63 | 9.82E-01 | 9.94E-01 | -0.45 | -5.07, 4.40 | 8.54E-01 | 9.43E-01 |
| c54805 | 3beta-hydroxy-5-cholestenoate | -3.23 | -7.81, 1.57 | 1.86E-01 | 4.70E-01 | -2.58 | -7.36, 2.45 | 3.10E-01 | 6.31E-01 |
|  | **Pregnenolone Steroids** |  |  |  |  |  |  |  |  |
| c38170 | pregnenolone sulfate | 4.26 | -4.95, 14.35 | 3.78E-01 | 6.68E-01 | 4.67 | -4.89, 15.19 | 3.52E-01 | 6.68E-01 |
| c46115 | 21-hydroxypregnenolone disulfate | 9.17 | 1.02, 17.97 | 2.80E-02 | 1.48E-01 | 8.73 | 0.20, 17.99 | 4.61E-02 | 2.27E-01 |
| c32619 | pregnenediol sulfate (C21H34O5S)* | 8.47 | 0.09, 17.54 | 4.91E-02 | 2.01E-01 | 7.49 | -1.23, 16.98 | 9.61E-02 | 3.21E-01 |
| c32562 | pregnen-diol disulfate* | 9.19 | -0.94, 20.36 | 7.85E-02 | 2.72E-01 | 7.69 | -2.79, 19.29 | 1.58E-01 | 4.28E-01 |
|  | **Progestin Steroids** |  |  |  |  |  |  |  |  |
| c37196 | 5alpha-pregnan-3beta,20beta-diol monosulfate (1) | 23.84 | 11.24, 37.86 | 1.34E-04 | 4.13E-03 | 22.10 | 9.41, 36.26 | 4.70E-04 | 1.24E-02 |
| c37200 | 5alpha-pregnan-3beta,20alpha-diol monosulfate (2) | 16.34 | 4.13, 29.98 | 8.14E-03 | 7.04E-02 | 13.19 | 0.65, 27.28 | 4.00E-02 | 2.10E-01 |
| c37198 | 5alpha-pregnan-3beta,20alpha-diol disulfate | 12.10 | 0.58, 24.94 | 4.03E-02 | 1.87E-01 | 10.99 | -1.01, 24.45 | 7.57E-02 | 2.87E-01 |
| c46172 | 5alpha-pregnan-diol disulfate | 20.64 | 3.34, 40.85 | 1.86E-02 | 1.13E-01 | 17.27 | -0.06, 37.62 | 5.25E-02 | 2.42E-01 |
| c40708 | pregnanediol-3-glucuronide | 20.08 | 5.85, 36.22 | 4.98E-03 | 4.73E-02 | 17.10 | 2.80, 33.38 | 1.86E-02 | 1.23E-01 |
|  | **Corticosteroids** |  |  |  |  |  |  |  |  |
| c1712 | cortisol | -9.40 | -15.91, -2.38 | 1.03E-02 | 7.76E-02 | -10.08 | -16.70, -2.93 | 7.16E-03 | 6.49E-02 |
| c1769 | cortisone | -6.74 | -10.80, -2.51 | 2.41E-03 | 3.05E-02 | -6.19 | -10.45, -1.73 | 7.77E-03 | 6.72E-02 |
|  | **Androgenic Steroids** |  |  |  |  |  |  |  |  |
| c32425 | dehydroepiandrosterone sulfate (DHEA-S) | 18.07 | 7.89, 29.21 | 3.98E-04 | 9.08E-03 | 17.43 | 6.98, 28.89 | 8.95E-04 | 1.70E-02 |
| c38168 | 16a-hydroxy DHEA 3-sulfate | 25.04 | 8.40, 44.24 | 2.50E-03 | 3.05E-02 | 26.83 | 9.32, 47.15 | 2.02E-03 | 2.76E-02 |
| c33973 | epiandrosterone sulfate | 19.58 | 6.58, 34.17 | 2.67E-03 | 3.05E-02 | 18.20 | 4.90, 33.19 | 6.69E-03 | 6.16E-02 |
| c31591 | androsterone sulfate | 12.97 | 0.17, 27.40 | 4.83E-02 | 2.01E-01 | 13.68 | 0.12, 29.09 | 4.95E-02 | 2.36E-01 |
| c47112 | etiocholanolone glucuronide | 15.70 | 0.48, 33.23 | 4.41E-02 | 1.94E-01 | 15.39 | -0.52, 33.86 | 6.03E-02 | 2.61E-01 |
| c37211 | androstenediol (3beta,17beta) monosulfate (1) | 34.05 | 20.14, 49.57 | 4.43E-07 | 3.16E-05 | 32.72 | 18.52, 48.63 | 2.16E-06 | 1.54E-04 |
| c37210 | androstenediol (3beta,17beta) monosulfate (2) | 33.60 | 20.32, 48.34 | 1.88E-07 | 2.20E-05 | 33.61 | 19.66, 49.19 | 6.96E-07 | 7.95E-05 |
| c37202 | androstenediol (3beta,17beta) disulfate (1) | 27.78 | 14.63, 42.43 | 1.68E-05 | 6.85E-04 | 26.77 | 13.15, 42.02 | 6.56E-05 | 2.50E-03 |
| c37203 | androstenediol (3beta,17beta) disulfate (2) | 21.91 | 12.65, 31.93 | 1.99E-06 | 1.13E-04 | 21.64 | 11.96, 32.15 | 7.14E-06 | 2.91E-04 |
| c37207 | androstenediol (3alpha, 17alpha) monosulfate (2) | 26.48 | 14.76, 39.40 | 4.47E-06 | 2.13E-04 | 28.06 | 15.82, 41.59 | 3.02E-06 | 1.90E-04 |
| c37209 | androstenediol (3alpha, 17alpha) monosulfate (3) | 15.35 | 5.75, 25.82 | 1.52E-03 | 2.17E-02 | 16.07 | 5.97, 27.14 | 1.59E-03 | 2.27E-02 |
| c37186 | 5alpha-androstan-3alpha,17beta-diol monosulfate (1) | 36.12 | 16.57, 58.95 | 1.38E-04 | 4.13E-03 | 37.90 | 17.09, 62.41 | 1.66E-04 | 5.57E-03 |
| c37190 | 5alpha-androstan-3beta,17beta-diol disulfate | 31.32 | 15.07, 49.86 | 7.83E-05 | 2.63E-03 | 30.88 | 13.92, 50.35 | 1.99E-04 | 6.31E-03 |
| c32827 | andro steroid monosulfate C19H28O6S (1)* | 22.33 | 6.68, 40.27 | 4.38E-03 | 4.39E-02 | 24.49 | 8.05, 43.43 | 2.81E-03 | 3.21E-02 |
|  | **Primary Bile Acid Metabolism** |  |  |  |  |  |  |  |  |
| c22842 | cholate | 0.45 | -13.49, 16.64 | 9.53E-01 | 9.84E-01 | -2.25 | -16.15, 13.97 | 7.72E-01 | 9.16E-01 |
| c18476 | glycocholate | -1.73 | -15.81, 14.69 | 8.25E-01 | 9.40E-01 | -6.43 | -20.59, 10.26 | 4.29E-01 | 7.26E-01 |
| c1563 | chenodeoxycholate | 1.45 | -12.99, 18.29 | 8.55E-01 | 9.48E-01 | 0.55 | -13.92, 17.45 | 9.45E-01 | 9.79E-01 |
| c32346 | glycochenodeoxycholate | 1.47 | -13.32, 18.79 | 8.56E-01 | 9.48E-01 | -2.87 | -17.48, 14.32 | 7.27E-01 | 9.04E-01 |
| c18494 | taurochenodeoxycholate | -4.51 | -20.08, 14.08 | 6.11E-01 | 8.07E-01 | -6.31 | -21.98, 12.51 | 4.86E-01 | 7.67E-01 |
| c52983 | glycochenodeoxycholate glucuronide (1) | 24.30 | 7.09, 44.27 | 4.73E-03 | 4.57E-02 | 21.59 | 4.51, 41.45 | 1.22E-02 | 9.33E-02 |
| c52974 | glycochenodeoxycholate 3-sulfate | -13.41 | -23.00, -2.62 | 1.73E-02 | 1.11E-01 | -14.80 | -24.58, -3.75 | 1.09E-02 | 8.53E-02 |
|  | **Secondary Bile Acid Metabolism** |  |  |  |  |  |  |  |  |
| c32620 | glycolithocholate sulfate* | -4.40 | -16.05, 8.88 | 4.99E-01 | 7.65E-01 | -6.34 | -18.31, 7.38 | 3.49E-01 | 6.68E-01 |
| c36850 | taurolithocholate 3-sulfate | -5.80 | -17.96, 8.18 | 3.99E-01 | 6.84E-01 | -9.23 | -21.40, 4.82 | 1.89E-01 | 4.72E-01 |
| c1605 | ursodeoxycholate | 12.16 | -7.54, 36.06 | 2.46E-01 | 5.33E-01 | 14.53 | -6.44, 40.19 | 1.90E-01 | 4.72E-01 |
| c39379 | glycoursodeoxycholate | 1.34 | -14.12, 19.59 | 8.75E-01 | 9.51E-01 | 0.02 | -15.78, 18.79 | 9.98E-01 | 9.98E-01 |
| c34093 | hyocholate | -11.28 | -21.20, -0.12 | 4.93E-02 | 2.01E-01 | -12.58 | -22.29, -1.66 | 2.64E-02 | 1.64E-01 |
| c42574 | glycohyocholate | -18.94 | -29.22, -7.17 | 2.77E-03 | 3.10E-02 | -20.87 | -31.41, -8.70 | 1.59E-03 | 2.27E-02 |
| c32599 | glycocholenate sulfate* | 1.94 | -4.09, 8.35 | 5.37E-01 | 7.78E-01 | 1.67 | -4.57, 8.33 | 6.08E-01 | 8.41E-01 |
| c32807 | taurocholenate sulfate* | 2.49 | -6.57, 12.43 | 6.03E-01 | 8.04E-01 | 2.36 | -7.07, 12.74 | 6.37E-01 | 8.51E-01 |
| c52975 | glycodeoxycholate 3-sulfate | -0.54 | -15.44, 16.98 | 9.48E-01 | 9.83E-01 | -1.07 | -16.59, 17.33 | 9.02E-01 | 9.64E-01 |
|  | **NUCLEOTIDE** |  |  |  |  |  |  |  |  |
|  | **Purine Metabolism, (Hypo)Xanthine/Inosine containing** | |  |  |  |  |  |  |  |
| c1123 | inosine | 12.98 | -11.02, 43.47 | 3.18E-01 | 6.13E-01 | 11.53 | -12.96, 42.92 | 3.89E-01 | 6.95E-01 |
| c3127 | hypoxanthine | 3.33 | -2.58, 9.61 | 2.77E-01 | 5.77E-01 | 2.07 | -3.92, 8.43 | 5.07E-01 | 7.80E-01 |
| c3147 | xanthine | 1.60 | -2.26, 5.61 | 4.23E-01 | 7.01E-01 | 1.29 | -2.76, 5.52 | 5.38E-01 | 7.98E-01 |
| c48351 | N1-methylinosine | 6.43 | 2.48, 10.53 | 1.48E-03 | 2.16E-02 | 6.92 | 2.95, 11.05 | 6.67E-04 | 1.49E-02 |
| c1604 | urate | 8.93 | 5.30, 12.69 | 1.76E-06 | 1.12E-04 | 9.29 | 5.49, 13.21 | 1.91E-06 | 1.54E-04 |
| c1107 | allantoin | 7.89 | 1.39, 14.80 | 1.77E-02 | 1.12E-01 | 7.22 | 0.49, 14.42 | 3.66E-02 | 1.96E-01 |
|  | **Purine Metabolism, Adenine containing** |  |  |  |  |  |  |  |  |
| c32342 | AMP | -1.40 | -4.24, 1.53 | 3.46E-01 | 6.43E-01 | -0.72 | -3.72, 2.36 | 6.43E-01 | 8.52E-01 |
| c554 | adenine | 2.35 | -1.91, 6.79 | 2.86E-01 | 5.93E-01 | 2.23 | -2.09, 6.73 | 3.18E-01 | 6.44E-01 |
| c15650 | 1-methyladenosine | 5.57 | -0.12, 11.59 | 5.68E-02 | 2.24E-01 | 6.18 | 0.17, 12.55 | 4.53E-02 | 2.27E-01 |
| c37114 | N6-methyladenosine | -0.16 | -7.65, 7.93 | 9.67E-01 | 9.90E-01 | -0.15 | -8.01, 8.39 | 9.72E-01 | 9.90E-01 |
| c35157 | N6-carbamoylthreonyladenosine | 3.83 | 1.06, 6.68 | 7.13E-03 | 6.40E-02 | 4.41 | 1.62, 7.27 | 2.12E-03 | 2.76E-02 |
|  | **Purine Metabolism, Guanine containing** |  |  |  |  |  |  |  |  |
| c35114 | 7-methylguanine | 4.62 | 1.45, 7.89 | 4.55E-03 | 4.48E-02 | 4.77 | 1.45, 8.20 | 5.16E-03 | 5.08E-02 |
| c35137 | N2,N2-dimethylguanosine | 3.00 | -0.24, 6.35 | 7.20E-02 | 2.57E-01 | 3.45 | 0.13, 6.88 | 4.28E-02 | 2.20E-01 |
|  | **Pyrimidine Metabolism, Orotate containing** |  |  |  |  |  |  |  |  |
| c1505 | orotate | 1.12 | -3.61, 6.08 | 6.50E-01 | 8.29E-01 | 0.79 | -4.08, 5.91 | 7.56E-01 | 9.14E-01 |
| c35172 | orotidine | 5.88 | 1.85, 10.06 | 4.38E-03 | 4.39E-02 | 6.40 | 2.29, 10.67 | 2.33E-03 | 2.89E-02 |
|  | **Pyrimidine Metabolism, Uracil containing** |  |  |  |  |  |  |  |  |
| c606 | uridine | 2.23 | -0.64, 5.18 | 1.30E-01 | 3.74E-01 | 2.50 | -0.54, 5.64 | 1.09E-01 | 3.49E-01 |
| c33442 | pseudouridine | 1.27 | -1.29, 3.89 | 3.35E-01 | 6.31E-01 | 1.37 | -1.21, 4.02 | 3.03E-01 | 6.23E-01 |
| c35136 | 5-methyluridine (ribothymidine) | -1.69 | -4.40, 1.09 | 2.33E-01 | 5.19E-01 | -1.64 | -4.37, 1.16 | 2.49E-01 | 5.50E-01 |
| c3155 | 3-ureidopropionate | -1.31 | -6.42, 4.08 | 6.28E-01 | 8.19E-01 | -1.28 | -6.50, 4.23 | 6.43E-01 | 8.52E-01 |
| c55 | beta-alanine | 4.86 | -0.03, 10.00 | 5.31E-02 | 2.14E-01 | 5.56 | 0.51, 10.86 | 3.18E-02 | 1.79E-01 |
| c37432 | N-acetyl-beta-alanine | 1.16 | -2.86, 5.33 | 5.78E-01 | 7.92E-01 | 1.75 | -2.34, 6.02 | 4.08E-01 | 7.15E-01 |
|  | **Pyrimidine Metabolism, Cytidine containing** |  |  |  |  |  |  |  |  |
| c514 | cytidine | 2.21 | -4.39, 9.27 | 5.21E-01 | 7.77E-01 | 2.44 | -4.38, 9.75 | 4.93E-01 | 7.70E-01 |
|  | **Pyrimidine Metabolism, Thymine containing** |  |  |  |  |  |  |  |  |
| c1418 | 5,6-dihydrothymine | 2.77 | -0.66, 6.32 | 1.17E-01 | 3.57E-01 | 2.66 | -0.91, 6.35 | 1.48E-01 | 4.09E-01 |
| c1566 | 3-aminoisobutyrate | 1.56 | -5.88, 9.59 | 6.91E-01 | 8.56E-01 | 0.84 | -6.93, 9.25 | 8.38E-01 | 9.34E-01 |
|  | **COFACTORS** |  |  |  |  |  |  |  |  |
|  | **Nicotinate and Nicotinamide Metabolism** |  |  |  |  |  |  |  |  |
| c1899 | quinolinate | 5.81 | 0.59, 11.30 | 2.99E-02 | 1.54E-01 | 6.76 | 1.30, 12.51 | 1.57E-02 | 1.12E-01 |
| c594 | nicotinamide | -1.53 | -7.54, 4.88 | 6.33E-01 | 8.21E-01 | -1.60 | -7.96, 5.20 | 6.37E-01 | 8.51E-01 |
| c27665 | 1-methylnicotinamide | 6.37 | -3.99, 17.84 | 2.39E-01 | 5.27E-01 | 5.57 | -5.21, 17.58 | 3.26E-01 | 6.53E-01 |
| c32401 | trigonelline (N'-methylnicotinate) | 0.67 | -9.99, 12.59 | 9.07E-01 | 9.69E-01 | 0.24 | -10.86, 12.71 | 9.69E-01 | 9.90E-01 |
| c40469 | N1-Methyl-2-pyridone-5-carboxamide | 3.90 | -2.72, 10.97 | 2.56E-01 | 5.48E-01 | 4.88 | -2.07, 12.32 | 1.75E-01 | 4.52E-01 |
|  | **Pantothenate and CoA Metabolism** |  |  |  |  |  |  |  |  |
| c1508 | pantothenate (Vitamin B5) | 2.51 | -1.25, 6.41 | 1.95E-01 | 4.84E-01 | 2.83 | -0.94, 6.75 | 1.45E-01 | 4.05E-01 |
|  | **Ascorbate and Aldarate Metabolism** |  |  |  |  |  |  |  |  |
| c27738 | threonate | -2.33 | -9.95, 5.94 | 5.70E-01 | 7.87E-01 | -1.84 | -9.91, 6.95 | 6.71E-01 | 8.79E-01 |
| c20694 | oxalate (ethanedioate) | -2.62 | -9.45, 4.72 | 4.75E-01 | 7.45E-01 | -1.31 | -8.34, 6.26 | 7.26E-01 | 9.04E-01 |
| c46957 | gulonate* | 1.03 | -3.17, 5.40 | 6.38E-01 | 8.22E-01 | 2.15 | -2.10, 6.59 | 3.28E-01 | 6.53E-01 |
|  | **Tocopherol Metabolism** |  |  |  |  |  |  |  |  |
| c1561 | alpha-tocopherol | -6.50 | -14.74, 2.55 | 1.56E-01 | 4.21E-01 | -5.64 | -14.06, 3.61 | 2.25E-01 | 5.15E-01 |
| c44876 | gamma-CEHC | -9.46 | -16.87, -1.39 | 2.37E-02 | 1.33E-01 | -8.13 | -16.00, 0.48 | 6.53E-02 | 2.74E-01 |
|  | **Hemoglobin and Porphyrin Metabolism** |  |  |  |  |  |  |  |  |
| c43807 | bilirubin | -5.04 | -18.34, 10.43 | 5.03E-01 | 7.65E-01 | -6.17 | -19.71, 9.66 | 4.25E-01 | 7.24E-01 |
| c32586 | bilirubin (E,E)* | -7.73 | -19.92, 6.31 | 2.67E-01 | 5.63E-01 | -9.87 | -22.16, 4.36 | 1.67E-01 | 4.37E-01 |
| c47886 | bilirubin (E,Z or Z,E)* | -4.69 | -17.73, 10.40 | 5.22E-01 | 7.77E-01 | -5.06 | -18.62, 10.75 | 5.10E-01 | 7.80E-01 |
| c2137 | biliverdin | 3.93 | -7.94, 17.34 | 5.34E-01 | 7.77E-01 | 1.19 | -10.65, 14.60 | 8.52E-01 | 9.43E-01 |
|  | **Vitamin A Metabolism** |  |  |  |  |  |  |  |  |
| c1806 | retinol (Vitamin A) | 2.22 | -1.82, 6.42 | 2.87E-01 | 5.94E-01 | 2.16 | -2.07, 6.57 | 3.23E-01 | 6.51E-01 |
|  | **Vitamin B6 Metabolism** |  |  |  |  |  |  |  |  |
| c31555 | pyridoxate | 1.18 | -5.24, 8.04 | 7.26E-01 | 8.83E-01 | 0.72 | -5.99, 7.90 | 8.39E-01 | 9.34E-01 |
|  | **XENOBIOTICS** |  |  |  |  |  |  |  |  |
|  | **Benzoate Metabolism** |  |  |  |  |  |  |  |  |
| c15753 | hippurate | -10.25 | -20.07, 0.78 | 6.92E-02 | 2.52E-01 | -11.27 | -21.22, -0.08 | 5.01E-02 | 2.36E-01 |
| c39600 | 3-hydroxyhippurate | 7.44 | -6.20, 23.05 | 3.02E-01 | 6.03E-01 | 7.76 | -6.53, 24.24 | 3.05E-01 | 6.24E-01 |
| c35527 | 4-hydroxyhippurate | -5.28 | -13.88, 4.17 | 2.65E-01 | 5.60E-01 | -3.81 | -12.91, 6.24 | 4.45E-01 | 7.38E-01 |
| c15778 | benzoate | 3.27 | -2.11, 8.94 | 2.40E-01 | 5.27E-01 | 2.82 | -2.72, 8.68 | 3.26E-01 | 6.53E-01 |
| c35320 | catechol sulfate | -6.76 | -14.78, 2.02 | 1.29E-01 | 3.74E-01 | -7.09 | -15.33, 1.96 | 1.23E-01 | 3.61E-01 |
| c46111 | guaiacol sulfate | -4.38 | -13.54, 5.75 | 3.85E-01 | 6.72E-01 | -3.70 | -13.17, 6.81 | 4.77E-01 | 7.61E-01 |
| c46165 | 3-methyl catechol sulfate (1) | 3.88 | -14.55, 26.28 | 7.03E-01 | 8.61E-01 | 2.17 | -16.41, 24.88 | 8.34E-01 | 9.34E-01 |
| c46146 | 4-methylcatechol sulfate | 0.95 | -8.52, 11.41 | 8.50E-01 | 9.48E-01 | 1.50 | -8.46, 12.55 | 7.78E-01 | 9.16E-01 |
| c36099 | 4-ethylphenyl sulfate | -2.59 | -15.52, 12.31 | 7.18E-01 | 8.76E-01 | -1.35 | -15.23, 14.80 | 8.61E-01 | 9.46E-01 |
| c36098 | 4-vinylphenol sulfate | -11.77 | -27.79, 7.81 | 2.23E-01 | 5.13E-01 | -8.46 | -25.27, 12.14 | 3.95E-01 | 6.98E-01 |
| c48763 | 3-methoxycatechol sulfate (1) | -3.72 | -19.93, 15.78 | 6.87E-01 | 8.56E-01 | -2.44 | -19.49, 18.21 | 8.01E-01 | 9.20E-01 |
| c48429 | methyl-4-hydroxybenzoate sulfate | 3.05 | -21.49, 35.24 | 8.29E-01 | 9.41E-01 | -0.68 | -24.09, 29.96 | 9.61E-01 | 9.86E-01 |
| c36103 | p-cresol sulfate | -5.06 | -12.94, 3.53 | 2.42E-01 | 5.27E-01 | -6.21 | -14.29, 2.63 | 1.65E-01 | 4.37E-01 |
| c35635 | 3-(3-hydroxyphenyl)propionate | 1.78 | -10.57, 15.84 | 7.90E-01 | 9.23E-01 | 3.74 | -9.61, 19.06 | 6.02E-01 | 8.37E-01 |
| c15749 | 3-phenylpropionate (hydrocinnamate) | -12.12 | -23.33, 0.73 | 6.52E-02 | 2.40E-01 | -13.36 | -24.83, -0.14 | 4.93E-02 | 2.36E-01 |
|  | **Xanthine Metabolism** |  |  |  |  |  |  |  |  |
| c569 | caffeine | 15.87 | -11.77, 52.18 | 2.91E-01 | 5.95E-01 | 15.03 | -13.41, 52.82 | 3.35E-01 | 6.55E-01 |
| c18392 | theobromine | -4.29 | -21.26, 16.34 | 6.60E-01 | 8.40E-01 | -2.31 | -20.33, 19.78 | 8.22E-01 | 9.32E-01 |
| c32445 | 3-methylxanthine | -11.23 | -26.83, 7.71 | 2.29E-01 | 5.17E-01 | -11.99 | -27.95, 7.51 | 2.13E-01 | 4.98E-01 |
| c34390 | 7-methylxanthine | -14.04 | -31.98, 8.63 | 2.07E-01 | 4.96E-01 | -13.10 | -31.37, 10.05 | 2.46E-01 | 5.45E-01 |
| c34424 | 5-acetylamino-6-amino-3-methyluracil | -8.82 | -30.31, 19.30 | 5.02E-01 | 7.65E-01 | -11.06 | -32.72, 17.59 | 4.12E-01 | 7.17E-01 |
|  | **Food Component/Plant** |  |  |  |  |  |  |  |  |
| c43400 | 2-piperidinone | 3.52 | -10.56, 19.80 | 6.44E-01 | 8.24E-01 | 4.13 | -10.50, 21.16 | 6.01E-01 | 8.37E-01 |
| c38276 | 2,3-dihydroxyisovalerate | -1.58 | -14.42, 13.20 | 8.24E-01 | 9.40E-01 | -1.96 | -15.40, 13.62 | 7.93E-01 | 9.19E-01 |
| c587 | gluconate | 1.22 | -2.75, 5.35 | 5.54E-01 | 7.87E-01 | 1.21 | -2.86, 5.46 | 5.66E-01 | 8.14E-01 |
| c38637 | cinnamoylglycine | -21.62 | -34.54, -6.15 | 8.77E-03 | 7.26E-02 | -22.07 | -35.29, -6.14 | 9.36E-03 | 7.53E-02 |
| c37459 | ergothioneine | 0.03 | -7.47, 8.13 | 9.94E-01 | 9.98E-01 | 1.51 | -6.32, 10.00 | 7.15E-01 | 9.03E-01 |
| c20699 | erythritol | 1.48 | -1.80, 4.86 | 3.83E-01 | 6.70E-01 | 2.20 | -1.11, 5.62 | 1.97E-01 | 4.73E-01 |
| c33009 | homostachydrine* | -2.06 | -9.50, 6.00 | 6.07E-01 | 8.04E-01 | -2.62 | -10.27, 5.69 | 5.26E-01 | 7.88E-01 |
| c43374 | indolin-2-one | -3.14 | -10.37, 4.67 | 4.21E-01 | 7.01E-01 | -2.42 | -10.03, 5.84 | 5.55E-01 | 8.09E-01 |
| c33935 | piperine | 66.34 | 25.78,119.96 | 4.62E-04 | 1.01E-02 | 66.18 | 24.81,121.28 | 6.40E-04 | 1.49E-02 |
| c43239 | S-allylcysteine | -14.70 | -34.05, 10.32 | 2.27E-01 | 5.17E-01 | -8.47 | -29.93, 19.57 | 5.17E-01 | 7.87E-01 |
| c15336 | tartarate | -5.98 | -16.54, 5.91 | 3.12E-01 | 6.07E-01 | -8.38 | -19.32, 4.05 | 1.79E-01 | 4.57E-01 |
| c37181 | 4-allylphenol sulfate | 4.81 | -10.84, 23.20 | 5.70E-01 | 7.87E-01 | 4.93 | -11.46, 24.36 | 5.79E-01 | 8.27E-01 |
| c20693 | tartronate (hydroxymalonate) | 3.22 | -5.32, 12.54 | 4.73E-01 | 7.45E-01 | 2.90 | -6.01, 12.65 | 5.37E-01 | 7.98E-01 |
|  | **Drug - Topical Agents** |  |  |  |  |  |  |  |  |
| c1515 | salicylate | 2.46 | -9.09, 15.47 | 6.91E-01 | 8.56E-01 | 2.47 | -9.74, 16.33 | 7.07E-01 | 9.01E-01 |
|  | **Chemical** |  |  |  |  |  |  |  |  |
| c46960 | sulfate* | 0.23 | -2.42, 2.94 | 8.68E-01 | 9.48E-01 | 0.42 | -2.32, 3.24 | 7.65E-01 | 9.16E-01 |
| c45413 | O-sulfo-L-tyrosine | 0.96 | -2.23, 4.25 | 5.61E-01 | 7.87E-01 | 1.58 | -1.72, 5.01 | 3.53E-01 | 6.68E-01 |
| c43266 | 2-aminophenol sulfate | -3.98 | -17.36, 11.56 | 5.96E-01 | 8.01E-01 | -2.11 | -16.40, 14.62 | 7.91E-01 | 9.19E-01 |
| c48441 | 4-hydroxychlorothalonil | 2.31 | -3.43, 8.39 | 4.40E-01 | 7.17E-01 | 1.80 | -4.15, 8.11 | 5.63E-01 | 8.14E-01 |
| c48448 | 3-hydroxypyridine sulfate | -7.13 | -18.23, 5.47 | 2.56E-01 | 5.48E-01 | -8.43 | -19.83, 4.59 | 1.96E-01 | 4.73E-01 |
| c48698 | 6-hydroxyindole sulfate | -3.70 | -10.35, 3.44 | 3.03E-01 | 6.03E-01 | -2.95 | -10.00, 4.67 | 4.39E-01 | 7.35E-01 |
| c53231 | thioproline | -3.37 | -11.62, 5.65 | 4.53E-01 | 7.20E-01 | -3.98 | -12.62, 5.51 | 4.00E-01 | 7.04E-01 |
|  | **UNKNOWN** |  |  |  |  |  |  |  |  |
| c32578 | X - 11261 | 4.47 | -4.48, 14.26 | 3.40E-01 | 6.37E-01 | 3.07 | -6.00, 13.02 | 5.21E-01 | 7.88E-01 |
| c33132 | X - 11787 | 0.70 | -1.89, 3.37 | 6.00E-01 | 8.04E-01 | 1.23 | -1.51, 4.05 | 3.82E-01 | 6.86E-01 |
| c33140 | X - 11795 | 7.86 | 0.97, 15.22 | 2.60E-02 | 1.41E-01 | 8.97 | 1.65, 16.82 | 1.66E-02 | 1.14E-01 |
| c46259 | X - 21258 | -7.96 | -21.83, 8.37 | 3.21E-01 | 6.17E-01 | -7.41 | -22.00, 9.91 | 3.80E-01 | 6.86E-01 |
| c46266 | X - 15486 | 1.79 | -10.83, 16.18 | 7.93E-01 | 9.23E-01 | 0.35 | -12.62, 15.25 | 9.60E-01 | 9.86E-01 |
| c46283 | X - 15461 | 1.66 | -2.17, 5.64 | 4.01E-01 | 6.85E-01 | 1.91 | -2.12, 6.11 | 3.60E-01 | 6.76E-01 |
| c46295 | X - 21286 | -2.24 | -9.78, 5.93 | 5.80E-01 | 7.92E-01 | -2.23 | -10.17, 6.41 | 6.03E-01 | 8.37E-01 |
| c46347 | X - 11381 | -2.77 | -6.84, 1.47 | 1.99E-01 | 4.88E-01 | -2.02 | -6.23, 2.37 | 3.62E-01 | 6.76E-01 |
| c46354 | X - 21310 | -2.00 | -6.94, 3.19 | 4.43E-01 | 7.17E-01 | -1.14 | -6.34, 4.35 | 6.78E-01 | 8.81E-01 |
| c46356 | X - 21312 | 3.59 | -13.09, 23.48 | 6.94E-01 | 8.58E-01 | 4.97 | -11.72, 24.81 | 5.84E-01 | 8.27E-01 |
| c46363 | X - 21319 | 1.79 | -8.34, 13.03 | 7.41E-01 | 8.93E-01 | 1.45 | -9.07, 13.19 | 7.97E-01 | 9.20E-01 |
| c46364 | X - 12847 | 8.41 | -13.91, 36.53 | 4.93E-01 | 7.63E-01 | 6.52 | -16.37, 35.67 | 6.10E-01 | 8.41E-01 |
| c46384 | X - 21339 | 7.34 | -0.29, 15.56 | 6.14E-02 | 2.32E-01 | 6.05 | -1.78, 14.50 | 1.35E-01 | 3.84E-01 |
| c46390 | X - 11308 | -0.59 | -7.13, 6.41 | 8.65E-01 | 9.48E-01 | -0.95 | -7.60, 6.19 | 7.89E-01 | 9.19E-01 |
| c46398 | X - 21353 | 1.49 | -6.84, 10.57 | 7.35E-01 | 8.89E-01 | 1.43 | -7.19, 10.84 | 7.54E-01 | 9.14E-01 |
| c46409 | X - 21364 | 16.70 | 10.27, 23.50 | 2.75E-07 | 2.62E-05 | 15.51 | 8.91, 22.51 | 3.32E-06 | 1.90E-04 |
| c46417 | X - 13866 | 1.47 | -7.97, 11.88 | 7.70E-01 | 9.12E-01 | 0.74 | -9.10, 11.66 | 8.88E-01 | 9.59E-01 |
| c46428 | X - 21383 | 13.85 | -0.48, 30.25 | 6.04E-02 | 2.30E-01 | 13.84 | -0.51, 30.27 | 6.11E-02 | 2.62E-01 |
| c46460 | X - 11444 | 11.80 | 6.14, 17.75 | 4.03E-05 | 1.44E-03 | 11.23 | 5.66, 17.09 | 7.39E-05 | 2.64E-03 |
| c46466 | X - 11843 | -28.46 | -48.30, -1.02 | 4.47E-02 | 1.95E-01 | -31.49 | -51.30, -3.63 | 3.12E-02 | 1.78E-01 |
| c46486 | X - 21441 | 21.42 | 8.38, 36.02 | 9.89E-04 | 1.71E-02 | 20.13 | 6.63, 35.34 | 2.95E-03 | 3.30E-02 |
| c46507 | X - 11850 | -29.67 | -47.64, -5.55 | 2.04E-02 | 1.19E-01 | -33.75 | -51.23, -9.99 | 9.21E-03 | 7.51E-02 |
| c46510 | X - 12544 | 3.54 | -6.43, 14.59 | 5.01E-01 | 7.65E-01 | 4.87 | -5.84, 16.80 | 3.88E-01 | 6.94E-01 |
| c46512 | X - 21467 | 6.90 | -1.64, 16.18 | 1.18E-01 | 3.58E-01 | 7.98 | -0.88, 17.64 | 8.07E-02 | 2.90E-01 |
| c46515 | X - 21470 | 28.96 | 12.70, 47.56 | 2.88E-04 | 7.48E-03 | 28.16 | 11.20, 47.70 | 7.63E-04 | 1.56E-02 |
| c46516 | X - 21471 | -1.71 | -9.72, 7.01 | 6.91E-01 | 8.56E-01 | -1.65 | -10.04, 7.51 | 7.14E-01 | 9.03E-01 |
| c46517 | X - 16946 | 7.74 | -2.47, 19.01 | 1.44E-01 | 4.02E-01 | 6.28 | -4.24, 17.95 | 2.54E-01 | 5.57E-01 |
| c46521 | X - 11852 | 3.73 | -23.20, 40.09 | 8.12E-01 | 9.37E-01 | 4.18 | -23.68, 42.21 | 7.97E-01 | 9.20E-01 |
| c46590 | X - 07765 | -6.76 | -20.04, 8.72 | 3.73E-01 | 6.61E-01 | -3.82 | -18.13, 12.98 | 6.36E-01 | 8.51E-01 |
| c46592 | X - 11299 | 6.47 | -20.60, 42.78 | 6.76E-01 | 8.54E-01 | -0.11 | -25.87, 34.61 | 9.94E-01 | 9.98E-01 |
| c46594 | X - 11372 | 1.56 | -3.63, 7.03 | 5.63E-01 | 7.87E-01 | 1.00 | -4.39, 6.69 | 7.23E-01 | 9.04E-01 |
| c46601 | X - 11470 | 3.46 | -3.70, 11.16 | 3.54E-01 | 6.45E-01 | 1.54 | -5.58, 9.19 | 6.81E-01 | 8.81E-01 |
| c46602 | X - 11478 | 3.10 | -6.76, 14.01 | 5.52E-01 | 7.87E-01 | 1.84 | -8.29, 13.09 | 7.34E-01 | 9.07E-01 |
| c46607 | X - 11849 | 28.12 | -3.76, 70.55 | 9.14E-02 | 3.01E-01 | 28.36 | -4.96, 73.36 | 1.05E-01 | 3.38E-01 |
| c46608 | X - 11880 | 4.59 | -1.00, 10.50 | 1.11E-01 | 3.42E-01 | 4.12 | -1.70, 10.29 | 1.70E-01 | 4.44E-01 |
| c46613 | X - 12216 | -10.45 | -23.91, 5.40 | 1.86E-01 | 4.70E-01 | -13.22 | -26.52, 2.49 | 9.66E-02 | 3.21E-01 |
| c46616 | X - 12411 | 1.98 | -9.21, 14.56 | 7.41E-01 | 8.93E-01 | -0.53 | -12.11, 12.58 | 9.33E-01 | 9.73E-01 |
| c46623 | X - 12729 | 3.51 | -9.36, 18.20 | 6.12E-01 | 8.07E-01 | 1.08 | -11.52, 15.48 | 8.74E-01 | 9.52E-01 |
| c46624 | X - 12798 | -3.89 | -9.65, 2.24 | 2.10E-01 | 4.97E-01 | -2.57 | -8.49, 3.72 | 4.16E-01 | 7.22E-01 |
| c46632 | X - 14056 | -1.43 | -12.66, 11.24 | 8.16E-01 | 9.39E-01 | 0.64 | -11.15, 13.99 | 9.21E-01 | 9.70E-01 |
| c46633 | X - 12844 | 7.63 | 2.61, 12.88 | 2.89E-03 | 3.18E-02 | 7.40 | 2.19, 12.88 | 5.44E-03 | 5.27E-02 |
| c46636 | X - 12849 | -26.94 | -42.66, -6.91 | 1.20E-02 | 8.54E-02 | -28.04 | -43.79, -7.88 | 9.81E-03 | 7.78E-02 |
| c46640 | X - 15469 | -4.59 | -11.18, 2.49 | 2.00E-01 | 4.88E-01 | -4.34 | -10.87, 2.67 | 2.21E-01 | 5.10E-01 |
| c46645 | X - 13728 | -8.20 | -25.29, 12.80 | 4.17E-01 | 7.01E-01 | -7.10 | -24.89, 14.89 | 4.98E-01 | 7.72E-01 |
| c46646 | X - 13835 | 9.82 | -8.18, 31.34 | 3.06E-01 | 6.05E-01 | 7.39 | -11.14, 29.80 | 4.62E-01 | 7.53E-01 |
| c46657 | X - 14939 | 2.27 | -5.83, 11.07 | 5.95E-01 | 8.01E-01 | 1.43 | -6.83, 10.42 | 7.44E-01 | 9.13E-01 |
| c46661 | X - 15245 | -12.69 | -25.74, 2.66 | 1.02E-01 | 3.23E-01 | -8.94 | -22.95, 7.62 | 2.74E-01 | 5.85E-01 |
| c46662 | X - 15492 | 29.05 | 17.68, 41.52 | 1.93E-07 | 2.20E-05 | 29.01 | 17.12, 42.10 | 6.56E-07 | 7.95E-05 |
| c46666 | X - 15728 | 17.86 | -5.03, 46.26 | 1.38E-01 | 3.91E-01 | 17.40 | -6.39, 47.23 | 1.67E-01 | 4.37E-01 |
| c46673 | X - 16576 | -13.89 | -21.33, -5.76 | 1.39E-03 | 2.09E-02 | -13.65 | -21.53, -4.99 | 3.01E-03 | 3.31E-02 |
| c46674 | X - 21607 | 16.14 | 1.57, 32.81 | 3.00E-02 | 1.54E-01 | 15.07 | 0.07, 32.31 | 5.04E-02 | 2.36E-01 |
| c46681 | X - 16935 | 6.25 | -4.62, 18.35 | 2.72E-01 | 5.71E-01 | 6.29 | -4.76, 18.62 | 2.78E-01 | 5.89E-01 |
| c46683 | X - 16944 | 3.33 | -6.08, 13.68 | 5.02E-01 | 7.65E-01 | 2.44 | -7.35, 13.27 | 6.39E-01 | 8.51E-01 |
| c46685 | X - 16964 | -5.82 | -11.92, 0.70 | 8.10E-02 | 2.77E-01 | -5.94 | -12.27, 0.83 | 8.62E-02 | 3.00E-01 |
| c46690 | X - 18901 | -6.44 | -14.46, 2.33 | 1.47E-01 | 4.09E-01 | -8.04 | -16.21, 0.92 | 7.91E-02 | 2.90E-01 |
| c46695 | X - 18913 | 2.11 | -3.21, 7.73 | 4.45E-01 | 7.17E-01 | 1.67 | -3.84, 7.50 | 5.61E-01 | 8.13E-01 |
| c46700 | X - 18922 | -2.59 | -12.60, 8.56 | 6.35E-01 | 8.21E-01 | -5.27 | -15.07, 5.66 | 3.32E-01 | 6.55E-01 |
| c46701 | X - 19141 | 2.44 | -6.39, 12.11 | 6.01E-01 | 8.04E-01 | 1.49 | -7.37, 11.19 | 7.52E-01 | 9.14E-01 |
| c46710 | X - 17690 | 12.28 | -11.97, 43.21 | 3.52E-01 | 6.44E-01 | 12.08 | -12.75, 43.96 | 3.73E-01 | 6.83E-01 |
| c46902 | X - 21733 | -3.25 | -14.91, 10.02 | 6.15E-01 | 8.07E-01 | -1.50 | -13.94, 12.75 | 8.27E-01 | 9.34E-01 |
| c46905 | X - 21736 | 3.97 | -5.47, 14.36 | 4.24E-01 | 7.01E-01 | 2.46 | -7.19, 13.12 | 6.31E-01 | 8.51E-01 |
| c46909 | X - 21740 | -7.07 | -13.77, 0.15 | 5.65E-02 | 2.24E-01 | -7.48 | -14.36, -0.05 | 5.01E-02 | 2.36E-01 |
| c46932 | X - 12104 | 9.54 | 3.62, 15.81 | 1.57E-03 | 2.19E-02 | 9.66 | 3.48, 16.22 | 2.15E-03 | 2.76E-02 |
| c46972 | X - 21796 | 5.32 | -0.39, 11.35 | 7.03E-02 | 2.52E-01 | 5.07 | -0.78, 11.27 | 9.27E-02 | 3.11E-01 |
| c46977 | X - 15503 | 7.08 | 1.67, 12.77 | 1.04E-02 | 7.76E-02 | 6.82 | 1.25, 12.69 | 1.67E-02 | 1.14E-01 |
| c46997 | X - 12822 | 6.25 | -2.26, 15.52 | 1.56E-01 | 4.22E-01 | 7.47 | -1.57, 17.35 | 1.10E-01 | 3.49E-01 |
| c47006 | X - 21829 | 9.82 | -4.47, 26.25 | 1.89E-01 | 4.74E-01 | 6.33 | -7.59, 22.34 | 3.92E-01 | 6.96E-01 |
| c47013 | X - 16570 | 7.99 | -3.27, 20.56 | 1.73E-01 | 4.53E-01 | 6.70 | -4.63, 19.36 | 2.59E-01 | 5.65E-01 |
| c47301 | X - 18887 | 3.88 | -3.34, 11.63 | 3.02E-01 | 6.03E-01 | 6.62 | -0.51, 14.26 | 7.12E-02 | 2.80E-01 |
| c47417 | X - 22162 | -3.00 | -7.17, 1.36 | 1.76E-01 | 4.56E-01 | -1.40 | -5.75, 3.15 | 5.40E-01 | 7.98E-01 |
| c47439 | X - 13507 | -3.84 | -9.55, 2.22 | 2.11E-01 | 4.97E-01 | -4.23 | -10.26, 2.22 | 1.95E-01 | 4.73E-01 |
| c47642 | X - 12101 | -5.56 | -15.38, 5.40 | 3.09E-01 | 6.06E-01 | -5.96 | -16.17, 5.49 | 2.96E-01 | 6.17E-01 |
| c47664 | X - 13658 | 1.53 | -11.17, 16.04 | 8.24E-01 | 9.40E-01 | 1.65 | -11.44, 16.68 | 8.17E-01 | 9.32E-01 |
| c47670 | X - 18899 | -0.13 | -8.76, 9.32 | 9.77E-01 | 9.93E-01 | -1.37 | -10.28, 8.42 | 7.76E-01 | 9.16E-01 |
| c47671 | X - 18921 | -2.71 | -11.75, 7.25 | 5.81E-01 | 7.92E-01 | -4.46 | -13.71, 5.78 | 3.81E-01 | 6.86E-01 |
| c47673 | X - 19299 | -3.07 | -21.94, 20.36 | 7.78E-01 | 9.18E-01 | -2.17 | -22.00, 22.69 | 8.49E-01 | 9.43E-01 |
| c47687 | X - 12100 | 5.06 | 0.33, 10.01 | 3.73E-02 | 1.76E-01 | 5.60 | 0.61, 10.85 | 2.89E-02 | 1.71E-01 |
| c47708 | X - 12283 | 4.82 | -7.21, 18.41 | 4.50E-01 | 7.17E-01 | 4.45 | -7.98, 18.57 | 5.01E-01 | 7.76E-01 |
| c47783 | X - 22519 | -3.13 | -9.76, 3.99 | 3.80E-01 | 6.70E-01 | -2.57 | -9.20, 4.55 | 4.71E-01 | 7.61E-01 |
| c47802 | X - 16397 | 5.75 | -7.10, 20.39 | 3.99E-01 | 6.84E-01 | 5.23 | -7.84, 20.16 | 4.52E-01 | 7.45E-01 |
| c47804 | X - 16580 | 0.10 | -7.89, 8.78 | 9.81E-01 | 9.94E-01 | -0.44 | -8.55, 8.39 | 9.19E-01 | 9.70E-01 |
| c47872 | X - 17340 | 27.23 | 17.17, 38.16 | 4.25E-08 | 1.21E-05 | 28.52 | 17.89, 40.11 | 5.17E-08 | 1.48E-05 |
| c47905 | X - 12026 | 0.41 | -4.13, 5.15 | 8.64E-01 | 9.48E-01 | -0.43 | -4.95, 4.30 | 8.55E-01 | 9.43E-01 |
| c47929 | X - 12707 | -7.11 | -15.38, 1.97 | 1.23E-01 | 3.69E-01 | -6.16 | -14.93, 3.51 | 2.05E-01 | 4.84E-01 |
| c47959 | X - 13553 | -4.59 | -11.50, 2.87 | 2.23E-01 | 5.13E-01 | -5.31 | -12.50, 2.47 | 1.77E-01 | 4.57E-01 |
| c48001 | X - 17351 | 6.85 | -5.72, 21.10 | 3.01E-01 | 6.03E-01 | 8.11 | -5.07, 23.12 | 2.41E-01 | 5.42E-01 |
| c48047 | X - 18886 | 10.77 | 2.62, 19.58 | 9.51E-03 | 7.44E-02 | 10.18 | 1.69, 19.38 | 1.90E-02 | 1.23E-01 |
| c48076 | X - 22771 | -6.88 | -13.11, -0.20 | 4.53E-02 | 1.96E-01 | -7.61 | -14.04, -0.69 | 3.31E-02 | 1.82E-01 |
| c49463 | X - 23587 | 8.17 | -2.11, 19.53 | 1.25E-01 | 3.70E-01 | 8.83 | -1.88, 20.70 | 1.11E-01 | 3.49E-01 |
| c49466 | X - 23590 | 15.47 | 9.65, 21.61 | 1.69E-07 | 2.20E-05 | 16.37 | 10.23, 22.86 | 1.50E-07 | 2.85E-05 |
| c49469 | X - 23593 | 1.27 | -2.78, 5.48 | 5.45E-01 | 7.80E-01 | 0.93 | -3.38, 5.42 | 6.79E-01 | 8.81E-01 |
| c49515 | X - 23639 | -4.35 | -8.81, 0.32 | 6.92E-02 | 2.52E-01 | -4.52 | -9.17, 0.36 | 7.06E-02 | 2.80E-01 |
| c49517 | X - 23641 | 3.90 | -8.10, 17.46 | 5.42E-01 | 7.80E-01 | 2.91 | -9.45, 16.95 | 6.61E-01 | 8.69E-01 |
| c49521 | X - 23644 | -13.65 | -30.71, 7.60 | 1.93E-01 | 4.81E-01 | -12.98 | -30.45, 8.87 | 2.25E-01 | 5.15E-01 |
| c49536 | X - 23659 | -6.15 | -15.27, 3.94 | 2.25E-01 | 5.13E-01 | -4.25 | -13.64, 6.15 | 4.10E-01 | 7.16E-01 |
| c49557 | X - 23680 | 1.91 | -6.69, 11.30 | 6.75E-01 | 8.54E-01 | 0.49 | -8.26, 10.08 | 9.16E-01 | 9.70E-01 |
| c49592 | X - 11315 | -1.37 | -5.49, 2.93 | 5.27E-01 | 7.77E-01 | -1.34 | -5.67, 3.20 | 5.58E-01 | 8.10E-01 |
| c49637 | X - 23739 | 6.92 | 0.38, 13.88 | 3.93E-02 | 1.84E-01 | 6.20 | -0.55, 13.40 | 7.43E-02 | 2.87E-01 |
| c49679 | X - 23780 | -2.56 | -11.93, 7.81 | 6.16E-01 | 8.07E-01 | -2.62 | -12.41, 8.26 | 6.24E-01 | 8.48E-01 |
| c49681 | X - 23782 | -5.44 | -11.47, 1.01 | 9.83E-02 | 3.15E-01 | -4.85 | -11.20, 1.95 | 1.60E-01 | 4.28E-01 |
| c49883 | X - 23974 | 0.26 | -6.66, 7.71 | 9.42E-01 | 9.82E-01 | 0.96 | -6.25, 8.74 | 8.00E-01 | 9.20E-01 |
| c52483 | X - 24295 | -6.75 | -21.13, 10.26 | 4.15E-01 | 7.01E-01 | -4.72 | -20.27, 13.87 | 5.96E-01 | 8.36E-01 |
| c52524 | X - 24328 | 24.64 | 13.79, 36.52 | 4.34E-06 | 2.13E-04 | 25.75 | 14.38, 38.26 | 4.48E-06 | 2.32E-04 |
| c52533 | X - 24337 | 12.63 | 1.77, 24.65 | 2.27E-02 | 1.29E-01 | 10.60 | -0.48, 22.91 | 6.32E-02 | 2.67E-01 |
| c52636 | X - 24422 | -3.81 | -9.59, 2.35 | 2.21E-01 | 5.13E-01 | -2.04 | -8.04, 4.35 | 5.23E-01 | 7.88E-01 |
| c52665 | X - 24435 | -2.55 | -6.59, 1.66 | 2.33E-01 | 5.19E-01 | -2.31 | -6.55, 2.12 | 3.03E-01 | 6.23E-01 |
| c52772 | X - 24455 | 3.31 | -1.91, 8.81 | 2.19E-01 | 5.11E-01 | 3.64 | -1.86, 9.46 | 2.00E-01 | 4.79E-01 |
| c52773 | X - 24456 | 4.65 | -2.51, 12.34 | 2.10E-01 | 4.97E-01 | 3.33 | -3.91, 11.12 | 3.78E-01 | 6.86E-01 |
| c52865 | X - 24544 | 18.77 | 6.34, 32.65 | 2.64E-03 | 3.05E-02 | 18.68 | 5.63, 33.33 | 4.44E-03 | 4.61E-02 |
| c52867 | X - 24546 | 22.69 | 8.66, 38.52 | 1.16E-03 | 1.89E-02 | 21.25 | 7.24, 37.10 | 2.44E-03 | 2.97E-02 |
| c52877 | X - 24556 | -2.46 | -10.44, 6.24 | 5.69E-01 | 7.87E-01 | -4.22 | -12.25, 4.54 | 3.36E-01 | 6.55E-01 |
| c52909 | X - 24588 | 9.40 | 2.22, 17.08 | 1.02E-02 | 7.76E-02 | 8.18 | 0.73, 16.19 | 3.21E-02 | 1.79E-01 |
| c53127 | X - 24699 | 0.24 | -2.34, 2.89 | 8.58E-01 | 9.48E-01 | -0.14 | -2.79, 2.57 | 9.16E-01 | 9.70E-01 |
| c54840 | X - 24812 | 6.52 | -0.77, 14.34 | 8.23E-02 | 2.80E-01 | 6.76 | -0.88, 14.99 | 8.59E-02 | 3.00E-01 |
| c57714 | X - 24947 | 10.76 | -0.38, 23.15 | 6.04E-02 | 2.30E-01 | 10.48 | -1.13, 23.44 | 8.03E-02 | 2.90E-01 |
| c57716 | X - 24949 | 2.92 | -8.00, 15.14 | 6.16E-01 | 8.07E-01 | 0.74 | -9.67, 12.35 | 8.95E-01 | 9.60E-01 |
| c57720 | X - 24953 | 8.60 | 1.48, 16.21 | 1.81E-02 | 1.12E-01 | 9.28 | 1.84, 17.26 | 1.47E-02 | 1.07E-01 |
| c62636 | X - 25343 | -5.53 | -16.83, 7.30 | 3.83E-01 | 6.70E-01 | -5.38 | -17.19, 8.12 | 4.18E-01 | 7.23E-01 |
| c62664 | X - 25371 | 7.55 | 3.21, 12.09 | 6.78E-04 | 1.25E-02 | 7.58 | 3.34, 12.00 | 4.80E-04 | 1.24E-02 |
| c62716 | X - 25419 | -1.51 | -11.48, 9.59 | 7.81E-01 | 9.18E-01 | -3.75 | -14.06, 7.80 | 5.09E-01 | 7.80E-01 |
| c62717 | X - 25420 | -5.59 | -12.29, 1.62 | 1.27E-01 | 3.72E-01 | -6.02 | -12.97, 1.50 | 1.16E-01 | 3.53E-01 |
| c62719 | X - 25422 | -8.39 | -16.29, 0.25 | 5.83E-02 | 2.28E-01 | -7.03 | -15.30, 2.05 | 1.27E-01 | 3.72E-01 |
| c62963 | X - 25519 | -1.33 | -10.19, 8.39 | 7.80E-01 | 9.18E-01 | -0.02 | -9.27, 10.19 | 9.97E-01 | 9.98E-01 |
| c62964 | X - 25520 | 0.02 | -9.57, 10.63 | 9.97E-01 | 9.98E-01 | 2.03 | -8.52, 13.81 | 7.18E-01 | 9.03E-01 |
| c63560 | X - 25790 | -4.36 | -9.99, 1.62 | 1.51E-01 | 4.13E-01 | -5.62 | -11.37, 0.50 | 7.29E-02 | 2.85E-01 |
| c63908 | X - 25957 | 5.66 | 0.26, 11.36 | 4.12E-02 | 1.90E-01 | 4.98 | -0.56, 10.83 | 8.10E-02 | 2.90E-01 |

^1^ Estimates are from robust mixed effects multivariable linear regression models including ln(metabolite level) as the dependent variable; BMI z-score (continuous), age at BMI measurement (continuous), and treatment group assignment as fixed effects; and DISC clinic as a random effect.

^2^ Estimates are from robust mixed effects multivariable linear regression models including ln(metabolite level) as the dependent variable; BMI z-score (continuous), age at BMI measurement (continuous), treatment group assignment, race (white/non-white), and menstrual cycle phase at blood collection (premenarche/luteal/follicular/unknown); and DISC clinic as a random effect.
