# Supplementary material for: Childhood adiposity, serum metabolites and breast density in young women
Source: Breast Cancer Res. 2022 Dec 19;24:91. doi: 10.1186/s13058-022-01588-y (PMC9764542; doi:10.1186/s13058-022-01588-y)
Supplement: Supplementary file 2 — Additional file 2. Difference in %DBV associated with a 10% increase in metabolite. [file 13058_2022_1588_MOESM2_ESM.docx]

| **Supplemental Table 2. Difference in %DBV associated with 10% increase in serum metabolite** | | | | | | | | | |
| --- | --- | --- | --- | --- | --- | --- | --- | --- | --- |
|  |  | **Minimally Adjusted Model^1^** | | | | **Fully Adjusted Model^2^** | | | |
| **Compid** | **Biochemical** | **Δ** | **95% CI** | **P-value** | **Q-value** | **Δ** | **95% CI** | **P-value** | **Q-value** |
|  | **AMINO ACIDS** |  |  |  |  |  |  |  |  |
|  | **Glycine, Serine and Threonine Metabolism** |  |  |  |  |  |  |  |  |
| c58 | glycine | 0.33 | -0.55, 1.20 | 0.467 | 0.941 | 0.41 | -0.45, 1.27 | 0.350 | 0.971 |
| c27710 | N-acetylglycine | 0.18 | -0.35, 0.71 | 0.506 | 0.941 | 0.18 | -0.34, 0.70 | 0.496 | 0.980 |
| c1516 | sarcosine | 0.25 | -0.24, 0.75 | 0.322 | 0.919 | 0.07 | -0.43, 0.57 | 0.782 | 0.993 |
| c5086 | dimethylglycine | 0.08 | -0.53, 0.69 | 0.797 | 0.951 | 0.19 | -0.40, 0.78 | 0.534 | 0.980 |
| c3141 | betaine | -0.27 | -1.19, 0.65 | 0.572 | 0.941 | -0.25 | -1.14, 0.65 | 0.588 | 0.980 |
| c1648 | serine | 0.14 | -0.66, 0.94 | 0.734 | 0.949 | 0.03 | -0.75, 0.81 | 0.934 | 0.993 |
| c37076 | N-acetylserine | -0.46 | -1.45, 0.53 | 0.365 | 0.919 | -0.08 | -1.07, 0.90 | 0.866 | 0.993 |
| c1284 | threonine | 0.28 | -0.55, 1.11 | 0.509 | 0.941 | 0.08 | -0.74, 0.90 | 0.855 | 0.993 |
| c33939 | N-acetylthreonine | -0.12 | -1.26, 1.02 | 0.835 | 0.963 | 0.33 | -0.78, 1.43 | 0.563 | 0.980 |
|  | **Alanine and Aspartate Metabolism** |  |  |  |  |  |  |  |  |
| c1126 | alanine | 0.31 | -0.72, 1.35 | 0.553 | 0.941 | 0.37 | -0.65, 1.39 | 0.477 | 0.980 |
| c1585 | N-acetylalanine | 0.35 | -0.98, 1.68 | 0.603 | 0.941 | 0.76 | -0.53, 2.05 | 0.252 | 0.940 |
| c443 | aspartate | -0.05 | -0.55, 0.45 | 0.854 | 0.967 | -0.13 | -0.60, 0.34 | 0.583 | 0.980 |
| c22185 | N-acetylaspartate (NAA) | 0.19 | -0.54, 0.92 | 0.611 | 0.941 | 0.39 | -0.31, 1.09 | 0.280 | 0.940 |
| c512 | asparagine | 0.31 | -0.42, 1.04 | 0.407 | 0.919 | 0.1 | -0.61, 0.81 | 0.782 | 0.993 |
| c33942 | N-acetylasparagine | -0.03 | -0.61, 0.54 | 0.915 | 0.982 | -0.18 | -0.74, 0.39 | 0.540 | 0.980 |
|  | **Glutamate Metabolism** |  |  |  |  |  |  |  |  |
| c57 | glutamate | 0.14 | -0.26, 0.53 | 0.507 | 0.941 | 0.16 | -0.23, 0.54 | 0.429 | 0.980 |
| c53 | glutamine | 0.03 | -0.65, 0.71 | 0.925 | 0.983 | 0.02 | -0.64, 0.67 | 0.963 | 0.993 |
| c15720 | N-acetylglutamate | -0.16 | -0.88, 0.57 | 0.675 | 0.941 | -0.01 | -0.72, 0.69 | 0.970 | 0.993 |
| c46225 | pyroglutamine* | -0.02 | -0.40, 0.36 | 0.930 | 0.983 | -0.02 | -0.39, 0.35 | 0.933 | 0.993 |
| c35665 | N-acetyl-aspartyl-glutamate (NAAG) | -0.13 | -0.50, 0.24 | 0.492 | 0.941 | -0.18 | -0.54, 0.18 | 0.328 | 0.962 |
| c54923 | beta-citrylglutamate | 0.23 | -0.10, 0.57 | 0.169 | 0.844 | 0.26 | -0.06, 0.58 | 0.116 | 0.940 |
| c42370 | S-1-pyrroline-5-carboxylate | -0.23 | -0.71, 0.24 | 0.343 | 0.919 | -0.15 | -0.61, 0.31 | 0.520 | 0.980 |
|  | **Histidine Metabolism** |  |  |  |  |  |  |  |  |
| c59 | histidine | 0.15 | -0.77, 1.08 | 0.748 | 0.951 | 0.20 | -0.70, 1.10 | 0.665 | 0.980 |
| c30460 | 1-methylhistidine | -0.10 | -0.64, 0.43 | 0.710 | 0.949 | -0.11 | -0.63, 0.41 | 0.683 | 0.980 |
| c33946 | N-acetylhistidine | -0.08 | -0.54, 0.37 | 0.719 | 0.949 | -0.05 | -0.51, 0.40 | 0.817 | 0.993 |
| c43255 | N-acetyl-1-methylhistidine* | -0.10 | -0.41, 0.21 | 0.527 | 0.941 | -0.08 | -0.38, 0.22 | 0.610 | 0.980 |
| c40473 | hydantoin-5-propionate | -0.02 | -0.28, 0.24 | 0.879 | 0.967 | -0.10 | -0.36, 0.16 | 0.442 | 0.980 |
| c607 | trans-urocanate | -0.28 | -0.64, 0.07 | 0.121 | 0.844 | -0.29 | -0.63, 0.05 | 0.101 | 0.940 |
| c40730 | imidazole propionate | 0.02 | -0.29, 0.34 | 0.880 | 0.967 | -0.02 | -0.32, 0.27 | 0.872 | 0.993 |
| c15716 | imidazole lactate | -0.10 | -0.74, 0.55 | 0.769 | 0.951 | -0.15 | -0.77, 0.48 | 0.648 | 0.980 |
| c43488 | N-acetylcarnosine | -0.09 | -0.58, 0.41 | 0.733 | 0.949 | 0.01 | -0.48, 0.50 | 0.961 | 0.993 |
| c32350 | 1-methyl-4-imidazoleacetate | 0.06 | -0.77, 0.89 | 0.890 | 0.973 | 0.46 | -0.37, 1.28 | 0.279 | 0.940 |
|  | **Lysine Metabolism** |  |  |  |  |  |  |  |  |
| c1301 | lysine | -1.17 | -2.30,-0.04 | 0.044 | 0.628 | -1.13 | -2.23,-0.03 | 0.046 | 0.940 |
| c36752 | N6-acetyllysine | -0.67 | -1.45, 0.10 | 0.091 | 0.809 | -0.46 | -1.23, 0.30 | 0.236 | 0.940 |
| c1498 | N6,N6,N6-trimethyllysine | -0.29 | -0.87, 0.30 | 0.341 | 0.919 | -0.08 | -0.67, 0.51 | 0.795 | 0.993 |
| c15685 | 5-hydroxylysine | 0 | -0.47, 0.46 | 0.998 | 0.998 | 0.04 | -0.43, 0.51 | 0.874 | 0.993 |
| c44664 | glutarylcarnitine (C5-DC) | 0.18 | -0.34, 0.71 | 0.497 | 0.941 | 0.06 | -0.46, 0.58 | 0.827 | 0.993 |
| c1444 | pipecolate | -0.20 | -0.52, 0.12 | 0.214 | 0.870 | -0.40 | -0.71,-0.08 | 0.014 | 0.935 |
|  | **Phenylalanine Metabolism** |  |  |  |  |  |  |  |  |
| c64 | phenylalanine | 0.03 | -0.73, 0.79 | 0.935 | 0.985 | -0.07 | -0.81, 0.68 | 0.862 | 0.993 |
| c33950 | N-acetylphenylalanine | 0.08 | -0.43, 0.59 | 0.761 | 0.951 | 0.15 | -0.35, 0.64 | 0.560 | 0.980 |
| c566 | phenylpyruvate | -0.08 | -0.36, 0.20 | 0.577 | 0.941 | -0.07 | -0.34, 0.20 | 0.635 | 0.980 |
| c22130 | phenyllactate (PLA) | 0.33 | -0.41, 1.08 | 0.383 | 0.919 | 0.15 | -0.57, 0.87 | 0.680 | 0.980 |
|  | **Tyrosine Metabolism** |  |  |  |  |  |  |  |  |
| c1299 | tyrosine | 0.63 | -0.25, 1.51 | 0.164 | 0.844 | 0.54 | -0.32, 1.40 | 0.218 | 0.940 |
| c32197 | 3-(4-hydroxyphenyl)lactate (HPLA) | 0.58 | -0.21, 1.37 | 0.155 | 0.844 | 0.5 | -0.26, 1.27 | 0.200 | 0.940 |
| c32553 | phenol sulfate | 0.01 | -0.39, 0.42 | 0.944 | 0.987 | 0.08 | -0.32, 0.47 | 0.703 | 0.981 |
| c1567 | vanillylmandelate (VMA) | 0.17 | -0.63, 0.97 | 0.683 | 0.941 | 0.49 | -0.29, 1.27 | 0.222 | 0.940 |
| c12017 | 3-methoxytyrosine | -0.16 | -0.82, 0.49 | 0.623 | 0.941 | -0.21 | -0.84, 0.41 | 0.506 | 0.980 |
| c48841 | p-cresol glucuronide* | 0.05 | -0.12, 0.22 | 0.546 | 0.941 | 0.02 | -0.15, 0.19 | 0.795 | 0.993 |
| c2761 | thyroxine | -0.47 | -1.39, 0.46 | 0.322 | 0.919 | -0.29 | -1.20, 0.62 | 0.536 | 0.980 |
|  | **Tryptophan Metabolism** |  |  |  |  |  |  |  |  |
| c54 | tryptophan | -0.45 | -1.36, 0.47 | 0.339 | 0.919 | -0.32 | -1.20, 0.57 | 0.484 | 0.980 |
| c33959 | N-acetyltryptophan | 0.05 | -0.35, 0.44 | 0.809 | 0.951 | 0.19 | -0.20, 0.58 | 0.334 | 0.968 |
| c48782 | C-glycosyltryptophan | -1.16 | -2.34, 0.01 | 0.055 | 0.744 | -0.83 | -2.02, 0.35 | 0.168 | 0.940 |
| c37097 | tryptophan betaine | 0.12 | -0.08, 0.32 | 0.254 | 0.894 | 0.22 | 0.02, 0.43 | 0.033 | 0.940 |
| c15140 | kynurenine | -0.28 | -0.89, 0.33 | 0.373 | 0.919 | -0.09 | -0.69, 0.52 | 0.779 | 0.993 |
| c1417 | kynurenate | -0.12 | -0.60, 0.37 | 0.637 | 0.941 | -0.06 | -0.53, 0.40 | 0.788 | 0.993 |
| c15679 | xanthurenate | 0.06 | -0.25, 0.36 | 0.713 | 0.949 | 0.06 | -0.24, 0.35 | 0.704 | 0.981 |
| c2342 | serotonin | 0.15 | -0.16, 0.47 | 0.342 | 0.919 | 0.10 | -0.21, 0.41 | 0.521 | 0.980 |
| c18349 | indolelactate | 0.27 | -0.48, 1.02 | 0.483 | 0.941 | 0.32 | -0.41, 1.05 | 0.388 | 0.980 |
| c27513 | indoleacetate | -0.04 | -0.57, 0.48 | 0.876 | 0.967 | 0.03 | -0.48, 0.54 | 0.907 | 0.993 |
| c32405 | indolepropionate | 0 | -0.28, 0.27 | 0.981 | 0.991 | -0.09 | -0.37, 0.18 | 0.496 | 0.980 |
| c27672 | 3-indoxyl sulfate | 0.27 | -0.21, 0.75 | 0.270 | 0.895 | 0.27 | -0.20, 0.73 | 0.260 | 0.940 |
|  | **Leucine, Isoleucine and Valine Metabolism** |  |  |  |  |  |  |  |  |
| c60 | leucine | 0.24 | -0.99, 1.46 | 0.707 | 0.949 | 0.24 | -0.96, 1.43 | 0.698 | 0.981 |
| c22116 | 4-methyl-2-oxopentanoate | 0.10 | -0.33, 0.52 | 0.652 | 0.941 | 0.12 | -0.27, 0.52 | 0.546 | 0.980 |
| c44656 | isovalerate (C5) | -0.10 | -0.30, 0.10 | 0.326 | 0.919 | -0.15 | -0.32, 0.02 | 0.080 | 0.940 |
| c34407 | isovalerylcarnitine (C5) | -0.28 | -0.77, 0.22 | 0.274 | 0.895 | -0.46 | -0.96, 0.03 | 0.069 | 0.940 |
| c12129 | beta-hydroxyisovalerate | 0.19 | -0.40, 0.77 | 0.538 | 0.941 | 0.02 | -0.58, 0.61 | 0.953 | 0.993 |
| c46548 | 3-methylglutarylcarnitine (2) | -0.16 | -0.55, 0.22 | 0.413 | 0.920 | -0.02 | -0.43, 0.39 | 0.920 | 0.993 |
| c1125 | isoleucine | 0.16 | -1.03, 1.35 | 0.794 | 0.951 | 0.15 | -1.01, 1.30 | 0.804 | 0.993 |
| c15676 | 3-methyl-2-oxovalerate | 0 | -0.43, 0.44 | 0.983 | 0.991 | 0.02 | -0.39, 0.43 | 0.925 | 0.993 |
| c36746 | 2-hydroxy-3-methylvalerate | -0.10 | -0.59, 0.39 | 0.684 | 0.941 | -0.06 | -0.53, 0.42 | 0.819 | 0.993 |
| c45095 | 2-methylbutyrylcarnitine (C5) | -0.29 | -0.94, 0.36 | 0.376 | 0.919 | -0.27 | -0.91, 0.36 | 0.400 | 0.980 |
| c32397 | 3-hydroxy-2-ethylpropionate | -0.52 | -1.20, 0.16 | 0.136 | 0.844 | -0.53 | -1.19, 0.13 | 0.116 | 0.940 |
| c15765 | ethylmalonate | -0.29 | -0.68, 0.10 | 0.150 | 0.844 | -0.14 | -0.53, 0.25 | 0.486 | 0.980 |
| c53031 | methylsuccinoylcarnitine | -0.17 | -0.49, 0.14 | 0.283 | 0.898 | -0.06 | -0.38, 0.25 | 0.690 | 0.981 |
| c1649 | valine | -0.53 | -1.52, 0.47 | 0.300 | 0.907 | -0.42 | -1.39, 0.54 | 0.392 | 0.980 |
| c1591 | N-acetylvaline | -0.15 | -1.14, 0.85 | 0.771 | 0.951 | 0.02 | -0.94, 0.98 | 0.970 | 0.993 |
| c44526 | 3-methyl-2-oxobutyrate | 0.12 | -0.26, 0.49 | 0.544 | 0.941 | 0.20 | -0.12, 0.52 | 0.232 | 0.940 |
| c33937 | alpha-hydroxyisovalerate | -0.07 | -0.71, 0.58 | 0.840 | 0.967 | -0.10 | -0.72, 0.52 | 0.757 | 0.988 |
| c33441 | isobutyrylcarnitine (C4) | 0.10 | -0.34, 0.54 | 0.649 | 0.941 | 0.06 | -0.37, 0.49 | 0.783 | 0.993 |
|  | **Methionine, Cysteine, SAM and Taurine Metabolism** | |  |  |  |  |  |  |  |
| c1302 | methionine | 0.28 | -0.45, 1.01 | 0.458 | 0.941 | 0.21 | -0.50, 0.91 | 0.567 | 0.980 |
| c1589 | N-acetylmethionine | 0.12 | -0.06, 0.31 | 0.191 | 0.844 | 0.11 | -0.07, 0.29 | 0.230 | 0.940 |
| c2829 | N-formylmethionine | 0.22 | -0.38, 0.83 | 0.470 | 0.941 | 0.32 | -0.25, 0.88 | 0.274 | 0.940 |
| c44878 | methionine sulfone | -0.49 | -0.95,-0.03 | 0.040 | 0.628 | -0.45 | -0.91, 0.01 | 0.057 | 0.940 |
| c18374 | methionine sulfoxide | 0.06 | -0.22, 0.35 | 0.670 | 0.941 | -0.04 | -0.30, 0.23 | 0.795 | 0.993 |
| c45428 | N-acetylmethionine sulfoxide | 0.06 | -0.11, 0.23 | 0.513 | 0.941 | 0.04 | -0.13, 0.20 | 0.656 | 0.980 |
| c42382 | S-adenosylhomocysteine (SAH) | -0.12 | -0.42, 0.18 | 0.424 | 0.921 | -0.06 | -0.35, 0.22 | 0.669 | 0.980 |
| c15705 | cystathionine | 0 | -0.19, 0.20 | 0.961 | 0.991 | 0.06 | -0.14, 0.25 | 0.572 | 0.980 |
| c39592 | S-methylcysteine | 0.02 | -0.34, 0.39 | 0.894 | 0.976 | -0.06 | -0.41, 0.30 | 0.755 | 0.988 |
| c22176 | cysteine s-sulfate | -0.14 | -0.41, 0.14 | 0.337 | 0.919 | -0.10 | -0.38, 0.17 | 0.453 | 0.980 |
| c37443 | cysteine sulfinic acid | 0.10 | -0.33, 0.53 | 0.664 | 0.941 | -0.02 | -0.43, 0.38 | 0.908 | 0.993 |
| c590 | hypotaurine | 0.13 | -0.18, 0.44 | 0.418 | 0.920 | 0.13 | -0.17, 0.43 | 0.410 | 0.980 |
| c2125 | taurine | 0.10 | -0.41, 0.61 | 0.700 | 0.949 | 0.10 | -0.40, 0.59 | 0.701 | 0.981 |
|  | **Urea cycle; Arginine and Proline Metabolism** |  |  |  |  |  |  |  |  |
| c1638 | arginine | -0.21 | -0.90, 0.48 | 0.552 | 0.941 | -0.40 | -1.06, 0.26 | 0.237 | 0.940 |
| c1670 | urea | 0.16 | -0.58, 0.90 | 0.677 | 0.941 | 0.02 | -0.70, 0.75 | 0.953 | 0.993 |
| c1493 | ornithine | 0.06 | -0.58, 0.70 | 0.861 | 0.967 | 0.12 | -0.50, 0.74 | 0.707 | 0.982 |
| c55072 | 2-oxoarginine* | -0.04 | -0.34, 0.26 | 0.792 | 0.951 | -0.04 | -0.33, 0.24 | 0.760 | 0.988 |
| c2132 | citrulline | -0.49 | -1.28, 0.29 | 0.218 | 0.870 | -0.37 | -1.13, 0.38 | 0.336 | 0.968 |
| c22137 | homoarginine | -0.17 | -0.71, 0.37 | 0.535 | 0.941 | -0.36 | -0.88, 0.17 | 0.186 | 0.940 |
| c1898 | proline | 0.11 | -0.60, 0.81 | 0.770 | 0.951 | 0.06 | -0.63, 0.74 | 0.873 | 0.993 |
| c36808 | dimethylarginine (ADMA + SDMA) | -1.09 | -2.30, 0.11 | 0.077 | 0.776 | -0.99 | -2.21, 0.22 | 0.109 | 0.940 |
| c33953 | N-acetylarginine | 0.06 | -0.42, 0.55 | 0.793 | 0.951 | 0.08 | -0.39, 0.54 | 0.739 | 0.988 |
| c43249 | N-delta-acetylornithine | -0.09 | -0.43, 0.25 | 0.602 | 0.941 | -0.08 | -0.41, 0.25 | 0.647 | 0.980 |
| c32306 | hydroxyproline | -0.09 | -0.78, 0.61 | 0.810 | 0.951 | -0.11 | -0.80, 0.58 | 0.759 | 0.988 |
| c35127 | prolylhydroxyproline | 0.18 | 0.01, 0.35 | 0.042 | 0.628 | 0.15 | -0.01, 0.32 | 0.072 | 0.940 |
| c37431 | N-methylproline | 0.04 | -0.15, 0.23 | 0.690 | 0.941 | 0.09 | -0.10, 0.27 | 0.374 | 0.980 |
|  | **Creatine Metabolism** |  |  |  |  |  |  |  |  |
| c27718 | creatine | -0.20 | -0.78, 0.38 | 0.500 | 0.941 | -0.21 | -0.78, 0.36 | 0.474 | 0.980 |
| c513 | creatinine | 0.66 | -0.74, 2.06 | 0.358 | 0.919 | 0.45 | -0.91, 1.81 | 0.518 | 0.980 |
|  | **Polyamine Metabolism** |  |  |  |  |  |  |  |  |
| c37496 | N-acetylputrescine | -0.13 | -0.60, 0.35 | 0.613 | 0.941 | -0.05 | -0.50, 0.40 | 0.843 | 0.993 |
| c485 | spermidine | -0.29 | -0.50,-0.07 | 0.011 | 0.577 | -0.32 | -0.53,-0.10 | 0.005 | 0.935 |
| c1419 | 5-methylthioadenosine (MTA) | 0.05 | -0.19, 0.29 | 0.690 | 0.941 | 0.02 | -0.22, 0.25 | 0.891 | 0.993 |
|  | **Guanidino and Acetamido Metabolism** |  |  |  |  |  |  |  |  |
| c15681 | 4-guanidinobutanoate | -0.17 | -0.40, 0.06 | 0.160 | 0.844 | -0.15 | -0.37, 0.07 | 0.189 | 0.940 |
|  | **Glutathione Metabolism** |  |  |  |  |  |  |  |  |
| c35637 | cysteinylglycine | 0.07 | -0.14, 0.29 | 0.519 | 0.941 | 0.14 | -0.06, 0.35 | 0.163 | 0.940 |
| c1494 | 5-oxoproline | 0.11 | -0.26, 0.49 | 0.556 | 0.941 | 0.20 | -0.17, 0.56 | 0.294 | 0.940 |
| c42374 | 2-aminobutyrate | 0.16 | -0.41, 0.74 | 0.578 | 0.941 | 0.05 | -0.52, 0.62 | 0.863 | 0.993 |
| c52281 | 2-hydroxybutyrate/2-hydroxyisobutyrate | 0.01 | -0.48, 0.50 | 0.962 | 0.991 | -0.03 | -0.51, 0.44 | 0.888 | 0.993 |
|  | **PEPTIDE** |  |  |  |  |  |  |  |  |
|  | **Gamma-glutamyl Amino Acid** |  |  |  |  |  |  |  |  |
| c37063 | gamma-glutamylalanine | 0.07 | -0.22, 0.35 | 0.643 | 0.941 | 0.01 | -0.26, 0.28 | 0.949 | 0.993 |
| c36738 | gamma-glutamylglutamate | 0.07 | -0.11, 0.25 | 0.438 | 0.930 | 0.05 | -0.13, 0.22 | 0.601 | 0.980 |
| c2730 | gamma-glutamylglutamine | 0.15 | -0.16, 0.45 | 0.351 | 0.919 | 0.09 | -0.21, 0.38 | 0.570 | 0.980 |
| c33949 | gamma-glutamylglycine | 0.19 | -0.07, 0.45 | 0.152 | 0.844 | 0.14 | -0.12, 0.40 | 0.300 | 0.940 |
| c34456 | gamma-glutamylisoleucine* | 0.08 | -0.16, 0.31 | 0.512 | 0.941 | 0.06 | -0.17, 0.29 | 0.607 | 0.980 |
| c18369 | gamma-glutamylleucine | 0.06 | -0.23, 0.35 | 0.691 | 0.941 | 0.04 | -0.24, 0.32 | 0.790 | 0.993 |
| c55015 | gamma-glutamyl-alpha-lysine | 0.01 | -0.33, 0.36 | 0.944 | 0.987 | -0.07 | -0.41, 0.26 | 0.661 | 0.980 |
| c44872 | gamma-glutamylmethionine | 0.16 | -0.10, 0.42 | 0.228 | 0.870 | 0.12 | -0.13, 0.37 | 0.346 | 0.968 |
| c33422 | gamma-glutamylphenylalanine | -0.19 | -0.47, 0.09 | 0.188 | 0.844 | -0.17 | -0.44, 0.09 | 0.211 | 0.940 |
| c33364 | gamma-glutamylthreonine | 0.15 | -0.19, 0.49 | 0.379 | 0.919 | 0.13 | -0.21, 0.46 | 0.461 | 0.980 |
| c2734 | gamma-glutamyltyrosine | 0.02 | -0.39, 0.42 | 0.927 | 0.983 | 0.01 | -0.39, 0.42 | 0.956 | 0.993 |
| c43829 | gamma-glutamylvaline | 0.11 | -0.13, 0.35 | 0.367 | 0.919 | 0.09 | -0.15, 0.33 | 0.456 | 0.980 |
| c54914 | gamma-glutamylserine | 0.17 | -0.19, 0.52 | 0.363 | 0.919 | 0.07 | -0.27, 0.41 | 0.691 | 0.981 |
|  | **Dipeptide** |  |  |  |  |  |  |  |  |
| c42027 | histidylalanine | -0.04 | -0.23, 0.14 | 0.644 | 0.941 | -0.04 | -0.22, 0.14 | 0.648 | 0.980 |
| c40010 | leucylalanine | -0.07 | -0.21, 0.08 | 0.357 | 0.919 | -0.06 | -0.20, 0.08 | 0.402 | 0.980 |
| c40045 | leucylglycine | 0.03 | -0.20, 0.26 | 0.793 | 0.951 | 0 | -0.22, 0.22 | 0.994 | 0.999 |
| c39994 | valylleucine | -0.06 | -0.24, 0.12 | 0.497 | 0.941 | -0.10 | -0.27, 0.06 | 0.237 | 0.940 |
|  | Acetylated Peptides |  |  |  |  |  |  |  |  |
| c48425 | phenylacetylcarnitine | 0.03 | -0.20, 0.26 | 0.811 | 0.951 | -0.01 | -0.23, 0.21 | 0.934 | 0.993 |
| c35126 | phenylacetylglutamine | 0.29 | -0.07, 0.66 | 0.119 | 0.844 | 0.20 | -0.16, 0.56 | 0.276 | 0.940 |
| c55017 | 4-hydroxyphenylacetylglutamine | -0.37 | -0.64,-0.11 | 0.007 | 0.577 | -0.32 | -0.58,-0.06 | 0.016 | 0.935 |
|  | **CARBOHYDRATE** |  |  |  |  |  |  |  |  |
|  | **Glycolysis, Gluconeogenesis, and Pyruvate Metabolism** | |  |  |  |  |  |  |  |
| c20675 | 1,5-anhydroglucitol (1,5-AG) | 0.12 | -0.61, 0.84 | 0.752 | 0.951 | 0.36 | -0.36, 1.08 | 0.330 | 0.962 |
| c48152 | glucose | -0.03 | -1.85, 1.79 | 0.975 | 0.991 | 0.44 | -1.34, 2.22 | 0.628 | 0.980 |
| c48990 | pyruvate | 0.06 | -0.13, 0.26 | 0.510 | 0.941 | 0.12 | -0.05, 0.28 | 0.172 | 0.940 |
| c527 | lactate | 0.35 | -0.29, 0.98 | 0.285 | 0.898 | 0.34 | -0.28, 0.95 | 0.285 | 0.940 |
| c1572 | glycerate | 0.08 | -0.47, 0.63 | 0.775 | 0.951 | 0.03 | -0.50, 0.56 | 0.917 | 0.993 |
|  | **Pentose Metabolism** |  |  |  |  |  |  |  |  |
| c15772 | ribitol | -0.38 | -1.52, 0.76 | 0.518 | 0.941 | -0.29 | -1.41, 0.83 | 0.609 | 0.980 |
| c48885 | arabitol/xylitol | -0.23 | -1.00, 0.54 | 0.557 | 0.941 | -0.02 | -0.79, 0.76 | 0.967 | 0.993 |
| c48255 | arabonate/xylonate | -0.02 | -0.54, 0.51 | 0.950 | 0.990 | 0.06 | -0.45, 0.58 | 0.815 | 0.993 |
|  | **Glycogen Metabolism** |  |  |  |  |  |  |  |  |
| c15586 | maltose | 0.17 | -0.02, 0.37 | 0.089 | 0.809 | 0.21 | 0.03, 0.40 | 0.026 | 0.940 |
|  | **Disaccharides and Oligosaccharides** |  |  |  |  |  |  |  |  |
| c1519 | sucrose | 0.10 | -0.14, 0.33 | 0.419 | 0.920 | 0.15 | -0.07, 0.38 | 0.192 | 0.940 |
|  | **Fructose, Mannose and Galactose Metabolism** |  |  |  |  |  |  |  |  |
| c48195 | fructose | 0.02 | -0.47, 0.51 | 0.929 | 0.983 | 0.02 | -0.46, 0.49 | 0.948 | 0.993 |
| c46142 | mannitol/sorbitol | 0.01 | -0.36, 0.38 | 0.974 | 0.991 | 0.02 | -0.35, 0.38 | 0.931 | 0.993 |
| c48153 | mannose | -0.30 | -1.05, 0.45 | 0.441 | 0.930 | -0.18 | -0.92, 0.55 | 0.628 | 0.980 |
| c27719 | galactonate | 0.08 | -0.06, 0.23 | 0.269 | 0.895 | 0.09 | -0.05, 0.23 | 0.219 | 0.940 |
|  | **Aminosugar Metabolism** |  |  |  |  |  |  |  |  |
| c15443 | glucuronate | -0.24 | -0.92, 0.45 | 0.500 | 0.941 | 0.06 | -0.62, 0.75 | 0.852 | 0.993 |
| c42420 | erythronate* | 0.09 | -0.62, 0.81 | 0.797 | 0.951 | 0.11 | -0.60, 0.82 | 0.762 | 0.988 |
|  | **ENERGY** |  |  |  |  |  |  |  |  |
|  | **TCA Cycle** |  |  |  |  |  |  |  |  |
| c1564 | citrate | 0.62 | -0.07, 1.30 | 0.080 | 0.776 | 0.68 | 0.01, 1.36 | 0.047 | 0.940 |
| c528 | alpha-ketoglutarate | 0.05 | -0.14, 0.24 | 0.638 | 0.941 | 0.11 | -0.05, 0.28 | 0.194 | 0.940 |
| c37058 | succinylcarnitine (C4-DC) | 0.06 | -0.69, 0.81 | 0.875 | 0.967 | 0.04 | -0.68, 0.77 | 0.904 | 0.993 |
| c1437 | succinate | -0.30 | -0.85, 0.26 | 0.295 | 0.899 | -0.14 | -0.68, 0.40 | 0.611 | 0.980 |
| c1303 | malate | 0.22 | -0.39, 0.83 | 0.484 | 0.941 | 0.40 | -0.21, 1.01 | 0.199 | 0.940 |
| c52282 | 2-methylcitrate/homocitrate | -0.18 | -0.78, 0.43 | 0.569 | 0.941 | -0.16 | -0.75, 0.43 | 0.589 | 0.980 |
|  | **Oxidative Phosphorylation** |  |  |  |  |  |  |  |  |
| c42109 | phosphate | 0.42 | 0.06, 0.78 | 0.023 | 0.628 | 0.40 | 0.03, 0.76 | 0.034 | 0.940 |
|  | **LIPID** |  |  |  |  |  |  |  |  |
|  | **Medium Chain Fatty Acid** |  |  |  |  |  |  |  |  |
| c32489 | caproate (6:0) | 0.15 | -0.18, 0.48 | 0.367 | 0.919 | 0.09 | -0.25, 0.42 | 0.607 | 0.980 |
| c1644 | heptanoate (7:0) | 0.23 | -0.11, 0.56 | 0.190 | 0.844 | 0.14 | -0.20, 0.47 | 0.431 | 0.980 |
| c32492 | caprylate (8:0) | 0.34 | -0.26, 0.94 | 0.268 | 0.895 | 0.34 | -0.25, 0.92 | 0.260 | 0.940 |
| c1642 | caprate (10:0) | 0.16 | -0.34, 0.66 | 0.526 | 0.941 | 0.19 | -0.30, 0.67 | 0.450 | 0.980 |
| c32497 | 10-undecenoate (11:1n1) | -0.09 | -0.56, 0.37 | 0.688 | 0.941 | 0 | -0.45, 0.44 | 0.989 | 0.999 |
| c1645 | laurate (12:0) | 0.04 | -0.33, 0.42 | 0.828 | 0.961 | 0.13 | -0.23, 0.50 | 0.474 | 0.980 |
| c33968 | 5-dodecenoate (12:1n7) | -0.30 | -0.68, 0.07 | 0.114 | 0.844 | -0.20 | -0.57, 0.17 | 0.291 | 0.940 |
|  | **Long Chain Saturated Fatty Acid** |  |  |  |  |  |  |  |  |
| c1365 | myristate (14:0) | -0.26 | -0.69, 0.18 | 0.245 | 0.891 | -0.10 | -0.53, 0.32 | 0.632 | 0.980 |
| c1336 | palmitate (16:0) | -0.69 | -1.22,-0.16 | 0.012 | 0.577 | -0.52 | -1.05, 0.01 | 0.055 | 0.940 |
| c1121 | margarate (17:0) | -0.61 | -1.16,-0.06 | 0.030 | 0.628 | -0.48 | -1.02, 0.05 | 0.079 | 0.940 |
| c1358 | stearate (18:0) | -0.35 | -1.05, 0.35 | 0.326 | 0.919 | -0.12 | -0.80, 0.57 | 0.738 | 0.988 |
|  | **Long Chain Monounsaturated Fatty Acid** |  |  |  |  |  |  |  |  |
| c32418 | myristoleate (14:1n5) | -0.24 | -0.59, 0.12 | 0.190 | 0.844 | -0.11 | -0.47, 0.24 | 0.539 | 0.980 |
| c33447 | palmitoleate (16:1n7) | -0.4 | -0.74,-0.07 | 0.018 | 0.628 | -0.28 | -0.63, 0.06 | 0.106 | 0.940 |
| c33971 | 10-heptadecenoate (17:1n7) | -0.46 | -0.82,-0.10 | 0.014 | 0.577 | -0.34 | -0.71, 0.02 | 0.066 | 0.940 |
| c52285 | oleate/vaccenate (18:1) | -0.54 | -0.96,-0.12 | 0.013 | 0.577 | -0.41 | -0.83, 0.01 | 0.059 | 0.940 |
| c33972 | 10-nonadecenoate (19:1n9) | -0.61 | -1.04,-0.18 | 0.006 | 0.577 | -0.48 | -0.90,-0.06 | 0.028 | 0.940 |
|  | **Long Chain Polyunsaturated Fatty Acid (n3 and n6)** | |  |  |  |  |  |  |  |
| c33969 | stearidonate (18:4n3) | -0.07 | -0.32, 0.19 | 0.598 | 0.941 | -0.01 | -0.26, 0.25 | 0.961 | 0.993 |
| c18467 | eicosapentaenoate (EPA; 20:5n3) | -0.20 | -0.52, 0.13 | 0.233 | 0.870 | -0.07 | -0.39, 0.26 | 0.681 | 0.980 |
| c32504 | docosapentaenoate (DPA; 22:5n3) | -0.40 | -0.79,-0.02 | 0.043 | 0.628 | -0.23 | -0.62, 0.15 | 0.237 | 0.940 |
| c44675 | docosahexaenoate (DHA; 22:6n3) | -0.34 | -0.74, 0.07 | 0.104 | 0.844 | -0.28 | -0.68, 0.12 | 0.169 | 0.940 |
| c1105 | linoleate (18:2n6) | -0.42 | -0.83,-0.01 | 0.044 | 0.628 | -0.30 | -0.70, 0.11 | 0.153 | 0.940 |
| c34035 | linolenate (18:3n3 or 3n6) | -0.34 | -0.71, 0.02 | 0.064 | 0.749 | -0.26 | -0.62, 0.10 | 0.157 | 0.940 |
| c17805 | dihomolinoleate (20:2n6) | -0.51 | -0.95,-0.08 | 0.022 | 0.628 | -0.40 | -0.83, 0.03 | 0.069 | 0.940 |
| c35718 | dihomolinolenate (20:3n3 or 3n6) | -0.41 | -0.80,-0.01 | 0.044 | 0.628 | -0.26 | -0.66, 0.14 | 0.199 | 0.940 |
| c1110 | arachidonate (20:4n6) | -0.27 | -0.67, 0.13 | 0.180 | 0.844 | -0.14 | -0.53, 0.26 | 0.502 | 0.980 |
| c37478 | docosapentaenoate (n6 DPA; 22:5n6) | -0.42 | -0.83,-0.02 | 0.041 | 0.628 | -0.31 | -0.71, 0.09 | 0.133 | 0.940 |
|  | **Fatty Acid, Branched** |  |  |  |  |  |  |  |  |
| c38768 | (14 or 15)-methylpalmitate (a17:0 or i17:0) | -0.21 | -0.53, 0.10 | 0.187 | 0.844 | -0.18 | -0.48, 0.13 | 0.256 | 0.940 |
|  | **Fatty Acid, Dicarboxylate** |  |  |  |  |  |  |  |  |
| c396 | glutarate (C5-DC) | 0.04 | -0.19, 0.27 | 0.743 | 0.949 | -0.04 | -0.25, 0.17 | 0.722 | 0.988 |
| c37253 | 2-hydroxyglutarate | -0.01 | -0.44, 0.42 | 0.972 | 0.991 | -0.09 | -0.49, 0.31 | 0.668 | 0.980 |
| c32398 | sebacate (C10-DC) | 0.01 | -0.16, 0.18 | 0.907 | 0.980 | -0.03 | -0.19, 0.12 | 0.678 | 0.980 |
| c35678 | hexadecanedioate (C16) | -0.24 | -0.66, 0.18 | 0.271 | 0.895 | -0.15 | -0.56, 0.26 | 0.467 | 0.980 |
| c36754 | octadecanedioate (C18) | -0.20 | -0.61, 0.21 | 0.333 | 0.919 | -0.12 | -0.52, 0.28 | 0.565 | 0.980 |
| c39831 | eicosanedioate (C20-DC) | 0.06 | -0.32, 0.44 | 0.772 | 0.951 | 0.03 | -0.35, 0.40 | 0.889 | 0.993 |
| c31787 | 3-carboxy-4-methyl-5-propyl-2-furanpropanoate (CMPF) | 0.10 | -0.14, 0.34 | 0.413 | 0.920 | 0.09 | -0.15, 0.32 | 0.472 | 0.980 |
|  | **Fatty Acid, Amino** |  |  |  |  |  |  |  |  |
| c43761 | 2-aminoheptanoate | 0.22 | -0.23, 0.68 | 0.334 | 0.919 | 0.37 | -0.06, 0.81 | 0.096 | 0.940 |
| c43343 | 2-aminooctanoate | 0.14 | -0.17, 0.45 | 0.381 | 0.919 | 0.25 | -0.05, 0.56 | 0.108 | 0.940 |
|  | **Fatty Acid Metabolism (also BCAA Metabolism)** |  |  |  |  |  |  |  |  |
| c32412 | butyrylcarnitine (C4) | -0.17 | -0.42, 0.08 | 0.178 | 0.844 | -0.10 | -0.34, 0.14 | 0.427 | 0.980 |
| c32452 | propionylcarnitine (C3) | -0.18 | -0.68, 0.33 | 0.494 | 0.941 | -0.10 | -0.59, 0.39 | 0.702 | 0.981 |
| c54907 | hexanoylglutamine | -0.17 | -0.39, 0.06 | 0.143 | 0.844 | -0.16 | -0.37, 0.06 | 0.157 | 0.940 |
| c32198 | acetylcarnitine (C2) | -0.15 | -0.58, 0.28 | 0.496 | 0.941 | -0.17 | -0.59, 0.24 | 0.422 | 0.980 |
| c32328 | hexanoylcarnitine (C6) | -0.31 | -0.71, 0.09 | 0.126 | 0.844 | -0.22 | -0.62, 0.18 | 0.281 | 0.940 |
| c33936 | octanoylcarnitine (C8) | -0.21 | -0.59, 0.17 | 0.282 | 0.898 | -0.16 | -0.54, 0.22 | 0.403 | 0.980 |
| c33941 | decanoylcarnitine (C10) | -0.22 | -0.56, 0.11 | 0.196 | 0.844 | -0.19 | -0.52, 0.15 | 0.277 | 0.940 |
| c34534 | laurylcarnitine (C12) | -0.40 | -0.76,-0.03 | 0.033 | 0.628 | -0.32 | -0.68, 0.03 | 0.077 | 0.940 |
| c33952 | myristoylcarnitine (C14) | -0.53 | -1.02,-0.03 | 0.041 | 0.628 | -0.26 | -0.77, 0.24 | 0.306 | 0.940 |
| c44681 | palmitoylcarnitine (C16) | -0.38 | -0.94, 0.19 | 0.192 | 0.844 | -0.19 | -0.74, 0.37 | 0.506 | 0.980 |
| c38178 | cis-4-decenoylcarnitine (C10:1) | -0.33 | -0.77, 0.11 | 0.145 | 0.844 | -0.24 | -0.67, 0.20 | 0.290 | 0.940 |
| c48182 | myristoleoylcarnitine (C14:1)* | -0.38 | -0.74,-0.03 | 0.034 | 0.628 | -0.29 | -0.64, 0.06 | 0.107 | 0.940 |
| c53223 | palmitoleoylcarnitine (C16:1)* | -0.61 | -1.08,-0.13 | 0.014 | 0.577 | -0.39 | -0.87, 0.10 | 0.120 | 0.940 |
| c35160 | oleoylcarnitine (C18:1) | -0.55 | -1.12, 0.02 | 0.061 | 0.749 | -0.25 | -0.83, 0.34 | 0.410 | 0.980 |
| c46223 | linoleoylcarnitine (C18:2)* | -0.25 | -0.83, 0.33 | 0.403 | 0.919 | -0.02 | -0.60, 0.56 | 0.945 | 0.993 |
| c52988 | adipoylcarnitine (C6-DC) | -0.13 | -0.46, 0.21 | 0.456 | 0.941 | -0.11 | -0.44, 0.22 | 0.525 | 0.980 |
| c53224 | pimeloylcarnitine/3-methyladipoylcarnitine (C7-DC) | -0.14 | -0.44, 0.17 | 0.382 | 0.919 | -0.19 | -0.48, 0.11 | 0.216 | 0.940 |
| c43264 | (R)-3-hydroxybutyrylcarnitine | -0.01 | -0.21, 0.20 | 0.931 | 0.983 | 0.01 | -0.18, 0.21 | 0.897 | 0.993 |
|  | **Carnitine Metabolism** |  |  |  |  |  |  |  |  |
| c36747 | deoxycarnitine | -0.22 | -1.26, 0.81 | 0.673 | 0.941 | -0.29 | -1.30, 0.72 | 0.576 | 0.980 |
| c15500 | carnitine | 0 | -0.50, 0.49 | 0.986 | 0.991 | -0.11 | -0.59, 0.37 | 0.665 | 0.980 |
|  | **Fatty Acid Metabolism (Acyl Choline)** |  |  |  |  |  |  |  |  |
| c53257 | palmitoloelycholine | -0.12 | -0.30, 0.06 | 0.192 | 0.844 | -0.03 | -0.21, 0.15 | 0.730 | 0.988 |
| c53262 | dihomo-linolenoyl-choline | -0.10 | -0.30, 0.10 | 0.352 | 0.919 | -0.03 | -0.23, 0.17 | 0.759 | 0.988 |
| c53263 | docosahexaenoylcholine | -0.05 | -0.27, 0.16 | 0.627 | 0.941 | -0.01 | -0.22, 0.20 | 0.918 | 0.993 |
|  | Fatty Acid, Monohydroxy |  |  |  |  |  |  |  |  |
| c22036 | 2-hydroxyoctanoate | 0.20 | -0.27, 0.67 | 0.404 | 0.919 | 0.25 | -0.20, 0.70 | 0.284 | 0.940 |
| c42489 | 2-hydroxydecanoate | -0.13 | -0.79, 0.54 | 0.714 | 0.949 | -0.12 | -0.79, 0.55 | 0.729 | 0.988 |
| c35675 | 2-hydroxypalmitate | -0.20 | -1.08, 0.68 | 0.652 | 0.941 | -0.06 | -0.93, 0.82 | 0.900 | 0.993 |
| c17945 | 2-hydroxystearate | -0.65 | -1.54, 0.25 | 0.158 | 0.844 | -0.51 | -1.39, 0.37 | 0.258 | 0.940 |
| c53230 | 3-hydroxyhexanoate | -0.27 | -0.76, 0.23 | 0.289 | 0.898 | -0.30 | -0.79, 0.18 | 0.216 | 0.940 |
| c22001 | 3-hydroxyoctanoate | -0.33 | -0.80, 0.15 | 0.182 | 0.844 | -0.35 | -0.83, 0.12 | 0.147 | 0.940 |
| c22053 | 3-hydroxydecanoate | -0.27 | -0.67, 0.14 | 0.201 | 0.844 | -0.27 | -0.68, 0.13 | 0.192 | 0.940 |
| c32457 | 3-hydroxylaurate | -0.26 | -0.63, 0.10 | 0.160 | 0.844 | -0.25 | -0.61, 0.11 | 0.181 | 0.940 |
| c39609 | 16-hydroxypalmitate | -0.46 | -1.04, 0.11 | 0.117 | 0.844 | -0.28 | -0.86, 0.29 | 0.330 | 0.962 |
|  | **Fatty Acid, Dihydroxy** |  |  |  |  |  |  |  |  |
| c38395 | 12,13-DiHOME | 0.09 | -0.20, 0.39 | 0.550 | 0.941 | 0.09 | -0.19, 0.38 | 0.521 | 0.980 |
|  | **Eicosanoid** |  |  |  |  |  |  |  |  |
| c37536 | 12-HETE | 0.07 | -0.09, 0.23 | 0.385 | 0.919 | 0.04 | -0.11, 0.20 | 0.580 | 0.980 |
|  | **Endocannabinoid** |  |  |  |  |  |  |  |  |
| c52608 | linoleoyl ethanolamide | -0.01 | -0.16, 0.14 | 0.901 | 0.980 | 0 | -0.14, 0.15 | 0.961 | 0.993 |
|  | **Inositol Metabolism** |  |  |  |  |  |  |  |  |
| c1124 | myo-inositol | 0.42 | -0.31, 1.16 | 0.262 | 0.895 | 0.55 | -0.16, 1.26 | 0.132 | 0.940 |
|  | **Phospholipid Metabolism** |  |  |  |  |  |  |  |  |
| c15506 | choline | 0.03 | -0.36, 0.43 | 0.881 | 0.967 | 0.12 | -0.27, 0.50 | 0.550 | 0.980 |
| c34396 | phosphocholine | 0.37 | -0.19, 0.92 | 0.197 | 0.844 | 0.32 | -0.22, 0.86 | 0.241 | 0.940 |
| c15990 | glycerophosphorylcholine (GPC) | -0.22 | -0.72, 0.29 | 0.403 | 0.919 | -0.09 | -0.57, 0.40 | 0.732 | 0.988 |
| c40406 | trimethylamine N-oxide | 0 | -0.31, 0.31 | 0.981 | 0.991 | -0.10 | -0.41, 0.20 | 0.506 | 0.980 |
|  | **Phosphatidylcholine (PC)** |  |  |  |  |  |  |  |  |
| c19130 | 1,2-dipalmitoyl-GPC (16:0/16:0) | 0.40 | -0.84, 1.63 | 0.528 | 0.941 | 0.38 | -0.85, 1.60 | 0.549 | 0.980 |
| c52470 | 1-palmitoyl-2-palmitoleoyl-GPC (16:0/16:1)* | -0.05 | -0.45, 0.36 | 0.828 | 0.961 | 0.02 | -0.38, 0.41 | 0.932 | 0.993 |
| c52461 | 1-palmitoyl-2-oleoyl-GPC (16:0/18:1) | 0 | -1.01, 1.01 | 0.997 | 0.998 | 0.15 | -0.83, 1.13 | 0.763 | 0.988 |
| c42446 | 1-palmitoyl-2-linoleoyl-GPC (16:0/18:2) | 0.30 | -0.74, 1.35 | 0.568 | 0.941 | 0.20 | -0.81, 1.21 | 0.694 | 0.981 |
| c52462 | 1-palmitoyl-2-arachidonoyl-GPC (16:0/20:4n6) | 0.26 | -0.71, 1.22 | 0.601 | 0.941 | 0.18 | -0.75, 1.11 | 0.710 | 0.983 |
| c52438 | 1-stearoyl-2-oleoyl-GPC (18:0/18:1) | -0.37 | -1.02, 0.29 | 0.274 | 0.895 | -0.19 | -0.84, 0.46 | 0.568 | 0.980 |
| c52452 | 1-stearoyl-2-linoleoyl-GPC (18:0/18:2)* | 0.01 | -1.14, 1.16 | 0.988 | 0.991 | 0.08 | -1.04, 1.20 | 0.888 | 0.993 |
| c42450 | 1-stearoyl-2-arachidonoyl-GPC (18:0/20:4) | 0.22 | -0.85, 1.29 | 0.690 | 0.941 | 0.19 | -0.84, 1.22 | 0.717 | 0.984 |
| c52453 | 1-oleoyl-2-linoleoyl-GPC (18:1/18:2)* | -0.72 | -1.71, 0.26 | 0.152 | 0.844 | -0.54 | -1.50, 0.42 | 0.274 | 0.940 |
| c52603 | 1,2-dilinoleoyl-GPC (18:2/18:2) | 0.27 | -0.34, 0.88 | 0.391 | 0.919 | 0.22 | -0.38, 0.82 | 0.478 | 0.980 |
| c53176 | 1-linoleoyl-2-linolenoyl-GPC (18:2/18:3)* | -0.06 | -0.43, 0.31 | 0.737 | 0.949 | -0.12 | -0.47, 0.24 | 0.523 | 0.980 |
| c52710 | 1-linoleoyl-2-arachidonoyl-GPC (18:2/20:4n6)* | -0.10 | -0.72, 0.51 | 0.740 | 0.949 | 0.03 | -0.55, 0.62 | 0.909 | 0.993 |
|  | **Phosphatidylethanolamine (PE)** |  |  |  |  |  |  |  |  |
| c19263 | 1-palmitoyl-2-oleoyl-GPE (16:0/18:1) | -0.28 | -0.64, 0.07 | 0.123 | 0.844 | -0.19 | -0.54, 0.16 | 0.290 | 0.940 |
| c42449 | 1-palmitoyl-2-linoleoyl-GPE (16:0/18:2) | -0.29 | -0.70, 0.11 | 0.155 | 0.844 | -0.23 | -0.62, 0.16 | 0.251 | 0.940 |
| c52464 | 1-palmitoyl-2-arachidonoyl-GPE (16:0/20:4)* | -0.13 | -0.55, 0.29 | 0.545 | 0.941 | -0.10 | -0.50, 0.30 | 0.632 | 0.980 |
| c42448 | 1-stearoyl-2-oleoyl-GPE (18:0/18:1) | -0.19 | -0.61, 0.23 | 0.367 | 0.919 | -0.07 | -0.47, 0.34 | 0.751 | 0.988 |
| c52446 | 1-stearoyl-2-linoleoyl-GPE (18:0/18:2)* | -0.31 | -0.75, 0.13 | 0.166 | 0.844 | -0.18 | -0.61, 0.25 | 0.407 | 0.980 |
| c52447 | 1-stearoyl-2-arachidonoyl-GPE (18:0/20:4) | -0.17 | -0.65, 0.31 | 0.489 | 0.941 | -0.07 | -0.54, 0.39 | 0.752 | 0.988 |
|  | **Phosphatidylinositol (PI)** |  |  |  |  |  |  |  |  |
| c52669 | 1-palmitoyl-2-oleoyl-GPI (16:0/18:1)* | -0.31 | -0.84, 0.22 | 0.251 | 0.892 | -0.11 | -0.63, 0.41 | 0.682 | 0.980 |
| c52450 | 1-palmitoyl-2-linoleoyl-GPI (16:0/18:2) | -0.20 | -0.76, 0.36 | 0.487 | 0.941 | 0.03 | -0.53, 0.59 | 0.919 | 0.993 |
| c52726 | 1-stearoyl-2-oleoyl-GPI (18:0/18:1)* | -0.13 | -0.63, 0.36 | 0.597 | 0.941 | 0 | -0.48, 0.49 | 0.995 | 0.999 |
| c52468 | 1-stearoyl-2-linoleoyl-GPI (18:0/18:2) | -0.37 | -1.06, 0.31 | 0.289 | 0.898 | -0.12 | -0.81, 0.57 | 0.736 | 0.988 |
| c52449 | 1-stearoyl-2-arachidonoyl-GPI (18:0/20:4) | -0.55 | -1.30, 0.20 | 0.153 | 0.844 | -0.24 | -0.98, 0.50 | 0.521 | 0.980 |
|  | **Lysophospholipid** |  |  |  |  |  |  |  |  |
| c33955 | 1-palmitoyl-GPC (16:0) | -0.29 | -0.87, 0.28 | 0.320 | 0.919 | -0.15 | -0.72, 0.41 | 0.594 | 0.980 |
| c33230 | 1-palmitoleoyl-GPC* (16:1)* | -0.28 | -0.73, 0.17 | 0.224 | 0.870 | -0.10 | -0.55, 0.35 | 0.669 | 0.980 |
| c47118 | 2-palmitoleoyl-GPC* (16:1)* | -0.10 | -0.34, 0.13 | 0.389 | 0.919 | -0.08 | -0.31, 0.14 | 0.467 | 0.980 |
| c33961 | 1-stearoyl-GPC (18:0) | -0.27 | -0.80, 0.26 | 0.325 | 0.919 | -0.13 | -0.65, 0.38 | 0.611 | 0.980 |
| c48258 | 1-oleoyl-GPC (18:1) | -0.23 | -0.74, 0.28 | 0.373 | 0.919 | -0.07 | -0.57, 0.42 | 0.775 | 0.993 |
| c34419 | 1-linoleoyl-GPC (18:2) | 0.13 | -0.68, 0.94 | 0.756 | 0.951 | 0.29 | -0.51, 1.10 | 0.478 | 0.980 |
| c45951 | 1-linolenoyl-GPC (18:3)* | 0.07 | -0.42, 0.55 | 0.785 | 0.951 | 0.13 | -0.35, 0.61 | 0.590 | 0.980 |
| c33228 | 1-arachidonoyl-GPC* (20:4)* | 0.03 | -0.58, 0.64 | 0.927 | 0.983 | 0.17 | -0.41, 0.75 | 0.571 | 0.980 |
| c49617 | 1-lignoceroyl-GPC (24:0) | -0.18 | -0.77, 0.40 | 0.540 | 0.941 | -0.02 | -0.61, 0.56 | 0.944 | 0.993 |
| c35631 | 1-palmitoyl-GPE (16:0) | -0.48 | -0.95,-0.02 | 0.043 | 0.628 | -0.38 | -0.84, 0.08 | 0.107 | 0.940 |
| c42398 | 1-stearoyl-GPE (18:0) | -0.28 | -0.74, 0.17 | 0.224 | 0.870 | -0.13 | -0.58, 0.32 | 0.576 | 0.980 |
| c35628 | 1-oleoyl-GPE (18:1) | -0.02 | -0.37, 0.33 | 0.910 | 0.982 | 0.13 | -0.21, 0.48 | 0.456 | 0.980 |
| c36600 | 1-linoleoyl-GPE (18:2)* | 0.12 | -0.34, 0.58 | 0.607 | 0.941 | 0.16 | -0.29, 0.61 | 0.482 | 0.980 |
| c35186 | 1-arachidonoyl-GPE (20:4n6)* | 0.25 | -0.35, 0.84 | 0.418 | 0.920 | 0.36 | -0.21, 0.94 | 0.219 | 0.940 |
| c19324 | 1-stearoyl-GPI (18:0) | -0.31 | -0.76, 0.15 | 0.196 | 0.844 | -0.17 | -0.62, 0.28 | 0.453 | 0.980 |
| c36594 | 1-linoleoyl-GPI* (18:2)* | -0.04 | -0.67, 0.59 | 0.906 | 0.980 | 0.04 | -0.58, 0.66 | 0.904 | 0.993 |
| c34214 | 1-arachidonoyl-GPI* (20:4)* | -0.13 | -0.75, 0.48 | 0.676 | 0.941 | 0.07 | -0.53, 0.67 | 0.819 | 0.993 |
|  | **Plasmalogen** |  |  |  |  |  |  |  |  |
| c52477 | 1-(1-enyl-palmitoyl)-2-oleoyl-GPE (P-16:0/18:1)* | -0.06 | -0.57, 0.44 | 0.814 | 0.952 | -0.16 | -0.66, 0.35 | 0.547 | 0.980 |
| c52677 | 1-(1-enyl-palmitoyl)-2-linoleoyl-GPE (P-16:0/18:2)* | -0.01 | -0.51, 0.49 | 0.978 | 0.991 | -0.12 | -0.62, 0.37 | 0.632 | 0.980 |
| c52716 | 1-(1-enyl-palmitoyl)-2-palmitoyl-GPC (P-16:0/16:0)* | 0.57 | -0.23, 1.37 | 0.164 | 0.844 | 0.25 | -0.56, 1.05 | 0.547 | 0.980 |
| c52713 | 1-(1-enyl-palmitoyl)-2-palmitoleoyl-GPC (P-16:0/16:1)* | 0.55 | -0.03, 1.13 | 0.063 | 0.749 | 0.35 | -0.22, 0.92 | 0.237 | 0.940 |
| c52673 | 1-(1-enyl-palmitoyl)-2-arachidonoyl-GPE (P-16:0/20:4)* | 0.26 | -0.23, 0.75 | 0.296 | 0.899 | 0.15 | -0.34, 0.63 | 0.554 | 0.980 |
| c52478 | 1-(1-enyl-palmitoyl)-2-oleoyl-GPC (P-16:0/18:1)* | 0.64 | -0.21, 1.50 | 0.141 | 0.844 | 0.33 | -0.53, 1.19 | 0.454 | 0.980 |
| c52614 | 1-(1-enyl-stearoyl)-2-oleoyl-GPE (P-18:0/18:1) | 0.11 | -0.41, 0.63 | 0.681 | 0.941 | 0 | -0.51, 0.51 | 0.996 | 0.999 |
| c52748 | 1-(1-enyl-stearoyl)-2-linoleoyl-GPE (P-18:0/18:2)* | 0.12 | -0.36, 0.59 | 0.634 | 0.941 | 0.01 | -0.47, 0.48 | 0.977 | 0.996 |
| c52689 | 1-(1-enyl-palmitoyl)-2-arachidonoyl-GPC (P-16:0/20:4)* | 0.56 | -0.05, 1.16 | 0.073 | 0.770 | 0.38 | -0.22, 0.98 | 0.220 | 0.940 |
| c52682 | 1-(1-enyl-palmitoyl)-2-linoleoyl-GPC (P-16:0/18:2)* | 0.44 | -0.36, 1.24 | 0.281 | 0.898 | 0.05 | -0.75, 0.86 | 0.898 | 0.993 |
| c52475 | 1-(1-enyl-stearoyl)-2-arachidonoyl-GPE (P-18:0/20:4)* | 0.25 | -0.22, 0.72 | 0.294 | 0.899 | 0.11 | -0.36, 0.57 | 0.658 | 0.980 |
|  | **Lysoplasmalogen** |  |  |  |  |  |  |  |  |
| c39270 | 1-(1-enyl-palmitoyl)-GPE (P-16:0)* | -0.15 | -0.52, 0.23 | 0.450 | 0.936 | -0.18 | -0.54, 0.19 | 0.343 | 0.968 |
| c39271 | 1-(1-enyl-stearoyl)-GPE (P-18:0)* | -0.08 | -0.48, 0.32 | 0.685 | 0.941 | -0.14 | -0.53, 0.24 | 0.473 | 0.980 |
|  | Glycerolipid Metabolism |  |  |  |  |  |  |  |  |
| c15122 | glycerol | -0.34 | -0.72, 0.04 | 0.083 | 0.780 | -0.23 | -0.61, 0.15 | 0.234 | 0.940 |
|  | **Monoacylglycerol** |  |  |  |  |  |  |  |  |
| c21184 | 1-oleoylglycerol (18:1) | -0.39 | -0.75,-0.03 | 0.034 | 0.628 | -0.21 | -0.57, 0.15 | 0.258 | 0.940 |
|  | **Diacylglycerol** |  |  |  |  |  |  |  |  |
| c46799 | oleoyl-linoleoyl-glycerol (18:1/18:2) [2] | -0.38 | -0.69,-0.07 | 0.016 | 0.608 | -0.25 | -0.56, 0.06 | 0.118 | 0.940 |
|  | Sphingolipid Synthesis |  |  |  |  |  |  |  |  |
| c52605 | sphinganine-1-phosphate | 0.21 | -0.13, 0.54 | 0.232 | 0.870 | 0.18 | -0.15, 0.50 | 0.288 | 0.940 |
|  | **Ceramides** |  |  |  |  |  |  |  |  |
| c44877 | N-palmitoyl-sphingosine (d18:1/16:0) | -0.67 | -1.61, 0.26 | 0.161 | 0.844 | -0.51 | -1.42, 0.41 | 0.279 | 0.940 |
| c54979 | N-stearoyl-sphingosine (d18:1/18:0)* | -0.36 | -0.92, 0.20 | 0.205 | 0.844 | -0.37 | -0.91, 0.17 | 0.184 | 0.940 |
|  | **Hexosylceramides (HCER)** |  |  |  |  |  |  |  |  |
| c53013 | glycosyl-N-palmitoyl-sphingosine (d18:1/16:0) | -0.15 | -1.03, 0.74 | 0.742 | 0.949 | -0.24 | -1.09, 0.61 | 0.586 | 0.980 |
| c52234 | glycosyl-N-stearoyl-sphingosine (d18:1/18:0) | -0.19 | -0.90, 0.52 | 0.606 | 0.941 | -0.13 | -0.82, 0.56 | 0.711 | 0.983 |
|  | **Lactosylceramides (LCER)** |  |  |  |  |  |  |  |  |
| c57370 | lactosyl-N-nervonoyl-sphingosine (d18:1/24:1)* | -0.17 | -0.92, 0.58 | 0.662 | 0.941 | -0.24 | -0.96, 0.49 | 0.523 | 0.980 |
|  | **Dihydrosphingomyelins** |  |  |  |  |  |  |  |  |
| c57365 | myristoyl dihydrosphingomyelin (d18:0/14:0)* | -0.52 | -1.21, 0.17 | 0.144 | 0.844 | -0.26 | -0.95, 0.43 | 0.469 | 0.980 |
| c52434 | palmitoyl dihydrosphingomyelin (d18:0/16:0)* | 0.72 | -0.33, 1.77 | 0.178 | 0.844 | 0.59 | -0.43, 1.61 | 0.256 | 0.940 |
| c57331 | behenoyl dihydrosphingomyelin (d18:0/22:0)* | 0 | -0.50, 0.51 | 0.986 | 0.991 | 0.15 | -0.34, 0.65 | 0.552 | 0.980 |
|  | Sphingomyelins |  |  |  |  |  |  |  |  |
| c37506 | palmitoyl sphingomyelin (d18:1/16:0) | 0.75 | -0.62, 2.11 | 0.287 | 0.898 | 0.49 | -0.84, 1.82 | 0.471 | 0.980 |
| c19503 | stearoyl sphingomyelin (d18:1/18:0) | 0.27 | -0.67, 1.21 | 0.579 | 0.941 | 0.01 | -0.92, 0.94 | 0.987 | 0.999 |
| c48492 | behenoyl sphingomyelin (d18:1/22:0)* | -0.05 | -1.21, 1.11 | 0.932 | 0.983 | 0.08 | -1.04, 1.20 | 0.889 | 0.993 |
| c52436 | tricosanoyl sphingomyelin (d18:1/23:0)* | -0.42 | -1.39, 0.56 | 0.402 | 0.919 | -0.29 | -1.24, 0.67 | 0.559 | 0.980 |
| c57330 | lignoceroyl sphingomyelin (d18:1/24:0) | 0.08 | -0.81, 0.98 | 0.854 | 0.967 | 0.08 | -0.80, 0.95 | 0.866 | 0.993 |
| c42463 | sphingomyelin (d18:1/14:0, d16:1/16:0)* | -0.58 | -1.53, 0.37 | 0.233 | 0.870 | -0.39 | -1.32, 0.54 | 0.411 | 0.980 |
| c47154 | sphingomyelin (d18:2/14:0, d18:1/14:1)* | -0.69 | -1.44, 0.06 | 0.072 | 0.770 | -0.31 | -1.09, 0.46 | 0.426 | 0.980 |
| c52433 | sphingomyelin (d17:1/16:0, d18:1/15:0, d16:1/17:0)* | -0.43 | -1.43, 0.56 | 0.393 | 0.919 | -0.38 | -1.35, 0.58 | 0.438 | 0.980 |
| c42459 | sphingomyelin (d18:2/16:0, d18:1/16:1)* | -0.41 | -1.70, 0.89 | 0.538 | 0.941 | -0.50 | -1.77, 0.77 | 0.443 | 0.980 |
| c52615 | sphingomyelin (d18:1/17:0, d17:1/18:0, d19:1/16:0) | 0.08 | -0.90, 1.07 | 0.871 | 0.967 | -0.21 | -1.18, 0.76 | 0.676 | 0.980 |
| c37529 | sphingomyelin (d18:1/18:1, d18:2/18:0) | 0.16 | -0.83, 1.15 | 0.753 | 0.951 | -0.04 | -1.02, 0.93 | 0.933 | 0.993 |
| c48490 | sphingomyelin (d18:1/20:0, d16:1/22:0)* | -0.24 | -1.14, 0.67 | 0.606 | 0.941 | -0.33 | -1.20, 0.55 | 0.462 | 0.980 |
| c48491 | sphingomyelin (d18:1/20:1, d18:2/20:0)* | 0.42 | -0.60, 1.44 | 0.420 | 0.920 | 0.21 | -0.79, 1.22 | 0.678 | 0.980 |
| c52495 | sphingomyelin (d18:1/21:0, d17:1/22:0, d16:1/23:0)* | -0.28 | -0.90, 0.35 | 0.383 | 0.919 | -0.19 | -0.80, 0.43 | 0.556 | 0.980 |
| c48493 | sphingomyelin (d18:1/22:1, d18:2/22:0, d16:1/24:1)* | -0.10 | -1.14, 0.95 | 0.854 | 0.967 | -0.22 | -1.25, 0.81 | 0.676 | 0.980 |
| c52435 | sphingomyelin (d18:2/23:0, d18:1/23:1, d17:1/24:1)* | -0.25 | -1.02, 0.53 | 0.531 | 0.941 | -0.26 | -1.03, 0.52 | 0.518 | 0.980 |
| c47153 | sphingomyelin (d18:1/24:1, d18:2/24:0)* | -0.33 | -1.67, 1.01 | 0.631 | 0.941 | -0.62 | -1.92, 0.69 | 0.356 | 0.973 |
| c34445 | sphingosine 1-phosphate | 0.37 | -0.42, 1.17 | 0.360 | 0.919 | 0.33 | -0.43, 1.10 | 0.394 | 0.980 |
|  | **Mevalonate Metabolism** |  |  |  |  |  |  |  |  |
| c531 | 3-hydroxy-3-methylglutarate | -0.24 | -0.85, 0.36 | 0.435 | 0.930 | -0.02 | -0.61, 0.58 | 0.957 | 0.993 |
|  | **Sterol** |  |  |  |  |  |  |  |  |
| c63 | cholesterol | -0.19 | -0.89, 0.51 | 0.595 | 0.941 | -0.30 | -0.98, 0.37 | 0.377 | 0.980 |
| c36776 | 7-HOCA | 0.27 | -0.44, 0.98 | 0.458 | 0.941 | 0.44 | -0.24, 1.13 | 0.209 | 0.940 |
| c36803 | 3beta,7alpha-dihydroxy-5-cholestenoate | -0.19 | -0.73, 0.35 | 0.489 | 0.941 | -0.33 | -0.85, 0.19 | 0.217 | 0.940 |
| c54805 | 3beta-hydroxy-5-cholestenoate | -0.05 | -0.68, 0.57 | 0.863 | 0.967 | -0.04 | -0.63, 0.56 | 0.906 | 0.993 |
|  | **Pregnenolone Steroids** |  |  |  |  |  |  |  |  |
| c38170 | pregnenolone sulfate | 0.03 | -0.28, 0.34 | 0.844 | 0.967 | -0.04 | -0.34, 0.27 | 0.808 | 0.993 |
| c46115 | 21-hydroxypregnenolone disulfate | -0.12 | -0.52, 0.28 | 0.557 | 0.941 | -0.10 | -0.49, 0.29 | 0.627 | 0.980 |
| c32619 | pregnenediol sulfate (C21H34O5S)* | 0.01 | -0.39, 0.40 | 0.971 | 0.991 | -0.02 | -0.40, 0.37 | 0.936 | 0.993 |
| c32562 | pregnen-diol disulfate* | -0.07 | -0.40, 0.25 | 0.660 | 0.941 | -0.06 | -0.38, 0.26 | 0.701 | 0.981 |
|  | **Progestin Steroids** |  |  |  |  |  |  |  |  |
| c37196 | 5alpha-pregnan-3beta,20beta-diol monosulfate (1) | -0.08 | -0.33, 0.18 | 0.562 | 0.941 | -0.13 | -0.39, 0.13 | 0.330 | 0.962 |
| c37200 | 5alpha-pregnan-3beta,20alpha-diol monosulfate (2) | 0.03 | -0.19, 0.25 | 0.803 | 0.951 | -0.04 | -0.26, 0.19 | 0.760 | 0.988 |
| c37198 | 5alpha-pregnan-3beta,20alpha-diol disulfate | 0.04 | -0.20, 0.29 | 0.725 | 0.949 | 0.01 | -0.24, 0.25 | 0.964 | 0.993 |
| c46172 | 5alpha-pregnan-diol disulfate | -0.04 | -0.23, 0.16 | 0.715 | 0.949 | -0.06 | -0.25, 0.14 | 0.562 | 0.980 |
| c40708 | pregnanediol-3-glucuronide | -0.07 | -0.31, 0.16 | 0.551 | 0.941 | -0.12 | -0.36, 0.11 | 0.314 | 0.953 |
|  | **Corticosteroids** |  |  |  |  |  |  |  |  |
| c1712 | cortisol | -0.09 | -0.53, 0.34 | 0.677 | 0.941 | -0.20 | -0.63, 0.22 | 0.352 | 0.971 |
| c1769 | cortisone | -0.06 | -0.73, 0.60 | 0.850 | 0.967 | -0.33 | -0.98, 0.31 | 0.311 | 0.949 |
|  | **Androgenic Steroids** |  |  |  |  |  |  |  |  |
| c32425 | dehydroepiandrosterone sulfate (DHEA-S) | -0.01 | -0.35, 0.34 | 0.977 | 0.991 | -0.08 | -0.42, 0.26 | 0.643 | 0.980 |
| c38168 | 16a-hydroxy DHEA 3-sulfate | 0.04 | -0.15, 0.24 | 0.651 | 0.941 | 0.06 | -0.13, 0.25 | 0.542 | 0.980 |
| c33973 | epiandrosterone sulfate | 0.02 | -0.22, 0.27 | 0.868 | 0.967 | -0.05 | -0.29, 0.19 | 0.660 | 0.980 |
| c31591 | androsterone sulfate | -0.02 | -0.25, 0.21 | 0.873 | 0.967 | -0.08 | -0.31, 0.14 | 0.472 | 0.980 |
| c47112 | etiocholanolone glucuronide | -0.09 | -0.30, 0.13 | 0.440 | 0.930 | -0.15 | -0.36, 0.06 | 0.163 | 0.940 |
| c37211 | androstenediol (3beta,17beta) monosulfate (1) | -0.02 | -0.31, 0.26 | 0.867 | 0.967 | -0.08 | -0.36, 0.20 | 0.590 | 0.980 |
| c37210 | androstenediol (3beta,17beta) monosulfate (2) | -0.24 | -0.51, 0.04 | 0.090 | 0.809 | -0.23 | -0.50, 0.04 | 0.095 | 0.940 |
| c37202 | androstenediol (3beta,17beta) disulfate (1) | -0.20 | -0.48, 0.08 | 0.169 | 0.844 | -0.20 | -0.48, 0.07 | 0.146 | 0.940 |
| c37203 | androstenediol (3beta,17beta) disulfate (2) | -0.23 | -0.60, 0.15 | 0.244 | 0.891 | -0.18 | -0.54, 0.19 | 0.343 | 0.968 |
| c37207 | androstenediol (3alpha, 17alpha) monosulfate (2) | -0.01 | -0.33, 0.32 | 0.968 | 0.991 | -0.01 | -0.32, 0.31 | 0.959 | 0.993 |
| c37209 | androstenediol (3alpha, 17alpha) monosulfate (3) | 0.10 | -0.21, 0.42 | 0.519 | 0.941 | 0.03 | -0.27, 0.34 | 0.835 | 0.993 |
| c37186 | 5alpha-androstan-3alpha,17beta-diol monosulfate (1) | 0.04 | -0.16, 0.25 | 0.690 | 0.941 | -0.02 | -0.22, 0.19 | 0.880 | 0.993 |
| c37190 | 5alpha-androstan-3beta,17beta-diol disulfate | -0.07 | -0.31, 0.17 | 0.556 | 0.941 | -0.11 | -0.34, 0.12 | 0.366 | 0.980 |
| c32827 | andro steroid monosulfate C19H28O6S (1)* | -0.03 | -0.26, 0.21 | 0.821 | 0.957 | 0.02 | -0.22, 0.25 | 0.890 | 0.993 |
|  | **Primary Bile Acid Metabolism** |  |  |  |  |  |  |  |  |
| c22842 | cholate | 0.07 | -0.10, 0.25 | 0.410 | 0.920 | 0.05 | -0.13, 0.22 | 0.606 | 0.980 |
| c18476 | glycocholate | 0.13 | -0.05, 0.32 | 0.157 | 0.844 | 0.09 | -0.10, 0.27 | 0.351 | 0.971 |
| c1563 | chenodeoxycholate | 0.03 | -0.15, 0.20 | 0.777 | 0.951 | -0.01 | -0.18, 0.17 | 0.934 | 0.993 |
| c32346 | glycochenodeoxycholate | 0.22 | 0.03, 0.41 | 0.024 | 0.628 | 0.19 | 0.01, 0.38 | 0.046 | 0.940 |
| c18494 | taurochenodeoxycholate | 0.06 | -0.11, 0.23 | 0.478 | 0.941 | 0 | -0.17, 0.17 | 0.965 | 0.993 |
| c52983 | glycochenodeoxycholate glucuronide (1) | 0.10 | -0.12, 0.31 | 0.381 | 0.919 | 0.06 | -0.15, 0.27 | 0.579 | 0.980 |
| c52974 | glycochenodeoxycholate 3-sulfate | -0.05 | -0.26, 0.17 | 0.675 | 0.941 | -0.05 | -0.26, 0.16 | 0.629 | 0.980 |
|  | **Secondary Bile Acid Metabolism** |  |  |  |  |  |  |  |  |
| c32620 | glycolithocholate sulfate* | 0.14 | -0.07, 0.35 | 0.199 | 0.844 | 0.16 | -0.04, 0.37 | 0.117 | 0.940 |
| c36850 | taurolithocholate 3-sulfate | 0.05 | -0.15, 0.24 | 0.637 | 0.941 | 0.01 | -0.17, 0.20 | 0.897 | 0.993 |
| c1605 | ursodeoxycholate | 0.15 | -0.01, 0.30 | 0.068 | 0.749 | 0.16 | 0.01, 0.32 | 0.037 | 0.940 |
| c39379 | glycoursodeoxycholate | 0.16 | -0.02, 0.33 | 0.075 | 0.776 | 0.16 | -0.01, 0.33 | 0.065 | 0.940 |
| c34093 | hyocholate | 0.06 | -0.19, 0.30 | 0.662 | 0.941 | 0.02 | -0.23, 0.27 | 0.886 | 0.993 |
| c42574 | glycohyocholate | 0.21 | -0.01, 0.43 | 0.061 | 0.749 | 0.19 | -0.04, 0.41 | 0.102 | 0.940 |
| c32599 | glycocholenate sulfate* | -0.10 | -0.60, 0.40 | 0.700 | 0.949 | 0 | -0.49, 0.49 | 0.992 | 0.999 |
| c32807 | taurocholenate sulfate* | -0.29 | -0.62, 0.03 | 0.079 | 0.776 | -0.30 | -0.62, 0.02 | 0.064 | 0.940 |
| c52975 | glycodeoxycholate 3-sulfate | 0.07 | -0.09, 0.24 | 0.372 | 0.919 | 0.12 | -0.04, 0.28 | 0.157 | 0.940 |
|  | **NUCLEOTIDE** |  |  |  |  |  |  |  |  |
|  | **Purine Metabolism, (Hypo)Xanthine/Inosine containing** | |  |  |  |  |  |  |  |
| c1123 | inosine | -0.07 | -0.20, 0.07 | 0.329 | 0.919 | -0.08 | -0.21, 0.04 | 0.207 | 0.940 |
| c3127 | hypoxanthine | 0.24 | -0.16, 0.64 | 0.237 | 0.871 | 0.13 | -0.26, 0.52 | 0.507 | 0.980 |
| c3147 | xanthine | 0.16 | -0.53, 0.85 | 0.651 | 0.941 | 0.05 | -0.63, 0.72 | 0.896 | 0.993 |
| c48351 | N1-methylinosine | 0.51 | -0.25, 1.28 | 0.190 | 0.844 | 0.76 | 0.01, 1.51 | 0.048 | 0.940 |
| c1604 | urate | -0.26 | -1.05, 0.53 | 0.520 | 0.941 | 0.02 | -0.76, 0.79 | 0.962 | 0.993 |
| c1107 | allantoin | -0.04 | -0.46, 0.39 | 0.869 | 0.967 | -0.05 | -0.46, 0.35 | 0.796 | 0.993 |
|  | **Purine Metabolism, Adenine containing** |  |  |  |  |  |  |  |  |
| c32342 | AMP | 0.12 | -0.80, 1.03 | 0.801 | 0.951 | 0.15 | -0.74, 1.05 | 0.736 | 0.988 |
| c554 | adenine | 0.08 | -0.53, 0.68 | 0.807 | 0.951 | 0.21 | -0.38, 0.79 | 0.488 | 0.980 |
| c15650 | 1-methyladenosine | 0.08 | -0.42, 0.58 | 0.750 | 0.951 | 0.10 | -0.38, 0.59 | 0.675 | 0.980 |
| c37114 | N6-methyladenosine | -0.07 | -0.44, 0.31 | 0.729 | 0.949 | -0.08 | -0.43, 0.27 | 0.654 | 0.980 |
| c35157 | N6-carbamoylthreonyladenosine | -0.10 | -1.15, 0.95 | 0.846 | 0.967 | 0.34 | -0.68, 1.37 | 0.512 | 0.980 |
|  | **Purine Metabolism, Guanine containing** |  |  |  |  |  |  |  |  |
| c35114 | 7-methylguanine | -0.67 | -1.60, 0.26 | 0.159 | 0.844 | -0.35 | -1.27, 0.57 | 0.454 | 0.980 |
| c35137 | N2,N2-dimethylguanosine | -0.21 | -1.11, 0.69 | 0.648 | 0.941 | 0.14 | -0.76, 1.03 | 0.766 | 0.990 |
|  | **Pyrimidine Metabolism, Orotate containing** |  |  |  |  |  |  |  |  |
| c1505 | orotate | -0.11 | -0.64, 0.42 | 0.688 | 0.941 | 0.05 | -0.48, 0.57 | 0.863 | 0.993 |
| c35172 | orotidine | -0.03 | -0.72, 0.66 | 0.925 | 0.983 | 0.33 | -0.35, 1.01 | 0.343 | 0.968 |
|  | **Pyrimidine Metabolism, Uracil containing** |  |  |  |  |  |  |  |  |
| c606 | uridine | -0.48 | -1.35, 0.38 | 0.273 | 0.895 | -0.60 | -1.44, 0.25 | 0.169 | 0.940 |
| c33442 | pseudouridine | -0.44 | -1.56, 0.69 | 0.451 | 0.936 | -0.29 | -1.39, 0.82 | 0.610 | 0.980 |
| c35136 | 5-methyluridine (ribothymidine) | 0.39 | -0.50, 1.29 | 0.389 | 0.919 | 0.34 | -0.53, 1.22 | 0.445 | 0.980 |
| c3155 | 3-ureidopropionate | 0.07 | -0.46, 0.61 | 0.786 | 0.951 | -0.12 | -0.65, 0.42 | 0.665 | 0.980 |
| c55 | beta-alanine | 0.10 | -0.54, 0.75 | 0.758 | 0.951 | 0.29 | -0.34, 0.92 | 0.365 | 0.980 |
| c37432 | N-acetyl-beta-alanine | -0.66 | -1.34, 0.02 | 0.061 | 0.749 | -0.40 | -1.08, 0.27 | 0.243 | 0.940 |
|  | **Pyrimidine Metabolism, Cytidine containing** |  |  |  |  |  |  |  |  |
| c514 | cytidine | -0.25 | -0.59, 0.09 | 0.157 | 0.844 | -0.16 | -0.50, 0.17 | 0.343 | 0.968 |
|  | **Pyrimidine Metabolism, Thymine containing** |  |  |  |  |  |  |  |  |
| c1418 | 5,6-dihydrothymine | 0.11 | -0.73, 0.95 | 0.803 | 0.951 | 0.18 | -0.66, 1.01 | 0.676 | 0.980 |
| c1566 | 3-aminoisobutyrate | -0.13 | -0.52, 0.26 | 0.522 | 0.941 | -0.08 | -0.46, 0.31 | 0.700 | 0.981 |
|  | **COFACTORS** |  |  |  |  |  |  |  |  |
|  | **Nicotinate and Nicotinamide Metabolism** |  |  |  |  |  |  |  |  |
| c1899 | quinolinate | -0.25 | -0.78, 0.29 | 0.366 | 0.919 | -0.11 | -0.65, 0.42 | 0.674 | 0.980 |
| c594 | nicotinamide | 0.59 | 0.19, 1.00 | 0.005 | 0.577 | 0.51 | 0.11, 0.91 | 0.013 | 0.935 |
| c27665 | 1-methylnicotinamide | 0.27 | -0.01, 0.55 | 0.057 | 0.749 | 0.20 | -0.07, 0.47 | 0.145 | 0.940 |
| c32401 | trigonelline (N'-methylnicotinate) | 0.03 | -0.23, 0.29 | 0.815 | 0.952 | -0.03 | -0.29, 0.22 | 0.789 | 0.993 |
| c40469 | N1-Methyl-2-pyridone-5-carboxamide | -0.11 | -0.56, 0.33 | 0.622 | 0.941 | -0.04 | -0.48, 0.40 | 0.857 | 0.993 |
|  | **Pantothenate and CoA Metabolism** |  |  |  |  |  |  |  |  |
| c1508 | pantothenate (Vitamin B5) | -0.76 | -1.48,-0.05 | 0.039 | 0.628 | -0.53 | -1.25, 0.18 | 0.146 | 0.940 |
|  | **Ascorbate and Aldarate Metabolism** |  |  |  |  |  |  |  |  |
| c27738 | threonate | 0.02 | -0.32, 0.35 | 0.925 | 0.983 | 0.05 | -0.28, 0.38 | 0.770 | 0.992 |
| c20694 | oxalate (ethanedioate) | -0.10 | -0.51, 0.32 | 0.652 | 0.941 | -0.10 | -0.52, 0.32 | 0.639 | 0.980 |
| c46957 | gulonate* | -0.76 | -1.25,-0.27 | 0.003 | 0.577 | -0.62 | -1.10,-0.13 | 0.014 | 0.935 |
|  | **Tocopherol Metabolism** |  |  |  |  |  |  |  |  |
| c1561 | alpha-tocopherol | 0.08 | -0.22, 0.39 | 0.598 | 0.941 | 0.20 | -0.06, 0.47 | 0.132 | 0.940 |
| c44876 | gamma-CEHC | 0.12 | -0.19, 0.43 | 0.451 | 0.936 | 0.29 | 0.00, 0.58 | 0.055 | 0.940 |
|  | **Hemoglobin and Porphyrin Metabolism** |  |  |  |  |  |  |  |  |
| c43807 | bilirubin | 0.04 | -0.14, 0.21 | 0.685 | 0.941 | 0.08 | -0.07, 0.24 | 0.294 | 0.940 |
| c32586 | bilirubin (E,E)* | 0.04 | -0.14, 0.22 | 0.678 | 0.941 | 0.09 | -0.07, 0.26 | 0.261 | 0.940 |
| c47886 | bilirubin (E,Z or Z,E)* | -0.06 | -0.28, 0.15 | 0.576 | 0.941 | -0.02 | -0.23, 0.19 | 0.851 | 0.993 |
| c2137 | biliverdin | -0.10 | -0.33, 0.14 | 0.422 | 0.920 | -0.05 | -0.28, 0.18 | 0.652 | 0.980 |
|  | **Vitamin A Metabolism** |  |  |  |  |  |  |  |  |
| c1806 | retinol (Vitamin A) | -0.33 | -1.01, 0.35 | 0.340 | 0.919 | -0.06 | -0.71, 0.60 | 0.860 | 0.993 |
|  | **Vitamin B6 Metabolism** |  |  |  |  |  |  |  |  |
| c31555 | pyridoxate | -0.04 | -0.51, 0.42 | 0.858 | 0.967 | 0.04 | -0.43, 0.50 | 0.877 | 0.993 |
|  | **XENOBIOTICS** |  |  |  |  |  |  |  |  |
|  | **Benzoate Metabolism** |  |  |  |  |  |  |  |  |
| c15753 | hippurate | 0.14 | -0.11, 0.39 | 0.270 | 0.895 | 0.17 | -0.08, 0.42 | 0.190 | 0.940 |
| c39600 | 3-hydroxyhippurate | 0.05 | -0.17, 0.26 | 0.663 | 0.941 | 0.04 | -0.17, 0.24 | 0.737 | 0.988 |
| c35527 | 4-hydroxyhippurate | 0.21 | -0.13, 0.54 | 0.230 | 0.870 | 0.17 | -0.16, 0.50 | 0.322 | 0.962 |
| c15778 | benzoate | -0.29 | -0.79, 0.21 | 0.255 | 0.894 | -0.41 | -0.90, 0.09 | 0.107 | 0.940 |
| c35320 | catechol sulfate | 0.31 | 0.00, 0.63 | 0.055 | 0.744 | 0.39 | 0.08, 0.70 | 0.016 | 0.935 |
| c46111 | guaiacol sulfate | 0.19 | -0.10, 0.49 | 0.204 | 0.844 | 0.31 | 0.02, 0.60 | 0.041 | 0.940 |
| c46165 | 3-methyl catechol sulfate (1) | 0.07 | -0.09, 0.24 | 0.394 | 0.919 | 0.13 | -0.04, 0.29 | 0.129 | 0.940 |
| c46146 | 4-methylcatechol sulfate | 0.04 | -0.24, 0.32 | 0.786 | 0.951 | 0.04 | -0.23, 0.32 | 0.749 | 0.988 |
| c36099 | 4-ethylphenyl sulfate | 0.09 | -0.10, 0.28 | 0.369 | 0.919 | 0.16 | -0.03, 0.34 | 0.106 | 0.940 |
| c36098 | 4-vinylphenol sulfate | -0.03 | -0.19, 0.13 | 0.727 | 0.949 | 0.01 | -0.15, 0.17 | 0.889 | 0.993 |
| c48763 | 3-methoxycatechol sulfate (1) | 0.12 | -0.05, 0.29 | 0.167 | 0.844 | 0.13 | -0.03, 0.30 | 0.116 | 0.940 |
| c48429 | methyl-4-hydroxybenzoate sulfate | 0.01 | -0.11, 0.12 | 0.912 | 0.982 | -0.05 | -0.16, 0.07 | 0.456 | 0.980 |
| c36103 | p-cresol sulfate | 0.08 | -0.24, 0.41 | 0.618 | 0.941 | 0 | -0.32, 0.32 | 0.999 | 0.999 |
| c35635 | 3-(3-hydroxyphenyl)propionate | 0.16 | -0.08, 0.40 | 0.190 | 0.844 | 0.14 | -0.10, 0.37 | 0.254 | 0.940 |
| c15749 | 3-phenylpropionate (hydrocinnamate) | 0.07 | -0.14, 0.29 | 0.507 | 0.941 | 0.06 | -0.15, 0.28 | 0.549 | 0.980 |
|  | **Xanthine Metabolism** |  |  |  |  |  |  |  |  |
| c569 | caffeine | -0.01 | -0.14, 0.12 | 0.896 | 0.976 | 0.04 | -0.09, 0.16 | 0.584 | 0.980 |
| c18392 | theobromine | 0 | -0.14, 0.14 | 0.946 | 0.987 | 0.06 | -0.08, 0.20 | 0.379 | 0.980 |
| c32445 | 3-methylxanthine | 0.02 | -0.13, 0.17 | 0.798 | 0.951 | 0.10 | -0.05, 0.25 | 0.187 | 0.940 |
| c34390 | 7-methylxanthine | -0.01 | -0.16, 0.13 | 0.880 | 0.967 | 0.05 | -0.10, 0.19 | 0.514 | 0.980 |
| c34424 | 5-acetylamino-6-amino-3-methyluracil | -0.03 | -0.16, 0.10 | 0.679 | 0.941 | 0.01 | -0.12, 0.14 | 0.829 | 0.993 |
|  | **Food Component/Plant** |  |  |  |  |  |  |  |  |
| c43400 | 2-piperidinone | 0.06 | -0.16, 0.29 | 0.579 | 0.941 | 0.10 | -0.12, 0.32 | 0.385 | 0.980 |
| c38276 | 2,3-dihydroxyisovalerate | -0.14 | -0.32, 0.04 | 0.134 | 0.844 | -0.16 | -0.33, 0.02 | 0.077 | 0.940 |
| c587 | gluconate | -0.34 | -0.89, 0.21 | 0.224 | 0.870 | -0.25 | -0.78, 0.29 | 0.370 | 0.980 |
| c38637 | cinnamoylglycine | 0 | -0.17, 0.16 | 0.977 | 0.991 | 0.01 | -0.15, 0.18 | 0.864 | 0.993 |
| c37459 | ergothioneine | 0.40 | 0.09, 0.71 | 0.013 | 0.577 | 0.37 | 0.07, 0.67 | 0.016 | 0.935 |
| c20699 | erythritol | -0.66 | -1.56, 0.23 | 0.148 | 0.844 | -0.22 | -1.12, 0.69 | 0.640 | 0.980 |
| c33009 | homostachydrine* | -0.42 | -0.77,-0.06 | 0.023 | 0.628 | -0.38 | -0.72,-0.04 | 0.033 | 0.940 |
| c43374 | indolin-2-one | 0.36 | 0.03, 0.69 | 0.033 | 0.628 | 0.36 | 0.04, 0.68 | 0.027 | 0.940 |
| c33935 | piperine | 0.02 | -0.08, 0.13 | 0.671 | 0.941 | -0.01 | -0.11, 0.10 | 0.927 | 0.993 |
| c43239 | S-allylcysteine | 0.12 | -0.01, 0.25 | 0.082 | 0.776 | 0.11 | -0.02, 0.25 | 0.092 | 0.940 |
| c15336 | tartarate | -0.13 | -0.35, 0.09 | 0.235 | 0.871 | -0.17 | -0.38, 0.04 | 0.119 | 0.940 |
| c37181 | 4-allylphenol sulfate | 0.11 | -0.06, 0.29 | 0.206 | 0.844 | 0.10 | -0.07, 0.28 | 0.233 | 0.940 |
| c20693 | tartronate (hydroxymalonate) | 0.15 | -0.19, 0.50 | 0.385 | 0.919 | 0.04 | -0.30, 0.38 | 0.833 | 0.993 |
|  | **Drug - Topical Agents** |  |  |  |  |  |  |  |  |
| c1515 | salicylate | 0.08 | -0.14, 0.31 | 0.483 | 0.941 | 0 | -0.22, 0.22 | 0.996 | 0.999 |
|  | **Chemical** |  |  |  |  |  |  |  |  |
| c46960 | sulfate* | 0.19 | -0.93, 1.31 | 0.739 | 0.949 | -0.03 | -1.14, 1.08 | 0.959 | 0.993 |
| c45413 | O-sulfo-L-tyrosine | -0.40 | -1.27, 0.47 | 0.371 | 0.919 | 0.04 | -0.84, 0.91 | 0.934 | 0.993 |
| c43266 | 2-aminophenol sulfate | 0.11 | -0.08, 0.31 | 0.260 | 0.895 | 0.08 | -0.12, 0.28 | 0.444 | 0.980 |
| c48441 | 4-hydroxychlorothalonil | 0.04 | -0.42, 0.50 | 0.870 | 0.967 | 0.01 | -0.43, 0.45 | 0.971 | 0.993 |
| c48448 | 3-hydroxypyridine sulfate | 0.19 | -0.04, 0.42 | 0.115 | 0.844 | 0.16 | -0.06, 0.39 | 0.157 | 0.940 |
| c48698 | 6-hydroxyindole sulfate | 0.31 | -0.11, 0.74 | 0.153 | 0.844 | 0.25 | -0.16, 0.67 | 0.230 | 0.940 |
| c53231 | thioproline | 0.19 | -0.04, 0.42 | 0.120 | 0.844 | 0.19 | -0.04, 0.41 | 0.123 | 0.940 |
|  | **UNKNOWN** |  |  |  |  |  |  |  |  |
| c32578 | X - 11261 | -0.12 | -0.47, 0.23 | 0.493 | 0.941 | -0.06 | -0.40, 0.28 | 0.745 | 0.988 |
| c33132 | X - 11787 | -0.72 | -1.76, 0.31 | 0.174 | 0.844 | -0.53 | -1.58, 0.52 | 0.324 | 0.962 |
| c33140 | X - 11795 | -0.15 | -0.60, 0.29 | 0.493 | 0.941 | -0.14 | -0.56, 0.29 | 0.535 | 0.980 |
| c46259 | X - 21258 | 0.14 | -0.06, 0.33 | 0.168 | 0.844 | 0.16 | -0.03, 0.34 | 0.102 | 0.940 |
| c46266 | X - 15486 | -0.10 | -0.33, 0.14 | 0.430 | 0.922 | -0.10 | -0.33, 0.13 | 0.390 | 0.980 |
| c46283 | X - 15461 | -0.70 | -1.22,-0.17 | 0.010 | 0.577 | -0.80 | -1.30,-0.30 | 0.002 | 0.613 |
| c46295 | X - 21286 | 0.14 | -0.21, 0.49 | 0.438 | 0.930 | 0.14 | -0.21, 0.49 | 0.427 | 0.980 |
| c46347 | X - 11381 | 0.35 | -0.09, 0.79 | 0.117 | 0.844 | 0.37 | -0.05, 0.80 | 0.089 | 0.940 |
| c46354 | X - 21310 | 0.40 | -0.17, 0.97 | 0.169 | 0.844 | 0.39 | -0.15, 0.94 | 0.161 | 0.940 |
| c46356 | X - 21312 | 0.03 | -0.16, 0.22 | 0.788 | 0.951 | 0.10 | -0.09, 0.29 | 0.306 | 0.940 |
| c46363 | X - 21319 | -0.17 | -0.45, 0.11 | 0.224 | 0.870 | -0.15 | -0.42, 0.12 | 0.266 | 0.940 |
| c46364 | X - 12847 | 0.05 | -0.10, 0.19 | 0.541 | 0.941 | 0.04 | -0.10, 0.18 | 0.552 | 0.980 |
| c46384 | X - 21339 | 0.17 | -0.24, 0.58 | 0.407 | 0.919 | 0.10 | -0.30, 0.50 | 0.627 | 0.980 |
| c46390 | X - 11308 | 0.36 | -0.12, 0.85 | 0.141 | 0.844 | 0.28 | -0.20, 0.76 | 0.259 | 0.940 |
| c46398 | X - 21353 | -0.30 | -0.63, 0.03 | 0.078 | 0.776 | -0.28 | -0.61, 0.05 | 0.102 | 0.940 |
| c46409 | X - 21364 | -0.12 | -0.59, 0.35 | 0.607 | 0.941 | -0.06 | -0.52, 0.40 | 0.799 | 0.993 |
| c46417 | X - 13866 | 0.21 | -0.11, 0.53 | 0.200 | 0.844 | 0.19 | -0.12, 0.50 | 0.235 | 0.940 |
| c46428 | X - 21383 | -0.13 | -0.37, 0.11 | 0.289 | 0.898 | -0.13 | -0.37, 0.12 | 0.306 | 0.940 |
| c46460 | X - 11444 | -0.21 | -0.79, 0.36 | 0.470 | 0.941 | -0.13 | -0.71, 0.45 | 0.657 | 0.980 |
| c46466 | X - 11843 | 0.02 | -0.09, 0.13 | 0.690 | 0.941 | 0.02 | -0.09, 0.13 | 0.713 | 0.984 |
| c46486 | X - 21441 | -0.13 | -0.40, 0.15 | 0.365 | 0.919 | -0.13 | -0.40, 0.13 | 0.327 | 0.962 |
| c46507 | X - 11850 | 0.01 | -0.11, 0.13 | 0.889 | 0.973 | 0.01 | -0.10, 0.13 | 0.831 | 0.993 |
| c46510 | X - 12544 | 0.14 | -0.11, 0.39 | 0.270 | 0.895 | 0.18 | -0.06, 0.42 | 0.138 | 0.940 |
| c46512 | X - 21467 | -0.11 | -0.44, 0.21 | 0.503 | 0.941 | 0 | -0.33, 0.32 | 0.979 | 0.996 |
| c46515 | X - 21470 | -0.07 | -0.30, 0.17 | 0.581 | 0.941 | -0.05 | -0.29, 0.18 | 0.642 | 0.980 |
| c46516 | X - 21471 | -0.10 | -0.37, 0.17 | 0.482 | 0.941 | -0.03 | -0.29, 0.24 | 0.851 | 0.993 |
| c46517 | X - 16946 | -0.18 | -0.48, 0.12 | 0.252 | 0.892 | -0.15 | -0.44, 0.14 | 0.305 | 0.940 |
| c46521 | X - 11852 | 0.09 | -0.02, 0.21 | 0.121 | 0.844 | 0.09 | -0.02, 0.20 | 0.108 | 0.940 |
| c46590 | X - 07765 | 0.04 | -0.15, 0.23 | 0.670 | 0.941 | 0.04 | -0.14, 0.22 | 0.657 | 0.980 |
| c46592 | X - 11299 | 0.01 | -0.11, 0.13 | 0.831 | 0.963 | 0 | -0.12, 0.11 | 0.986 | 0.999 |
| c46594 | X - 11372 | 0.65 | 0.06, 1.25 | 0.034 | 0.628 | 0.62 | 0.04, 1.21 | 0.039 | 0.940 |
| c46601 | X - 11470 | -0.19 | -0.59, 0.21 | 0.357 | 0.919 | -0.07 | -0.48, 0.35 | 0.758 | 0.988 |
| c46602 | X - 11478 | -0.10 | -0.41, 0.21 | 0.518 | 0.941 | -0.10 | -0.40, 0.20 | 0.511 | 0.980 |
| c46607 | X - 11849 | -0.02 | -0.14, 0.10 | 0.759 | 0.951 | -0.02 | -0.13, 0.10 | 0.785 | 0.993 |
| c46608 | X - 11880 | 0.38 | -0.15, 0.92 | 0.162 | 0.844 | 0.33 | -0.20, 0.86 | 0.223 | 0.940 |
| c46613 | X - 12216 | 0.10 | -0.10, 0.29 | 0.341 | 0.919 | 0.08 | -0.11, 0.27 | 0.425 | 0.980 |
| c46616 | X - 12411 | 0.12 | -0.12, 0.36 | 0.327 | 0.919 | 0.21 | -0.03, 0.44 | 0.083 | 0.940 |
| c46623 | X - 12729 | -0.09 | -0.35, 0.17 | 0.484 | 0.941 | -0.21 | -0.47, 0.05 | 0.113 | 0.940 |
| c46624 | X - 12798 | -0.11 | -0.57, 0.36 | 0.660 | 0.941 | -0.03 | -0.50, 0.43 | 0.885 | 0.993 |
| c46632 | X - 14056 | 0.03 | -0.21, 0.28 | 0.791 | 0.951 | 0.07 | -0.17, 0.31 | 0.565 | 0.980 |
| c46633 | X - 12844 | -0.72 | -1.36,-0.08 | 0.030 | 0.628 | -0.48 | -1.12, 0.16 | 0.143 | 0.940 |
| c46636 | X - 12849 | 0.02 | -0.12, 0.17 | 0.735 | 0.949 | -0.04 | -0.18, 0.10 | 0.571 | 0.980 |
| c46640 | X - 15469 | -0.23 | -0.63, 0.16 | 0.248 | 0.891 | -0.19 | -0.58, 0.21 | 0.355 | 0.973 |
| c46645 | X - 13728 | 0.02 | -0.13, 0.17 | 0.784 | 0.951 | 0.10 | -0.04, 0.25 | 0.174 | 0.940 |
| c46646 | X - 13835 | -0.03 | -0.21, 0.15 | 0.734 | 0.949 | 0.03 | -0.15, 0.21 | 0.742 | 0.988 |
| c46657 | X - 14939 | -0.13 | -0.50, 0.24 | 0.486 | 0.941 | -0.22 | -0.58, 0.13 | 0.222 | 0.940 |
| c46661 | X - 15245 | 0 | -0.15, 0.16 | 0.966 | 0.991 | 0.06 | -0.08, 0.20 | 0.426 | 0.980 |
| c46662 | X - 15492 | -0.40 | -0.73,-0.07 | 0.019 | 0.628 | -0.31 | -0.64, 0.01 | 0.061 | 0.940 |
| c46666 | X - 15728 | 0.22 | 0.06, 0.37 | 0.006 | 0.577 | 0.24 | 0.09, 0.38 | 0.002 | 0.613 |
| c46673 | X - 16576 | 0.45 | 0.12, 0.77 | 0.008 | 0.577 | 0.39 | 0.08, 0.71 | 0.016 | 0.935 |
| c46674 | X - 21607 | -0.05 | -0.25, 0.15 | 0.608 | 0.941 | -0.09 | -0.28, 0.10 | 0.365 | 0.980 |
| c46681 | X - 16935 | 0.08 | -0.21, 0.37 | 0.588 | 0.941 | 0.03 | -0.26, 0.31 | 0.860 | 0.993 |
| c46683 | X - 16944 | -0.14 | -0.46, 0.18 | 0.388 | 0.919 | -0.13 | -0.45, 0.18 | 0.407 | 0.980 |
| c46685 | X - 16964 | 0.05 | -0.32, 0.42 | 0.794 | 0.951 | 0.19 | -0.13, 0.51 | 0.261 | 0.940 |
| c46690 | X - 18901 | 0.04 | -0.24, 0.33 | 0.761 | 0.951 | 0.08 | -0.20, 0.36 | 0.565 | 0.980 |
| c46695 | X - 18913 | -0.10 | -0.64, 0.43 | 0.705 | 0.949 | -0.06 | -0.58, 0.46 | 0.829 | 0.993 |
| c46700 | X - 18922 | 0.07 | -0.23, 0.36 | 0.658 | 0.941 | 0.05 | -0.25, 0.34 | 0.748 | 0.988 |
| c46701 | X - 19141 | -0.15 | -0.51, 0.20 | 0.395 | 0.919 | -0.08 | -0.43, 0.26 | 0.643 | 0.980 |
| c46710 | X - 17690 | 0.07 | -0.07, 0.21 | 0.335 | 0.919 | 0.09 | -0.05, 0.23 | 0.226 | 0.940 |
| c46902 | X - 21733 | 0.17 | -0.07, 0.42 | 0.163 | 0.844 | 0.12 | -0.12, 0.36 | 0.316 | 0.954 |
| c46905 | X - 21736 | -0.05 | -0.37, 0.27 | 0.766 | 0.951 | 0.03 | -0.29, 0.34 | 0.873 | 0.993 |
| c46909 | X - 21740 | -0.15 | -0.53, 0.22 | 0.429 | 0.922 | -0.08 | -0.44, 0.29 | 0.689 | 0.981 |
| c46932 | X - 12104 | 0.51 | 0.03, 1.00 | 0.041 | 0.628 | 0.50 | 0.03, 0.97 | 0.039 | 0.940 |
| c46972 | X - 21796 | 0.13 | -0.37, 0.62 | 0.621 | 0.941 | 0.11 | -0.37, 0.60 | 0.648 | 0.980 |
| c46977 | X - 15503 | -0.10 | -0.68, 0.49 | 0.748 | 0.951 | 0.02 | -0.54, 0.59 | 0.940 | 0.993 |
| c46997 | X - 12822 | 0.09 | -0.27, 0.46 | 0.614 | 0.941 | 0.14 | -0.22, 0.50 | 0.440 | 0.980 |
| c47006 | X - 21829 | 0.03 | -0.14, 0.20 | 0.740 | 0.949 | 0.02 | -0.15, 0.19 | 0.824 | 0.993 |
| c47013 | X - 16570 | -0.06 | -0.36, 0.23 | 0.672 | 0.941 | 0.03 | -0.27, 0.32 | 0.863 | 0.993 |
| c47301 | X - 18887 | 0.01 | -0.39, 0.40 | 0.978 | 0.991 | 0.02 | -0.38, 0.42 | 0.923 | 0.993 |
| c47417 | X - 22162 | 0.12 | -0.56, 0.80 | 0.731 | 0.949 | 0.29 | -0.41, 0.99 | 0.417 | 0.980 |
| c47439 | X - 13507 | -0.34 | -0.80, 0.11 | 0.140 | 0.844 | -0.38 | -0.82, 0.07 | 0.099 | 0.940 |
| c47642 | X - 12101 | -0.04 | -0.27, 0.18 | 0.721 | 0.949 | -0.08 | -0.29, 0.13 | 0.471 | 0.980 |
| c47664 | X - 13658 | 0.11 | -0.13, 0.35 | 0.374 | 0.919 | 0.09 | -0.15, 0.32 | 0.464 | 0.980 |
| c47670 | X - 18899 | 0.32 | -0.02, 0.65 | 0.067 | 0.749 | 0.35 | 0.01, 0.68 | 0.043 | 0.940 |
| c47671 | X - 18921 | -0.13 | -0.44, 0.19 | 0.428 | 0.922 | -0.17 | -0.48, 0.14 | 0.284 | 0.940 |
| c47673 | X - 19299 | 0.03 | -0.12, 0.18 | 0.702 | 0.949 | 0.04 | -0.11, 0.18 | 0.621 | 0.980 |
| c47687 | X - 12100 | -0.29 | -0.81, 0.24 | 0.295 | 0.899 | -0.38 | -0.85, 0.10 | 0.135 | 0.940 |
| c47708 | X - 12283 | -0.10 | -0.34, 0.14 | 0.401 | 0.919 | -0.16 | -0.39, 0.07 | 0.177 | 0.940 |
| c47783 | X - 22519 | -0.27 | -0.70, 0.17 | 0.227 | 0.870 | -0.28 | -0.70, 0.15 | 0.202 | 0.940 |
| c47802 | X - 16397 | 0.05 | -0.22, 0.31 | 0.732 | 0.949 | 0 | -0.26, 0.26 | 0.999 | 0.999 |
| c47804 | X - 16580 | -0.05 | -0.41, 0.30 | 0.771 | 0.951 | -0.08 | -0.42, 0.27 | 0.660 | 0.980 |
| c47872 | X - 17340 | -0.45 | -0.80,-0.10 | 0.013 | 0.577 | -0.31 | -0.66, 0.03 | 0.080 | 0.940 |
| c47905 | X - 12026 | -0.29 | -0.88, 0.30 | 0.342 | 0.919 | -0.11 | -0.69, 0.48 | 0.715 | 0.984 |
| c47929 | X - 12707 | 0 | -0.35, 0.36 | 0.985 | 0.991 | 0.03 | -0.32, 0.37 | 0.885 | 0.993 |
| c47959 | X - 13553 | 0.08 | -0.37, 0.53 | 0.720 | 0.949 | 0.16 | -0.27, 0.59 | 0.473 | 0.980 |
| c48001 | X - 17351 | -0.10 | -0.35, 0.15 | 0.422 | 0.920 | -0.14 | -0.39, 0.10 | 0.254 | 0.940 |
| c48047 | X - 18886 | 0.08 | -0.35, 0.50 | 0.721 | 0.949 | 0.11 | -0.30, 0.52 | 0.601 | 0.980 |
| c48076 | X - 22771 | 0.03 | -0.31, 0.38 | 0.864 | 0.967 | 0.07 | -0.26, 0.39 | 0.688 | 0.981 |
| c49463 | X - 23587 | 0.05 | -0.18, 0.28 | 0.663 | 0.941 | 0.02 | -0.20, 0.24 | 0.851 | 0.993 |
| c49466 | X - 23590 | -0.34 | -0.87, 0.20 | 0.218 | 0.870 | -0.02 | -0.55, 0.50 | 0.933 | 0.993 |
| c49469 | X - 23593 | -0.51 | -1.04, 0.03 | 0.067 | 0.749 | -0.50 | -1.02, 0.02 | 0.059 | 0.940 |
| c49515 | X - 23639 | -0.06 | -0.69, 0.57 | 0.854 | 0.967 | 0.07 | -0.54, 0.68 | 0.823 | 0.993 |
| c49517 | X - 23641 | -0.08 | -0.36, 0.21 | 0.585 | 0.941 | 0.01 | -0.27, 0.30 | 0.931 | 0.993 |
| c49521 | X - 23644 | 0.02 | -0.13, 0.17 | 0.806 | 0.951 | 0.04 | -0.10, 0.18 | 0.600 | 0.980 |
| c49536 | X - 23659 | -0.26 | -0.54, 0.01 | 0.063 | 0.749 | -0.27 | -0.54, 0.00 | 0.048 | 0.940 |
| c49557 | X - 23680 | -0.23 | -0.56, 0.11 | 0.183 | 0.844 | -0.19 | -0.51, 0.14 | 0.267 | 0.940 |
| c49592 | X - 11315 | -0.06 | -0.66, 0.54 | 0.833 | 0.963 | -0.31 | -0.90, 0.27 | 0.299 | 0.940 |
| c49637 | X - 23739 | 0.27 | -0.11, 0.65 | 0.161 | 0.844 | 0.17 | -0.22, 0.55 | 0.396 | 0.980 |
| c49679 | X - 23780 | -0.02 | -0.30, 0.25 | 0.881 | 0.967 | -0.06 | -0.33, 0.20 | 0.637 | 0.980 |
| c49681 | X - 23782 | -0.30 | -0.74, 0.14 | 0.185 | 0.844 | -0.22 | -0.64, 0.20 | 0.301 | 0.940 |
| c49883 | X - 23974 | 0.09 | -0.27, 0.44 | 0.628 | 0.941 | -0.03 | -0.38, 0.31 | 0.850 | 0.993 |
| c52483 | X - 24295 | 0.01 | -0.12, 0.13 | 0.907 | 0.980 | 0 | -0.12, 0.12 | 0.971 | 0.993 |
| c52524 | X - 24328 | 0.01 | -0.34, 0.36 | 0.945 | 0.987 | 0.01 | -0.33, 0.35 | 0.972 | 0.993 |
| c52533 | X - 24337 | 0.15 | -0.18, 0.49 | 0.370 | 0.919 | 0.21 | -0.10, 0.53 | 0.187 | 0.940 |
| c52636 | X - 24422 | 0.10 | -0.36, 0.56 | 0.669 | 0.941 | 0.24 | -0.21, 0.69 | 0.301 | 0.940 |
| c52665 | X - 24435 | -0.32 | -1.00, 0.36 | 0.355 | 0.919 | -0.42 | -1.08, 0.24 | 0.211 | 0.940 |
| c52772 | X - 24455 | 0.01 | -0.49, 0.51 | 0.982 | 0.991 | -0.12 | -0.58, 0.35 | 0.631 | 0.980 |
| c52773 | X - 24456 | 0.01 | -0.32, 0.34 | 0.944 | 0.987 | -0.03 | -0.35, 0.28 | 0.830 | 0.993 |
| c52865 | X - 24544 | -0.18 | -0.46, 0.11 | 0.224 | 0.870 | -0.15 | -0.42, 0.13 | 0.289 | 0.940 |
| c52867 | X - 24546 | 0 | -0.27, 0.27 | 0.987 | 0.991 | -0.04 | -0.31, 0.23 | 0.748 | 0.988 |
| c52877 | X - 24556 | -0.22 | -0.56, 0.12 | 0.201 | 0.844 | -0.11 | -0.44, 0.23 | 0.533 | 0.980 |
| c52909 | X - 24588 | -0.46 | -0.89,-0.02 | 0.042 | 0.628 | -0.32 | -0.76, 0.12 | 0.152 | 0.940 |
| c53127 | X - 24699 | -0.67 | -1.57, 0.22 | 0.139 | 0.844 | -0.55 | -1.44, 0.33 | 0.224 | 0.940 |
| c54840 | X - 24812 | -0.25 | -0.61, 0.12 | 0.189 | 0.844 | -0.25 | -0.61, 0.11 | 0.172 | 0.940 |
| c57714 | X - 24947 | -0.10 | -0.35, 0.15 | 0.444 | 0.932 | -0.09 | -0.33, 0.16 | 0.496 | 0.980 |
| c57716 | X - 24949 | -0.07 | -0.38, 0.24 | 0.651 | 0.941 | -0.12 | -0.43, 0.19 | 0.453 | 0.980 |
| c57720 | X - 24953 | 0.29 | -0.15, 0.72 | 0.204 | 0.844 | 0.27 | -0.16, 0.70 | 0.217 | 0.940 |
| c62636 | X - 25343 | 0.06 | -0.14, 0.25 | 0.581 | 0.941 | 0.10 | -0.09, 0.28 | 0.304 | 0.940 |
| c62664 | X - 25371 | -0.45 | -1.22, 0.31 | 0.248 | 0.891 | 0.23 | -0.60, 1.05 | 0.593 | 0.980 |
| c62716 | X - 25419 | -0.01 | -0.25, 0.22 | 0.913 | 0.982 | -0.06 | -0.29, 0.16 | 0.589 | 0.980 |
| c62717 | X - 25420 | 0.24 | -0.18, 0.66 | 0.269 | 0.895 | 0.19 | -0.21, 0.60 | 0.345 | 0.968 |
| c62719 | X - 25422 | 0.17 | -0.14, 0.47 | 0.283 | 0.898 | 0.21 | -0.09, 0.51 | 0.167 | 0.940 |
| c62963 | X - 25519 | 0.21 | -0.05, 0.46 | 0.113 | 0.844 | 0.21 | -0.04, 0.46 | 0.108 | 0.940 |
| c62964 | X - 25520 | -0.09 | -0.34, 0.17 | 0.502 | 0.941 | -0.03 | -0.29, 0.22 | 0.808 | 0.993 |
| c63560 | X - 25790 | -0.20 | -0.68, 0.27 | 0.406 | 0.919 | -0.29 | -0.76, 0.17 | 0.217 | 0.940 |
| c63908 | X - 25957 | 0.13 | -0.29, 0.54 | 0.547 | 0.941 | 0.10 | -0.31, 0.50 | 0.648 | 0.980 |

^1^ Estimates are from robust mixed effects multivariable linear regression models including %DBV as the dependent variable; childhood fixed effects – ln(metabolite level) (continuous), BMI z-score (continuous), age at BMI measurement (continuous), treatment group assignment; adult fixed effects - BMI and BMI^2^ at breast density (continuous); and DISC clinic as a random effect.

^2^ Estimates are from robust mixed effects multivariable linear regression models including %DBV as the dependent variable; childhood fixed effects – ln(metabolite level) (continuous), BMI z-score (continuous), age at BMI measurement (continuous), treatment group assignment, race (white/non-white), and menstrual cycle phase at blood collection (premenarche/luteal/follicular/unknown); adult fixed effects - BMI and BMI^2^ at breast density (continuous), college graduate (yes/no), duration hormone use (continuous), number live births (0/1+), current smoker (yes/no); and DISC clinic as a random effect.
