# Supplementary material for: Childhood adiposity, serum metabolites and breast density in young women
Source: Breast Cancer Res. 2022 Dec 19;24:91. doi: 10.1186/s13058-022-01588-y (PMC9764542; doi:10.1186/s13058-022-01588-y)
Supplement: Supplementary file 3 — Additional file 3. Difference in ADBV associated with a 10% increase in metabolite. [file 13058_2022_1588_MOESM3_ESM.docx]

| **Supplemental Table 3. Difference in ADBV associated with 10% increase in serum metabolite** | | | | | | | | | |  |  |  |  |  |  |  |  |  |  |
| --- | --- | --- | --- | --- | --- | --- | --- | --- | --- | --- | --- | --- | --- | --- | --- | --- | --- | --- | --- |
|  |  | **Minimally Adjusted Model^1^** | | | | **Fully Adjusted Model^2^** | | | |  |  |  |  |  |  |  |  |  |  |
| **Compid** | **Biochemical** | **Δ** | **95% CI** | **P-value** | **Q-value** | **Δ** | **95% CI** | **P-value** | **Q-value** |  |  |  |  |  |  |  |  |  |  |
|  | **AMINO ACID** |  |  |  |  |  |  |  |  |  |  |  |  |  |  |  |  |  |  |
|  | **Glycine, Serine and Threonine Metabolism** | |  |  |  |  |  |  |  |  |  |  |  |  |  |  |  |  |  |
| c58 | glycine | 0.45 | -4.22, 5.35 | 0.854 | 0.997 | 1.13 | -3.37, 5.83 | 0.630 | 0.999 |  |  |  |  |  |  |  |  |  |  |
| c27710 | N-acetylglycine | 2.18 | -0.76, 5.20 | 0.149 | 0.962 | 2.24 | -0.54, 5.09 | 0.117 | 0.999 |  |  |  |  |  |  |  |  |  |  |
| c1516 | sarcosine | 0.58 | -2.12, 3.37 | 0.676 | 0.997 | -0.77 | -3.39, 1.91 | 0.569 | 0.999 |  |  |  |  |  |  |  |  |  |  |
| c5086 | dimethylglycine | -2.39 | -5.60, 0.93 | 0.158 | 0.962 | -2.38 | -5.42, 0.76 | 0.138 | 0.999 |  |  |  |  |  |  |  |  |  |  |
| c3141 | betaine | 0.15 | -4.71, 5.26 | 0.952 | 0.997 | 1.07 | -3.56, 5.92 | 0.657 | 0.999 |  |  |  |  |  |  |  |  |  |  |
| c1648 | serine | -0.40 | -4.64, 4.02 | 0.855 | 0.997 | -1.11 | -5.11, 3.05 | 0.596 | 0.999 |  |  |  |  |  |  |  |  |  |  |
| c37076 | N-acetylserine | -1.71 | -6.88, 3.75 | 0.533 | 0.997 | -0.08 | -5.14, 5.25 | 0.975 | 0.999 |  |  |  |  |  |  |  |  |  |  |
| c1284 | threonine | -0.83 | -5.25, 3.80 | 0.722 | 0.997 | -3.27 | -7.37, 1.01 | 0.134 | 0.999 |  |  |  |  |  |  |  |  |  |  |
| c33939 | N-acetylthreonine | -3.41 | -9.15, 2.70 | 0.270 | 0.969 | -1.61 | -7.24, 4.37 | 0.590 | 0.999 |  |  |  |  |  |  |  |  |  |  |
|  | **Alanine and Aspartate Metabolism** |  |  |  |  |  |  |  |  |  |  |  |  |  |  |  |  |  |  |
| c1126 | alanine | 2.54 | -3.09, 8.49 | 0.385 | 0.994 | 2.48 | -2.91, 8.18 | 0.375 | 0.999 |  |  |  |  |  |  |  |  |  |  |
| c1585 | N-acetylalanine | 0.16 | -6.79, 7.64 | 0.964 | 0.999 | -0.03 | -6.66, 7.07 | 0.993 | 0.999 |  |  |  |  |  |  |  |  |  |  |
| c443 | aspartate | -0.48 | -2.90, 2.00 | 0.703 | 0.997 | -0.54 | -2.83, 1.82 | 0.654 | 0.999 |  |  |  |  |  |  |  |  |  |  |
| c22185 | N-acetylaspartate (NAA) | -0.90 | -4.74, 3.09 | 0.653 | 0.997 | -0.35 | -4.03, 3.47 | 0.856 | 0.999 |  |  |  |  |  |  |  |  |  |  |
| c512 | asparagine | 1.55 | -2.28, 5.54 | 0.434 | 0.994 | -0.08 | -3.70, 3.67 | 0.966 | 0.999 |  |  |  |  |  |  |  |  |  |  |
| c33942 | N-acetylasparagine | 2.19 | -0.91, 5.39 | 0.169 | 0.962 | 1.55 | -1.44, 4.63 | 0.314 | 0.999 |  |  |  |  |  |  |  |  |  |  |
|  | **Glutamate Metabolism** |  |  |  |  |  |  |  |  |  |  |  |  |  |  |  |  |  |  |
| c57 | glutamate | 0.49 | -1.52, 2.54 | 0.639 | 0.997 | 0.67 | -1.25, 2.62 | 0.500 | 0.999 |  |  |  |  |  |  |  |  |  |  |
| c53 | glutamine | 0.53 | -2.99, 4.19 | 0.770 | 0.997 | -0.05 | -3.38, 3.40 | 0.977 | 0.999 |  |  |  |  |  |  |  |  |  |  |
| c15720 | N-acetylglutamate | -4.20 | -7.88,-0.37 | 0.033 | 0.962 | -3.26 | -6.83, 0.46 | 0.087 | 0.999 |  |  |  |  |  |  |  |  |  |  |
| c46225 | pyroglutamine* | -0.04 | -2.09, 2.06 | 0.971 | 0.999 | -0.25 | -2.20, 1.75 | 0.808 | 0.999 |  |  |  |  |  |  |  |  |  |  |
| c35665 | N-acetyl-aspartyl-glutamate (NAAG) | -0.80 | -2.67, 1.11 | 0.412 | 0.994 | -1.01 | -2.80, 0.81 | 0.279 | 0.999 |  |  |  |  |  |  |  |  |  |  |
| c54923 | beta-citrylglutamate | 0.02 | -1.79, 1.86 | 0.984 | 0.999 | -0.14 | -1.84, 1.59 | 0.876 | 0.999 |  |  |  |  |  |  |  |  |  |  |
| c42370 | S-1-pyrroline-5-carboxylate | -0.66 | -3.10, 1.84 | 0.601 | 0.997 | -0.07 | -2.44, 2.35 | 0.955 | 0.999 |  |  |  |  |  |  |  |  |  |  |
|  | **Histidine Metabolism** |  |  |  |  |  |  |  |  |  |  |  |  |  |  |  |  |  |  |
| c59 | histidine | 1.70 | -3.12, 6.75 | 0.497 | 0.994 | 2.00 | -2.64, 6.86 | 0.405 | 0.999 |  |  |  |  |  |  |  |  |  |  |
| c30460 | 1-methylhistidine | -1.51 | -4.33, 1.39 | 0.305 | 0.969 | -1.89 | -4.55, 0.85 | 0.177 | 0.999 |  |  |  |  |  |  |  |  |  |  |
| c33946 | N-acetylhistidine | -0.48 | -2.93, 2.03 | 0.704 | 0.997 | -0.17 | -2.55, 2.28 | 0.894 | 0.999 |  |  |  |  |  |  |  |  |  |  |
| c43255 | N-acetyl-1-methylhistidine* | -0.50 | -2.17, 1.20 | 0.562 | 0.997 | -0.53 | -2.10, 1.07 | 0.517 | 0.999 |  |  |  |  |  |  |  |  |  |  |
| c40473 | hydantoin-5-propionate | -0.01 | -1.45, 1.44 | 0.986 | 0.999 | -0.34 | -1.71, 1.06 | 0.637 | 0.999 |  |  |  |  |  |  |  |  |  |  |
| c607 | trans-urocanate | -1.36 | -3.24, 0.56 | 0.173 | 0.962 | -1.49 | -3.27, 0.32 | 0.112 | 0.999 |  |  |  |  |  |  |  |  |  |  |
| c40730 | imidazole propionate | -0.07 | -1.65, 1.54 | 0.934 | 0.997 | 0.00 | -1.50, 1.52 | 0.998 | 0.999 |  |  |  |  |  |  |  |  |  |  |
| c15716 | imidazole lactate | -0.79 | -4.21, 2.74 | 0.656 | 0.997 | -1.20 | -4.44, 2.14 | 0.477 | 0.999 |  |  |  |  |  |  |  |  |  |  |
| c43488 | N-acetylcarnosine | -0.52 | -3.14, 2.17 | 0.700 | 0.997 | -0.48 | -3.03, 2.14 | 0.717 | 0.999 |  |  |  |  |  |  |  |  |  |  |
| c32350 | 1-methyl-4-imidazoleacetate | -0.50 | -4.91, 4.12 | 0.830 | 0.997 | 1.50 | -2.85, 6.05 | 0.506 | 0.999 |  |  |  |  |  |  |  |  |  |  |
|  | **Lysine Metabolism** |  |  |  |  |  |  |  |  |  |  |  |  |  |  |  |  |  |  |
| c1301 | lysine | -6.75 | -12.23,-0.92 | 0.025 | 0.962 | -7.68 | -12.76,-2.31 | 0.006 | 0.711 |  |  |  |  |  |  |  |  |  |  |
| c36752 | N6-acetyllysine | -4.41 | -8.29,-0.36 | 0.035 | 0.962 | -3.23 | -7.01, 0.70 | 0.108 | 0.999 |  |  |  |  |  |  |  |  |  |  |
| c1498 | N6,N6,N6-trimethyllysine | -3.76 | -6.73,-0.70 | 0.018 | 0.962 | -3.18 | -6.10,-0.17 | 0.040 | 0.999 |  |  |  |  |  |  |  |  |  |  |
| c15685 | 5-hydroxylysine | -0.93 | -3.41, 1.62 | 0.473 | 0.994 | -0.80 | -3.26, 1.72 | 0.530 | 0.999 |  |  |  |  |  |  |  |  |  |  |
| c44664 | glutarylcarnitine (C5-DC) | 0.38 | -2.46, 3.30 | 0.797 | 0.997 | -0.90 | -3.60, 1.88 | 0.524 | 0.999 |  |  |  |  |  |  |  |  |  |  |
| c1444 | pipecolate | -0.65 | -2.36, 1.09 | 0.464 | 0.994 | -1.63 | -3.27, 0.03 | 0.057 | 0.999 |  |  |  |  |  |  |  |  |  |  |
|  | **Phenylalanine Metabolism** |  |  |  |  |  |  |  |  |  |  |  |  |  |  |  |  |  |  |
| c64 | phenylalanine | -0.21 | -4.20, 3.95 | 0.920 | 0.997 | -0.82 | -4.62, 3.13 | 0.679 | 0.999 |  |  |  |  |  |  |  |  |  |  |
| c33950 | N-acetylphenylalanine | 0.19 | -2.45, 2.91 | 0.887 | 0.997 | 0.18 | -2.33, 2.76 | 0.889 | 0.999 |  |  |  |  |  |  |  |  |  |  |
| c566 | phenylpyruvate | -0.98 | -2.34, 0.41 | 0.169 | 0.962 | -0.87 | -2.17, 0.44 | 0.193 | 0.999 |  |  |  |  |  |  |  |  |  |  |
| c22130 | phenyllactate (PLA) | -0.76 | -4.69, 3.33 | 0.711 | 0.997 | -1.72 | -5.39, 2.10 | 0.375 | 0.999 |  |  |  |  |  |  |  |  |  |  |
|  | **Tyrosine Metabolism** |  |  |  |  |  |  |  |  |  |  |  |  |  |  |  |  |  |  |
| c1299 | tyrosine | 1.53 | -3.21, 6.50 | 0.535 | 0.997 | -0.26 | -4.67, 4.35 | 0.909 | 0.999 |  |  |  |  |  |  |  |  |  |  |
| c32197 | 3-(4-hydroxyphenyl)lactate (HPLA) | -0.13 | -4.30, 4.23 | 0.953 | 0.997 | -1.23 | -5.16, 2.86 | 0.551 | 0.999 |  |  |  |  |  |  |  |  |  |  |
| c32553 | phenol sulfate | -0.05 | -2.24, 2.19 | 0.964 | 0.999 | 0.08 | -2.01, 2.21 | 0.942 | 0.999 |  |  |  |  |  |  |  |  |  |  |
| c1567 | vanillylmandelate (VMA) | -0.92 | -5.11, 3.46 | 0.677 | 0.997 | 0.23 | -3.84, 4.48 | 0.914 | 0.999 |  |  |  |  |  |  |  |  |  |  |
| c12017 | 3-methoxytyrosine | -0.39 | -3.85, 3.19 | 0.828 | 0.997 | -0.48 | -3.74, 2.89 | 0.778 | 0.999 |  |  |  |  |  |  |  |  |  |  |
| c48841 | p-cresol glucuronide* | -0.36 | -1.29, 0.57 | 0.447 | 0.994 | -0.49 | -1.39, 0.42 | 0.290 | 0.999 |  |  |  |  |  |  |  |  |  |  |
| c2761 | thyroxine | -3.24 | -7.98, 1.74 | 0.200 | 0.962 | -1.56 | -6.20, 3.32 | 0.526 | 0.999 |  |  |  |  |  |  |  |  |  |  |
|  | **Tryptophan Metabolism** |  |  |  |  |  |  |  |  |  |  |  |  |  |  |  |  |  |  |
| c54 | tryptophan | -3.59 | -8.13, 1.17 | 0.139 | 0.962 | -3.01 | -7.36, 1.55 | 0.194 | 0.999 |  |  |  |  |  |  |  |  |  |  |
| c33959 | N-acetyltryptophan | -0.69 | -2.78, 1.44 | 0.523 | 0.997 | -0.28 | -2.32, 1.80 | 0.789 | 0.999 |  |  |  |  |  |  |  |  |  |  |
| c48782 | C-glycosyltryptophan | -8.77 | -14.39,-2.79 | 0.005 | 0.589 | -7.29 | -12.84,-1.38 | 0.017 | 0.841 |  |  |  |  |  |  |  |  |  |  |
| c37097 | tryptophan betaine | 0.33 | -0.79, 1.46 | 0.566 | 0.997 | 0.71 | -0.41, 1.84 | 0.214 | 0.999 |  |  |  |  |  |  |  |  |  |  |
| c15140 | kynurenine | -3.10 | -6.25, 0.15 | 0.063 | 0.962 | -2.72 | -5.76, 0.42 | 0.091 | 0.999 |  |  |  |  |  |  |  |  |  |  |
| c1417 | kynurenate | -1.45 | -4.02, 1.20 | 0.283 | 0.969 | -1.06 | -3.51, 1.45 | 0.404 | 0.999 |  |  |  |  |  |  |  |  |  |  |
| c15679 | xanthurenate | -0.66 | -2.28, 1.00 | 0.436 | 0.994 | -0.86 | -2.39, 0.68 | 0.272 | 0.999 |  |  |  |  |  |  |  |  |  |  |
| c2342 | serotonin | 0.47 | -1.24, 2.22 | 0.593 | 0.997 | 0.17 | -1.45, 1.81 | 0.841 | 0.999 |  |  |  |  |  |  |  |  |  |  |
| c18349 | indolelactate | -1.28 | -5.23, 2.83 | 0.537 | 0.997 | -1.02 | -4.80, 2.91 | 0.606 | 0.999 |  |  |  |  |  |  |  |  |  |  |
| c27513 | indoleacetate | 0.01 | -2.77, 2.86 | 0.996 | 0.999 | 0.07 | -2.59, 2.81 | 0.957 | 0.999 |  |  |  |  |  |  |  |  |  |  |
| c32405 | indolepropionate | 0.21 | -1.24, 1.68 | 0.780 | 0.997 | 0.12 | -1.31, 1.57 | 0.870 | 0.999 |  |  |  |  |  |  |  |  |  |  |
| c27672 | 3-indoxyl sulfate | -1.48 | -4.00, 1.09 | 0.258 | 0.969 | -2.12 | -4.49, 0.32 | 0.090 | 0.999 |  |  |  |  |  |  |  |  |  |  |
|  | **Leucine, Isoleucine and Valine Metabolism** | |  |  |  |  |  |  |  |  |  |  |  |  |  |  |  |  |  |
| c60 | leucine | -1.63 | -7.86, 5.01 | 0.622 | 0.997 | -2.51 | -8.37, 3.73 | 0.424 | 0.999 |  |  |  |  |  |  |  |  |  |  |
| c22116 | 4-methyl-2-oxopentanoate | -0.25 | -2.35, 1.89 | 0.816 | 0.997 | -0.43 | -2.40, 1.58 | 0.674 | 0.999 |  |  |  |  |  |  |  |  |  |  |
| c44656 | isovalerate (C5) | -0.03 | -0.99, 0.93 | 0.945 | 0.997 | -0.14 | -1.05, 0.78 | 0.762 | 0.999 |  |  |  |  |  |  |  |  |  |  |
| c34407 | isovalerylcarnitine (C5) | -2.19 | -4.78, 0.47 | 0.107 | 0.962 | -3.67 | -6.15,-1.12 | 0.006 | 0.711 |  |  |  |  |  |  |  |  |  |  |
| c12129 | beta-hydroxyisovalerate | 1.39 | -1.79, 4.67 | 0.397 | 0.994 | -0.21 | -3.32, 3.00 | 0.897 | 0.999 |  |  |  |  |  |  |  |  |  |  |
| c46548 | 3-methylglutarylcarnitine (2) | -2.36 | -4.28,-0.40 | 0.020 | 0.962 | -1.84 | -3.87, 0.23 | 0.083 | 0.999 |  |  |  |  |  |  |  |  |  |  |
| c1125 | isoleucine | -1.16 | -7.28, 5.37 | 0.721 | 0.997 | -2.20 | -7.96, 3.92 | 0.473 | 0.999 |  |  |  |  |  |  |  |  |  |  |
| c15676 | 3-methyl-2-oxovalerate | -0.62 | -2.74, 1.54 | 0.570 | 0.997 | -0.76 | -2.74, 1.27 | 0.465 | 0.999 |  |  |  |  |  |  |  |  |  |  |
| c36746 | 2-hydroxy-3-methylvalerate | -1.37 | -3.94, 1.27 | 0.307 | 0.969 | -1.02 | -3.46, 1.48 | 0.421 | 0.999 |  |  |  |  |  |  |  |  |  |  |
| c45095 | 2-methylbutyrylcarnitine (C5) | -2.77 | -6.15, 0.73 | 0.121 | 0.962 | -3.97 | -7.13,-0.71 | 0.019 | 0.841 |  |  |  |  |  |  |  |  |  |  |
| c32397 | 3-hydroxy-2-ethylpropionate | -1.96 | -5.59, 1.81 | 0.305 | 0.969 | -2.60 | -6.00, 0.92 | 0.147 | 0.999 |  |  |  |  |  |  |  |  |  |  |
| c15765 | ethylmalonate | -1.35 | -3.46, 0.79 | 0.216 | 0.969 | -0.79 | -2.83, 1.30 | 0.458 | 0.999 |  |  |  |  |  |  |  |  |  |  |
| c53031 | methylsuccinoylcarnitine | -1.43 | -3.10, 0.28 | 0.103 | 0.962 | -0.98 | -2.60, 0.67 | 0.244 | 0.999 |  |  |  |  |  |  |  |  |  |  |
| c1649 | valine | -4.56 | -9.62, 0.77 | 0.094 | 0.962 | -5.28 | -10.01,-0.31 | 0.039 | 0.999 |  |  |  |  |  |  |  |  |  |  |
| c1591 | N-acetylvaline | -4.31 | -9.33, 0.98 | 0.110 | 0.962 | -4.55 | -9.25, 0.39 | 0.072 | 0.999 |  |  |  |  |  |  |  |  |  |  |
| c44526 | 3-methyl-2-oxobutyrate | 0.00 | -1.79, 1.83 | 0.997 | 0.999 | -0.18 | -1.87, 1.54 | 0.837 | 0.999 |  |  |  |  |  |  |  |  |  |  |
| c33937 | alpha-hydroxyisovalerate | -1.35 | -4.73, 2.14 | 0.445 | 0.994 | -1.58 | -4.75, 1.70 | 0.342 | 0.999 |  |  |  |  |  |  |  |  |  |  |
| c33441 | isobutyrylcarnitine (C4) | 0.44 | -1.94, 2.89 | 0.718 | 0.997 | -0.29 | -2.54, 2.02 | 0.805 | 0.999 |  |  |  |  |  |  |  |  |  |  |
|  | **Methionine, Cysteine, SAM and Taurine Metabolism** | |  |  |  |  |  |  |  |  |  |  |  |  |  |  |  |  |  |
| c1302 | methionine | -0.27 | -4.03, 3.63 | 0.889 | 0.997 | -1.07 | -4.58, 2.58 | 0.562 | 0.999 |  |  |  |  |  |  |  |  |  |  |
| c1589 | N-acetylmethionine | 0.37 | -0.63, 1.37 | 0.474 | 0.994 | -0.01 | -0.96, 0.94 | 0.976 | 0.999 |  |  |  |  |  |  |  |  |  |  |
| c2829 | N-formylmethionine | -0.78 | -3.88, 2.42 | 0.631 | 0.997 | -1.17 | -4.06, 1.80 | 0.437 | 0.999 |  |  |  |  |  |  |  |  |  |  |
| c44878 | methionine sulfone | -1.95 | -4.45, 0.61 | 0.136 | 0.962 | -1.65 | -4.09, 0.86 | 0.197 | 0.999 |  |  |  |  |  |  |  |  |  |  |
| c18374 | methionine sulfoxide | 0.63 | -0.67, 1.96 | 0.348 | 0.994 | 0.34 | -0.92, 1.60 | 0.603 | 0.999 |  |  |  |  |  |  |  |  |  |  |
| c45428 | N-acetylmethionine sulfoxide | 0.38 | -0.48, 1.24 | 0.395 | 0.994 | 0.23 | -0.58, 1.06 | 0.575 | 0.999 |  |  |  |  |  |  |  |  |  |  |
| c42382 | S-adenosylhomocysteine (SAH) | -0.20 | -1.76, 1.39 | 0.804 | 0.997 | 0.19 | -1.28, 1.68 | 0.803 | 0.999 |  |  |  |  |  |  |  |  |  |  |
| c15705 | cystathionine | -0.33 | -1.37, 0.73 | 0.544 | 0.997 | -0.17 | -1.16, 0.84 | 0.740 | 0.999 |  |  |  |  |  |  |  |  |  |  |
| c39592 | S-methylcysteine | -0.11 | -2.06, 1.87 | 0.910 | 0.997 | -0.34 | -2.21, 1.57 | 0.728 | 0.999 |  |  |  |  |  |  |  |  |  |  |
| c22176 | cysteine s-sulfate | -0.31 | -1.71, 1.11 | 0.666 | 0.997 | -0.19 | -1.55, 1.19 | 0.788 | 0.999 |  |  |  |  |  |  |  |  |  |  |
| c37443 | cysteine sulfinic acid | 0.79 | -1.30, 2.92 | 0.465 | 0.994 | 0.53 | -1.45, 2.56 | 0.604 | 0.999 |  |  |  |  |  |  |  |  |  |  |
| c590 | hypotaurine | -0.25 | -1.88, 1.41 | 0.767 | 0.997 | -0.37 | -1.92, 1.21 | 0.647 | 0.999 |  |  |  |  |  |  |  |  |  |  |
| c2125 | taurine | -0.21 | -2.82, 2.47 | 0.878 | 0.997 | -0.77 | -3.25, 1.78 | 0.553 | 0.999 |  |  |  |  |  |  |  |  |  |  |
|  | **Urea cycle; Arginine and Proline Metabolism** | |  |  |  |  |  |  |  |  |  |  |  |  |  |  |  |  |  |
| c1638 | arginine | -1.30 | -4.81, 2.33 | 0.479 | 0.994 | -1.43 | -4.80, 2.05 | 0.416 | 0.999 |  |  |  |  |  |  |  |  |  |  |
| c1670 | urea | -0.89 | -4.84, 3.23 | 0.669 | 0.997 | -2.25 | -5.93, 1.58 | 0.248 | 0.999 |  |  |  |  |  |  |  |  |  |  |
| c1493 | ornithine | -1.27 | -4.64, 2.23 | 0.474 | 0.994 | -1.48 | -4.69, 1.84 | 0.380 | 0.999 |  |  |  |  |  |  |  |  |  |  |
| c55072 | 2-oxoarginine* | -0.71 | -2.23, 0.84 | 0.370 | 0.994 | -0.86 | -2.28, 0.59 | 0.246 | 0.999 |  |  |  |  |  |  |  |  |  |  |
| c2132 | citrulline | -3.34 | -7.37, 0.87 | 0.121 | 0.962 | -2.48 | -6.37, 1.56 | 0.227 | 0.999 |  |  |  |  |  |  |  |  |  |  |
| c22137 | homoarginine | -0.75 | -3.63, 2.21 | 0.615 | 0.997 | -2.71 | -5.40, 0.06 | 0.056 | 0.999 |  |  |  |  |  |  |  |  |  |  |
| c1898 | proline | 0.65 | -3.16, 4.62 | 0.742 | 0.997 | 0.23 | -3.38, 3.97 | 0.904 | 0.999 |  |  |  |  |  |  |  |  |  |  |
| c36808 | dimethylarginine (ADMA + SDMA) | -6.93 | -12.87,-0.59 | 0.034 | 0.962 | -7.42 | -13.16,-1.31 | 0.019 | 0.841 |  |  |  |  |  |  |  |  |  |  |
| c33953 | N-acetylarginine | 0.02 | -2.54, 2.65 | 0.987 | 0.999 | -0.23 | -2.64, 2.24 | 0.855 | 0.999 |  |  |  |  |  |  |  |  |  |  |
| c43249 | N-delta-acetylornithine | -0.69 | -2.50, 1.17 | 0.466 | 0.994 | -0.56 | -2.30, 1.21 | 0.531 | 0.999 |  |  |  |  |  |  |  |  |  |  |
| c32306 | hydroxyproline | 0.25 | -3.47, 4.12 | 0.896 | 0.997 | -0.05 | -3.66, 3.69 | 0.978 | 0.999 |  |  |  |  |  |  |  |  |  |  |
| c35127 | prolylhydroxyproline | 0.39 | -0.55, 1.34 | 0.419 | 0.994 | -0.02 | -0.90, 0.87 | 0.963 | 0.999 |  |  |  |  |  |  |  |  |  |  |
| c37431 | N-methylproline | -0.12 | -1.15, 0.92 | 0.822 | 0.997 | -0.14 | -1.13, 0.86 | 0.788 | 0.999 |  |  |  |  |  |  |  |  |  |  |
|  | **Creatine Metabolism** |  |  |  |  |  |  |  |  |  |  |  |  |  |  |  |  |  |  |
| c27718 | creatine | -2.40 | -5.43, 0.72 | 0.132 | 0.962 | -3.63 | -6.47,-0.70 | 0.016 | 0.841 |  |  |  |  |  |  |  |  |  |  |
| c513 | creatinine | 0.27 | -7.12, 8.24 | 0.946 | 0.997 | -0.85 | -7.77, 6.60 | 0.818 | 0.999 |  |  |  |  |  |  |  |  |  |  |
|  | **Polyamine Metabolism** |  |  |  |  |  |  |  |  |  |  |  |  |  |  |  |  |  |  |
| c37496 | N-acetylputrescine | -0.59 | -2.74, 1.61 | 0.604 | 0.997 | -0.32 | -2.34, 1.76 | 0.767 | 0.999 |  |  |  |  |  |  |  |  |  |  |
| c485 | spermidine | -1.08 | -2.27, 0.13 | 0.083 | 0.962 | -1.19 | -2.36, 0.00 | 0.051 | 0.999 |  |  |  |  |  |  |  |  |  |  |
| c1419 | 5-methylthioadenosine (MTA) | 0.06 | -1.21, 1.35 | 0.928 | 0.997 | -0.29 | -1.49, 0.93 | 0.642 | 0.999 |  |  |  |  |  |  |  |  |  |  |
|  | **Guanidino and Acetamido Metabolism** |  |  |  |  |  |  |  |  |  |  |  |  |  |  |  |  |  |  |
| c15681 | 4-guanidinobutanoate | -0.35 | -1.59, 0.92 | 0.594 | 0.997 | -0.23 | -1.42, 0.97 | 0.706 | 0.999 |  |  |  |  |  |  |  |  |  |  |
|  | **Glutathione Metabolism** |  |  |  |  |  |  |  |  |  |  |  |  |  |  |  |  |  |  |
| c35637 | cysteinylglycine | -0.26 | -1.38, 0.88 | 0.656 | 0.997 | -0.05 | -1.12, 1.04 | 0.929 | 0.999 |  |  |  |  |  |  |  |  |  |  |
| c1494 | 5-oxoproline | 0.95 | -1.04, 2.98 | 0.355 | 0.994 | 1.40 | -0.51, 3.34 | 0.153 | 0.999 |  |  |  |  |  |  |  |  |  |  |
| c42374 | 2-aminobutyrate | 0.90 | -2.23, 4.14 | 0.577 | 0.997 | -0.71 | -3.68, 2.36 | 0.648 | 0.999 |  |  |  |  |  |  |  |  |  |  |
| c52281 | 2-hydroxybutyrate/2-hydroxyisobutyrate | 1.15 | -1.51, 3.88 | 0.403 | 0.994 | 0.49 | -2.04, 3.08 | 0.708 | 0.999 |  |  |  |  |  |  |  |  |  |  |
|  | **PEPTIDE** |  |  |  |  |  |  |  |  |  |  |  |  |  |  |  |  |  |  |
|  | **Gamma-glutamyl Amino Acid** |  |  |  |  |  |  |  |  |  |  |  |  |  |  |  |  |  |  |
| c37063 | gamma-glutamylalanine | 0.39 | -1.05, 1.84 | 0.600 | 0.997 | 0.06 | -1.31, 1.45 | 0.929 | 0.999 |  |  |  |  |  |  |  |  |  |  |
| c36738 | gamma-glutamylglutamate | 0.38 | -0.48, 1.25 | 0.392 | 0.994 | 0.27 | -0.57, 1.11 | 0.535 | 0.999 |  |  |  |  |  |  |  |  |  |  |
| c2730 | gamma-glutamylglutamine | 1.12 | -0.54, 2.79 | 0.189 | 0.962 | 0.42 | -1.15, 2.01 | 0.603 | 0.999 |  |  |  |  |  |  |  |  |  |  |
| c33949 | gamma-glutamylglycine | 1.13 | -0.20, 2.48 | 0.101 | 0.962 | 0.78 | -0.42, 2.00 | 0.206 | 0.999 |  |  |  |  |  |  |  |  |  |  |
| c34456 | gamma-glutamylisoleucine* | 0.35 | -0.78, 1.49 | 0.552 | 0.997 | 0.41 | -0.67, 1.50 | 0.464 | 0.999 |  |  |  |  |  |  |  |  |  |  |
| c18369 | gamma-glutamylleucine | 0.28 | -1.10, 1.68 | 0.697 | 0.997 | 0.30 | -1.03, 1.65 | 0.659 | 0.999 |  |  |  |  |  |  |  |  |  |  |
| c55015 | gamma-glutamyl-alpha-lysine | 0.27 | -1.43, 1.99 | 0.760 | 0.997 | -0.09 | -1.73, 1.58 | 0.916 | 0.999 |  |  |  |  |  |  |  |  |  |  |
| c44872 | gamma-glutamylmethionine | 0.34 | -1.04, 1.74 | 0.630 | 0.997 | 0.23 | -1.09, 1.57 | 0.733 | 0.999 |  |  |  |  |  |  |  |  |  |  |
| c33422 | gamma-glutamylphenylalanine | -1.06 | -2.48, 0.37 | 0.154 | 0.962 | -0.52 | -1.92, 0.89 | 0.469 | 0.999 |  |  |  |  |  |  |  |  |  |  |
| c33364 | gamma-glutamylthreonine | 0.66 | -1.09, 2.44 | 0.464 | 0.994 | 0.45 | -1.22, 2.16 | 0.599 | 0.999 |  |  |  |  |  |  |  |  |  |  |
| c2734 | gamma-glutamyltyrosine | -0.64 | -2.74, 1.51 | 0.560 | 0.997 | -0.50 | -2.56, 1.60 | 0.638 | 0.999 |  |  |  |  |  |  |  |  |  |  |
| c43829 | gamma-glutamylvaline | 0.51 | -0.63, 1.67 | 0.385 | 0.994 | 0.45 | -0.65, 1.57 | 0.424 | 0.999 |  |  |  |  |  |  |  |  |  |  |
| c54914 | gamma-glutamylserine | 0.77 | -0.96, 2.53 | 0.390 | 0.994 | 0.45 | -1.21, 2.13 | 0.601 | 0.999 |  |  |  |  |  |  |  |  |  |  |
|  | **Dipeptide** |  |  |  |  |  |  |  |  |  |  |  |  |  |  |  |  |  |  |
| c42027 | histidylalanine | -0.48 | -1.45, 0.49 | 0.330 | 0.990 | -0.42 | -1.34, 0.51 | 0.381 | 0.999 |  |  |  |  |  |  |  |  |  |  |
| c40010 | leucylalanine | -0.41 | -1.17, 0.35 | 0.291 | 0.969 | -0.40 | -1.11, 0.32 | 0.281 | 0.999 |  |  |  |  |  |  |  |  |  |  |
| c40045 | leucylglycine | 0.32 | -0.86, 1.52 | 0.595 | 0.997 | 0.29 | -0.86, 1.44 | 0.626 | 0.999 |  |  |  |  |  |  |  |  |  |  |
| c39994 | valylleucine | -0.26 | -1.18, 0.67 | 0.582 | 0.997 | -0.23 | -1.10, 0.64 | 0.601 | 0.999 |  |  |  |  |  |  |  |  |  |  |
|  | **Acetylated Peptides** |  |  |  |  |  |  |  |  |  |  |  |  |  |  |  |  |  |  |
| c48425 | phenylacetylcarnitine | -0.60 | -1.83, 0.65 | 0.350 | 0.994 | -1.02 | -2.18, 0.15 | 0.089 | 0.999 |  |  |  |  |  |  |  |  |  |  |
| c35126 | phenylacetylglutamine | 0.11 | -1.91, 2.17 | 0.919 | 0.997 | -0.53 | -2.44, 1.42 | 0.593 | 0.999 |  |  |  |  |  |  |  |  |  |  |
| c55017 | 4-hydroxyphenylacetylglutamine | -1.54 | -2.96,-0.10 | 0.038 | 0.962 | -1.02 | -2.40, 0.37 | 0.153 | 0.999 |  |  |  |  |  |  |  |  |  |  |
|  | **CARBOHYDRATE** |  |  |  |  |  |  |  |  |  |  |  |  |  |  |  |  |  |  |
|  | **Glycolysis, Gluconeogenesis, and Pyruvate Metabolism** | | |  |  |  |  |  |  |  |  |  |  |  |  |  |  |  |  |
| c20675 | 1,5-anhydroglucitol (1,5-AG) | -0.03 | -3.92, 4.02 | 0.990 | 0.999 | 1.46 | -2.39, 5.47 | 0.463 | 0.999 |  |  |  |  |  |  |  |  |  |  |
| c48152 | glucose | 4.23 | -5.26,14.68 | 0.396 | 0.994 | 6.00 | -3.26,16.14 | 0.214 | 0.999 |  |  |  |  |  |  |  |  |  |  |
| c48990 | pyruvate | -0.05 | -0.97, 0.89 | 0.922 | 0.997 | -0.02 | -0.90, 0.88 | 0.972 | 0.999 |  |  |  |  |  |  |  |  |  |  |
| c527 | lactate | 2.03 | -1.40, 5.59 | 0.251 | 0.969 | 1.36 | -1.89, 4.71 | 0.419 | 0.999 |  |  |  |  |  |  |  |  |  |  |
| c1572 | glycerate | 0.97 | -1.78, 3.79 | 0.497 | 0.994 | 0.83 | -1.84, 3.57 | 0.549 | 0.999 |  |  |  |  |  |  |  |  |  |  |
|  | **Pentose Metabolism** |  |  |  |  |  |  |  |  |  |  |  |  |  |  |  |  |  |  |
| c15772 | ribitol | -3.76 | -9.55, 2.41 | 0.228 | 0.969 | -2.92 | -8.52, 3.03 | 0.330 | 0.999 |  |  |  |  |  |  |  |  |  |  |
| c48885 | arabitol/xylitol | -0.89 | -4.91, 3.31 | 0.675 | 0.997 | -0.05 | -4.09, 4.15 | 0.980 | 0.999 |  |  |  |  |  |  |  |  |  |  |
| c48255 | arabonate/xylonate | 1.78 | -1.02, 4.66 | 0.216 | 0.969 | 2.16 | -0.54, 4.94 | 0.121 | 0.999 |  |  |  |  |  |  |  |  |  |  |
|  | **Glycogen Metabolism** |  |  |  |  |  |  |  |  |  |  |  |  |  |  |  |  |  |  |
| c15586 | maltose | 0.54 | -0.50, 1.59 | 0.314 | 0.969 | 0.62 | -0.37, 1.62 | 0.221 | 0.999 |  |  |  |  |  |  |  |  |  |  |
|  | **Disaccharides and Oligosaccharides** |  |  |  |  |  |  |  |  |  |  |  |  |  |  |  |  |  |  |
| c1519 | sucrose | -0.22 | -1.47, 1.05 | 0.736 | 0.997 | -0.03 | -1.24, 1.20 | 0.964 | 0.999 |  |  |  |  |  |  |  |  |  |  |
|  | **Fructose, Mannose and Galactose Metabolism** | |  |  |  |  |  |  |  |  |  |  |  |  |  |  |  |  |  |
| c48195 | fructose | 0.07 | -2.51, 2.72 | 0.959 | 0.999 | -0.14 | -2.59, 2.37 | 0.912 | 0.999 |  |  |  |  |  |  |  |  |  |  |
| c46142 | mannitol/sorbitol | -0.02 | -2.01, 2.01 | 0.983 | 0.999 | -0.26 | -2.17, 1.68 | 0.792 | 0.999 |  |  |  |  |  |  |  |  |  |  |
| c48153 | mannose | -0.28 | -4.18, 3.79 | 0.891 | 0.997 | 0.99 | -2.80, 4.93 | 0.614 | 0.999 |  |  |  |  |  |  |  |  |  |  |
| c27719 | galactonate | 0.15 | -0.66, 0.96 | 0.719 | 0.997 | 0.14 | -0.62, 0.92 | 0.713 | 0.999 |  |  |  |  |  |  |  |  |  |  |
|  | **Aminosugar Metabolism** |  |  |  |  |  |  |  |  |  |  |  |  |  |  |  |  |  |  |
| c15443 | glucuronate | -0.86 | -4.51, 2.93 | 0.652 | 0.997 | 0.01 | -3.54, 3.68 | 0.998 | 0.999 |  |  |  |  |  |  |  |  |  |  |
| c42420 | erythronate* | 1.80 | -1.91, 5.65 | 0.349 | 0.994 | 2.24 | -1.40, 6.01 | 0.234 | 0.999 |  |  |  |  |  |  |  |  |  |  |
|  | **ENERGY** |  |  |  |  |  |  |  |  |  |  |  |  |  |  |  |  |  |  |
|  | **TCA Cycle** |  |  |  |  |  |  |  |  |  |  |  |  |  |  |  |  |  |  |
| c1564 | citrate | 3.38 | -0.39, 7.30 | 0.081 | 0.962 | 2.70 | -0.93, 6.45 | 0.149 | 0.999 |  |  |  |  |  |  |  |  |  |  |
| c528 | alpha-ketoglutarate | -0.14 | -1.06, 0.79 | 0.768 | 0.997 | 0.00 | -0.88, 0.88 | 0.999 | 0.999 |  |  |  |  |  |  |  |  |  |  |
| c37058 | succinylcarnitine (C4-DC) | 0.50 | -3.36, 4.51 | 0.804 | 0.997 | 0.37 | -3.27, 4.14 | 0.846 | 0.999 |  |  |  |  |  |  |  |  |  |  |
| c1437 | succinate | -2.03 | -4.90, 0.92 | 0.178 | 0.962 | -0.98 | -3.75, 1.88 | 0.500 | 0.999 |  |  |  |  |  |  |  |  |  |  |
| c1303 | malate | -0.46 | -3.65, 2.84 | 0.781 | 0.997 | -0.22 | -3.34, 2.99 | 0.890 | 0.999 |  |  |  |  |  |  |  |  |  |  |
| c52282 | 2-methylcitrate/homocitrate | -1.08 | -4.27, 2.21 | 0.516 | 0.997 | -0.80 | -3.86, 2.37 | 0.618 | 0.999 |  |  |  |  |  |  |  |  |  |  |
|  | **Oxidative Phosphorylation** |  |  |  |  |  |  |  |  |  |  |  |  |  |  |  |  |  |  |
| c42109 | phosphate | 0.72 | -1.31, 2.78 | 0.492 | 0.994 | 0.51 | -1.45, 2.51 | 0.614 | 0.999 |  |  |  |  |  |  |  |  |  |  |
|  | **LIPID** |  |  |  |  |  |  |  |  |  |  |  |  |  |  |  |  |  |  |
|  | **Medium Chain Fatty Acid** |  |  |  |  |  |  |  |  |  |  |  |  |  |  |  |  |  |  |
| c32489 | caproate (6:0) | 0.61 | -1.12, 2.36 | 0.494 | 0.994 | -0.03 | -1.73, 1.69 | 0.971 | 0.999 |  |  |  |  |  |  |  |  |  |  |
| c1644 | heptanoate (7:0) | 0.69 | -1.00, 2.41 | 0.429 | 0.994 | 0.12 | -1.53, 1.79 | 0.891 | 0.999 |  |  |  |  |  |  |  |  |  |  |
| c32492 | caprylate (8:0) | 0.32 | -2.73, 3.46 | 0.840 | 0.997 | -0.30 | -3.19, 2.68 | 0.844 | 0.999 |  |  |  |  |  |  |  |  |  |  |
| c1642 | caprate (10:0) | 0.23 | -2.44, 2.98 | 0.865 | 0.997 | 0.02 | -2.50, 2.60 | 0.989 | 0.999 |  |  |  |  |  |  |  |  |  |  |
| c32497 | 10-undecenoate (11:1n1) | -0.17 | -2.56, 2.28 | 0.891 | 0.997 | -0.28 | -2.55, 2.04 | 0.812 | 0.999 |  |  |  |  |  |  |  |  |  |  |
| c1645 | laurate (12:0) | 0.06 | -1.98, 2.14 | 0.953 | 0.997 | 0.34 | -1.62, 2.33 | 0.739 | 0.999 |  |  |  |  |  |  |  |  |  |  |
| c33968 | 5-dodecenoate (12:1n7) | -1.02 | -3.03, 1.03 | 0.327 | 0.990 | -0.78 | -2.72, 1.21 | 0.442 | 0.999 |  |  |  |  |  |  |  |  |  |  |
|  | **Long Chain Saturated Fatty Acid** |  |  |  |  |  |  |  |  |  |  |  |  |  |  |  |  |  |  |
| c1365 | myristate (14:0) | -0.71 | -3.03, 1.67 | 0.559 | 0.997 | -0.05 | -2.32, 2.26 | 0.963 | 0.999 |  |  |  |  |  |  |  |  |  |  |
| c1336 | palmitate (16:0) | -2.07 | -4.91, 0.86 | 0.166 | 0.962 | -0.75 | -3.54, 2.11 | 0.604 | 0.999 |  |  |  |  |  |  |  |  |  |  |
| c1121 | margarate (17:0) | -1.08 | -4.02, 1.95 | 0.482 | 0.994 | -0.38 | -3.20, 2.51 | 0.794 | 0.999 |  |  |  |  |  |  |  |  |  |  |
| c1358 | stearate (18:0) | -0.38 | -4.11, 3.49 | 0.845 | 0.997 | 1.05 | -2.54, 4.78 | 0.572 | 0.999 |  |  |  |  |  |  |  |  |  |  |
|  | **Long Chain Monounsaturated Fatty Acid** |  |  |  |  |  |  |  |  |  |  |  |  |  |  |  |  |  |  |
| c32418 | myristoleate (14:1n5) | -0.33 | -2.22, 1.58 | 0.731 | 0.997 | 0.10 | -1.74, 1.98 | 0.917 | 0.999 |  |  |  |  |  |  |  |  |  |  |
| c33447 | palmitoleate (16:1n7) | -1.27 | -3.08, 0.56 | 0.175 | 0.962 | -0.59 | -2.40, 1.25 | 0.525 | 0.999 |  |  |  |  |  |  |  |  |  |  |
| c33971 | 10-heptadecenoate (17:1n7) | -0.98 | -2.94, 1.02 | 0.338 | 0.994 | -0.36 | -2.28, 1.60 | 0.715 | 0.999 |  |  |  |  |  |  |  |  |  |  |
| c52285 | oleate/vaccenate (18:1) | -1.43 | -3.70, 0.89 | 0.226 | 0.969 | -0.68 | -2.89, 1.58 | 0.555 | 0.999 |  |  |  |  |  |  |  |  |  |  |
| c33972 | 10-nonadecenoate (19:1n9) | -1.10 | -3.40, 1.26 | 0.359 | 0.994 | -0.44 | -2.66, 1.84 | 0.705 | 0.999 |  |  |  |  |  |  |  |  |  |  |
|  | **Long Chain Polyunsaturated Fatty Acid (n3 and n6)** | |  |  |  |  |  |  |  |  |  |  |  |  |  |  |  |  |  |
| c33969 | stearidonate (18:4n3) | 0.13 | -1.26, 1.54 | 0.859 | 0.997 | 0.37 | -0.98, 1.74 | 0.592 | 0.999 |  |  |  |  |  |  |  |  |  |  |
| c18467 | eicosapentaenoate (EPA; 20:5n3) | -0.67 | -2.40, 1.08 | 0.451 | 0.994 | 0.34 | -1.37, 2.08 | 0.699 | 0.999 |  |  |  |  |  |  |  |  |  |  |
| c32504 | docosapentaenoate (DPA; 22:5n3) | -1.54 | -3.59, 0.57 | 0.153 | 0.962 | -0.25 | -2.31, 1.84 | 0.811 | 0.999 |  |  |  |  |  |  |  |  |  |  |
| c44675 | docosahexaenoate (DHA; 22:6n3) | -1.60 | -3.76, 0.60 | 0.155 | 0.962 | -0.95 | -3.04, 1.19 | 0.382 | 0.999 |  |  |  |  |  |  |  |  |  |  |
| c1105 | linoleate (18:2n6) | -1.20 | -3.41, 1.06 | 0.296 | 0.969 | -0.23 | -2.38, 1.96 | 0.834 | 0.999 |  |  |  |  |  |  |  |  |  |  |
| c34035 | linolenate (18:3n3 or 3n6) | -0.79 | -2.77, 1.23 | 0.440 | 0.994 | -0.18 | -2.07, 1.75 | 0.856 | 0.999 |  |  |  |  |  |  |  |  |  |  |
| c17805 | dihomolinoleate (20:2n6) | -1.46 | -3.81, 0.94 | 0.233 | 0.969 | -0.68 | -2.94, 1.64 | 0.564 | 0.999 |  |  |  |  |  |  |  |  |  |  |
| c35718 | dihomolinolenate (20:3n3 or 3n6) | -1.96 | -4.05, 0.16 | 0.072 | 0.962 | -0.77 | -2.86, 1.36 | 0.474 | 0.999 |  |  |  |  |  |  |  |  |  |  |
| c1110 | arachidonate (20:4n6) | -1.12 | -3.26, 1.06 | 0.312 | 0.969 | 0.10 | -1.99, 2.23 | 0.927 | 0.999 |  |  |  |  |  |  |  |  |  |  |
| c37478 | docosapentaenoate (n6 DPA; 22:5n6) | -2.29 | -4.40,-0.14 | 0.039 | 0.962 | -1.35 | -3.44, 0.78 | 0.214 | 0.999 |  |  |  |  |  |  |  |  |  |  |
|  | **Fatty Acid, Branched** |  |  |  |  |  |  |  |  |  |  |  |  |  |  |  |  |  |  |
| c38768 | (14 or 15)-methylpalmitate (a17:0 or i17:0) | 0.10 | -1.62, 1.86 | 0.907 | 0.997 | 0.52 | -1.11, 2.18 | 0.532 | 0.999 |  |  |  |  |  |  |  |  |  |  |
|  | **Fatty Acid, Dicarboxylate** |  |  |  |  |  |  |  |  |  |  |  |  |  |  |  |  |  |  |
| c396 | glutarate (C5-DC) | -0.21 | -1.24, 0.83 | 0.690 | 0.997 | -0.34 | -1.34, 0.66 | 0.503 | 0.999 |  |  |  |  |  |  |  |  |  |  |
| c37253 | 2-hydroxyglutarate | -0.32 | -2.34, 1.75 | 0.770 | 0.997 | -0.50 | -2.46, 1.50 | 0.620 | 0.999 |  |  |  |  |  |  |  |  |  |  |
| c32398 | sebacate (C10-DC) | -0.27 | -1.03, 0.50 | 0.490 | 0.994 | -0.33 | -1.06, 0.41 | 0.382 | 0.999 |  |  |  |  |  |  |  |  |  |  |
| c35678 | hexadecanedioate (C16) | -0.91 | -3.15, 1.38 | 0.433 | 0.994 | -0.54 | -2.67, 1.64 | 0.625 | 0.999 |  |  |  |  |  |  |  |  |  |  |
| c36754 | octadecanedioate (C18) | -0.23 | -2.45, 2.05 | 0.845 | 0.997 | 0.08 | -2.03, 2.23 | 0.941 | 0.999 |  |  |  |  |  |  |  |  |  |  |
| c39831 | eicosanedioate (C20-DC) | 1.25 | -0.83, 3.36 | 0.243 | 0.969 | 0.79 | -1.21, 2.83 | 0.440 | 0.999 |  |  |  |  |  |  |  |  |  |  |
| c31787 | 3-carboxy-4-methyl-5-propyl-2-furanpropanoate (CMPF) | 0.64 | -0.53, 1.82 | 0.289 | 0.969 | 0.46 | -0.66, 1.60 | 0.426 | 0.999 |  |  |  |  |  |  |  |  |  |  |
|  | **Fatty Acid, Amino** |  |  |  |  |  |  |  |  |  |  |  |  |  |  |  |  |  |  |
| c43761 | 2-aminoheptanoate | 0.28 | -2.18, 2.80 | 0.827 | 0.997 | 0.96 | -1.41, 3.39 | 0.429 | 0.999 |  |  |  |  |  |  |  |  |  |  |
| c43343 | 2-aminooctanoate | 0.63 | -1.05, 2.35 | 0.464 | 0.994 | 0.82 | -0.83, 2.50 | 0.332 | 0.999 |  |  |  |  |  |  |  |  |  |  |
|  | **Fatty Acid Metabolism (also BCAA Metabolism)** | |  |  |  |  |  |  |  |  |  |  |  |  |  |  |  |  |  |
| c32412 | butyrylcarnitine (C4) | -0.95 | -2.19, 0.31 | 0.141 | 0.962 | -0.78 | -1.96, 0.40 | 0.196 | 0.999 |  |  |  |  |  |  |  |  |  |  |
| c32452 | propionylcarnitine (C3) | -1.84 | -4.51, 0.90 | 0.187 | 0.962 | -2.04 | -4.54, 0.52 | 0.119 | 0.999 |  |  |  |  |  |  |  |  |  |  |
|  | **Fatty Acid Metabolism (Acyl Glutamine)** |  |  |  |  |  |  |  |  |  |  |  |  |  |  |  |  |  |  |
| c54907 | hexanoylglutamine | -1.03 | -2.21, 0.17 | 0.094 | 0.962 | -1.01 | -2.13, 0.12 | 0.082 | 0.999 |  |  |  |  |  |  |  |  |  |  |
|  | **Fatty Acid Metabolism (Acyl Carnitine, Short Chain)** | |  |  |  |  |  |  |  |  |  |  |  |  |  |  |  |  |  |
| c32198 | acetylcarnitine (C2) | -0.61 | -2.89, 1.73 | 0.607 | 0.997 | -0.72 | -2.87, 1.47 | 0.516 | 0.999 |  |  |  |  |  |  |  |  |  |  |
|  | **Fatty Acid Metabolism (Acyl Carnitine, Medium Chain)** | | |  |  |  |  |  |  |  |  |  |  |  |  |  |  |  |  |
| c32328 | hexanoylcarnitine (C6) | -1.70 | -3.79, 0.44 | 0.120 | 0.962 | -1.78 | -3.77, 0.24 | 0.086 | 0.999 |  |  |  |  |  |  |  |  |  |  |
| c33936 | octanoylcarnitine (C8) | -1.58 | -3.60, 0.48 | 0.134 | 0.962 | -1.82 | -3.74, 0.14 | 0.070 | 0.999 |  |  |  |  |  |  |  |  |  |  |
| c33941 | decanoylcarnitine (C10) | -1.69 | -3.49, 0.15 | 0.074 | 0.962 | -1.92 | -3.63,-0.18 | 0.032 | 0.965 |  |  |  |  |  |  |  |  |  |  |
| c34534 | laurylcarnitine (C12) | -2.06 | -3.97,-0.11 | 0.040 | 0.962 | -1.98 | -3.80,-0.12 | 0.039 | 0.999 |  |  |  |  |  |  |  |  |  |  |
|  | **Fatty Acid Metabolism (Acyl Carnitine, Long Chain Saturated)** | | |  |  |  |  |  |  |  |  |  |  |  |  |  |  |  |  |
| c33952 | myristoylcarnitine (C14) | -2.57 | -5.18, 0.12 | 0.063 | 0.962 | -1.83 | -4.38, 0.79 | 0.172 | 0.999 |  |  |  |  |  |  |  |  |  |  |
| c44681 | palmitoylcarnitine (C16) | -1.47 | -4.40, 1.55 | 0.337 | 0.994 | -1.32 | -4.14, 1.58 | 0.369 | 0.999 |  |  |  |  |  |  |  |  |  |  |
|  | **Fatty Acid Metabolism (Acyl Carnitine, Monounsaturated)** | | |  |  |  |  |  |  |  |  |  |  |  |  |  |  |  |  |
| c38178 | cis-4-decenoylcarnitine (C10:1) | -2.24 | -4.54, 0.11 | 0.064 | 0.962 | -2.24 | -4.44, 0.02 | 0.054 | 0.999 |  |  |  |  |  |  |  |  |  |  |
| c48182 | myristoleoylcarnitine (C14:1)* | -1.62 | -3.52, 0.31 | 0.102 | 0.962 | -1.34 | -3.17, 0.52 | 0.159 | 0.999 |  |  |  |  |  |  |  |  |  |  |
| c53223 | palmitoleoylcarnitine (C16:1)* | -2.16 | -4.69, 0.45 | 0.106 | 0.962 | -1.45 | -3.95, 1.12 | 0.267 | 0.999 |  |  |  |  |  |  |  |  |  |  |
| c35160 | oleoylcarnitine (C18:1) | -1.35 | -4.35, 1.74 | 0.389 | 0.994 | -0.40 | -3.38, 2.66 | 0.795 | 0.999 |  |  |  |  |  |  |  |  |  |  |
|  | **Fatty Acid Metabolism (Acyl Carnitine, Polyunsaturated)** | | |  |  |  |  |  |  |  |  |  |  |  |  |  |  |  |  |
| c46223 | linoleoylcarnitine (C18:2)* | -0.21 | -3.31, 2.99 | 0.896 | 0.997 | 0.42 | -2.62, 3.54 | 0.791 | 0.999 |  |  |  |  |  |  |  |  |  |  |
|  | **Fatty Acid Metabolism (Acyl Carnitine, Dicarboxylate)** | | |  |  |  |  |  |  |  |  |  |  |  |  |  |  |  |  |
| c52988 | adipoylcarnitine (C6-DC) | -1.49 | -3.26, 0.31 | 0.105 | 0.962 | -1.60 | -3.31, 0.14 | 0.073 | 0.999 |  |  |  |  |  |  |  |  |  |  |
| c53224 | pimeloylcarnitine/3-methyladipoylcarnitine (C7-DC) | -0.70 | -2.31, 0.94 | 0.402 | 0.994 | -0.88 | -2.39, 0.66 | 0.263 | 0.999 |  |  |  |  |  |  |  |  |  |  |
|  | **Fatty Acid Metabolism (Acyl Carnitine, Hydroxy)** | |  |  |  |  |  |  |  |  |  |  |  |  |  |  |  |  |  |
| c43264 | (R)-3-hydroxybutyrylcarnitine | -0.10 | -1.19, 1.00 | 0.856 | 0.997 | -0.03 | -1.07, 1.01 | 0.951 | 0.999 |  |  |  |  |  |  |  |  |  |  |
|  | **Carnitine Metabolism** |  |  |  |  |  |  |  |  |  |  |  |  |  |  |  |  |  |  |
| c36747 | deoxycarnitine | -3.76 | -8.98, 1.76 | 0.180 | 0.962 | -4.90 | -9.80, 0.27 | 0.065 | 0.999 |  |  |  |  |  |  |  |  |  |  |
| c15500 | carnitine | -0.22 | -2.87, 2.49 | 0.870 | 0.997 | -1.69 | -4.14, 0.82 | 0.187 | 0.999 |  |  |  |  |  |  |  |  |  |  |
|  | **Fatty Acid Metabolism (Acyl Choline)** |  |  |  |  |  |  |  |  |  |  |  |  |  |  |  |  |  |  |
| c53257 | palmitoloelycholine | -0.66 | -1.60, 0.30 | 0.180 | 0.962 | -0.08 | -1.02, 0.87 | 0.864 | 0.999 |  |  |  |  |  |  |  |  |  |  |
| c53262 | dihomo-linolenoyl-choline | -0.65 | -1.71, 0.43 | 0.238 | 0.969 | -0.08 | -1.14, 0.99 | 0.884 | 0.999 |  |  |  |  |  |  |  |  |  |  |
| c53263 | docosahexaenoylcholine | -0.37 | -1.52, 0.79 | 0.532 | 0.997 | 0.08 | -1.03, 1.21 | 0.889 | 0.999 |  |  |  |  |  |  |  |  |  |  |
|  | **Fatty Acid, Monohydroxy** |  |  |  |  |  |  |  |  |  |  |  |  |  |  |  |  |  |  |
| c22036 | 2-hydroxyoctanoate | 0.21 | -2.32, 2.81 | 0.872 | 0.997 | 0.08 | -2.33, 2.55 | 0.948 | 0.999 |  |  |  |  |  |  |  |  |  |  |
| c42489 | 2-hydroxydecanoate | -1.00 | -4.57, 2.70 | 0.591 | 0.997 | -1.30 | -4.78, 2.32 | 0.478 | 0.999 |  |  |  |  |  |  |  |  |  |  |
| c35675 | 2-hydroxypalmitate | -0.84 | -5.49, 4.04 | 0.732 | 0.997 | 0.78 | -3.80, 5.58 | 0.743 | 0.999 |  |  |  |  |  |  |  |  |  |  |
| c17945 | 2-hydroxystearate | -1.63 | -6.33, 3.32 | 0.513 | 0.997 | -0.42 | -5.02, 4.40 | 0.861 | 0.999 |  |  |  |  |  |  |  |  |  |  |
| c53230 | 3-hydroxyhexanoate | -0.99 | -3.64, 1.73 | 0.473 | 0.994 | -0.95 | -3.46, 1.62 | 0.467 | 0.999 |  |  |  |  |  |  |  |  |  |  |
| c22001 | 3-hydroxyoctanoate | -1.12 | -3.71, 1.54 | 0.406 | 0.994 | -1.11 | -3.60, 1.44 | 0.391 | 0.999 |  |  |  |  |  |  |  |  |  |  |
| c22053 | 3-hydroxydecanoate | -1.27 | -3.47, 0.97 | 0.265 | 0.969 | -1.16 | -3.28, 1.01 | 0.293 | 0.999 |  |  |  |  |  |  |  |  |  |  |
| c32457 | 3-hydroxylaurate | -1.59 | -3.56, 0.41 | 0.121 | 0.962 | -1.35 | -3.24, 0.57 | 0.169 | 0.999 |  |  |  |  |  |  |  |  |  |  |
| c39609 | 16-hydroxypalmitate | -1.60 | -4.60, 1.50 | 0.310 | 0.969 | -0.36 | -3.31, 2.68 | 0.814 | 0.999 |  |  |  |  |  |  |  |  |  |  |
|  | **Fatty Acid, Dihydroxy** |  |  |  |  |  |  |  |  |  |  |  |  |  |  |  |  |  |  |
| c38395 | 12,13-DiHOME | 0.28 | -1.30, 1.90 | 0.728 | 0.997 | 0.40 | -1.10, 1.92 | 0.603 | 0.999 |  |  |  |  |  |  |  |  |  |  |
|  | **Eicosanoid** |  |  |  |  |  |  |  |  |  |  |  |  |  |  |  |  |  |  |
| c37536 | 12-HETE | 0.31 | -0.45, 1.08 | 0.429 | 0.994 | 0.25 | -0.48, 0.99 | 0.502 | 0.999 |  |  |  |  |  |  |  |  |  |  |
|  | **Endocannabinoid** |  |  |  |  |  |  |  |  |  |  |  |  |  |  |  |  |  |  |
| c52608 | linoleoyl ethanolamide | -0.09 | -0.89, 0.71 | 0.825 | 0.997 | -0.07 | -0.83, 0.69 | 0.852 | 0.999 |  |  |  |  |  |  |  |  |  |  |
|  | **Inositol Metabolism** |  |  |  |  |  |  |  |  |  |  |  |  |  |  |  |  |  |  |
| c1124 | myo-inositol | 2.08 | -1.91, 6.23 | 0.313 | 0.969 | 1.75 | -2.03, 5.67 | 0.370 | 0.999 |  |  |  |  |  |  |  |  |  |  |
|  | **Phospholipid Metabolism** |  |  |  |  |  |  |  |  |  |  |  |  |  |  |  |  |  |  |
| c15506 | choline | 0.09 | -2.01, 2.24 | 0.932 | 0.997 | 0.98 | -1.03, 3.03 | 0.341 | 0.999 |  |  |  |  |  |  |  |  |  |  |
| c34396 | phosphocholine | 2.84 | -0.14, 5.90 | 0.064 | 0.962 | 3.11 | 0.29, 6.01 | 0.032 | 0.965 |  |  |  |  |  |  |  |  |  |  |
| c15990 | glycerophosphorylcholine (GPC) | 0.09 | -2.59, 2.84 | 0.950 | 0.997 | 1.12 | -1.42, 3.72 | 0.391 | 0.999 |  |  |  |  |  |  |  |  |  |  |
| c40406 | trimethylamine N-oxide | 0.43 | -1.27, 2.15 | 0.625 | 0.997 | -0.44 | -2.05, 1.19 | 0.592 | 0.999 |  |  |  |  |  |  |  |  |  |  |
|  | **Phosphatidylcholine (PC)** |  |  |  |  |  |  |  |  |  |  |  |  |  |  |  |  |  |  |
| c19130 | 1,2-dipalmitoyl-GPC (16:0/16:0) | 2.45 | -4.23, 9.61 | 0.482 | 0.994 | 0.71 | -5.65, 7.50 | 0.832 | 0.999 |  |  |  |  |  |  |  |  |  |  |
| c52470 | 1-palmitoyl-2-palmitoleoyl-GPC (16:0/16:1)* | -0.81 | -2.95, 1.37 | 0.463 | 0.994 | -0.93 | -2.93, 1.12 | 0.373 | 0.999 |  |  |  |  |  |  |  |  |  |  |
| c52461 | 1-palmitoyl-2-oleoyl-GPC (16:0/18:1) | -0.20 | -5.51, 5.40 | 0.942 | 0.997 | -0.51 | -5.54, 4.78 | 0.846 | 0.999 |  |  |  |  |  |  |  |  |  |  |
| c42446 | 1-palmitoyl-2-linoleoyl-GPC (16:0/18:2) | 1.97 | -3.59, 7.86 | 0.496 | 0.994 | 0.12 | -5.09, 5.60 | 0.966 | 0.999 |  |  |  |  |  |  |  |  |  |  |
| c52462 | 1-palmitoyl-2-arachidonoyl-GPC (16:0/20:4n6) | 2.06 | -3.13, 7.53 | 0.445 | 0.994 | 0.94 | -3.91, 6.04 | 0.710 | 0.999 |  |  |  |  |  |  |  |  |  |  |
| c52438 | 1-stearoyl-2-oleoyl-GPC (18:0/18:1) | -1.24 | -4.75, 2.40 | 0.500 | 0.995 | -0.74 | -4.11, 2.75 | 0.675 | 0.999 |  |  |  |  |  |  |  |  |  |  |
| c52452 | 1-stearoyl-2-linoleoyl-GPC (18:0/18:2)* | -0.23 | -6.31, 6.25 | 0.943 | 0.997 | -1.28 | -6.97, 4.76 | 0.671 | 0.999 |  |  |  |  |  |  |  |  |  |  |
| c42450 | 1-stearoyl-2-arachidonoyl-GPC (18:0/20:4) | 1.56 | -4.16, 7.63 | 0.601 | 0.997 | 0.79 | -4.56, 6.44 | 0.777 | 0.999 |  |  |  |  |  |  |  |  |  |  |
| c52453 | 1-oleoyl-2-linoleoyl-GPC (18:1/18:2)* | -3.41 | -8.51, 1.98 | 0.212 | 0.969 | -3.01 | -7.84, 2.07 | 0.242 | 0.999 |  |  |  |  |  |  |  |  |  |  |
| c52603 | 1,2-dilinoleoyl-GPC (18:2/18:2) | 1.79 | -1.52, 5.21 | 0.295 | 0.969 | 0.83 | -2.32, 4.08 | 0.611 | 0.999 |  |  |  |  |  |  |  |  |  |  |
| c53176 | 1-linoleoyl-2-linolenoyl-GPC (18:2/18:3)* | 0.41 | -1.58, 2.44 | 0.688 | 0.997 | -0.08 | -1.95, 1.83 | 0.937 | 0.999 |  |  |  |  |  |  |  |  |  |  |
| c52710 | 1-linoleoyl-2-arachidonoyl-GPC (18:2/20:4n6)* | -0.31 | -3.44, 2.91 | 0.847 | 0.997 | -0.16 | -3.12, 2.88 | 0.915 | 0.999 |  |  |  |  |  |  |  |  |  |  |
|  | **Phosphatidylethanolamine (PE)** |  |  |  |  |  |  |  |  |  |  |  |  |  |  |  |  |  |  |
| c19263 | 1-palmitoyl-2-oleoyl-GPE (16:0/18:1) | -1.95 | -3.80,-0.05 | 0.046 | 0.962 | -1.36 | -3.16, 0.48 | 0.148 | 0.999 |  |  |  |  |  |  |  |  |  |  |
| c42449 | 1-palmitoyl-2-linoleoyl-GPE (16:0/18:2) | -1.77 | -3.91, 0.41 | 0.112 | 0.962 | -1.64 | -3.65, 0.42 | 0.119 | 0.999 |  |  |  |  |  |  |  |  |  |  |
| c52464 | 1-palmitoyl-2-arachidonoyl-GPE (16:0/20:4)* | -0.62 | -2.81, 1.63 | 0.587 | 0.997 | -0.51 | -2.58, 1.60 | 0.633 | 0.999 |  |  |  |  |  |  |  |  |  |  |
| c42448 | 1-stearoyl-2-oleoyl-GPE (18:0/18:1) | -1.28 | -3.49, 0.97 | 0.263 | 0.969 | -0.79 | -2.90, 1.36 | 0.470 | 0.999 |  |  |  |  |  |  |  |  |  |  |
| c52446 | 1-stearoyl-2-linoleoyl-GPE (18:0/18:2)* | -2.04 | -4.35, 0.33 | 0.093 | 0.962 | -1.65 | -3.86, 0.62 | 0.154 | 0.999 |  |  |  |  |  |  |  |  |  |  |
| c52447 | 1-stearoyl-2-arachidonoyl-GPE (18:0/20:4) | -0.55 | -3.05, 2.02 | 0.672 | 0.997 | -0.21 | -2.58, 2.22 | 0.864 | 0.999 |  |  |  |  |  |  |  |  |  |  |
|  | **Phosphatidylinositol (PI)** |  |  |  |  |  |  |  |  |  |  |  |  |  |  |  |  |  |  |
| c52669 | 1-palmitoyl-2-oleoyl-GPI (16:0/18:1)* | -0.92 | -3.73, 1.98 | 0.532 | 0.997 | -0.20 | -2.95, 2.63 | 0.888 | 0.999 |  |  |  |  |  |  |  |  |  |  |
| c52450 | 1-palmitoyl-2-linoleoyl-GPI (16:0/18:2) | -0.62 | -3.64, 2.50 | 0.695 | 0.997 | 0.31 | -2.66, 3.38 | 0.838 | 0.999 |  |  |  |  |  |  |  |  |  |  |
| c52726 | 1-stearoyl-2-oleoyl-GPI (18:0/18:1)* | -0.46 | -3.13, 2.28 | 0.740 | 0.997 | 0.09 | -2.47, 2.72 | 0.945 | 0.999 |  |  |  |  |  |  |  |  |  |  |
| c52468 | 1-stearoyl-2-linoleoyl-GPI (18:0/18:2) | 0.52 | -3.18, 4.37 | 0.786 | 0.997 | 1.41 | -2.26, 5.20 | 0.458 | 0.999 |  |  |  |  |  |  |  |  |  |  |
| c52449 | 1-stearoyl-2-arachidonoyl-GPI (18:0/20:4) | -1.81 | -5.68, 2.22 | 0.375 | 0.994 | -0.58 | -4.36, 3.36 | 0.771 | 0.999 |  |  |  |  |  |  |  |  |  |  |
|  | **Lysophospholipid** |  |  |  |  |  |  |  |  |  |  |  |  |  |  |  |  |  |  |
| c33955 | 1-palmitoyl-GPC (16:0) | 0.21 | -2.81, 3.32 | 0.893 | 0.997 | 0.86 | -2.05, 3.85 | 0.568 | 0.999 |  |  |  |  |  |  |  |  |  |  |
| c33230 | 1-palmitoleoyl-GPC* (16:1)* | -0.20 | -2.55, 2.19 | 0.866 | 0.997 | 0.57 | -1.75, 2.94 | 0.635 | 0.999 |  |  |  |  |  |  |  |  |  |  |
| c47118 | 2-palmitoleoyl-GPC* (16:1)* | -0.78 | -2.04, 0.48 | 0.225 | 0.969 | -0.62 | -1.82, 0.59 | 0.317 | 0.999 |  |  |  |  |  |  |  |  |  |  |
| c33961 | 1-stearoyl-GPC (18:0) | 0.09 | -2.71, 2.97 | 0.951 | 0.997 | 0.87 | -1.82, 3.63 | 0.531 | 0.999 |  |  |  |  |  |  |  |  |  |  |
| c48258 | 1-oleoyl-GPC (18:1) | 0.02 | -2.65, 2.77 | 0.987 | 0.999 | 0.93 | -1.64, 3.58 | 0.482 | 0.999 |  |  |  |  |  |  |  |  |  |  |
| c34419 | 1-linoleoyl-GPC (18:2) | 0.92 | -3.45, 5.49 | 0.685 | 0.997 | 1.07 | -3.17, 5.48 | 0.628 | 0.999 |  |  |  |  |  |  |  |  |  |  |
| c45951 | 1-linolenoyl-GPC (18:3)* | 0.61 | -2.00, 3.29 | 0.651 | 0.997 | 0.73 | -1.80, 3.32 | 0.576 | 0.999 |  |  |  |  |  |  |  |  |  |  |
| c33228 | 1-arachidonoyl-GPC* (20:4)* | 0.45 | -2.73, 3.74 | 0.784 | 0.997 | 1.19 | -1.84, 4.30 | 0.448 | 0.999 |  |  |  |  |  |  |  |  |  |  |
| c49617 | 1-lignoceroyl-GPC (24:0) | -1.51 | -4.55, 1.64 | 0.346 | 0.994 | -0.29 | -3.33, 2.85 | 0.856 | 0.999 |  |  |  |  |  |  |  |  |  |  |
| c35631 | 1-palmitoyl-GPE (16:0) | -1.68 | -4.13, 0.84 | 0.191 | 0.962 | -0.94 | -3.33, 1.52 | 0.452 | 0.999 |  |  |  |  |  |  |  |  |  |  |
| c42398 | 1-stearoyl-GPE (18:0) | -0.50 | -2.92, 1.98 | 0.688 | 0.997 | 0.39 | -1.97, 2.80 | 0.750 | 0.999 |  |  |  |  |  |  |  |  |  |  |
| c35628 | 1-oleoyl-GPE (18:1) | -0.09 | -1.98, 1.83 | 0.924 | 0.997 | 0.37 | -1.45, 2.24 | 0.691 | 0.999 |  |  |  |  |  |  |  |  |  |  |
| c36600 | 1-linoleoyl-GPE (18:2)* | -0.16 | -2.66, 2.41 | 0.903 | 0.997 | -0.37 | -2.74, 2.06 | 0.762 | 0.999 |  |  |  |  |  |  |  |  |  |  |
| c35186 | 1-arachidonoyl-GPE (20:4n6)* | 0.71 | -2.52, 4.05 | 0.671 | 0.997 | 0.70 | -2.34, 3.84 | 0.656 | 0.999 |  |  |  |  |  |  |  |  |  |  |
| c19324 | 1-stearoyl-GPI (18:0) | -0.56 | -2.93, 1.86 | 0.646 | 0.997 | 0.21 | -2.08, 2.55 | 0.859 | 0.999 |  |  |  |  |  |  |  |  |  |  |
| c36594 | 1-linoleoyl-GPI* (18:2)* | -0.53 | -3.88, 2.93 | 0.760 | 0.997 | -0.42 | -3.62, 2.88 | 0.799 | 0.999 |  |  |  |  |  |  |  |  |  |  |
| c34214 | 1-arachidonoyl-GPI* (20:4)* | -2.21 | -5.33, 1.02 | 0.180 | 0.962 | -1.02 | -4.08, 2.14 | 0.525 | 0.999 |  |  |  |  |  |  |  |  |  |  |
|  | **Plasmalogen** |  |  |  |  |  |  |  |  |  |  |  |  |  |  |  |  |  |  |
| c52477 | 1-(1-enyl-palmitoyl)-2-oleoyl-GPE (P-16:0/18:1)* | 0.28 | -2.45, 3.09 | 0.842 | 0.997 | -0.49 | -3.14, 2.22 | 0.720 | 0.999 |  |  |  |  |  |  |  |  |  |  |
| c52677 | 1-(1-enyl-palmitoyl)-2-linoleoyl-GPE (P-16:0/18:2)* | 0.57 | -2.12, 3.34 | 0.680 | 0.997 | -0.27 | -2.87, 2.39 | 0.840 | 0.999 |  |  |  |  |  |  |  |  |  |  |
| c52716 | 1-(1-enyl-palmitoyl)-2-palmitoyl-GPC (P-16:0/16:0)* | 2.68 | -1.70, 7.26 | 0.235 | 0.969 | -0.02 | -4.20, 4.33 | 0.991 | 0.999 |  |  |  |  |  |  |  |  |  |  |
| c52713 | 1-(1-enyl-palmitoyl)-2-palmitoleoyl-GPC (P-16:0/16:1)* | 2.39 | -0.77, 5.66 | 0.141 | 0.962 | 0.61 | -2.38, 3.68 | 0.695 | 0.999 |  |  |  |  |  |  |  |  |  |  |
| c52673 | 1-(1-enyl-palmitoyl)-2-arachidonoyl-GPE (P-16:0/20:4)* | 1.43 | -1.17, 4.10 | 0.286 | 0.969 | 0.50 | -2.03, 3.09 | 0.702 | 0.999 |  |  |  |  |  |  |  |  |  |  |
| c52478 | 1-(1-enyl-palmitoyl)-2-oleoyl-GPC (P-16:0/18:1)* | 4.48 | -0.22, 9.41 | 0.064 | 0.962 | 2.14 | -2.41, 6.89 | 0.364 | 0.999 |  |  |  |  |  |  |  |  |  |  |
| c52614 | 1-(1-enyl-stearoyl)-2-oleoyl-GPE (P-18:0/18:1) | 0.92 | -1.86, 3.79 | 0.520 | 0.997 | 0.10 | -2.56, 2.83 | 0.942 | 0.999 |  |  |  |  |  |  |  |  |  |  |
| c52748 | 1-(1-enyl-stearoyl)-2-linoleoyl-GPE (P-18:0/18:2)* | 0.99 | -1.59, 3.63 | 0.457 | 0.994 | 0.32 | -2.17, 2.88 | 0.803 | 0.999 |  |  |  |  |  |  |  |  |  |  |
| c52689 | 1-(1-enyl-palmitoyl)-2-arachidonoyl-GPC (P-16:0/20:4)* | 2.41 | -0.91, 5.84 | 0.160 | 0.962 | 1.03 | -2.16, 4.32 | 0.533 | 0.999 |  |  |  |  |  |  |  |  |  |  |
| c52682 | 1-(1-enyl-palmitoyl)-2-linoleoyl-GPC (P-16:0/18:2)* | 4.15 | -0.25, 8.73 | 0.066 | 0.962 | 1.70 | -2.58, 6.16 | 0.444 | 0.999 |  |  |  |  |  |  |  |  |  |  |
| c52475 | 1-(1-enyl-stearoyl)-2-arachidonoyl-GPE (P-18:0/20:4)* | 1.06 | -1.41, 3.58 | 0.407 | 0.994 | 0.06 | -2.32, 2.49 | 0.964 | 0.999 |  |  |  |  |  |  |  |  |  |  |
|  | **Lysoplasmalogen** |  |  |  |  |  |  |  |  |  |  |  |  |  |  |  |  |  |  |
| c39270 | 1-(1-enyl-palmitoyl)-GPE (P-16:0)* | 0.42 | -1.60, 2.48 | 0.688 | 0.997 | 0.39 | -1.51, 2.33 | 0.688 | 0.999 |  |  |  |  |  |  |  |  |  |  |
| c39271 | 1-(1-enyl-stearoyl)-GPE (P-18:0)* | 0.50 | -1.65, 2.70 | 0.650 | 0.997 | 0.46 | -1.56, 2.53 | 0.656 | 0.999 |  |  |  |  |  |  |  |  |  |  |
|  | **Glycerolipid Metabolism** |  |  |  |  |  |  |  |  |  |  |  |  |  |  |  |  |  |  |
| c15122 | glycerol | -0.79 | -2.81, 1.28 | 0.453 | 0.994 | -0.19 | -2.15, 1.81 | 0.852 | 0.999 |  |  |  |  |  |  |  |  |  |  |
|  | **Monoacylglycerol** |  |  |  |  |  |  |  |  |  |  |  |  |  |  |  |  |  |  |
| c21184 | 1-oleoylglycerol (18:1) | -2.10 | -3.99,-0.18 | 0.034 | 0.962 | -1.47 | -3.34, 0.44 | 0.133 | 0.999 |  |  |  |  |  |  |  |  |  |  |
|  | **Diacylglycerol** |  |  |  |  |  |  |  |  |  |  |  |  |  |  |  |  |  |  |
| c46799 | oleoyl-linoleoyl-glycerol (18:1/18:2) [2] | -1.52 | -3.13, 0.12 | 0.071 | 0.962 | -0.89 | -2.49, 0.73 | 0.280 | 0.999 |  |  |  |  |  |  |  |  |  |  |
|  | **Sphingolipid Synthesis** |  |  |  |  |  |  |  |  |  |  |  |  |  |  |  |  |  |  |
| c52605 | sphinganine-1-phosphate | 0.30 | -1.53, 2.17 | 0.751 | 0.997 | -0.44 | -2.15, 1.30 | 0.619 | 0.999 |  |  |  |  |  |  |  |  |  |  |
|  | **Ceramides** |  |  |  |  |  |  |  |  |  |  |  |  |  |  |  |  |  |  |
| c44877 | N-palmitoyl-sphingosine (d18:1/16:0) | -4.49 | -9.30, 0.58 | 0.083 | 0.962 | -3.96 | -8.53, 0.83 | 0.106 | 0.999 |  |  |  |  |  |  |  |  |  |  |
| c54979 | N-stearoyl-sphingosine (d18:1/18:0)* | -1.81 | -4.77, 1.23 | 0.242 | 0.969 | -2.07 | -4.84, 0.79 | 0.156 | 0.999 |  |  |  |  |  |  |  |  |  |  |
|  | **Hexosylceramides (HCER)** |  |  |  |  |  |  |  |  |  |  |  |  |  |  |  |  |  |  |
| c53013 | glycosyl-N-palmitoyl-sphingosine (d18:1/16:0) | 1.53 | -3.27, 6.58 | 0.539 | 0.997 | 1.24 | -3.27, 5.95 | 0.597 | 0.999 |  |  |  |  |  |  |  |  |  |  |
| c52234 | glycosyl-N-stearoyl-sphingosine (d18:1/18:0) | -0.56 | -4.32, 3.36 | 0.777 | 0.997 | -0.25 | -3.87, 3.49 | 0.892 | 0.999 |  |  |  |  |  |  |  |  |  |  |
|  | **Lactosylceramides (LCER)** |  |  |  |  |  |  |  |  |  |  |  |  |  |  |  |  |  |  |
| c57370 | lactosyl-N-nervonoyl-sphingosine (d18:1/24:1)* | -0.90 | -4.86, 3.22 | 0.664 | 0.997 | -0.94 | -4.68, 2.93 | 0.628 | 0.999 |  |  |  |  |  |  |  |  |  |  |
|  | **Dihydrosphingomyelins** |  |  |  |  |  |  |  |  |  |  |  |  |  |  |  |  |  |  |
| c57365 | myristoyl dihydrosphingomyelin (d18:0/14:0)* | -2.70 | -6.31, 1.04 | 0.157 | 0.962 | -1.74 | -5.28, 1.93 | 0.349 | 0.999 |  |  |  |  |  |  |  |  |  |  |
| c52434 | palmitoyl dihydrosphingomyelin (d18:0/16:0)* | 2.00 | -3.66, 8.00 | 0.497 | 0.994 | 1.25 | -4.09, 6.90 | 0.653 | 0.999 |  |  |  |  |  |  |  |  |  |  |
| c57331 | behenoyl dihydrosphingomyelin (d18:0/22:0)* | -2.15 | -4.77, 0.55 | 0.120 | 0.962 | -1.79 | -4.31, 0.81 | 0.177 | 0.999 |  |  |  |  |  |  |  |  |  |  |
|  | **Sphingomyelins** |  |  |  |  |  |  |  |  |  |  |  |  |  |  |  |  |  |  |
| c37506 | palmitoyl sphingomyelin (d18:1/16:0) | 6.12 | -1.51,14.35 | 0.121 | 0.962 | 4.63 | -2.54,12.33 | 0.213 | 0.999 |  |  |  |  |  |  |  |  |  |  |
| c19503 | stearoyl sphingomyelin (d18:1/18:0) | 2.69 | -2.43, 8.08 | 0.311 | 0.969 | 1.36 | -3.55, 6.52 | 0.595 | 0.999 |  |  |  |  |  |  |  |  |  |  |
| c48492 | behenoyl sphingomyelin (d18:1/22:0)* | -1.86 | -7.87, 4.53 | 0.560 | 0.997 | -1.15 | -6.88, 4.94 | 0.705 | 0.999 |  |  |  |  |  |  |  |  |  |  |
| c52436 | tricosanoyl sphingomyelin (d18:1/23:0)* | -0.90 | -6.07, 4.56 | 0.742 | 0.997 | -0.65 | -5.62, 4.58 | 0.805 | 0.999 |  |  |  |  |  |  |  |  |  |  |
| c57330 | lignoceroyl sphingomyelin (d18:1/24:0) | -0.15 | -4.91, 4.85 | 0.952 | 0.997 | 0.00 | -4.54, 4.75 | 0.999 | 0.999 |  |  |  |  |  |  |  |  |  |  |
| c42463 | sphingomyelin (d18:1/14:0, d16:1/16:0)* | -0.57 | -5.60, 4.73 | 0.830 | 0.997 | -0.64 | -5.43, 4.39 | 0.800 | 0.999 |  |  |  |  |  |  |  |  |  |  |
| c47154 | sphingomyelin (d18:2/14:0, d18:1/14:1)* | -3.07 | -7.00, 1.03 | 0.142 | 0.962 | -1.60 | -5.60, 2.56 | 0.445 | 0.999 |  |  |  |  |  |  |  |  |  |  |
| c52433 | sphingomyelin (d17:1/16:0, d18:1/15:0, d16:1/17:0)* | 1.38 | -4.00, 7.06 | 0.623 | 0.997 | 0.86 | -4.25, 6.24 | 0.748 | 0.999 |  |  |  |  |  |  |  |  |  |  |
| c42459 | sphingomyelin (d18:2/16:0, d18:1/16:1)* | 0.56 | -6.30, 7.91 | 0.878 | 0.997 | -0.44 | -6.99, 6.56 | 0.898 | 0.999 |  |  |  |  |  |  |  |  |  |  |
| c52615 | sphingomyelin (d18:1/17:0, d17:1/18:0, d19:1/16:0) | 2.88 | -2.51, 8.56 | 0.303 | 0.969 | 0.89 | -4.22, 6.28 | 0.738 | 0.999 |  |  |  |  |  |  |  |  |  |  |
| c37529 | sphingomyelin (d18:1/18:1, d18:2/18:0) | 2.31 | -2.99, 7.90 | 0.402 | 0.994 | 1.05 | -4.02, 6.40 | 0.691 | 0.999 |  |  |  |  |  |  |  |  |  |  |
| c48490 | sphingomyelin (d18:1/20:0, d16:1/22:0)* | -1.20 | -5.98, 3.82 | 0.633 | 0.997 | -1.85 | -6.31, 2.82 | 0.432 | 0.999 |  |  |  |  |  |  |  |  |  |  |
| c48491 | sphingomyelin (d18:1/20:1, d18:2/20:0)* | 3.34 | -2.28, 9.28 | 0.251 | 0.969 | 2.46 | -2.89, 8.11 | 0.375 | 0.999 |  |  |  |  |  |  |  |  |  |  |
| c52495 | sphingomyelin (d18:1/21:0, d17:1/22:0, d16:1/23:0)* | -1.87 | -5.17, 1.55 | 0.282 | 0.969 | -1.83 | -4.99, 1.44 | 0.270 | 0.999 |  |  |  |  |  |  |  |  |  |  |
| c48493 | sphingomyelin (d18:1/22:1, d18:2/22:0, d16:1/24:1)* | 1.72 | -3.95, 7.72 | 0.560 | 0.997 | 1.48 | -3.96, 7.23 | 0.601 | 0.999 |  |  |  |  |  |  |  |  |  |  |
| c52435 | sphingomyelin (d18:2/23:0, d18:1/23:1, d17:1/24:1)* | -1.71 | -5.82, 2.57 | 0.429 | 0.994 | -2.07 | -6.02, 2.06 | 0.323 | 0.999 |  |  |  |  |  |  |  |  |  |  |
| c47153 | sphingomyelin (d18:1/24:1, d18:2/24:0)* | 0.65 | -6.55, 8.41 | 0.864 | 0.997 | -0.29 | -7.09, 7.02 | 0.937 | 0.999 |  |  |  |  |  |  |  |  |  |  |
|  | **Sphingosines** |  |  |  |  |  |  |  |  |  |  |  |  |  |  |  |  |  |  |
| c34445 | sphingosine 1-phosphate | 0.07 | -4.12, 4.45 | 0.973 | 0.999 | -1.22 | -5.11, 2.82 | 0.549 | 0.999 |  |  |  |  |  |  |  |  |  |  |
|  | **Mevalonate Metabolism** |  |  |  |  |  |  |  |  |  |  |  |  |  |  |  |  |  |  |
| c531 | 3-hydroxy-3-methylglutarate | -1.88 | -5.02, 1.36 | 0.255 | 0.969 | -1.07 | -4.11, 2.07 | 0.502 | 0.999 |  |  |  |  |  |  |  |  |  |  |
|  | **Sterol** |  |  |  |  |  |  |  |  |  |  |  |  |  |  |  |  |  |  |
| c63 | cholesterol | -0.95 | -4.62, 2.87 | 0.622 | 0.997 | -1.75 | -5.18, 1.81 | 0.332 | 0.999 |  |  |  |  |  |  |  |  |  |  |
| c36776 | 7-HOCA | -1.34 | -5.01, 2.47 | 0.486 | 0.994 | -1.14 | -4.64, 2.50 | 0.536 | 0.999 |  |  |  |  |  |  |  |  |  |  |
| c36803 | 3beta,7alpha-dihydroxy-5-cholestenoate | -0.72 | -3.59, 2.24 | 0.630 | 0.997 | -0.99 | -3.70, 1.80 | 0.485 | 0.999 |  |  |  |  |  |  |  |  |  |  |
| c54805 | 3beta-hydroxy-5-cholestenoate | 1.08 | -2.28, 4.56 | 0.534 | 0.997 | 1.10 | -2.06, 4.37 | 0.499 | 0.999 |  |  |  |  |  |  |  |  |  |  |
|  | **Pregnenolone Steroids** |  |  |  |  |  |  |  |  |  |  |  |  |  |  |  |  |  |  |
| c38170 | pregnenolone sulfate | 0.35 | -1.33, 2.05 | 0.689 | 0.997 | -0.10 | -1.71, 1.53 | 0.901 | 0.999 |  |  |  |  |  |  |  |  |  |  |
| c46115 | 21-hydroxypregnenolone disulfate | 0.11 | -2.08, 2.35 | 0.920 | 0.997 | 0.13 | -1.95, 2.25 | 0.904 | 0.999 |  |  |  |  |  |  |  |  |  |  |
| c32619 | pregnenediol sulfate (C21H34O5S)* | 0.98 | -1.18, 3.18 | 0.379 | 0.994 | 0.62 | -1.43, 2.71 | 0.559 | 0.999 |  |  |  |  |  |  |  |  |  |  |
| c32562 | pregnen-diol disulfate* | 0.06 | -1.74, 1.89 | 0.950 | 0.997 | -0.07 | -1.76, 1.66 | 0.940 | 0.999 |  |  |  |  |  |  |  |  |  |  |
|  | **Progestin Steroids** |  |  |  |  |  |  |  |  |  |  |  |  |  |  |  |  |  |  |
| c37196 | 5alpha-pregnan-3beta,20beta-diol monosulfate (1) | 0.43 | -0.95, 1.82 | 0.547 | 0.997 | 0.21 | -1.14, 1.59 | 0.758 | 0.999 |  |  |  |  |  |  |  |  |  |  |
| c37200 | 5alpha-pregnan-3beta,20alpha-diol monosulfate (2) | 0.22 | -0.99, 1.45 | 0.721 | 0.997 | -0.12 | -1.32, 1.09 | 0.840 | 0.999 |  |  |  |  |  |  |  |  |  |  |
| c37198 | 5alpha-pregnan-3beta,20alpha-diol disulfate | -0.20 | -1.54, 1.15 | 0.766 | 0.997 | -0.51 | -1.82, 0.81 | 0.446 | 0.999 |  |  |  |  |  |  |  |  |  |  |
| c46172 | 5alpha-pregnan-diol disulfate | 0.04 | -1.03, 1.11 | 0.947 | 0.997 | -0.09 | -1.12, 0.96 | 0.872 | 0.999 |  |  |  |  |  |  |  |  |  |  |
| c40708 | pregnanediol-3-glucuronide | 0.28 | -1.02, 1.59 | 0.676 | 0.997 | 0.11 | -1.15, 1.39 | 0.863 | 0.999 |  |  |  |  |  |  |  |  |  |  |
|  | **Corticosteroids** |  |  |  |  |  |  |  |  |  |  |  |  |  |  |  |  |  |  |
| c1712 | cortisol | 0.04 | -2.26, 2.40 | 0.972 | 0.999 | -0.25 | -2.49, 2.04 | 0.829 | 0.999 |  |  |  |  |  |  |  |  |  |  |
| c1769 | cortisone | -0.02 | -3.50, 3.59 | 0.992 | 0.999 | -1.17 | -4.47, 2.24 | 0.497 | 0.999 |  |  |  |  |  |  |  |  |  |  |
|  | **Androgenic Steroids** |  |  |  |  |  |  |  |  |  |  |  |  |  |  |  |  |  |  |
| c32425 | dehydroepiandrosterone sulfate (DHEA-S) | 1.21 | -0.69, 3.16 | 0.216 | 0.969 | 0.88 | -0.94, 2.75 | 0.347 | 0.999 |  |  |  |  |  |  |  |  |  |  |
| c38168 | 16a-hydroxy DHEA 3-sulfate | -0.12 | -1.16, 0.93 | 0.826 | 0.997 | -0.14 | -1.14, 0.87 | 0.785 | 0.999 |  |  |  |  |  |  |  |  |  |  |
| c33973 | epiandrosterone sulfate | 1.29 | -0.05, 2.65 | 0.062 | 0.962 | 0.95 | -0.33, 2.25 | 0.148 | 0.999 |  |  |  |  |  |  |  |  |  |  |
| c31591 | androsterone sulfate | 1.08 | -0.20, 2.38 | 0.100 | 0.962 | 0.79 | -0.43, 2.03 | 0.206 | 0.999 |  |  |  |  |  |  |  |  |  |  |
| c47112 | etiocholanolone glucuronide | 0.48 | -0.70, 1.67 | 0.429 | 0.994 | 0.24 | -0.89, 1.38 | 0.679 | 0.999 |  |  |  |  |  |  |  |  |  |  |
| c37211 | androstenediol (3beta,17beta) monosulfate (1) | 1.04 | -0.50, 2.60 | 0.188 | 0.962 | 0.77 | -0.72, 2.28 | 0.314 | 0.999 |  |  |  |  |  |  |  |  |  |  |
| c37210 | androstenediol (3beta,17beta) monosulfate (2) | -0.34 | -1.86, 1.20 | 0.661 | 0.997 | -0.07 | -1.52, 1.39 | 0.920 | 0.999 |  |  |  |  |  |  |  |  |  |  |
| c37202 | androstenediol (3beta,17beta) disulfate (1) | 0.08 | -1.47, 1.66 | 0.917 | 0.997 | -0.04 | -1.51, 1.44 | 0.955 | 0.999 |  |  |  |  |  |  |  |  |  |  |
| c37203 | androstenediol (3beta,17beta) disulfate (2) | 0.02 | -2.06, 2.15 | 0.983 | 0.999 | 0.17 | -1.79, 2.17 | 0.863 | 0.999 |  |  |  |  |  |  |  |  |  |  |
| c37207 | androstenediol (3alpha, 17alpha) monosulfate (2) | 0.32 | -1.45, 2.12 | 0.726 | 0.997 | 0.54 | -1.13, 2.24 | 0.531 | 0.999 |  |  |  |  |  |  |  |  |  |  |
| c37209 | androstenediol (3alpha, 17alpha) monosulfate (3) | 1.23 | -0.50, 3.00 | 0.165 | 0.962 | 0.87 | -0.77, 2.54 | 0.300 | 0.999 |  |  |  |  |  |  |  |  |  |  |
| c37186 | 5alpha-androstan-3alpha,17beta-diol monosulfate (1) | 1.26 | 0.15, 2.38 | 0.028 | 0.962 | 0.99 | -0.08, 2.06 | 0.071 | 0.999 |  |  |  |  |  |  |  |  |  |  |
| c37190 | 5alpha-androstan-3beta,17beta-diol disulfate | 0.39 | -0.91, 1.71 | 0.560 | 0.997 | 0.20 | -1.03, 1.44 | 0.754 | 0.999 |  |  |  |  |  |  |  |  |  |  |
| c32827 | andro steroid monosulfate C19H28O6S (1)* | -0.34 | -1.61, 0.95 | 0.604 | 0.997 | -0.15 | -1.37, 1.09 | 0.815 | 0.999 |  |  |  |  |  |  |  |  |  |  |
|  | **Primary Bile Acid Metabolism** |  |  |  |  |  |  |  |  |  |  |  |  |  |  |  |  |  |  |
| c22842 | cholate | 0.49 | -0.47, 1.45 | 0.320 | 0.982 | 0.31 | -0.61, 1.23 | 0.511 | 0.999 |  |  |  |  |  |  |  |  |  |  |
| c18476 | glycocholate | 0.46 | -0.54, 1.48 | 0.371 | 0.994 | 0.10 | -0.88, 1.09 | 0.846 | 0.999 |  |  |  |  |  |  |  |  |  |  |
| c1563 | chenodeoxycholate | 0.22 | -0.75, 1.20 | 0.658 | 0.997 | -0.07 | -1.00, 0.87 | 0.888 | 0.999 |  |  |  |  |  |  |  |  |  |  |
| c32346 | glycochenodeoxycholate | 0.71 | -0.36, 1.79 | 0.194 | 0.962 | 0.45 | -0.59, 1.49 | 0.398 | 0.999 |  |  |  |  |  |  |  |  |  |  |
| c18494 | taurochenodeoxycholate | 0.13 | -0.78, 1.06 | 0.780 | 0.997 | -0.35 | -1.24, 0.55 | 0.448 | 0.999 |  |  |  |  |  |  |  |  |  |  |
| c52983 | glycochenodeoxycholate glucuronide (1) | -0.04 | -1.17, 1.11 | 0.947 | 0.997 | -0.29 | -1.38, 0.81 | 0.603 | 0.999 |  |  |  |  |  |  |  |  |  |  |
| c52974 | glycochenodeoxycholate 3-sulfate | -0.86 | -2.03, 0.32 | 0.154 | 0.962 | -1.01 | -2.10, 0.10 | 0.076 | 0.999 |  |  |  |  |  |  |  |  |  |  |
|  | **Secondary Bile Acid Metabolism** |  |  |  |  |  |  |  |  |  |  |  |  |  |  |  |  |  |  |
| c32620 | glycolithocholate sulfate* | -0.48 | -1.64, 0.69 | 0.418 | 0.994 | -0.38 | -1.49, 0.74 | 0.504 | 0.999 |  |  |  |  |  |  |  |  |  |  |
| c36850 | taurolithocholate 3-sulfate | -0.49 | -1.53, 0.56 | 0.359 | 0.994 | -0.71 | -1.69, 0.27 | 0.158 | 0.999 |  |  |  |  |  |  |  |  |  |  |
| c1605 | ursodeoxycholate | 0.85 | -0.02, 1.73 | 0.056 | 0.962 | 0.86 | 0.03, 1.68 | 0.043 | 0.999 |  |  |  |  |  |  |  |  |  |  |
| c39379 | glycoursodeoxycholate | 0.84 | -0.12, 1.81 | 0.088 | 0.962 | 0.76 | -0.15, 1.67 | 0.104 | 0.999 |  |  |  |  |  |  |  |  |  |  |
| c34093 | hyocholate | 0.73 | -0.61, 2.10 | 0.289 | 0.969 | 0.40 | -0.92, 1.73 | 0.556 | 0.999 |  |  |  |  |  |  |  |  |  |  |
| c42574 | glycohyocholate | 0.80 | -0.41, 2.02 | 0.198 | 0.962 | 0.45 | -0.75, 1.66 | 0.469 | 0.999 |  |  |  |  |  |  |  |  |  |  |
| c32599 | glycocholenate sulfate* | -2.41 | -5.05, 0.31 | 0.083 | 0.962 | -2.36 | -4.86, 0.21 | 0.073 | 0.999 |  |  |  |  |  |  |  |  |  |  |
| c32807 | taurocholenate sulfate* | -1.98 | -3.71,-0.21 | 0.030 | 0.962 | -2.37 | -3.98,-0.73 | 0.005 | 0.711 |  |  |  |  |  |  |  |  |  |  |
| c52975 | glycodeoxycholate 3-sulfate | -0.51 | -1.39, 0.38 | 0.263 | 0.969 | -0.34 | -1.20, 0.53 | 0.442 | 0.999 |  |  |  |  |  |  |  |  |  |  |
|  | **NUCLEOTIDE** |  |  |  |  |  |  |  |  |  |  |  |  |  |  |  |  |  |  |
|  | **Purine Metabolism, (Hypo)Xanthine/Inosine containing** | | |  |  |  |  |  |  |  |  |  |  |  |  |  |  |  |  |
| c1123 | inosine | -0.26 | -0.94, 0.43 | 0.459 | 0.994 | -0.33 | -0.97, 0.32 | 0.321 | 0.999 |  |  |  |  |  |  |  |  |  |  |
| c3127 | hypoxanthine | 0.93 | -1.13, 3.03 | 0.384 | 0.994 | 0.36 | -1.59, 2.34 | 0.724 | 0.999 |  |  |  |  |  |  |  |  |  |  |
| c3147 | xanthine | -0.30 | -3.93, 3.47 | 0.875 | 0.997 | -1.08 | -4.54, 2.51 | 0.553 | 0.999 |  |  |  |  |  |  |  |  |  |  |
| c48351 | N1-methylinosine | 1.41 | -2.73, 5.72 | 0.510 | 0.997 | 2.61 | -1.41, 6.80 | 0.208 | 0.999 |  |  |  |  |  |  |  |  |  |  |
| c1604 | urate | -1.38 | -5.50, 2.92 | 0.525 | 0.997 | -1.01 | -4.98, 3.12 | 0.626 | 0.999 |  |  |  |  |  |  |  |  |  |  |
| c1107 | allantoin | 0.49 | -1.65, 2.69 | 0.656 | 0.997 | 0.20 | -1.85, 2.29 | 0.850 | 0.999 |  |  |  |  |  |  |  |  |  |  |
|  | **Purine Metabolism, Adenine containing** |  |  |  |  |  |  |  |  |  |  |  |  |  |  |  |  |  |  |
| c32342 | AMP | -0.84 | -5.59, 4.15 | 0.737 | 0.997 | -0.84 | -5.40, 3.94 | 0.725 | 0.999 |  |  |  |  |  |  |  |  |  |  |
| c554 | adenine | -1.47 | -4.59, 1.74 | 0.366 | 0.994 | -0.27 | -3.31, 2.86 | 0.863 | 0.999 |  |  |  |  |  |  |  |  |  |  |
| c15650 | 1-methyladenosine | -0.88 | -3.53, 1.84 | 0.521 | 0.997 | -0.98 | -3.46, 1.56 | 0.448 | 0.999 |  |  |  |  |  |  |  |  |  |  |
| c37114 | N6-methyladenosine | 0.03 | -1.74, 1.83 | 0.975 | 0.999 | 0.07 | -1.58, 1.76 | 0.931 | 0.999 |  |  |  |  |  |  |  |  |  |  |
| c35157 | N6-carbamoylthreonyladenosine | -3.26 | -8.60, 2.40 | 0.255 | 0.969 | -1.72 | -6.97, 3.82 | 0.536 | 0.999 |  |  |  |  |  |  |  |  |  |  |
|  | **Purine Metabolism, Guanine containing** |  |  |  |  |  |  |  |  |  |  |  |  |  |  |  |  |  |  |
| c35114 | 7-methylguanine | -6.69 | -11.31,-1.83 | 0.008 | 0.674 | -5.13 | -9.63,-0.39 | 0.036 | 0.999 |  |  |  |  |  |  |  |  |  |  |
| c35137 | N2,N2-dimethylguanosine | -2.23 | -6.84, 2.62 | 0.362 | 0.994 | -1.26 | -5.82, 3.53 | 0.601 | 0.999 |  |  |  |  |  |  |  |  |  |  |
|  | **Pyrimidine Metabolism, Orotate containing** | |  |  |  |  |  |  |  |  |  |  |  |  |  |  |  |  |  |
| c1505 | orotate | -1.77 | -4.55, 1.10 | 0.225 | 0.969 | -0.77 | -3.48, 2.03 | 0.587 | 0.999 |  |  |  |  |  |  |  |  |  |  |
| c35172 | orotidine | -1.38 | -4.93, 2.31 | 0.460 | 0.994 | -0.62 | -4.15, 3.03 | 0.735 | 0.999 |  |  |  |  |  |  |  |  |  |  |
|  | **Pyrimidine Metabolism, Uracil containing** |  |  |  |  |  |  |  |  |  |  |  |  |  |  |  |  |  |  |
| c606 | uridine | -2.31 | -6.76, 2.36 | 0.328 | 0.990 | -2.72 | -6.96, 1.72 | 0.228 | 0.999 |  |  |  |  |  |  |  |  |  |  |
| c33442 | pseudouridine | -3.15 | -8.90, 2.97 | 0.307 | 0.969 | -2.02 | -7.62, 3.91 | 0.497 | 0.999 |  |  |  |  |  |  |  |  |  |  |
| c35136 | 5-methyluridine (ribothymidine) | 2.56 | -2.28, 7.65 | 0.307 | 0.969 | 2.13 | -2.49, 6.97 | 0.374 | 0.999 |  |  |  |  |  |  |  |  |  |  |
| c3155 | 3-ureidopropionate | -0.55 | -3.40, 2.39 | 0.713 | 0.997 | -0.82 | -3.62, 2.06 | 0.573 | 0.999 |  |  |  |  |  |  |  |  |  |  |
| c55 | beta-alanine | -1.46 | -4.88, 2.07 | 0.414 | 0.994 | -1.52 | -4.77, 1.83 | 0.371 | 0.999 |  |  |  |  |  |  |  |  |  |  |
| c37432 | N-acetyl-beta-alanine | -2.09 | -5.54, 1.49 | 0.252 | 0.969 | -1.18 | -4.56, 2.32 | 0.506 | 0.999 |  |  |  |  |  |  |  |  |  |  |
|  | **Pyrimidine Metabolism, Cytidine containing** | |  |  |  |  |  |  |  |  |  |  |  |  |  |  |  |  |  |
| c514 | cytidine | -1.76 | -3.57, 0.09 | 0.064 | 0.962 | -1.02 | -2.76, 0.76 | 0.262 | 0.999 |  |  |  |  |  |  |  |  |  |  |
|  | **Pyrimidine Metabolism, Thymine containing** | |  |  |  |  |  |  |  |  |  |  |  |  |  |  |  |  |  |
| c1418 | 5,6-dihydrothymine | 0.00 | -4.51, 4.72 | 0.999 | 0.999 | -0.48 | -4.82, 4.07 | 0.834 | 0.999 |  |  |  |  |  |  |  |  |  |  |
| c1566 | 3-aminoisobutyrate | -0.88 | -2.98, 1.27 | 0.422 | 0.994 | -0.62 | -2.62, 1.43 | 0.553 | 0.999 |  |  |  |  |  |  |  |  |  |  |
|  | **COFACTORS** |  |  |  |  |  |  |  |  |  |  |  |  |  |  |  |  |  |  |
|  | **Nicotinate and Nicotinamide Metabolism** |  |  |  |  |  |  |  |  |  |  |  |  |  |  |  |  |  |  |
| c1899 | quinolinate | -1.98 | -4.79, 0.92 | 0.180 | 0.962 | -1.14 | -3.92, 1.71 | 0.428 | 0.999 |  |  |  |  |  |  |  |  |  |  |
| c594 | nicotinamide | 3.34 | 1.12, 5.61 | 0.003 | 0.492 | 2.55 | 0.39, 4.75 | 0.021 | 0.876 |  |  |  |  |  |  |  |  |  |  |
| c27665 | 1-methylnicotinamide | 0.70 | -0.82, 2.25 | 0.371 | 0.994 | 0.03 | -1.41, 1.49 | 0.968 | 0.999 |  |  |  |  |  |  |  |  |  |  |
| c32401 | trigonelline (N'-methylnicotinate) | 0.08 | -1.30, 1.48 | 0.908 | 0.997 | 0.00 | -1.35, 1.37 | 0.998 | 0.999 |  |  |  |  |  |  |  |  |  |  |
| c40469 | N1-Methyl-2-pyridone-5-carboxamide | -0.53 | -2.91, 1.91 | 0.667 | 0.997 | -0.34 | -2.66, 2.02 | 0.775 | 0.999 |  |  |  |  |  |  |  |  |  |  |
|  | **Pantothenate and CoA Metabolism** |  |  |  |  |  |  |  |  |  |  |  |  |  |  |  |  |  |  |
| c1508 | pantothenate (Vitamin B5) | -2.65 | -6.38, 1.23 | 0.180 | 0.962 | -0.87 | -4.54, 2.95 | 0.652 | 0.999 |  |  |  |  |  |  |  |  |  |  |
|  | **Ascorbate and Aldarate Metabolism** |  |  |  |  |  |  |  |  |  |  |  |  |  |  |  |  |  |  |
| c27738 | threonate | 0.26 | -1.55, 2.09 | 0.784 | 0.997 | 0.18 | -1.55, 1.95 | 0.837 | 0.999 |  |  |  |  |  |  |  |  |  |  |
| c20694 | oxalate (ethanedioate) | -0.47 | -2.68, 1.79 | 0.679 | 0.997 | -0.76 | -2.90, 1.44 | 0.497 | 0.999 |  |  |  |  |  |  |  |  |  |  |
| c46957 | gulonate* | -2.35 | -4.97, 0.35 | 0.089 | 0.962 | -1.48 | -4.03, 1.13 | 0.264 | 0.999 |  |  |  |  |  |  |  |  |  |  |
|  | **Tocopherol Metabolism** |  |  |  |  |  |  |  |  |  |  |  |  |  |  |  |  |  |  |
| c1561 | alpha-tocopherol | -0.33 | -1.81, 1.17 | 0.666 | 0.997 | -0.01 | -1.42, 1.42 | 0.992 | 0.999 |  |  |  |  |  |  |  |  |  |  |
| c44876 | gamma-CEHC | -0.06 | -1.67, 1.58 | 0.943 | 0.997 | 0.74 | -0.83, 2.33 | 0.362 | 0.999 |  |  |  |  |  |  |  |  |  |  |
|  | **Hemoglobin and Porphyrin Metabolism** |  |  |  |  |  |  |  |  |  |  |  |  |  |  |  |  |  |  |
| c43807 | bilirubin | -0.11 | -0.96, 0.74 | 0.795 | 0.997 | -0.06 | -0.86, 0.76 | 0.894 | 0.999 |  |  |  |  |  |  |  |  |  |  |
| c32586 | bilirubin (E,E)* | 0.07 | -0.83, 0.98 | 0.875 | 0.997 | 0.10 | -0.76, 0.97 | 0.815 | 0.999 |  |  |  |  |  |  |  |  |  |  |
| c47886 | bilirubin (E,Z or Z,E)* | -0.45 | -1.54, 0.66 | 0.428 | 0.994 | -0.38 | -1.42, 0.68 | 0.482 | 0.999 |  |  |  |  |  |  |  |  |  |  |
| c2137 | biliverdin | -0.28 | -1.54, 1.00 | 0.670 | 0.997 | -0.19 | -1.39, 1.02 | 0.759 | 0.999 |  |  |  |  |  |  |  |  |  |  |
|  | **Vitamin A Metabolism** |  |  |  |  |  |  |  |  |  |  |  |  |  |  |  |  |  |  |
| c1806 | retinol (Vitamin A) | -1.70 | -5.10, 1.82 | 0.342 | 0.994 | -0.92 | -4.18, 2.45 | 0.588 | 0.999 |  |  |  |  |  |  |  |  |  |  |
|  | **Vitamin B6 Metabolism** |  |  |  |  |  |  |  |  |  |  |  |  |  |  |  |  |  |  |
| c31555 | pyridoxate | 0.75 | -1.73, 3.29 | 0.558 | 0.997 | 1.70 | -0.75, 4.21 | 0.177 | 0.999 |  |  |  |  |  |  |  |  |  |  |
|  | **XENOBIOTICS** |  |  |  |  |  |  |  |  |  |  |  |  |  |  |  |  |  |  |
|  | **Benzoate Metabolism** |  |  |  |  |  |  |  |  |  |  |  |  |  |  |  |  |  |  |
| c15753 | hippurate | 0.42 | -0.93, 1.78 | 0.546 | 0.997 | 0.65 | -0.68, 1.99 | 0.340 | 0.999 |  |  |  |  |  |  |  |  |  |  |
| c39600 | 3-hydroxyhippurate | 0.00 | -1.17, 1.18 | 0.997 | 0.999 | 0.19 | -0.92, 1.31 | 0.742 | 0.999 |  |  |  |  |  |  |  |  |  |  |
| c35527 | 4-hydroxyhippurate | 1.42 | -0.44, 3.32 | 0.136 | 0.962 | 1.72 | -0.05, 3.52 | 0.058 | 0.999 |  |  |  |  |  |  |  |  |  |  |
| c15778 | benzoate | -0.15 | -2.85, 2.63 | 0.915 | 0.997 | -0.54 | -3.15, 2.15 | 0.692 | 0.999 |  |  |  |  |  |  |  |  |  |  |
| c35320 | catechol sulfate | 1.19 | -0.56, 2.96 | 0.185 | 0.962 | 1.90 | 0.20, 3.62 | 0.030 | 0.965 |  |  |  |  |  |  |  |  |  |  |
| c46111 | guaiacol sulfate | 1.00 | -0.63, 2.66 | 0.232 | 0.969 | 1.95 | 0.38, 3.54 | 0.016 | 0.841 |  |  |  |  |  |  |  |  |  |  |
| c46165 | 3-methyl catechol sulfate (1) | -0.09 | -1.00, 0.82 | 0.841 | 0.997 | 0.12 | -0.76, 1.00 | 0.795 | 0.999 |  |  |  |  |  |  |  |  |  |  |
| c46146 | 4-methylcatechol sulfate | 0.05 | -1.44, 1.57 | 0.946 | 0.997 | 0.44 | -1.00, 1.90 | 0.554 | 0.999 |  |  |  |  |  |  |  |  |  |  |
| c36099 | 4-ethylphenyl sulfate | 0.22 | -0.83, 1.27 | 0.689 | 0.997 | 0.32 | -0.69, 1.35 | 0.533 | 0.999 |  |  |  |  |  |  |  |  |  |  |
| c36098 | 4-vinylphenol sulfate | 0.07 | -0.81, 0.95 | 0.883 | 0.997 | 0.42 | -0.45, 1.30 | 0.347 | 0.999 |  |  |  |  |  |  |  |  |  |  |
| c48763 | 3-methoxycatechol sulfate (1) | 0.68 | -0.23, 1.59 | 0.143 | 0.962 | 0.77 | -0.10, 1.64 | 0.085 | 0.999 |  |  |  |  |  |  |  |  |  |  |
| c48429 | methyl-4-hydroxybenzoate sulfate | 0.30 | -0.32, 0.91 | 0.347 | 0.994 | 0.13 | -0.50, 0.76 | 0.692 | 0.999 |  |  |  |  |  |  |  |  |  |  |
| c36103 | p-cresol sulfate | -0.78 | -2.54, 1.02 | 0.394 | 0.994 | -1.04 | -2.74, 0.69 | 0.240 | 0.999 |  |  |  |  |  |  |  |  |  |  |
| c35635 | 3-(3-hydroxyphenyl)propionate | 0.71 | -0.58, 2.03 | 0.284 | 0.969 | 1.03 | -0.21, 2.29 | 0.106 | 0.999 |  |  |  |  |  |  |  |  |  |  |
| c15749 | 3-phenylpropionate (hydrocinnamate) | -0.09 | -1.23, 1.07 | 0.878 | 0.997 | -0.31 | -1.41, 0.81 | 0.587 | 0.999 |  |  |  |  |  |  |  |  |  |  |
|  | **Xanthine Metabolism** |  |  |  |  |  |  |  |  |  |  |  |  |  |  |  |  |  |  |
| c569 | caffeine | -0.29 | -0.98, 0.40 | 0.411 | 0.994 | -0.06 | -0.72, 0.61 | 0.867 | 0.999 |  |  |  |  |  |  |  |  |  |  |
| c18392 | theobromine | -0.17 | -0.92, 0.59 | 0.661 | 0.997 | 0.18 | -0.56, 0.93 | 0.633 | 0.999 |  |  |  |  |  |  |  |  |  |  |
| c32445 | 3-methylxanthine | -0.16 | -0.96, 0.65 | 0.700 | 0.997 | 0.26 | -0.54, 1.07 | 0.522 | 0.999 |  |  |  |  |  |  |  |  |  |  |
| c34390 | 7-methylxanthine | -0.15 | -0.93, 0.64 | 0.710 | 0.997 | 0.24 | -0.53, 1.00 | 0.544 | 0.999 |  |  |  |  |  |  |  |  |  |  |
| c34424 | 5-acetylamino-6-amino-3-methyluracil | -0.27 | -0.98, 0.45 | 0.465 | 0.994 | 0.00 | -0.69, 0.69 | 0.994 | 0.999 |  |  |  |  |  |  |  |  |  |  |
|  | **Food Component/Plant** |  |  |  |  |  |  |  |  |  |  |  |  |  |  |  |  |  |  |
| c43400 | 2-piperidinone | -0.54 | -1.76, 0.69 | 0.391 | 0.994 | -0.40 | -1.58, 0.80 | 0.513 | 0.999 |  |  |  |  |  |  |  |  |  |  |
| c38276 | 2,3-dihydroxyisovalerate | -0.89 | -1.85, 0.07 | 0.072 | 0.962 | -0.80 | -1.70, 0.12 | 0.090 | 0.999 |  |  |  |  |  |  |  |  |  |  |
| c587 | gluconate | -0.94 | -3.88, 2.10 | 0.542 | 0.997 | -0.92 | -3.71, 1.94 | 0.525 | 0.999 |  |  |  |  |  |  |  |  |  |  |
| c38637 | cinnamoylglycine | -0.28 | -1.16, 0.60 | 0.530 | 0.997 | -0.25 | -1.11, 0.62 | 0.572 | 0.999 |  |  |  |  |  |  |  |  |  |  |
| c37459 | ergothioneine | 0.25 | -1.47, 2.00 | 0.779 | 0.997 | -0.02 | -1.64, 1.63 | 0.985 | 0.999 |  |  |  |  |  |  |  |  |  |  |
| c20699 | erythritol | -3.25 | -7.81, 1.53 | 0.181 | 0.962 | -0.26 | -4.93, 4.64 | 0.916 | 0.999 |  |  |  |  |  |  |  |  |  |  |
| c33009 | homostachydrine* | -1.37 | -3.01, 0.29 | 0.109 | 0.962 | -1.11 | -2.73, 0.53 | 0.186 | 0.999 |  |  |  |  |  |  |  |  |  |  |
| c43374 | indolin-2-one | 0.12 | -1.71, 1.98 | 0.903 | 0.997 | -0.35 | -2.06, 1.39 | 0.691 | 0.999 |  |  |  |  |  |  |  |  |  |  |
| c33935 | piperine | 0.24 | -0.33, 0.82 | 0.405 | 0.994 | 0.02 | -0.55, 0.59 | 0.955 | 0.999 |  |  |  |  |  |  |  |  |  |  |
| c43239 | S-allylcysteine | 1.17 | 0.47, 1.86 | 0.001 | 0.336 | 0.95 | 0.26, 1.64 | 0.007 | 0.711 |  |  |  |  |  |  |  |  |  |  |
| c15336 | tartarate | -0.43 | -1.58, 0.74 | 0.475 | 0.994 | -0.61 | -1.70, 0.49 | 0.279 | 0.999 |  |  |  |  |  |  |  |  |  |  |
| c37181 | 4-allylphenol sulfate | 0.52 | -0.44, 1.49 | 0.289 | 0.969 | 0.51 | -0.40, 1.42 | 0.274 | 0.999 |  |  |  |  |  |  |  |  |  |  |
| c20693 | tartronate (hydroxymalonate) | 1.56 | -0.28, 3.44 | 0.099 | 0.962 | 1.21 | -0.58, 3.03 | 0.188 | 0.999 |  |  |  |  |  |  |  |  |  |  |
|  | **Drug - Topical Agents** |  |  |  |  |  |  |  |  |  |  |  |  |  |  |  |  |  |  |
| c1515 | salicylate | 0.18 | -1.03, 1.41 | 0.771 | 0.997 | -0.24 | -1.41, 0.94 | 0.691 | 0.999 |  |  |  |  |  |  |  |  |  |  |
|  | **Chemical** |  |  |  |  |  |  |  |  |  |  |  |  |  |  |  |  |  |  |
| c46960 | sulfate* | 0.42 | -5.56, 6.78 | 0.893 | 0.997 | -1.98 | -7.60, 3.97 | 0.506 | 0.999 |  |  |  |  |  |  |  |  |  |  |
| c45413 | O-sulfo-L-tyrosine | -3.58 | -8.05, 1.11 | 0.134 | 0.962 | -2.06 | -6.52, 2.61 | 0.382 | 0.999 |  |  |  |  |  |  |  |  |  |  |
| c43266 | 2-aminophenol sulfate | 0.60 | -0.48, 1.69 | 0.280 | 0.969 | 0.68 | -0.36, 1.73 | 0.205 | 0.999 |  |  |  |  |  |  |  |  |  |  |
| c48441 | 4-hydroxychlorothalonil | 0.29 | -1.98, 2.61 | 0.803 | 0.997 | 0.41 | -1.74, 2.61 | 0.709 | 0.999 |  |  |  |  |  |  |  |  |  |  |
| c48448 | 3-hydroxypyridine sulfate | 0.73 | -0.54, 2.01 | 0.262 | 0.969 | 0.73 | -0.48, 1.96 | 0.238 | 0.999 |  |  |  |  |  |  |  |  |  |  |
| c48698 | 6-hydroxyindole sulfate | -0.81 | -3.10, 1.54 | 0.498 | 0.994 | -1.45 | -3.61, 0.76 | 0.198 | 0.999 |  |  |  |  |  |  |  |  |  |  |
| c53231 | thioproline | -0.16 | -1.40, 1.10 | 0.808 | 0.997 | -0.19 | -1.38, 1.01 | 0.753 | 0.999 |  |  |  |  |  |  |  |  |  |  |
|  | **UNKNOWN** |  |  |  |  |  |  |  |  |  |  |  |  |  |  |  |  |  |  |
| c32578 | X - 11261 | -0.46 | -2.34, 1.46 | 0.640 | 0.997 | -0.30 | -2.10, 1.54 | 0.751 | 0.999 |  |  |  |  |  |  |  |  |  |  |
| c33132 | X - 11787 | -5.00 | -10.20, 0.51 | 0.076 | 0.962 | -4.08 | -9.29, 1.42 | 0.145 | 0.999 |  |  |  |  |  |  |  |  |  |  |
| c33140 | X - 11795 | -1.13 | -3.44, 1.24 | 0.348 | 0.994 | -1.29 | -3.47, 0.94 | 0.256 | 0.999 |  |  |  |  |  |  |  |  |  |  |
| c46259 | X - 21258 | 0.72 | -0.33, 1.78 | 0.180 | 0.962 | 0.76 | -0.23, 1.77 | 0.136 | 0.999 |  |  |  |  |  |  |  |  |  |  |
| c46266 | X - 15486 | -0.41 | -1.69, 0.89 | 0.534 | 0.997 | -0.51 | -1.71, 0.71 | 0.411 | 0.999 |  |  |  |  |  |  |  |  |  |  |
| c46283 | X - 15461 | -3.15 | -5.95,-0.25 | 0.035 | 0.962 | -3.46 | -6.10,-0.74 | 0.014 | 0.841 |  |  |  |  |  |  |  |  |  |  |
| c46295 | X - 21286 | -0.08 | -1.99, 1.87 | 0.936 | 0.997 | -0.14 | -1.99, 1.75 | 0.885 | 0.999 |  |  |  |  |  |  |  |  |  |  |
| c46347 | X - 11381 | 1.30 | -1.11, 3.77 | 0.294 | 0.969 | 1.18 | -1.13, 3.54 | 0.321 | 0.999 |  |  |  |  |  |  |  |  |  |  |
| c46354 | X - 21310 | -1.56 | -4.57, 1.55 | 0.323 | 0.986 | -2.11 | -4.96, 0.82 | 0.158 | 0.999 |  |  |  |  |  |  |  |  |  |  |
| c46356 | X - 21312 | 0.36 | -0.68, 1.41 | 0.502 | 0.995 | 0.65 | -0.35, 1.66 | 0.205 | 0.999 |  |  |  |  |  |  |  |  |  |  |
| c46363 | X - 21319 | -0.08 | -1.59, 1.44 | 0.914 | 0.997 | 0.06 | -1.37, 1.51 | 0.936 | 0.999 |  |  |  |  |  |  |  |  |  |  |
| c46364 | X - 12847 | 0.44 | -0.35, 1.24 | 0.275 | 0.969 | 0.42 | -0.32, 1.18 | 0.268 | 0.999 |  |  |  |  |  |  |  |  |  |  |
| c46384 | X - 21339 | 1.44 | -0.70, 3.62 | 0.191 | 0.962 | 1.06 | -1.00, 3.16 | 0.318 | 0.999 |  |  |  |  |  |  |  |  |  |  |
| c46390 | X - 11308 | 2.29 | -0.32, 4.96 | 0.088 | 0.962 | 1.51 | -1.04, 4.12 | 0.251 | 0.999 |  |  |  |  |  |  |  |  |  |  |
| c46398 | X - 21353 | -1.21 | -3.00, 0.62 | 0.194 | 0.962 | -0.88 | -2.62, 0.89 | 0.328 | 0.999 |  |  |  |  |  |  |  |  |  |  |
| c46409 | X - 21364 | 0.33 | -2.24, 2.97 | 0.805 | 0.997 | 0.51 | -1.94, 3.02 | 0.687 | 0.999 |  |  |  |  |  |  |  |  |  |  |
| c46417 | X - 13866 | 0.00 | -1.73, 1.76 | 0.999 | 0.999 | 0.13 | -1.51, 1.80 | 0.877 | 0.999 |  |  |  |  |  |  |  |  |  |  |
| c46428 | X - 21383 | -0.70 | -1.99, 0.62 | 0.298 | 0.969 | -0.53 | -1.82, 0.77 | 0.423 | 0.999 |  |  |  |  |  |  |  |  |  |  |
| c46460 | X - 11444 | -1.31 | -4.38, 1.86 | 0.416 | 0.994 | -0.87 | -3.90, 2.26 | 0.584 | 0.999 |  |  |  |  |  |  |  |  |  |  |
| c46466 | X - 11843 | -0.17 | -0.76, 0.43 | 0.582 | 0.997 | -0.22 | -0.79, 0.34 | 0.439 | 0.999 |  |  |  |  |  |  |  |  |  |  |
| c46486 | X - 21441 | -0.98 | -2.45, 0.51 | 0.198 | 0.962 | -0.88 | -2.30, 0.56 | 0.230 | 0.999 |  |  |  |  |  |  |  |  |  |  |
| c46507 | X - 11850 | -0.19 | -0.83, 0.46 | 0.563 | 0.997 | -0.19 | -0.81, 0.43 | 0.541 | 0.999 |  |  |  |  |  |  |  |  |  |  |
| c46510 | X - 12544 | 0.80 | -0.55, 2.18 | 0.248 | 0.969 | 0.87 | -0.42, 2.17 | 0.188 | 0.999 |  |  |  |  |  |  |  |  |  |  |
| c46512 | X - 21467 | -1.54 | -3.26, 0.21 | 0.085 | 0.962 | -0.98 | -2.68, 0.74 | 0.264 | 0.999 |  |  |  |  |  |  |  |  |  |  |
| c46515 | X - 21470 | -0.17 | -1.47, 1.15 | 0.801 | 0.997 | -0.19 | -1.41, 1.06 | 0.769 | 0.999 |  |  |  |  |  |  |  |  |  |  |
| c46516 | X - 21471 | -1.29 | -2.74, 0.18 | 0.087 | 0.962 | -0.74 | -2.14, 0.68 | 0.305 | 0.999 |  |  |  |  |  |  |  |  |  |  |
| c46517 | X - 16946 | -0.60 | -2.23, 1.06 | 0.478 | 0.994 | -0.62 | -2.15, 0.93 | 0.432 | 0.999 |  |  |  |  |  |  |  |  |  |  |
| c46521 | X - 11852 | 0.49 | -0.14, 1.13 | 0.129 | 0.962 | 0.48 | -0.13, 1.09 | 0.126 | 0.999 |  |  |  |  |  |  |  |  |  |  |
| c46590 | X - 07765 | 0.13 | -0.88, 1.15 | 0.799 | 0.997 | 0.20 | -0.75, 1.17 | 0.677 | 0.999 |  |  |  |  |  |  |  |  |  |  |
| c46592 | X - 11299 | 0.24 | -0.41, 0.89 | 0.474 | 0.994 | 0.09 | -0.52, 0.71 | 0.770 | 0.999 |  |  |  |  |  |  |  |  |  |  |
| c46594 | X - 11372 | 3.93 | 0.63, 7.33 | 0.020 | 0.962 | 3.72 | 0.53, 7.01 | 0.023 | 0.887 |  |  |  |  |  |  |  |  |  |  |
| c46601 | X - 11470 | -0.47 | -2.64, 1.75 | 0.675 | 0.997 | 0.05 | -2.12, 2.27 | 0.965 | 0.999 |  |  |  |  |  |  |  |  |  |  |
| c46602 | X - 11478 | -0.28 | -1.96, 1.43 | 0.744 | 0.997 | 0.06 | -1.54, 1.68 | 0.944 | 0.999 |  |  |  |  |  |  |  |  |  |  |
| c46607 | X - 11849 | 0.16 | -0.50, 0.82 | 0.637 | 0.997 | 0.08 | -0.54, 0.70 | 0.808 | 0.999 |  |  |  |  |  |  |  |  |  |  |
| c46608 | X - 11880 | 1.69 | -1.19, 4.66 | 0.254 | 0.969 | 1.46 | -1.33, 4.33 | 0.310 | 0.999 |  |  |  |  |  |  |  |  |  |  |
| c46613 | X - 12216 | -0.05 | -1.08, 1.00 | 0.932 | 0.997 | -0.27 | -1.25, 0.72 | 0.596 | 0.999 |  |  |  |  |  |  |  |  |  |  |
| c46616 | X - 12411 | -0.10 | -1.39, 1.20 | 0.879 | 0.997 | 0.12 | -1.12, 1.37 | 0.853 | 0.999 |  |  |  |  |  |  |  |  |  |  |
| c46623 | X - 12729 | -0.05 | -1.43, 1.34 | 0.940 | 0.997 | -0.38 | -1.75, 1.00 | 0.585 | 0.999 |  |  |  |  |  |  |  |  |  |  |
| c46624 | X - 12798 | 0.19 | -2.11, 2.54 | 0.876 | 0.997 | 0.23 | -2.05, 2.57 | 0.844 | 0.999 |  |  |  |  |  |  |  |  |  |  |
| c46632 | X - 14056 | -0.20 | -1.47, 1.08 | 0.758 | 0.997 | -0.18 | -1.40, 1.06 | 0.777 | 0.999 |  |  |  |  |  |  |  |  |  |  |
| c46633 | X - 12844 | -2.16 | -5.68, 1.49 | 0.244 | 0.969 | -1.10 | -4.50, 2.41 | 0.534 | 0.999 |  |  |  |  |  |  |  |  |  |  |
| c46636 | X - 12849 | 0.41 | -0.37, 1.19 | 0.304 | 0.969 | 0.04 | -0.71, 0.79 | 0.926 | 0.999 |  |  |  |  |  |  |  |  |  |  |
| c46640 | X - 15469 | -0.68 | -2.82, 1.50 | 0.538 | 0.997 | -0.66 | -2.71, 1.43 | 0.533 | 0.999 |  |  |  |  |  |  |  |  |  |  |
| c46645 | X - 13728 | -0.12 | -0.91, 0.66 | 0.757 | 0.997 | 0.30 | -0.48, 1.09 | 0.448 | 0.999 |  |  |  |  |  |  |  |  |  |  |
| c46646 | X - 13835 | -0.40 | -1.40, 0.60 | 0.431 | 0.994 | -0.31 | -1.27, 0.66 | 0.529 | 0.999 |  |  |  |  |  |  |  |  |  |  |
| c46657 | X - 14939 | -0.30 | -2.22, 1.66 | 0.764 | 0.997 | -0.42 | -2.28, 1.48 | 0.664 | 0.999 |  |  |  |  |  |  |  |  |  |  |
| c46661 | X - 15245 | -0.20 | -0.95, 0.55 | 0.604 | 0.997 | -0.01 | -0.73, 0.71 | 0.970 | 0.999 |  |  |  |  |  |  |  |  |  |  |
| c46662 | X - 15492 | -1.47 | -3.28, 0.36 | 0.117 | 0.962 | -1.05 | -2.79, 0.71 | 0.243 | 0.999 |  |  |  |  |  |  |  |  |  |  |
| c46666 | X - 15728 | 0.40 | -0.45, 1.25 | 0.362 | 0.994 | 0.42 | -0.37, 1.22 | 0.298 | 0.999 |  |  |  |  |  |  |  |  |  |  |
| c46673 | X - 16576 | 3.06 | 1.28, 4.88 | 0.001 | 0.336 | 2.91 | 1.25, 4.60 | 0.001 | 0.395 |  |  |  |  |  |  |  |  |  |  |
| c46674 | X - 21607 | -0.29 | -1.38, 0.80 | 0.601 | 0.997 | -0.59 | -1.61, 0.44 | 0.259 | 0.999 |  |  |  |  |  |  |  |  |  |  |
| c46681 | X - 16935 | 0.15 | -1.39, 1.71 | 0.850 | 0.997 | -0.23 | -1.75, 1.31 | 0.767 | 0.999 |  |  |  |  |  |  |  |  |  |  |
| c46683 | X - 16944 | -0.50 | -2.23, 1.26 | 0.574 | 0.997 | -0.50 | -2.15, 1.19 | 0.562 | 0.999 |  |  |  |  |  |  |  |  |  |  |
| c46685 | X - 16964 | -0.71 | -2.44, 1.05 | 0.432 | 0.994 | -0.49 | -2.16, 1.20 | 0.570 | 0.999 |  |  |  |  |  |  |  |  |  |  |
| c46690 | X - 18901 | -0.27 | -1.82, 1.30 | 0.733 | 0.997 | 0.03 | -1.45, 1.54 | 0.965 | 0.999 |  |  |  |  |  |  |  |  |  |  |
| c46695 | X - 18913 | 0.80 | -2.08, 3.77 | 0.590 | 0.997 | 1.00 | -1.75, 3.82 | 0.481 | 0.999 |  |  |  |  |  |  |  |  |  |  |
| c46700 | X - 18922 | 0.19 | -1.38, 1.78 | 0.817 | 0.997 | -0.31 | -1.86, 1.26 | 0.697 | 0.999 |  |  |  |  |  |  |  |  |  |  |
| c46701 | X - 19141 | 0.41 | -1.48, 2.35 | 0.672 | 0.997 | 1.10 | -0.71, 2.95 | 0.237 | 0.999 |  |  |  |  |  |  |  |  |  |  |
| c46710 | X - 17690 | 0.54 | -0.24, 1.32 | 0.178 | 0.962 | 0.56 | -0.18, 1.32 | 0.140 | 0.999 |  |  |  |  |  |  |  |  |  |  |
| c46902 | X - 21733 | 0.20 | -1.11, 1.53 | 0.768 | 0.997 | -0.34 | -1.58, 0.92 | 0.594 | 0.999 |  |  |  |  |  |  |  |  |  |  |
| c46905 | X - 21736 | -0.17 | -1.90, 1.60 | 0.853 | 0.997 | 0.15 | -1.51, 1.84 | 0.860 | 0.999 |  |  |  |  |  |  |  |  |  |  |
| c46909 | X - 21740 | -0.71 | -2.48, 1.09 | 0.441 | 0.994 | -0.74 | -2.47, 1.02 | 0.411 | 0.999 |  |  |  |  |  |  |  |  |  |  |
| c46932 | X - 12104 | 3.72 | 1.07, 6.44 | 0.006 | 0.614 | 3.81 | 1.24, 6.45 | 0.004 | 0.711 |  |  |  |  |  |  |  |  |  |  |
| c46972 | X - 21796 | 0.69 | -1.83, 3.27 | 0.596 | 0.997 | 0.19 | -2.24, 2.67 | 0.882 | 0.999 |  |  |  |  |  |  |  |  |  |  |
| c46977 | X - 15503 | -2.05 | -5.10, 1.09 | 0.200 | 0.962 | -1.80 | -4.71, 1.19 | 0.237 | 0.999 |  |  |  |  |  |  |  |  |  |  |
| c46997 | X - 12822 | -0.29 | -2.23, 1.68 | 0.770 | 0.997 | 0.05 | -1.82, 1.96 | 0.958 | 0.999 |  |  |  |  |  |  |  |  |  |  |
| c47006 | X - 21829 | 0.28 | -0.62, 1.19 | 0.542 | 0.997 | 0.18 | -0.71, 1.08 | 0.693 | 0.999 |  |  |  |  |  |  |  |  |  |  |
| c47013 | X - 16570 | -0.20 | -1.83, 1.45 | 0.810 | 0.997 | 0.40 | -1.17, 2.00 | 0.619 | 0.999 |  |  |  |  |  |  |  |  |  |  |
| c47301 | X - 18887 | -0.11 | -2.27, 2.09 | 0.922 | 0.997 | -0.25 | -2.37, 1.93 | 0.823 | 0.999 |  |  |  |  |  |  |  |  |  |  |
| c47417 | X - 22162 | 0.47 | -3.20, 4.28 | 0.804 | 0.997 | 1.85 | -1.88, 5.73 | 0.338 | 0.999 |  |  |  |  |  |  |  |  |  |  |
| c47439 | X - 13507 | -1.47 | -3.86, 0.98 | 0.239 | 0.969 | -1.29 | -3.61, 1.09 | 0.286 | 0.999 |  |  |  |  |  |  |  |  |  |  |
| c47642 | X - 12101 | -0.08 | -1.20, 1.04 | 0.884 | 0.997 | -0.23 | -1.28, 0.84 | 0.678 | 0.999 |  |  |  |  |  |  |  |  |  |  |
| c47664 | X - 13658 | 0.40 | -0.83, 1.65 | 0.528 | 0.997 | 0.06 | -1.15, 1.28 | 0.925 | 0.999 |  |  |  |  |  |  |  |  |  |  |
| c47670 | X - 18899 | 1.59 | -0.26, 3.47 | 0.094 | 0.962 | 1.34 | -0.46, 3.18 | 0.148 | 0.999 |  |  |  |  |  |  |  |  |  |  |
| c47671 | X - 18921 | -0.54 | -2.27, 1.21 | 0.541 | 0.997 | -0.82 | -2.45, 0.83 | 0.328 | 0.999 |  |  |  |  |  |  |  |  |  |  |
| c47673 | X - 19299 | -0.09 | -0.92, 0.74 | 0.825 | 0.997 | -0.08 | -0.86, 0.70 | 0.833 | 0.999 |  |  |  |  |  |  |  |  |  |  |
| c47687 | X - 12100 | -0.25 | -2.86, 2.43 | 0.856 | 0.997 | -0.71 | -3.21, 1.86 | 0.589 | 0.999 |  |  |  |  |  |  |  |  |  |  |
| c47708 | X - 12283 | -0.35 | -1.63, 0.95 | 0.599 | 0.997 | -0.34 | -1.58, 0.92 | 0.596 | 0.999 |  |  |  |  |  |  |  |  |  |  |
| c47783 | X - 22519 | -1.30 | -3.61, 1.06 | 0.279 | 0.969 | -1.41 | -3.61, 0.84 | 0.218 | 0.999 |  |  |  |  |  |  |  |  |  |  |
| c47802 | X - 16397 | 0.42 | -1.01, 1.87 | 0.565 | 0.997 | 0.17 | -1.18, 1.55 | 0.804 | 0.999 |  |  |  |  |  |  |  |  |  |  |
| c47804 | X - 16580 | 0.17 | -1.73, 2.10 | 0.865 | 0.997 | -0.07 | -1.87, 1.77 | 0.942 | 0.999 |  |  |  |  |  |  |  |  |  |  |
| c47872 | X - 17340 | -2.23 | -4.12,-0.30 | 0.025 | 0.962 | -1.70 | -3.51, 0.14 | 0.071 | 0.999 |  |  |  |  |  |  |  |  |  |  |
| c47905 | X - 12026 | -2.59 | -5.63, 0.55 | 0.106 | 0.962 | -1.32 | -4.32, 1.78 | 0.402 | 0.999 |  |  |  |  |  |  |  |  |  |  |
| c47929 | X - 12707 | -1.14 | -3.03, 0.79 | 0.247 | 0.969 | -0.86 | -2.66, 0.99 | 0.362 | 0.999 |  |  |  |  |  |  |  |  |  |  |
| c47959 | X - 13553 | -0.86 | -3.26, 1.60 | 0.492 | 0.994 | -0.22 | -2.50, 2.11 | 0.853 | 0.999 |  |  |  |  |  |  |  |  |  |  |
| c48001 | X - 17351 | -0.13 | -1.45, 1.20 | 0.846 | 0.997 | -0.02 | -1.32, 1.31 | 0.980 | 0.999 |  |  |  |  |  |  |  |  |  |  |
| c48047 | X - 18886 | 0.08 | -2.19, 2.41 | 0.943 | 0.997 | 0.09 | -2.07, 2.31 | 0.933 | 0.999 |  |  |  |  |  |  |  |  |  |  |
| c48076 | X - 22771 | -0.61 | -2.24, 1.05 | 0.474 | 0.994 | -0.50 | -2.07, 1.10 | 0.541 | 0.999 |  |  |  |  |  |  |  |  |  |  |
| c49463 | X - 23587 | 0.02 | -1.18, 1.22 | 0.980 | 0.999 | -0.14 | -1.28, 1.01 | 0.810 | 0.999 |  |  |  |  |  |  |  |  |  |  |
| c49466 | X - 23590 | -1.58 | -4.39, 1.32 | 0.284 | 0.969 | -0.28 | -3.05, 2.57 | 0.848 | 0.999 |  |  |  |  |  |  |  |  |  |  |
| c49469 | X - 23593 | -2.41 | -5.22, 0.48 | 0.103 | 0.962 | -2.27 | -4.96, 0.49 | 0.108 | 0.999 |  |  |  |  |  |  |  |  |  |  |
| c49515 | X - 23639 | -0.45 | -3.82, 3.03 | 0.797 | 0.997 | 0.29 | -2.92, 3.61 | 0.861 | 0.999 |  |  |  |  |  |  |  |  |  |  |
| c49517 | X - 23641 | -0.42 | -1.95, 1.14 | 0.597 | 0.997 | 0.09 | -1.42, 1.62 | 0.908 | 0.999 |  |  |  |  |  |  |  |  |  |  |
| c49521 | X - 23644 | -0.04 | -0.84, 0.77 | 0.924 | 0.997 | 0.03 | -0.73, 0.81 | 0.931 | 0.999 |  |  |  |  |  |  |  |  |  |  |
| c49536 | X - 23659 | -0.73 | -2.19, 0.75 | 0.335 | 0.994 | -0.62 | -2.02, 0.80 | 0.392 | 0.999 |  |  |  |  |  |  |  |  |  |  |
| c49557 | X - 23680 | -1.06 | -2.86, 0.77 | 0.257 | 0.969 | -0.95 | -2.67, 0.81 | 0.291 | 0.999 |  |  |  |  |  |  |  |  |  |  |
| c49592 | X - 11315 | -0.54 | -3.74, 2.77 | 0.747 | 0.997 | -1.78 | -4.82, 1.35 | 0.263 | 0.999 |  |  |  |  |  |  |  |  |  |  |
| c49637 | X - 23739 | 1.56 | -0.50, 3.67 | 0.141 | 0.962 | 0.94 | -1.06, 2.97 | 0.361 | 0.999 |  |  |  |  |  |  |  |  |  |  |
| c49679 | X - 23780 | 0.64 | -0.85, 2.17 | 0.402 | 0.994 | 0.37 | -1.05, 1.81 | 0.615 | 0.999 |  |  |  |  |  |  |  |  |  |  |
| c49681 | X - 23782 | -0.02 | -2.33, 2.34 | 0.986 | 0.999 | 0.46 | -1.72, 2.69 | 0.679 | 0.999 |  |  |  |  |  |  |  |  |  |  |
| c49883 | X - 23974 | 0.14 | -1.76, 2.07 | 0.889 | 0.997 | -0.49 | -2.29, 1.34 | 0.596 | 0.999 |  |  |  |  |  |  |  |  |  |  |
| c52483 | X - 24295 | -0.03 | -0.63, 0.59 | 0.934 | 0.997 | -0.12 | -0.70, 0.46 | 0.675 | 0.999 |  |  |  |  |  |  |  |  |  |  |
| c52524 | X - 24328 | -0.21 | -2.10, 1.71 | 0.828 | 0.997 | -0.36 | -2.16, 1.47 | 0.695 | 0.999 |  |  |  |  |  |  |  |  |  |  |
| c52533 | X - 24337 | 0.00 | -1.80, 1.83 | 0.999 | 0.999 | 0.38 | -1.32, 2.12 | 0.662 | 0.999 |  |  |  |  |  |  |  |  |  |  |
| c52636 | X - 24422 | 0.79 | -1.66, 3.30 | 0.532 | 0.997 | 1.35 | -1.04, 3.79 | 0.273 | 0.999 |  |  |  |  |  |  |  |  |  |  |
| c52665 | X - 24435 | -2.10 | -5.67, 1.61 | 0.265 | 0.969 | -2.93 | -6.27, 0.52 | 0.097 | 0.999 |  |  |  |  |  |  |  |  |  |  |
| c52772 | X - 24455 | 1.35 | -1.10, 3.87 | 0.285 | 0.969 | 0.94 | -1.40, 3.34 | 0.435 | 0.999 |  |  |  |  |  |  |  |  |  |  |
| c52773 | X - 24456 | 0.59 | -1.04, 2.24 | 0.483 | 0.994 | 0.50 | -1.06, 2.08 | 0.534 | 0.999 |  |  |  |  |  |  |  |  |  |  |
| c52865 | X - 24544 | 0.22 | -1.34, 1.80 | 0.786 | 0.997 | 0.25 | -1.21, 1.74 | 0.735 | 0.999 |  |  |  |  |  |  |  |  |  |  |
| c52867 | X - 24546 | -0.02 | -1.50, 1.49 | 0.983 | 0.999 | -0.37 | -1.80, 1.09 | 0.619 | 0.999 |  |  |  |  |  |  |  |  |  |  |
| c52877 | X - 24556 | -0.97 | -2.79, 0.89 | 0.308 | 0.969 | -0.38 | -2.16, 1.42 | 0.675 | 0.999 |  |  |  |  |  |  |  |  |  |  |
| c52909 | X - 24588 | -3.52 | -5.73,-1.25 | 0.003 | 0.492 | -2.84 | -5.05,-0.57 | 0.015 | 0.841 |  |  |  |  |  |  |  |  |  |  |
| c53127 | X - 24699 | -3.64 | -8.23, 1.19 | 0.139 | 0.962 | -3.35 | -7.79, 1.31 | 0.158 | 0.999 |  |  |  |  |  |  |  |  |  |  |
| c54840 | X - 24812 | -0.53 | -2.53, 1.51 | 0.609 | 0.997 | -0.62 | -2.52, 1.31 | 0.527 | 0.999 |  |  |  |  |  |  |  |  |  |  |
| c57714 | X - 24947 | 0.15 | -1.23, 1.55 | 0.835 | 0.997 | 0.12 | -1.20, 1.45 | 0.862 | 0.999 |  |  |  |  |  |  |  |  |  |  |
| c57716 | X - 24949 | -0.32 | -1.97, 1.36 | 0.710 | 0.997 | -0.70 | -2.34, 0.96 | 0.408 | 0.999 |  |  |  |  |  |  |  |  |  |  |
| c57720 | X - 24953 | 1.10 | -1.23, 3.48 | 0.359 | 0.994 | 1.03 | -1.24, 3.35 | 0.376 | 0.999 |  |  |  |  |  |  |  |  |  |  |
| c62636 | X - 25343 | -0.01 | -0.97, 0.97 | 0.991 | 0.999 | 0.00 | -0.92, 0.93 | 0.997 | 0.999 |  |  |  |  |  |  |  |  |  |  |
| c62664 | X - 25371 | -1.14 | -5.22, 3.11 | 0.594 | 0.997 | 2.83 | -1.60, 7.45 | 0.216 | 0.999 |  |  |  |  |  |  |  |  |  |  |
| c62716 | X - 25419 | 0.07 | -1.10, 1.25 | 0.913 | 0.997 | -0.06 | -1.18, 1.07 | 0.918 | 0.999 |  |  |  |  |  |  |  |  |  |  |
| c62717 | X - 25420 | 2.33 | 0.06, 4.65 | 0.046 | 0.962 | 2.35 | 0.22, 4.53 | 0.032 | 0.965 |  |  |  |  |  |  |  |  |  |  |
| c62719 | X - 25422 | 0.90 | -0.74, 2.58 | 0.285 | 0.969 | 1.10 | -0.50, 2.72 | 0.182 | 0.999 |  |  |  |  |  |  |  |  |  |  |
| c62963 | X - 25519 | 0.69 | -0.59, 1.98 | 0.297 | 0.969 | 0.50 | -0.72, 1.74 | 0.425 | 0.999 |  |  |  |  |  |  |  |  |  |  |
| c62964 | X - 25520 | -0.83 | -2.19, 0.55 | 0.238 | 0.969 | -0.43 | -1.77, 0.92 | 0.530 | 0.999 |  |  |  |  |  |  |  |  |  |  |
| c63560 | X - 25790 | -0.21 | -2.77, 2.42 | 0.875 | 0.997 | -0.63 | -3.05, 1.86 | 0.618 | 0.999 |  |  |  |  |  |  |  |  |  |  |
| c63908 | X - 25957 | 1.26 | -0.82, 3.37 | 0.239 | 0.969 | 0.83 | -1.17, 2.87 | 0.419 | 0.999 |  |  |  |  |  |  |  |  |  |  |

^1^ Estimates are from robust mixed effects multivariable linear regression models including ADBV as the dependent variable; childhood fixed effects – ln(metabolite level) (continuous), BMI z-score (continuous), age at BMI measurement (continuous), treatment group assignment; adult fixed effects - BMI and BMI^2^ at breast density (continuous); and DISC clinic as a random effect.

^2^ Estimates are from robust mixed effects multivariable linear regression models including ADBV as the dependent variable; childhood fixed effects – ln(metabolite level) (continuous), BMI z-score (continuous), age at BMI measurement (continuous), treatment group assignment, race (white/non-white), and menstrual cycle phase at blood collection (premenarche/luteal/follicular/unknown); adult fixed effects - BMI and BMI^2^ at breast density (continuous), college graduate (yes/no), duration hormone use (continuous), number live births (0/1+), current smoker (yes/no); and DISC clinic as a random effect.
